# The SGLT2 inhibitor canagliflozin in heart failure: the CHIEF-HF remote, patient-centered randomized trial

**DOI:** 10.1038/s41591-022-01703-8

**Published:** 2022-02-28

**Authors:** John A. Spertus, Mary C. Birmingham, Michael Nassif, C. V. Damaraju, Antonio Abbate, Javed Butler, David E. Lanfear, Ildiko Lingvay, Mikhail N. Kosiborod, James L. Januzzi

**Affiliations:** 1 Saint Luke’s Mid America Heart Institute/University of Missouri-Kansas City, Kansas City, MO USA; 2 Janssen Scientific Affairs, LLC, Titusville, NJ USA; 3 Janssen Research & Development, LLC, Raritan, NJ USA; 4 Wright Center for Clinical and Translation Research and Pauley Heart Center, Virginia Commonwealth University, Richmond, VA USA; 5 University of Mississippi Medical Center, Jackson, MS USA; 6 Henry Ford Health System, Detroit, MI USA; 7 University of Texas Southwestern Medical Center, Dallas, TX USA; 8 Massachusetts General Hospital, Harvard Medical School and Baim Institute for Clinical Research, Boston, MA USA

**Keywords:** Heart failure, Randomized controlled trials

## Abstract

Large traditional clinical trials suggest that sodium-glucose co-transporter 2 inhibitors improve symptoms in patients with heart failure and reduced ejection fraction (HFrEF) and in patients with heart failure and preserved ejection fraction (HFpEF). In the midst of the Coronavirus Disease 2019 pandemic, we sought to confirm these benefits in a new type of trial that was patient centered and conducted in a completely remote fashion. In the CHIEF-HF trial (NCT04252287), 476 participants with HF, regardless of EF or diabetes status, were randomized to 100 mg of canagliflozin or placebo. Enrollment was stopped early due to shifting sponsor priorities, without unblinding. The primary outcome was change in the Kansas City Cardiomyopathy Questionnaire Total Symptom Score (KCCQ TSS) at 12 weeks. The 12-week change in KCCQ TSS was 4.3 points (95% confidence interval, 0.8–7.8; *P* = 0.016) higher with canagliflozin than with placebo, meeting the primary endpoint. Similar effects were observed in participants with HFpEF and in those with HFrEF and in participants with and without diabetes, demonstrating that canagliflozin significantly improves symptom burden in HF, regardless of EF or diabetes status. This randomized, double-blind trial, conducted without in-person interactions between doctor and patient, can serve as a model for future all-virtual clinical trials.

## Abstract (web summary)

The potential of all-virtual clinical trials in cardiology is shown by the CHIEF-HF trial, conducted in the midst of the COVID-19 pandemic, which found that an SGLT2 inhibitor can alleviate heart failure symptoms in patients irrespective of ejection fraction or diabetes status.

## Main

The costs of conducting clinical trials have risen substantially over time, leading to calls for novel study designs to generate the evidence needed to guide care^1–3^. A large component (up to 50%) of these costs is the burden of data collection on sites, which have nearly quadrupled from 1990 to 2010 (ref. ^4^). The ongoing Coronavirus Disease 2019 (COVID-19) global pandemic further highlighted the challenges of traditional study designs that depend on in-person visits and resource-intense data acquisition and verification. In response to the growing demands to make clinical trials more pragmatic, novel study designs have been implemented, from leveraging existing registries for data collection^5^ to the use of electronic health records to identify, enroll, randomize and follow-up eligible patients^6,7^. Although the innovation of eliminating in-person clinical trial visits has been proposed, it has not, to our knowledge, been tested on a large scale.

Heart failure (HF) is a common, chronic condition with a high burden of debilitating symptoms, physical limitations and poor quality of life. Many approved HF therapies have neutral or modest effects on symptoms, making treatments that address this key goal of management a critical unmet need. Sodium-glucose co-transporter 2 inhibitors (SGLT2is) not only reduce cardiovascular death and hospitalization in patients with HFrEF and in patients with HFpEF, but they have also recently been shown to improve health status (symptoms, function and quality of life)^8–16^. Given the importance of symptoms, function and quality of life to patients, confirming these health status benefits across the spectrum of HF, and in patients with and without diabetes, can underscore the importance of increasing their use in routine care.

Addressing the call both for more efficient and cost-effective clinical trials and to confirm the health status benefits of SGLT2is in patients with HF of all types, Canagliflozin: Impact on Health Status, Quality of Life and Functional Status in Heart Failure (CHIEF-HF) was designed to be a completely decentralized trial without any in-person interaction with participants.

## Results

### Study design

In light of regulatory shifts that have increased the priority of patient-reported outcomes in approving new medications^17^, and the recent qualification of the Kansas City Cardiomyopathy Questionnaire (KCCQ) as a clinical outcome assessment^18^, CHIEF-HF was designed to test the primary hypothesis that canagliflozin, compared to placebo, would improve the KCCQ Total Symptom Score (TSS) at 12 weeks. Given the ability to collect the KCCQ via smart devices, CHIEF-HF was designed as a completely decentralized, virtual (that is, no in-person visits) study with direct engagement of patients through a study website, electronic informed consent, direct home delivery of study medication, completion of the primary endpoint by a mobile application and a Fitbit to monitor activity. To ensure protection of participants’ personal health information (PHI), the mobile application was compliant with 21 CFR part 11 with access only by study participants; all potential sources of PHI collection were disclosed in the consent process; PHI was firewalled from the sponsor and contract research organization; and insurance claim information was presented in de-identified formats. Eighteen health systems were selected to participate (Supplementary Note 1) and agreed to the use of a central institutional review board (Advarra). Eligible patients were centrally randomized 1:1 using a computer-generated randomization schedule, stratified by the type of heart failure (HFrEF or HFpEF), to either canagliflozin 100 mg daily or matching placebo for 12 weeks, which was shipped directly to participants. Further details regarding randomization are provided in the study protocol as supplementary materials. The study app asked patients, each week, to report the number of days they took the study drug. The original sample size was to include 1,900 randomized participants, but shifting priorities of the sponsor (Janssen Scientific Affairs) led to administrative closing of the study by the sponsor to enrollment on 12 February 2021. This decision was made without an interim analysis of unblinded data or recalculation of sample sizes and power and was in consultation with the Academic Steering Committee.

### Baseline characteristics

**Fig. 1:**
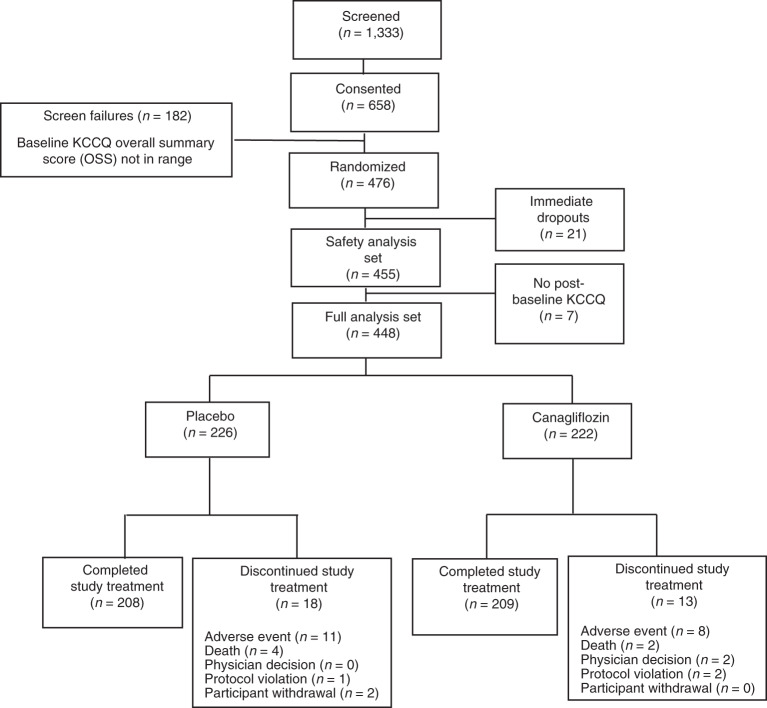
Study CONSORT diagram showing the process of subject participation. KCCQ, Kansas City Cardiomyopathy Questionnaire.

Participants were recruited between 26 March 2020 and 12 February 2021. Among 476 participants randomized, 21 immediately withdrew without ever taking study medications, and seven did not provide a follow-up KCCQ, resulting in 448 participants being included in the primary intention-to-treat analyses, of whom 222 were randomized to canagliflozin and 226 to placebo (Fig. 1). Baseline characteristics of these 448 participants are detailed in Table 1 and were well balanced between treatment groups. Overall, mean age was 63.4 ± 13.3 years (range, 20–94); 84% of participants were White; 45% of participants were women; 28% of participants had type 2 diabetes; and 60% of participants had HFpEF. At 12 weeks, KCCQ scores were available in 414 participants (92.4%), 206 randomized to placebo and 208 to canagliflozin.

**Table 1:** Baseline characteristics

	Placebo	Canagliflozin	Total
Sample size	226	222	448
Age (years)			
Mean (s.d.)	64.0 (13.45)	62.9 (13.19)	63.4 (13.32)
Median	66.0	65.0	66.0
Range	(22; 94)	(20; 89)	(20; 94)
18–25	2 (0.9%)	4 (1.8%)	6 (1.3%)
26–50	38 (16.8%)	35 (15.8%)	73 (16.3%)
51–64	59 (26.1%)	68 (30.6%)	127 (28.3%)
≥65	127 (56.2%)	115 (51.8%)	242 (54.0%)
Gender			
Female	97 (42.9%)	104 (46.8%)	201 (44.9%)
Race			
White	194 (85.8%)	182 (82.0%)	376 (83.9%)
Black or African American	30 (13.3%)	35 (15.8%)	65 (14.5%)
Asian	1 (0.4%)	1 (0.5%)	2 (0.4%)
Other	1 (0.4%)	4 (1.8%)	5 (1.1%)
Diabetes			
Type 2 diabetes mellitus	59 (26.1%)	66 (29.7%)	125 (27.9%)
Non-type 2 diabetes mellitus	167 (73.9%)	156 (70.3%)	323 (72.1%)
Randomization stratification			
HFpEF	135 (59.7%)	132 (59.5%)	267 (59.6%)
HFrEF	91 (40.3%)	90 (40.5%)	181 (40.4%)
KCCQ scores			
Total symptom score	58.0 ± 21.1	57.4 ± 21.3	57.7 ± 21.2
Overall summary score	52.7 ± 18.3	51.6 ± 18.8	52.1 ± 18.5
Clinical summary score	56.3 ± 19.5	54.6 ± 19.7	55.5 ± 19.6
Physical limitation score	54.4 ± 21.5	51.9 ± 21.2	53.1 ± 21.4
Social limitation score	50.9 ± 22.4	50.9 ± 23.8	50.9 ± 23.1
Quality of life score	47.4 ± 21.8	45.8 ± 21.2	46.6 ± 21.5
Step counts	4,041.4 ± 2,774.9	4,583.8 ± 3150.5	4,310.1 ± 2,975.8

### Study execution

Among the 448 randomized participants included in the intention-to-treat analyses, all received their study medication and Fitbit (Supplementary Table 1). The diagnosis of HF was confirmed by claims data in all participants. The compliance with completing an eDiary of medication use was 95%, and 91% reported taking more than 80% of their study medications. Participants’ Fitbit data transmissions indicated that 94% wore their Fitbit 70% or more of the time. The KCCQ data were very complete, being completed more than 97% of the time at each scheduled assessment.

### Outcomes

**Fig. 2:**
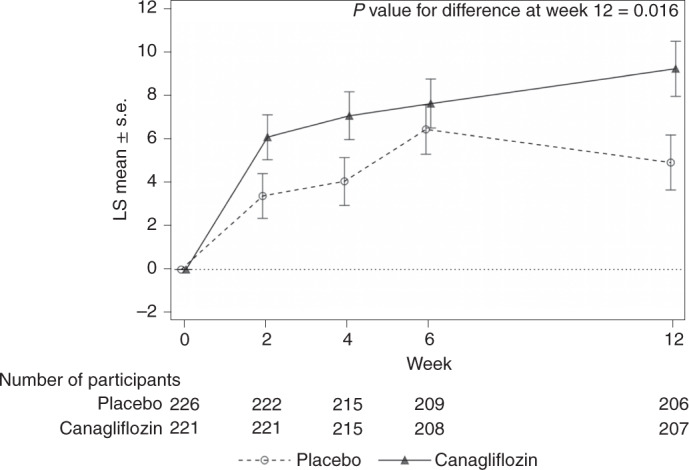
KCCQ TSS over time. Mean changes in KCCQ TSSs (error bars represent standard errors) at 2, 4, 6 and 12 weeks after randomization.

**Extended Data Fig. 1:**
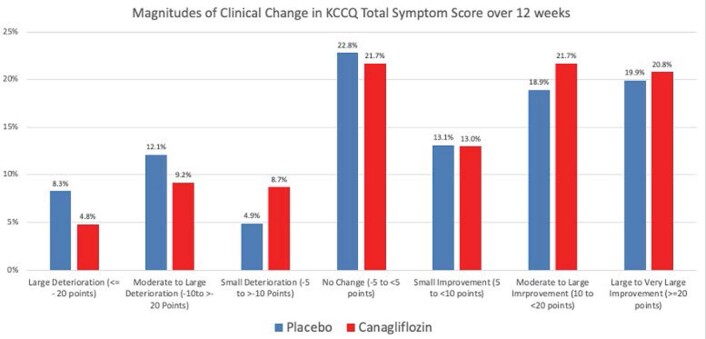
Distributions of 3-month Changes in KCCQ Total Symptom Scores. The proportions of patients experiencing the indicated magnitudes of clinical change are shown. KCCQ=Kansas City Cardiomyopathy Questionnaire.

The baseline KCCQ TSS was 58 ± 21 in participants randomized to placebo and 57.4 ± 21 in participants randomized to canagliflozin. At 12 weeks, both groups had improvements in their scores, to 63.2 ± 22 and 67.1 ± 22, with changes of 5.2 ± 20 and 8.9 ± 20 in the placebo and canagliflozin groups, respectively. Figure 2 and Table 2 show the changes in scores over time, which begin to separate at 2 weeks. The mean difference in the changes in scores at 12 weeks was 4.3 points (95% confidence interval (CI), 0.8–7.8; *P* = 0.016) in favor of canagliflozin. Extended Data Fig. 1 shows the proportions of patients with different magnitudes of clinical change. A larger number of patients deteriorated by a moderate or greater amount on placebo, whereas a larger number of participants had moderate to large improvements with canagliflozin. Improvements in mean scores were also observed for most other KCCQ domains but not for changes in step counts, which did not change over 12 weeks in either group (mean difference favoring canagliflozin of 29.8 steps (95% CI, −284 to 344)).

**Table 2:** Primary outcome—KCCQ TSS

	Observed values				Change from baseline				
	Placebo		Canagliflozin		Placebo	Canagliflozin	Difference of change		
	*n*	Mean (s.d.)	*n*	Mean (s.d.)	LS mean (s.e.)	LS mean (s.e.)	LS mean (s.e.)	95% CI	*P* value
Baseline	226	58.0 (21.12)	221	57.4 (21.32)					
Week 2	222	61.5 (21.21)	222	63.5 (20.90)	3.4 (1.03)	6.1 (1.03)	2.7 (1.44)	(−0.1, 5.5)	
Week 4	215	62.1 (21.50)	216	64.5 (21.01)	4.1 (1.10)	7.1 (1.10)	3.0 (1.54)	(−0.0, 6.1)	
Week 6	209	64.8 (21.44)	209	65.0 (21.62)	6.4 (1.13)	7.6 (1.13)	1.2 (1.59)	(−1.9, 4.3)	
Week 12	206	63.2 (22.32)	208	67.1 (22.19)	4.9 (1.27)	9.2 (1.27)	4.3 (1.78)	(0.8, 7.8)	0.016

The LS means, standard errors, 95% CIs and *P* values are based on a repeated-measures, mixed-effects ANCOVA model with treatment, stratification factor (HFrEF or HFpEF), time, time by treatment and baseline KCCQ TSS values as covariates, with an unstructured covariance structure.

**Fig. 3:**
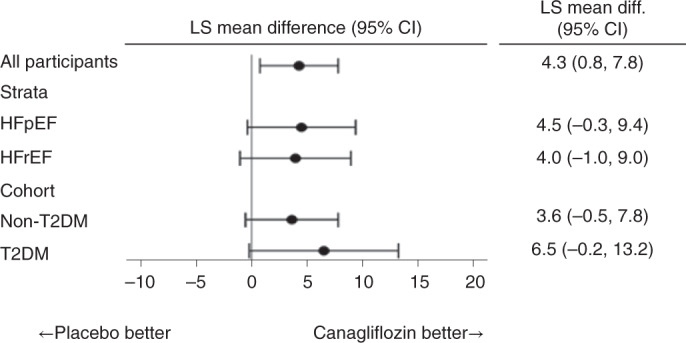
Effects of treatment by HF type and diabetes statu*s*. Estimates of the mean difference in KCCQ TSSs by subgroups are stratified by EF and diabetes status. In total, 208 participants were treated with canagliflozin and 206 with placebo. T2DM, type 2 diabetes mellitus.

The effects of canagliflozin on the change in the KCCQ TSS at 12 weeks were consistent in patients with HFrEF (4.0; 95% CI, −1.0 to 9.0) and HFpEF (4.5; 95% CI, −0.3 to 9.4) (*P* value for interaction = 0.35; Fig. 3). Similar benefits were also observed in participants with type 2 diabetes (6.5; 95% CI, −0.2 to 13.2) and participants without type 2 diabetes (3.6; 95% CI, −0.5 to 7.8) (*P* value for interaction = 0.90).

### Safety

Serious adverse events and death are summarized in Supplementary Tables 2 and 3. Based on the claims data, 9.9% (45/455) of participants had a serious adverse event (emergency room visit or hospitalization) through week 12 (27 (12.1%) in canagliflozin and 18 (7.8%) in placebo). Four participants randomized to the canagliflozin group and two randomized to the placebo group were hospitalized for HF. Six participants (two in the canagliflozin group and four in the placebo group) died during the 12-week treatment period. No diabetic ketoacidosis or lower limb amputations occurred (Table 3).

**Table 3:** Secondary outcomes

	Observed values					Change from baseline		
	Placebo		Canagliflozin		Placebo	Canagliflozin	Difference of change	
	*n*	Mean (s.d.)	*n*	Mean (s.d.)	LS mean (s.e.)	LS mean (s.e.)	LS mean (s.e.)	95% CI
KCCQ domain scores								
Overall summary score								
Week 12	206	59.1 (21.39)	208	61.7 (22.22)	6.2 (1.18)	9.5 (1.18)	3.3 (1.66)	(0.0, 6.6)
Clinical summary score								
Week 12	206	61.3 (20.75)	208	63.7 (21.81)	4.7 (1.16)	8.5 (1.17)	3.7 (1.64)	(0.5, 7.0)
Physical limitation score								
Week 12	206	59.5 (23.00)	204	60.5 (23.58)	4.8 (1.26)	7.8 (1.27)	3.0 (1.78)	(−0.5, 6.5)
Quality of life score								
Week 12	206	56.3 (24.88)	208	58.9 (23.51)	9.1 (1.40)	12.4 (1.41)	3.3 (1.98)	(−0.5, 7.2)
Social limitation score								
Week 12	202	57.2 (26.15)	204	60.3 (27.49)	6.2 (1.48)	8.8 (1.48)	2.6 (2.08)	(−1.4, 6.7)
Total daily step counts								
Week 12	208	4,013.6 (2,624.28)	205	4,480.5 (3,033.79)	−74.9 (112.85)	−45.1 (113.78)	29.8 (159.84)	(−284.4, 344.1)

The LS means, standard errors, 95% CIs and *P* values are based on a repeated-measures, mixed-effects ANCOVA model with treatment, stratification factor (HFrEF or HFpEF), time, time by treatment and baseline KCCQ TSS values as covariates, with an unstructured covariance structure.

## Discussion

The results of this trial demonstrate the feasibility of a decentralized, virtual study design that was successfully launched and executed during the COVID-19 pandemic and which adds considerable new insights into the health status effects of SGLT2is in HF. Improving symptom burden is a critical goal for HF management. CHIEF-HF, a randomized, placebo-controlled trial accomplished without any in-person visits, demonstrated that canagliflozin resulted in a rapid and clinically meaningful improvement in the symptoms of patients with HF, as quantified by the KCCQ. This benefit was consistent across the range of EF and in patients with and without type 2 diabetes. Although canagliflozin does not have an HF indication, this study adds important supporting evidence about the beneficial effects of the class of SGLT2 inhibitors on improving HF symptoms, with novel data indicating that these benefits can occur as early as 2 weeks after initiation of therapy.

The fact that CHIEF-HF launched 2 weeks before a national shutdown due to the COVID-19 pandemic highlights the potential advantages of a decentralized, virtual clinical trial^19^. Underscoring the challenge of research in the COVID-19 era, the US Food & Drug Administration^20^ and a Heart Failure Collaboratory Statement^21^ have highlighted the need to prioritize safety, even if sacrificing protocol adherence. Because the primary outcome was the KCCQ, and given the well-established safety of the SGLT2i class, the study drug was distributed remotely, and the outcomes were collected virtually on participants’ phones. In fact, the ability to use a smartphone app to enroll and collect KCCQ and adherence data with good data quality supported recruitment that was five times faster than the average enrollment rate in HF trials^22^. Of course, the use of mobile technology can introduce potential selection biases, from requiring patients to own (potential socio-economic biases) and be able to use (potential age and cognitive biases) a smart device, although access to such devices are growing over time. Future studies examining interventions to improve the health status of patients can consider such an approach, if there are no anticipated safety concerns that might require sequential monitoring with blood work or imaging tests. The CHIEF-HF trial also modeled how an SGLT2i could be safely initiated without a requirement for in-person visits and, if desired, patients’ health status monitored remotely to assess its effect.

The findings of this study augment a growing body of literature on the benefits of SGLT2is in patients with HF. Several large clinical trials have documented reduced cardiovascular mortality and hospitalizations in patients with HFrEF with this class, regardless of type 2 diabetes^8,10^, and a recent trial demonstrated a clinical benefit of empafligozin in patients with HFpEF^14,16^. Several trials with dapafliglozin demonstrated a similar magnitude of improvement in patients’ health status in HFrEF and HFpEF, as found in CHIEF-HF^9,13^. CHIEF-HF findings agree closely with these previous observations in terms of the magnitude of benefit in HFrEF, but it is, to our knowledge, the first to demonstrate an early benefit on participants’ health status in a broad spectrum of patients with HF, including those with HFpEF. Collectively, these data indicate that the use of SGLT2i not only improves prognosis but also meaningfully improves symptoms, function and quality of life.

There have been concerns that the COVID-19 pandemic might alter patient-reported outcomes independently of any treatment effect^23^. In fact, the largest effect of treatment was observed in the symptom scale of the KCCQ, with slightly lesser effects on physical and social limitations. Whether these domains were affected by other factors, such as home isolation, is unknown, and the minimal changes in step counts might have been affected by behavior changes in the setting of COVID-19. Future studies will need to define the effect of SGLT2is on measures of physical activity. It is also noteworthy that the symptoms improved in patients treated with placebo. Although this might be considered a placebo effect, it is also possible that participants’ adherence to other HF medications improved during the trial, given that they had weekly reminders for reporting their medication use.

These findings should be interpreted in the context of several potential limitations. First, the trial design originally planned for 1,900 patients to have 95% power to detect a treatment benefit of 3 points, which was arguably overpowered. Thus, although study enrollment stopped early, a statistically significant benefit of treatment was still detected. Second, although the enrollment of women and minorities is higher than in most previous SGLT2i trials, including 15% African American participants, additional studies in these populations are warranted. Third, the study was not designed or powered to examine clinical events, which have been studied in other trials. In addition, given its unique design, there were no case report forms in this study, and less detailed clinical and comorbidity data are available. Future trials using this approach might want to design a more detailed case report form to be completed at screening by sites, although this would increase the burden and costs of the trial. Finally, the nature of the study design precluded capturing biomarker or imaging data that could potentially illuminate the potential mechanisms of benefit, and changes in concomitant medications were not captured, although the short duration of the trial likely minimized the importance of this latter concern.

In conclusion, the CHIEF-HF study executed a novel, decentralized, double-blind, randomized controlled trial design focusing on patient-centered outcomes. It also demonstrates the benefits of canagliflozin in significantly improving patients’ symptom burden, regardless of EF or type 2 diabetes status, further underscoring the benefits of SGLT2is in addressing a key treatment goal for patients with HF. Such novel approaches to generating important evidence offer the potential for future clinical trials to lower the cost and increase the speed of acquiring new evidence to improve clinical practice.

## Methods

### Study population

The design of the CHIEF-HF study was previously described, and all patients provided informed consent^24^. A central institutional review board (Advarra) approved the study. The complete inclusion and exclusion criteria are provided in the study protocol, along with the statistical analysis plan, as supplementary material. In brief, different recruitment sites used different strategies for identifying patients to participate, including email, patient portals through the health system’s electronic medical record, phone calls and contacting providers before a scheduled visit. Potential participants expressed interest in enrolling and confirmed that they were in sole possession of an Apple iPhone 6 (or later) or a Samsung Galaxy phone and were willing to wear a Fitbit device (Fitbit Versa 2). The site principal investigators then confirmed study inclusion criteria (the screening process), including a diagnosis of HF (HFrEF with an EF < 40% and a primary or 2 HF diagnosis in any position within 18 months; HFpEF with an EF ≥ 40% and similar diagnosis codes as HFrEF and treatment with a loop diuretic or mineralocorticoid receptor antagonist). They also confirmed that no exclusion criteria were present, including no use of an SGLT2i within 3 months, no history of diabetic ketoacidosis or type 1 diabetes and an estimated glomerular filtration rate <30 ml min^−1^. Eligible patients then provided electronic informed consent via the app, after reviewing it over the phone with the site principal investigator. Once consented, they completed the KCCQ on the study app. Those with an overall summary score of 80 or lower were then enrolled and randomized. Of 658 participants who consented, 182 (27.6%) were excluded based on their KCCQ scores.

### Outcomes

The primary outcome was change in the KCCQ TSS—a domain of the KCCQ scale that quantifies patient symptom frequency and severity over the past 2 weeks. The KCCQ scale has extensive data supporting its validity, reliability, sensitivity to clinical change and association with other clinical events, including HF hospitalization and death^25–30^. The KCCQ was collected at screening and at 2, 4, 6 and 12 weeks after randomization. Scores are transformed from 0 points (the worst) to 100 points. Although lower thresholds for minimal clinically important differences in the KCCQ have been reported^31^, changes of 5, 10 and 20 points are generally considered to represent small (but clinically important), moderate to large and large to very large clinical changes, respectively.^32–34^ A shift of one response category in a symptom-informative question increases the TSS by 2.08–4.2 points, depending on the item, meaning that a 5-point change requires a net improvement of at least two responses^34^.

Secondary endpoints included change from baseline in the 2-week average of daily step counts acquired from the Fitbit and changes in other domain scores of the KCCQ scores at 12 weeks. Adverse event reporting was collected from patients by self-report through the coordinating center, and serious adverse events were collected through claims data. Vital status was obtained at the end of the study in those lost to follow-up.

### Statistical analyses

The original protocol was approved on 7 November 2019 and amended on 7 February 2020 (to remove the original plan to return study results to patients) and again on 2 June 2020 (to remove an initial exclusion of those with a KCCQ overall summary score <40 and to add mineralocorticoid receptor antagonists as confirmation of an HFpEF diagnosis). The Statistical Analysis Plan was developed on 10 April 2020 and finalized before database lock on 21 July 2021. The Protocols and Statistical Analysis Plan are provided in Supplementary Note 2.

Because of the novel study design, it was anticipated that some patients would sign up for the study but not ultimately participate. Thus, the intention-to-treat analysis was based on all randomized patients who took at least one dose of the study drug and had at least one post-randomization KCCQ (full analysis set). A valid post-randomization KCCQ TSS, which was the primary endpoint of the study, was required for the intention-to-treat analysis to test changes in KCCQ TSS. The safety analysis set included all randomized patients who took at least one dose of the study drug (safety analysis set). Baseline data are reported as means ± s.d. and categorical variables as frequencies. Outcome data are reported as means ± s.d. with 95% CIs. The mechanics of study excecution are described as the frequency of complete data collection and self-reported medication adherence.

The primary outcome—change in the KCCQ TSS—was assessed with a mixed-effects model for repeated measures (MMRM) that included treatment (canagliflozin or placebo), stratification (HFrEF versus HFpEF), time, time-by-study intervention interaction and baseline KCCQ TSS score, using an unstructured covariance matrix. Least squares (LS) mean differences and 95% CIs were estimated at week 12 for placebo versus canagliflozin. This was repeated for key pre-specified subgroups: HFrEF versus HFpEF and participants with and without type 2 diabetes. To support clinical interpretation of the mean differences in scores, the distribution of patients with different clinical magnitudes of change were calculated. Although imputation approaches were planned for, the very high completion of the KCCQ did not require their use. The key secondary outcome of daily step count was to be analyzed hierarchically after the primary outcome using the same MMRM method, as were the other KCCQ domains. No *P* values are reported for the secondary analyses because the smaller-than-planned sample size left no room for additional analyses; this also aligns with current recommendations to minimize the reporting of *P* values^35^. Analyses were conducted by Janssen and independently validated at Saint Luke’s Mid America Heart Institute. SAS version 9.4 software was used, and two-sided *P* values less than 0.05 were considered statistically significant.

The trial was sponsored by Janssen Scientific Affairs. The sponsor participated in the design and conduct of the study; the collection, management, analysis and interpretation of the data; the review of the manuscript; and the decision to submit the manuscript for publication. The sponsor did not have the right to veto publication and did not have control regarding the journal to which the paper was submitted.

### Reporting Summary

Further information on research design is available in the Nature Research Reporting Summary linked to this article.

## Online content

Any methods, additional references, Nature Research reporting summaries, source data, extended data, supplementary information, acknowledgements, peer review information; details of author contributions and competing interests; and statements of data and code availability are available at 10.1038/s41591-022-01703-8.

## Supplementary information

Supplementary Information Supplementary Information File: Supplementary Tables 1–3, Supplementary Note 1 (participating sites and Extended Data Fig. 1) and Supplementary Note 2 (Protocols and Statistical Analysis Plans)

Reporting Summary

## Data Availability

Requests for access to the study data can be made through Yale Open Data Access (http://yoda.yale.edu) 18 months after completion of the trial, which is 1 March 2022 (last contact for extended follow-up).
