# Supplementary material for: The SGLT2 inhibitor canagliflozin in heart failure: the CHIEF-HF remote, patient-centered randomized trial
Source: Nat Med. 2022 Feb 28;28(4):809–13. doi: 10.1038/s41591-022-01703-8 (PMC9018422; doi:10.1038/s41591-022-01703-8)
Supplement: Supplementary file 1 — Supplementary Information File: Supplementary Tables 1–3, Supplementary Note 1 (participating sites and Extended Data Fig. 1) and Supplementary Note 2 (Protocols and Statistical Analysis Plans) [file 41591_2022_1703_MOESM1_ESM.pdf]

nature portfolio

https://doi.org/10.1038/s41591-022-01703-8

Supplementary information

The SGLT2 inhibitor canagliflozin in heart failure: the CHIEF-HF remote, patient-centered randomized trial

In the format provided by the authors and unedited

Supplemental Information File

Supplemental Table 1:

Study Compliance Metrics – Full Analysis Set

**Participant Eligibility**	**Collected/Confirmed**	**Expected***	**Rate**
Claims validation of HF at Baseline	448	448	100%
**Study Drug Delivery and Adherence**			
Direct to Patient Drug Delivered**	448	448	100%
Self-reported medication compliance $\geq$80%	385	426	91%
**Data Collection**			
eDiary Compliance ≥80%	426	448	95%
KCCQ at 2 weeks	444	448	99%
KCCQ at 4 weeks	431	438	98%
KCCQ at 6 weeks	418	429	97%
KCCQ at 12 weeks	414	422	98%
Fitbit Data compliance ≥80%	422	448	94%

*Participants were included at all time points until withdrawal, death or end of study.
**5 shipments were delayed by the vendor

Supplemental Table 2: Overall summary Adverse Events

Overall Summary of Post-randomization Adverse Events Through Week 12 As Reported in Claims Database			
	Placebo	Canagliflozin	Total
Number of participants	231	224	455
Serious adverse events	18 (7.8%)	27 (12.1%)	45 (9.9%)
Any Adverse Events of Interests	16 (6.9%)	24 (10.7%)	40 (8.8%)
Death	4 (1.7%)	2 (0.9%)	6 (1.3%)
By Cohort			
T2DM	3 (1.3%)	1 (0.4%)	4 (0.9%)
No T2DM	1 (0.4%)	1 (0.4%)	2 (0.4%)
By Strata			
HFrEF	3 (1.3%)	2 (0.9%)	5 (1.1%)
HFpEF	1 (0.4%)	0	1 (0.2%)

Note: Serious adverse event numbers are from hospitalization/emergency room visit data and Day (87+30) is the cutoff day. Death numbers are from Disposition data including records up to 30 days after end of treatment.

Supplemental Table 3: Deaths

Treatment	T2DM	Stratum	Cause (Decoded Term)	Relationship to Study Agent
Placebo	N	HFpEF	Cardiac Arrest	Not Related
			Gastrointestinal Hemorrhage	Not Related
	N	HFpEF	Cardiac Failure Congestive	Not Related
	N	HFrEF	Sudden Cardiac Death	Not Related
	Y	HFpEF	Pneumonitis	Not Related
			Implantable Defibrillator Replacement	Not Related
			Dyspnea	Not Related
Canagliflozin	Y	HFpEF	Covid-19	Not Related
	N	HFpEF	Covid-19	Not Related
			Cerebrovascular Accident	Not Related

Supplemental Note 1: Participating Health Systems

1. Clearwater Cardiovascular Consultants, Clearwater, FL (PI: Douglas Spriggs)
2. Emory University, Atlanta, GA (PI: Arash Harzand)
3. Henry Ford Health System, Detroit, MI (PI: David Lanfear)
4. MedStar Health Research Institute, Hyattsville, MD (PI: Jean Park)
5. Mercy Clinic Cardiology, Fort Smith, AR (PI: Surbhi Chamaria)
6. Mercy Health Research, Chesterfield, MO (PI: Vernon Young)
7. Northwestern Medical Center – DuPage Hospital, Winfield, IL (PI: Anjan Tibrewala)
8. OSF Cardiovascular Institute, Peoria, IL (PI: Christopher Sparrow)
9. Parkview Research Center, Fort Wayne, IN (PI: Mark O'Shaughnessy)
10. Robert Wood Johnson Medical School, New Brunswick, NJ (PI: Abel Moreyra)
11. Saint Luke's Boise Medical Center, Boise ID (PI: David Hinchman)
12. Saint Luke's Mid America Heart Institute, Kansas City, MO (PI: Michael Nassif)
13. Texas Heart Institute, Houston, TX (PI: Emerson Perin)
14. Thomas Spann Clinic, Corpus Christi, TX (PI: Brandi Addison)
15. University of Colorado Hospital, Denver CO (PI: Laura Peters)
16. University Hospitals, Cleveland OH (PI: Varun Sundaram)
17. University of Kansas Medical Center, Kansas City, KS (PI: Bhanu Gupta)
18. University of Michigan, Ann Arbor, MI (PI: Brahmajee Nallamothu)

Supplementary Note 2: Protocols and Statistical Analysis Plans

Original Protocol……………………………………………………………………………………………………...2
Final Protocol……………………………………………………………………………………………………….68
Summary of Changes to Protocol…………………………………………………………………………...143
Original SAP……………………………………………………………………………………………………...145
Final SAP……...188
Summary of Changes to SAP………………………………………………………………………………….235

Original Protocol

Janssen Scientific Affairs*

Clinical Protocol

Protocol Title
Canagliflozin: Impact on Health Status, Quality of Life, and Functional Status in Heart Failure

CHIEF-HF

Protocol 28431754HFA3002; Phase 3B

JNJ-28431754 (canagliflozin) (INVOKANA)

*Janssen Research & Development is a global organization that operates through different legal entities in various countries. Therefore, the legal entity acting as the sponsor for Janssen Research & Development studies may vary, such as, but not limited to Janssen Biotech, Inc.; Janssen Products, LP; Janssen Biologics, BV; Janssen-Cilag International NV; Janssen, Inc; Janssen Pharmaceutica NV; Janssen Sciences Ireland UC; or Janssen Research & Development, LLC. The term “sponsor” is used throughout the protocol to represent these various legal entities; the sponsor is identified on the Contact Information page that accompanies the protocol.

US sites of this study will be conducted under US Food & Drug Administration IND regulations (21 CFR Part 312).

EudraCT NUMBER: 2018-000538-36

Status: Approved
Date: 7 November 2019
Prepared by: Janssen Scientific Affairs
EDMS number: EDMS-ERI-154820237, 2.0

GCP Compliance: This study will be conducted in compliance with Good Clinical Practice, and applicable regulatory requirements.

Confidentiality Statement
The information in this document contains trade secrets and commercial information that are privileged or confidential and may not be disclosed unless such disclosure is required by applicable law or regulations. In any event, persons to whom the information is disclosed must be informed that the information is privileged or confidential and may not be further disclosed by them. These restrictions on disclosure will apply equally to all future information supplied to you that is indicated as privileged or confidential.

TABLE OF CONTENTS

TABLE OF CONTENTS **2**

LIST OF IN-TEXT TABLES AND FIGURES **4**

1. PROTOCOL SUMMARY **5**
1.1. Synopsis 5
1.2. Schema 10
1.3. Schedule of Activities (SoA) 11

2. INTRODUCTION **13**
2.1. Background and Study Rationale 17
2.2. Benefit/Risk Assessment 18

3. OBJECTIVES AND ENDPOINTS **19**

4. STUDY DESIGN **21**
4.1. Overall Design 21
4.2. Scientific Rationale for Study Design 23
4.2.1. Study-Specific Ethical Design Considerations 25
4.3. Justification for Dose 25
4.4. End of Study Definition 25

5. STUDY POPULATION **25**
5.1. Inclusion Criteria 26
5.2. Exclusion Criteria 26
5.3. Prohibitions and Restrictions 27
5.4. Screen Failures 27

6. STUDY INTERVENTION **28**
6.1. Study Interventions Administered 28
6.2. Preparation/Handling/Storage/Accountability 30
6.3. Measures to Minimize Bias: Randomization and Blinding 30
6.4. Study Intervention Compliance 31
6.5. Prohibited Medications 31

7. DISCONTINUATION OF STUDY INTERVENTION AND PARTICIPANT DISCONTINUATION/WITHDRAWAL **31**
7.1. Discontinuation of Study Intervention 31
7.1.1. Temporary Discontinuation of Study Intervention 32
7.2. Participant Discontinuation/Withdrawal From the Study 32

8. STUDY ASSESSMENTS AND PROCEDURES **33**
8.1. Effectiveness Assessments 33
8.1.1. KCCQ Scores 33
8.2. Exploratory Assessments 34
8.2.1. Patient Global Impression of Change (PGIC) Scores 34
8.2.2. Patient Global Impression of Severity (PGI-S) Scores 34
8.2.3. Participant Satisfaction Survey 34
8.2.4. Healthcare Resource Utilization and Health Economics 35
8.3. Adverse Events and Serious Adverse Events and Special Situations 35
8.3.1. Time Period and Frequency for Collecting Adverse Event and Serious Adverse Event Information 35
8.3.2. Regulatory Reporting Requirements for Serious Adverse Events 36
8.3.3. Pregnancy 36
8.3.4. Adverse Events of Interest 36

9. STATISTICAL CONSIDERATIONS 36
9.1. Statistical Hypotheses 37
9.2. Sample Size Determination 37
9.3. Populations for Analyses 37
9.4. Statistical Analyses 37
9.4.1. Handling of Missing Data 37
9.4.2. Effectiveness Analyses 38
9.4.2.1. Primary and Secondary Effectiveness Analyses 38
9.4.2.2. Exploratory Analyses 38
9.4.3. Adverse Events Analysis 39
9.4.4. Healthcare Resource Utilization and Health Economics Analyses 39
9.5. Interim Analysis 39

10. SUPPORTING DOCUMENTATION AND OPERATIONAL CONSIDERATIONS 40
10.1 Appendix 1: Abbreviations and Trademarks 40
10.2 Appendix 2: Regulatory, Ethical, and Study Oversight Considerations 42
10.3 Appendix 3: Kansas City Cardiomyopathy Questionnaire (KCCQ) 50
10.4 Appendix 4: Patient Global Impression of Change (PGIC) 54
10.5 Appendix 5: Patient Global Impression of Severity (PGI-S) 55
10.6 Appendix 6: Participant Satisfaction Survey 56
10.7 Appendix 7: Adverse Events: Definitions and Procedures for Recording, Evaluating, Follow-up, and Reporting 57
10.8 Appendix 8: Protocol Amendment History 62

11. REFERENCES 63

INVESTIGATOR AGREEMENT 65

LIST OF IN-TEXT TABLES AND FIGURES

TABLES

Table 1: Effects of Sodium-glucose Cotransports-2 Inhibitors (SGLT-2is) on Heart Failure Outcomes 18
Table 2: Analysis Populations 37

FIGURES

Figure 1: Schematic Overview of the Study 10

1. PROTOCOL SUMMARY

1.1. Synopsis

Canagliflozin: Impact on Health Status, Quality of Life, and Functional Status in Heart Failure

Canagliflozin (JNJ-28431754) is an inhibitor of sodium-glucose co-transporter 2 (SGLT2) that has been developed as an oral antihyperglycemic agent (AHA) for the treatment of patients with type 2 diabetes mellitus (T2DM). The full chemical name for canagliflozin is (1S)-1,5-anhydro-1-[3-[[5-(4-fluorophenyl)-2-thienyl]methyl]-4-methylphenyl]-D-glucitol hemihydrate.

Canagliflozin therapy has been shown to improve clinical outcomes including hospitalizations for heart failure (HHF) in patients with T2DM and HF based on results from the CANVAS program (CANVAS [DIA3008] /CANVAS-Renal [DIA4003]) and CREDENCE [DNE3001] study. An improvement in patient's symptoms of HF would be an important advance in the clinical management of HF. This randomized study is designed to assess whether canagliflozin therapy improves HF symptoms as assessed by the Total Symptom Score (TSS) of the Kansas City Cardiomyopathy Questionnaire (KCCQ) patient-reported outcome (PRO) scale in participants with HF and with or without T2DM in a real-world setting.

OBJECTIVES AND ENDPOINTS

Primary Objective

The primary objective is:

- to determine the superiority of the effectiveness of canagliflozin 100 mg daily versus placebo in participants with symptomatic HF in improving the overall KCCQ TSS.

Secondary Objectives

The first key secondary objective is:

- to determine the superiority of the effectiveness of canagliflozin 100 mg daily versus placebo in improving the total daily step count.

The second key secondary objective is:

- to determine the superiority of the effectiveness of canagliflozin 100 mg daily versus placebo in improving the KCCQ individual domain scores (physical limitation, quality of life, clinical summary, and overall).

Exploratory Objectives

The exploratory objectives are to assess the effectiveness of canagliflozin 100 mg daily in participants with symptomatic HF by:

- responses on the Patient Global Impression of Change (PGIC) and the Patient Global Impression of Severity (PGI-S)
- healthcare resource utilization (HRU) and health economics data
- associations (correlations and/or categorical association measures) between digital markers (step count and stairs climbed), and clinical events such as need for outpatient intravenous therapies, emergency department (ED) visits, and hospitalizations
- daily stairs climbed
- time to first hospitalization/readmission for heart failure

- time to first hospitalization/readmission for other events
- time to death
- impact of elective use of the interventional therapy class (sodium-glucose co-transporter 2 inhibitor [SGLT2i]) by staged data disclosure
- the participant's satisfaction with their experience with the virtual design at the end of the study.

Endpoints

The primary effectiveness endpoint is the change in KCCQ TSS from baseline to Month 3.

The key secondary effectiveness endpoints are 1) change in total daily step count from baseline to Month 3, and 2) changes in KCCQ individual domain scores (physical limitation, quality of life, clinical summary, and overall) from baseline to Month 3.

The exploratory endpoints are the following:

- mean PGIC scores over time
- changes in PGI-S scores from baseline to Month 3
- change in number of daily stairs climbed
- change in HRU and health economics data
- number of outpatient intravenous therapies, ED visits, and hospitalizations
- time to first hospitalization/readmission for heart failure
- time to first hospitalization/readmission for other events
- time to death
- change in elective use of the interventional therapy class (ie, SGLT2i) by staged data disclosure.

Adverse Event Data Collection

Due to the virtual method of data collection for this study, there will be 2 types of adverse event data collection. One will be done by participants instructed to self-report any adverse events from the study intervention to a call center, and the other by an analysis of medical claims data from the participant's provider to their insurance system. Self-reported events will be captured from the date of the first dose of study intervention through the end of treatment (Month 3) plus 30 days. Medical claims data will be reviewed and evaluated in aggregate at the end of the 3-month, double-blind treatment period when the unblinded data are available.

Hypotheses

The primary hypothesis is that canagliflozin is superior to placebo as assessed by improvement in the TSS on the KCCQ scale during the 3-month, double-blind treatment period.

OVERALL DESIGN

This is a randomized, double-blind, placebo-controlled, decentralized, virtual, interventional, superiority study conducted in the United States (US) in participants with symptomatic HF (stratified by heart failure with reduced ejection fraction [HFrEF] and heart failure with preserved ejection fraction [HFpEF]) to examine the improvement in the KCCQ TSS after 3 months of treatment with canagliflozin 100 mg or placebo. The study will continue for an additional 6 months without any study intervention to collect participant follow-up in a real-world setting.

The study will enroll participants from large, integrated, health networks and large physician practices in the US with the participants conducting the majority of their study-related activities through an app on a smartphone and actigraphy data from a Fitbit device. There will be no in-person clinic visits required by the study.

The study consists of a screening period and a 3-month, double-blind treatment period. Approximately 1,900 participants will be randomized into the study in a 1:1 ratio to canagliflozin 100 mg daily or placebo groups (950 in each study intervention group). After Month 3 (the end of the double-blind treatment period), the study will continue for an additional 6 months when study intervention will no longer be provided. Data collection from claims, smartphone, Fitbit device, and selected PROs will continue until Month 9 for the purpose of secondary and further exploratory endpoints analyses. In addition, the KCCQ, PGIC, and PGI-S data will be collected at 6 and 9 months. An optional participant satisfaction survey about the virtual nature of the study will be provided to the participant at end of the study.

Participants will be of any gender, 18 years of age or older, and have clinically stable, symptomatic HF with or without T2DM with a KCCQ score of >40 and <80 prior to randomization.

Study assessments include the KCCQ, Fitbit device data (step count and stairs climbed), PGIC, PGI-S, participant satisfaction survey, medical and pharmacy claims data (including up to 36 months history for eligibility confirmation), HRU and health economics (including hospitalization/readmission due to HF or other reason, ED visits, the duration of stay at each hospitalization/readmission), review of claims for prohibited medications and new diagnoses that may require discontinuation, and adverse events.

NUMBER OF PARTICIPANTS

Approximately 1,900 participants will be randomized in the study in a 1:1 ratio to canagliflozin 100 mg daily or placebo. Randomization of participants into the study will be stratified by the type of ejection fraction (ie, HFrEF or HFpEF) at study entry as recorded in the participant’s electronic health record (EHR).

INTERVENTION GROUPS AND DURATION

The total duration of study participation for each participant is approximately 9 months. Participants will be randomly assigned to receive canagliflozin 100 mg daily immediate-release over-encapsulated tablets (capsules) taken orally or placebo capsules for the duration of the 3-month, double-blind treatment period, followed by a 6-month, no treatment period during which claims data will continue to be collected.

Description of Interventions

The study intervention, JNJ-28431754 (canagliflozin), will be provided as immediate-release, over-encapsulated tablets (as a capsule) or a placebo capsule taken orally once daily for the duration of the 3-month, double-blind treatment period.

Return of Results (RoR)

As each randomized participant completes the 3-month, double-blind treatment period of the study, the RoR to participants will begin. Participants will be provided with their treatment allocation, whether they had been randomized to canagliflozin or placebo. After the last randomized participant completes the 3-month, double-blind treatment period, and after the data are analyzed, participants will be provided aggregate interim study results (ie, KCCQ and activity data). At the end of the study and after all data has been analyzed, the participants will be provided with a final participant study report.

EFFECTIVENESS EVALUATIONS

Effectiveness evaluations include the KCCQ, daily step count, and daily stairs climbed.

SECONDARY AND EXPLORATORY EVALUATIONS

Secondary and exploratory evaluations include KCCQ individual domains, PGIC, PGI-S, participant satisfaction survey, the HRU and health economics data collected in this study, and associations (correlations and/or categorical association measures) between digital markers (step count and stairs climbed) and clinical events such as need for outpatient intravenous therapies, ED visits, and hospitalizations.

ADVERSE EVENT EVALUATIONS

Self-reported events will be captured from the date of the first dose of study intervention through the end of treatment (Month 3) plus 30 days. Medical claims data will be reviewed and evaluated in aggregate at the end of the 3-month, double-blind treatment period when the unblinded data are available.

STATISTICAL METHODS

Sample Size Determination

The primary effectiveness endpoint is the change from baseline in the KCCQ TSS at the end of the 3-month, double-blind treatment period. Although a 5-point improvement in the KCCQ TSS is generally viewed as clinically meaningful, recent studies have shown that improvements in the range of 3 to 5 points on the KCCQ TSS are equally meaningful in the overall HF-related health status measured by the KCCQ over a 12-week time period. Based on the evidence from these studies, the current study is powered to detect a mean change from baseline of 3 points in the KCCQ TSS between canagliflozin 100 mg and placebo groups at 12 weeks. Assuming a significance level of 5% and a standard deviation of 18 points for mean change in KCCQ TSS, a total of 1,900 participants will be randomized in this study providing approximately 95% power. This sample size is adequate to account for a potential 5% dropout rate.

Effectiveness Analyses

Primary and Secondary Effectiveness Analysis

The primary and key secondary effectiveness analyses will be based on the intent-to-treat (ITT) analysis population (using all randomized participants).

As the KCCQ TSS is measured repeatedly over time (ie, at baseline, Weeks 4, 6, and 12 respectively), the primary effectiveness endpoint (change in KCCQ TSS from baseline to Month 3) will be analyzed by a mixed effect model repeated measures (MMRM) method simultaneously adjusting for study intervention group, stratification factor (HFrEF versus HFpEF), time, time by study intervention group, and baseline KCCQ TSS value as covariates. An unstructured covariance structure will be assumed across study intervention groups to model the within-participant errors. The primary comparison will be based on the difference in least squares means between treatments at Month 3. The treatment difference in the least-squares means and their 2-sided 95% confidence interval (CI) will be provided. An MMRM model accounts for data missing at random.

A responder analysis will also be performed when comparing proportions of participants with a 5- (or 10-) point improvement in KCCQ TSS from baseline between the groups. This will be based on a two-sample difference in proportions test along with a 95% confidence interval for the difference in 2 proportions. Additionally, empirical cumulative distribution functions of the KCCQ TSS and other domain scores will be presented.

Subgroups defined by age, gender, race and baseline disease/demographic characteristics will be pre-specified in the Statistical Analysis Plan (SAP) for the primary effectiveness analysis. Details about the imputation of any missing data will be provided in the SAP. Each subgroup will be analyzed following a similar MMRM model used in the analysis of the primary effectiveness endpoint (change in KCCQ TSS

from baseline to Month 3). The treatment difference in the least-squares means and their 2-sided 95% CI will be provided from each subgroup analysis. Quantitative and/or qualitative interactions will be identified. Qualitative interactions will be investigated further by the Gail and Simon test and interpreted accordingly.

The first key secondary endpoint of daily step count will be tested once the primary endpoint is shown to be significant at the 5% significance level using a two-sided t-test. This approach will control for the overall Type I error rate at 5%.

For the second key effectiveness endpoint, an MMRM model (like that used in the analysis of the primary endpoint) will be employed to summarize changes in KCCQ individual domain scores (physical limitation, quality of life, clinical summary, and overall) from baseline to Month 3. This hypothesis will be independently tested at 5% without any control for overall Type I error rate.

Exploratory Analyses

Exploratory analyses include the following:

- summary statistics of PGIC and PGI-S scores
- associations (correlations and/or categorical association measures) between digital markers (step count and stairs climbed) and clinical events such as need for outpatient intravenous therapies, ED visits, and hospitalizations
- tabulation and comparison of total HRU and health economics data across groups
- descriptive statistics (eg, mean, median, and standard deviation) of daily stairs climbed
- association between the KCCQ change from baseline to Month 3 and the PGIC at Month 3 will be examined and summarized
- descriptive statistics of the participant's satisfaction with his/her experience with the virtual design at the end of the study.

Adverse Event Analyses

Adverse events reported to the call center will be assessed by Janssen Global Medical Safety (GMS) for causality and meeting suspected unexpected serious adverse reaction (SUSAR) reporting requirements. Adverse events will be coded according to the Medical Dictionary for Regulatory Activities (MedDRA) and will be reported by preferred term by T2DM and non-T2DM by treatment arm. Adverse events collected from claims will be categorized by diagnosis code and reported by T2DM and non-T2DM by treatment arm. Discontinuations will be summarized based on any available data.

Healthcare Resource Utilization (HRU) and Health Economics

HRU and health economics data will be descriptively summarized by study intervention group.

1.2. Schema

Figure 1: Schematic Overview of the Study

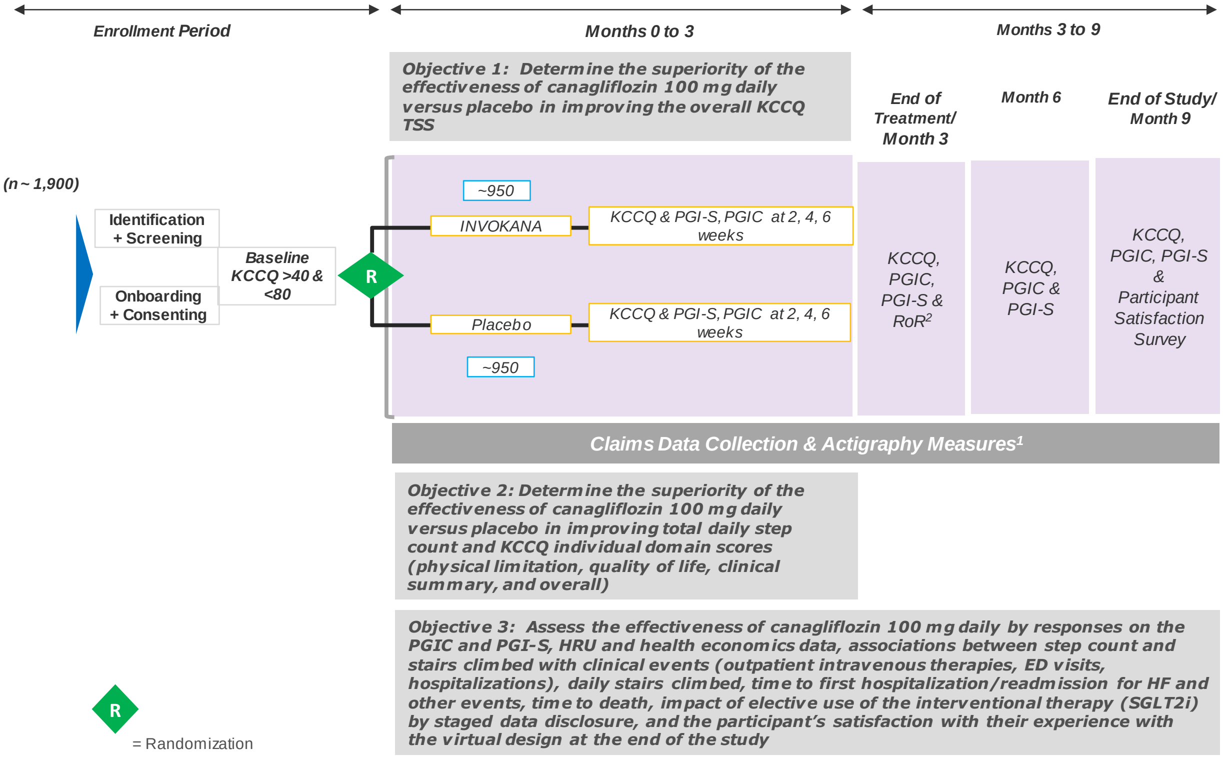

[1] – Actigraphy measures include step count, walk speed, stairs climbed
[2] – Return of Results

1.3. Schedule of Activities (SoA)

Period	Screening		Double-Blind Treatment				No Treatment		Notes
Day	-21 to 0	1+7 Baseline	14	28±3	42±3	84±3 End of Treatment	85-251-±7	252-±7 End of Study	Day -21 to 0=Screening; Day 84= end of treatment; Day 252=End of Study
Week			2	4	6	12	13-36		
Month		0				3	6	9	
**Study Procedure**									
Screening/Administrative									
Electronic Informed consent[a]	X								
Review medical history	X								
Inclusion/exclusion criteria[b]	X	X							All inclusion/exclusion verification should be completed within the 21-day screening period and before randomization
Baseline KCCQ >40 and <80		X							
Study Intervention Administration									
Randomization		X							
Dispense/administer study intervention		←				→			
Participant-reported adherence via app - weekly		←				→			
Assessments									
KCCQ			X	X	X	X	X[c]	X	Kansas City Cardiomyopathy Questionnaire
PGIC			X	X	X	X	X[c]	X	Patient Global Impression of Change
PGI-S		X	X	X	X	X	X[c]	X	Patient Global Impression of Severity
Step count and stairs climbed		←						→	
Claims data collection[d]		←						→	
Participant Satisfaction Survey (optional)								X	
Health Resource Utilization and Health Economics									
HHF for any reason and duration		←						→	Hospitalizations for heart failure
Emergency department visits		←						→	
Readmissions and duration		←						→	

Period	Screening		Double-Blind Treatment				No Treatment		Notes
Day	-21 to 0	1+7 Baseline	14	28±3	42±3	84±3 End of Treatment	85-251-±7	252-±7 End of Study	Day -21 to 0=Screening; Day 84= end of treatment; Day 252=End of Study
Week			2	4	6	12	13-36		
Month		0				3	6	9	
Ongoing Review of Participant Data									
Prohibited medications and new diagnoses		←——————————————————————→							
Reporting of adverse events[e]	←——————————————————→								

Footnotes:

a. Must be signed before first study-related activity.

b. Minimum criteria for the availability of documentation supporting the eligibility criteria are described in Source Documentation in Section 10.1, Appendix 2: Regulatory, Ethical, and Study Oversight Considerations.

c. To be completed at Month 6.

d. Includes up to 36 months of medical and pharmacy claims data for eligibility confirmation.

e. Lasts from the time of informed consent until 30 days after the end of treatment (ie, the last dose of study intervention).

2. INTRODUCTION

Canagliflozin (JNJ-28431754) is an inhibitor of sodium-glucose co-transporter 2 (SGLT2) that has been developed as an oral antihyperglycemic agent (AHA) for the treatment of patients with type 2 diabetes mellitus (T2DM). The full chemical name for JNJ-28431754 is (1S)-1,5-anhydro-1-[3-[[5-(4-fluorophenyl)-2-thienyl]methyl]-4-methylphenyl]-D-glucitol hemihydrate.

The development program for canagliflozin has investigated the compound's efficacy and safety profile both as a monotherapy and in combination with other AHAs as adjunctive treatment to diet and exercise to improve glycemic control in the treatment of adult participants with T2DM. Three of the Phase 3 and Phase 4 studies evaluated canagliflozin in special populations, including older adults with T2DM and participants with T2DM who had moderate renal impairment. Recently completed cardiovascular (CV) safety studies have evaluated canagliflozin as a treatment to reduce the risk of major adverse CV events (MACE - CV death, nonfatal myocardial infarction (MI), and nonfatal stroke) in adults with T2DM who have established cardiovascular disease (CVD) or at least 2 risk factors for CVD. Canagliflozin therapy has been shown to be associated with improved clinical outcomes in patients with T2DM and heart failure (HF) based on results from the CANVAS program (CANVAS [DIA3008] /CANVAS-Renal [DIA4003]) and CREDENCE [DNE3001] studies.

Proposed mechanisms of action of SGLT2is and recent information from the DAPA-HF and DEFINE-HF studies[13,18] suggest that the effects of the SGLT2i class of medicines in patients with HF may extend to HF patients with or without T2DM.

This randomized study is designed to assess whether canagliflozin therapy improves HF symptoms as assessed by the Total Symptom Score (TSS) of the Kansas City Cardiomyopathy Questionnaire (KCCQ) patient-reported outcome (PRO) scale in participants with HF and with or without T2DM in a real-world setting.

CANVAS Heart Failure Data

Approximately 14.4% of all participants in the CANVAS Program had a prior history of HF. While the CANVAS Program met its primary endpoint,[12] the principal secondary endpoint (superiority of canagliflozin with respect to reducing all-cause mortality) did not meet statistical significance, hence the results of the subsequent analyses examining endpoints including hospitalization for HF (HHF) must be viewed as exploratory. The hazard ratio (HR) for HHF in the combined canagliflozin group versus placebo was 0.67 (95% CI: 0.52, 0.87), with similar effects seen in the canagliflozin 100 mg and 300 mg groups in the CANVAS study.[23] These HR results resulted in choosing the lower dose of canagliflozin 100 mg for this study.

Canagliflozin lowered the risk of HHF in both patient groups: primary (participants with only risk factors for CV disease) (HR: 0.64; 95% CI: 0.35 to 1.15) and secondary (participants with a history of CV disease) (HR: 0.68; 95% CI: 0.51 to 0.90) with no evidence of statistical heterogeneity (p=0.91).

An analysis of CANVAS[23] program demonstrated that canagliflozin decreased the risk of HHF in those with pre-existing history of HF (HR: 0.51; 95% CI: 0.33 to 0.78), and in those without a history of HF (HR: 0.79; 95% CI: 0.57 to 1.09) with no evidence of statistical heterogeneity (p = 0.47). This effect of reducing the risk of HHF in those with or without a history of HF was consistent irrespective of established treatments for the prevention or management of HF (ie, inhibitors of the renin-angiotensin-aldosterone system, beta-blockers, and diuretics).

Therefore, a study to demonstrate that canagliflozin improves patient symptoms of HF would be an important advance in the clinical management of HF patients and would offer an important and valuable therapeutic option.

CREDENCE Study

The CREDENCE study (NDA 204042 S-032, approved 27 September 2019, was a large, international, randomized, double-blind, event-driven study undertaken to formally test whether canagliflozin 100 mg, administered once daily, reduces the risk of kidney failure and CV events in participants with Stage 2 or 3 chronic kidney disease (eGFR ≥30 to <90 mL/min/1.73 m^2) and macroalbuminuria (urinary ACR >300 to ≤5,000 mg/g) and T2DM who were receiving Standard of Care (SoC) therapy. SoC was defined in the National Kidney Foundation Kidney Disease Outcomes Quality Initiative consensus guidelines[19] and included a maximum tolerated labeled daily dose of an angiotensin-converting enzyme inhibitor (ACEi) or angiotensin II receptor blocker (ARB). This study was conducted between 21 February 2014 and 30 October 2018.

The renal protective effect of canagliflozin relative to placebo in a population with established CKD and T2DM was measured using a composite endpoint of doubling of serum creatinine, progression to end-stage kidney disease (ESKD) (defined as dialysis, renal transplantation, or sustained eGFR <15 L/min/1.73 m^2), and renal or CV death. The study also assessed the effects of canagliflozin on several adverse CV outcomes (CV death, HHF, nonfatal MI, nonfatal stroke, and hospitalization for unstable angina), and all-cause mortality. The results demonstrated clinical benefit of the 100 mg dose of canagliflozin in reducing important cardiovascular and renal clinical endpoints in patients with T2DM and impaired renal function.[11] Specifically, HHF (which was a secondary endpoint) was significantly decreased in the canagliflozin versus placebo group (HR= 0.61 [0.47-0.80]). Additionally, the rate of amputation was not shown to be statistically significantly increased with canagliflozin treatment in the CREDENCE study in the overall population(HR=1.11 [0.79-1.37]).

Recently, INVOKANA® was approved as the only diabetes medicine indicated to reduce the risk of ESKD, worsening of kidney function, cardiovascular death, and HHF in adults with type 2 diabetes and diabetic kidney disease (nephropathy) with a certain amount of protein in the urine.

Use of SGLT2is in Heart Failure With or Without T2DM

A mechanistic basis has been proposed for the SGLT2i class of medicines to exert beneficial effects in HF irrespective of the diabetic state and effects on glycemic control or osmotic diuresis.[22] Inhibition of sodium-hydrogen (Na/H) exchange in the kidney by SGLT2 inhibitors may reduce diuretic and endogenous natriuretic peptide resistance, and similar inhibition in the

myocardium may lead to a reduction in cardiac injury, hypertrophy, fibrosis, remodeling, and systolic dysfunction. Furthermore, the major pathophysiological derangements of HF and a preserved ejection fraction may be mitigated by the actions of SGLT2 inhibitors to reduce blood pressure, body weight, and fluid retention as well as to improve renal function.[22]

The use of canagliflozin in patients without T2DM has been studied in approximately 1,200 healthy subjects in multiple Phase 1 studies. A thorough QT/QTc study in healthy subjects (study DIA1010 NDA 204042 Sequence 0000 Module 5.3.4.1) demonstrated that canagliflozin at single therapeutic (300 mg) or supra-therapeutic (1,200 mg) doses does not lead to QT/QTc prolongation. At the 1,200 mg dose, canagliflozin C_{max} was approximately 1.4 times that for steady-state C_{max} at a 300 mg QD dose in T2DM subjects. This is important in light of the proarrhythmic myocardial substrate present in HF, in particular HFrEF, which is more prevalent in the non-type 2 diabetes population than in the type 2 diabetes population who experiences a higher incidence of HFpEF. For this study, the proposed dose of canagliflozin is 100 mg daily.

Additional evidence for the safety of canagliflozin in patients with HF comes from sub-group analyses of patients with T2DM in the CANVAS Program and in the CREDENCE study, in which there was no signal of increased arrhythmic death, or hyperkalemia – the latter of which is reassuring since many HF patients are on medications that are potassium-sparing, such as ACEis, ARBs, or mineralocorticoid receptor antagonists (MRAs), and diabetics are more predisposed to hyperkalemia with these agents than non-diabetics. CREDENCE in particular, which studied a population with a greater prevalence of kidney disease and a higher likelihood of hyperkalemia than the population in the CANVAS Program, demonstrated no increased incidence of hyperkalemia with canagliflozin versus placebo. In addition, in the CANVAS Program, there was no evidence of proportional differences in the risk of volume depletion, fracture, amputation, osmotic diuresis, acute kidney injury events, adverse events leading to discontinuation, and all serious adverse events (SAEs) between patients with or without HF at baseline (p interaction >0.160).[23]

Recently, a randomized clinical study, DAPA-HF, in 4,774 HFrEF patients with (42%) and without (58%) T2DM demonstrated safety consistent with the known profile of dapagliflozin.[13] Ninety-four percent of patients were on ACEis or ARBs, and the mean eGFR was 66 mL/min/1.73m^2. In the study, dapagliflozin met the primary composite endpoint with a statistically-significant and clinically meaningful reduction of cardiovascular death or the worsening of HF (defined as hospitalization or an urgent HF visit), compared to placebo.

Most recently a second randomized clinical study, DEFINE-HF, in 510 HFrEF was completed in 510 patients with (62%) and without (36%) T2DM.[18] Results were consistent among patients with or without T2DM, including safety analyses. Mean eGFR was 69 mL/min/1.73m^2. In the study, patients treated with dapagliflozin vs. placebo had clinically meaningful improvement in health status (KCCQ Overall Summary score ≥5 points). Finally, there are multiple ongoing randomized studies with SGLT2 inhibitors in HF with or without T2DM (see below). As such, there is ample precedent to justify starting another study in HF patients irrespective of a diagnosis of T2DM.

Dapagliflozin:

- DEFINE-HF: biomarkers, symptoms, health status and quality of life in HFrEF patients (listed in clinicaltrials.gov March 2016)
- DELIVER: Outcomes study in HFpEF patients (listed in clinicaltrials.gov Aug. 2018)
- DETERMINE-REDUCED: Exercise capacity study in HFrEF patients (listed in clinicaltrials.gov March 2019)
- DETERMINE-PRESERVED: Exercise capacity study in HFpEF patients (listed in clinicaltrials.gov March 2019)

Empagliflozin:

- EMPEROR-Reduced: Outcomes study in HFrEF patients (listed in clinicaltrials.gov Feb. 2017)
- EMPEROR-Preserved: Outcomes study in HFpEF patients (listed in clinicaltrials.gov Feb. 2017)
- EMPERIAL-Reduced: Exercise capacity study in HFrEF patients (listed in clinicaltrials.gov Feb. 2018)
- EMPERIAL-Preserved: Exercise capacity study in HFpEF patients (listed in clinicaltrials.gov Feb. 2018)

Safety

The safety profile of canagliflozin is well established having been broadly evaluated for safety in several well-designed, randomized, clinical studies. The broader Phase 3/4 canagliflozin program was conducted in 22,645 patients, including 13,278 patients treated with canagliflozin and 9,367 patients treated with a comparator, in 15 double-blind, controlled, clinical studies; among them, more than 1,100 patients reported a history of HF at baseline. In addition, there is an extensive, real-world base of adverse event reporting based on approximately 1,475,000,000 person days treated worldwide. The SGLT2 inhibitor, dapagliflozin, was studied in HFrEF patients both with and without diabetes in the DAPA-HF and the DEFINE-HF studies[13,18] (those with HFrEF and type 2 diabetes). In those studies, the side effect profile was not different between those with or without T2DM or HF. Taken together, the evidence indicates that the safety profile of SGLT2 inhibitors, of which canagliflozin is a member, is well described and broadly understood in the study population included in this study.

For the most comprehensive nonclinical and clinical information regarding canagliflozin, refer to the latest version of the Investigator's Brochure[8] and Addenda for JNJ-28431754 (canagliflozin).

The term "sponsor" used throughout this document refers to the entities listed in the Contact Information page(s), which will be provided as a separate document.

The term “study intervention” refers to canagliflozin or placebo.

2.1. Background and Study Rationale

Outcomes in studies of antidiabetic agents improving HF outcomes include canagliflozin, empagliflozin, and dapagliflozin CV that have shown a strong signal for improvement in HF endpoints with SGLT2i, but further studies are needed to validate these findings. However, very recently, the Food and Drug Administration (FDA) approved dapagliflozin to reduce the risk of HHF in adults with T2DM and established CV disease or multiple CV risk factors. Based on results of the DECLARE-TIMI 58, the possible role that SGTL2i may play in HF has been hypothesized but evaluation of the various pathways has not been performed.[27]

Heart failure contributes to one in nine deaths and is a leading cause of hospitalization in the US, yet there are limited treatment options for people living with this debilitating disease. It is highly prevalent and despite best available therapy, is associated with poor patient outcomes.[20,21] The CANVAS Program demonstrated that canagliflozin treatment in patients at cardiovascular (CV) risk was associated with improved clinical outcomes, including a reduction in the composite of CV death and HHF. A similar finding was observed with empagliflozin in the EMPA-REG cardiovascular outcomes study and dapagliflozin in DECLARE-TIMI 58.[16]

A meta-analysis of EMPA-REG OUTCOME, CANVAS PROGRAM, and DECLARE-TIMI 58 looked at a total of 20,060 T2DM patients and the CV outcomes of SGLT2i therapies. Enrolling 3,891 HF patients at baseline, this meta-analysis demonstrated similar significant reduction in HHF, regardless of the patient's baseline HF status (HR 0.68 vs HR 0.71) for those with or without HF. The benefit for these groups was affected only by renal function, with greater benefit observed in patients with lower estimated glomerular filtration rate (eGFR).[15]

In addition, the OBSERVE-4D real-world meta-analysis is the largest retrospective observational study of effects of SGLT2i in this patient population. It compared the experience of over 140,000 new canagliflozin patients to over 100,000 empagliflozin and dapagliflozin new users, as well as 460,000 new users of non-SGLT2i. Patients treated with canagliflozin showed comparable HHF reduction to other SGLT2is on the market, and greater reduced risk for HHF and below knee lower extremity amputations when compared to non-SGLT2is. This held true even when comparing patients with established CV disease to the overall study population. While limitations exist in OBSERVE-4D pertaining to the length of treatment duration with canagliflozin, this may not be entirely comparable to the randomized clinical study data discussed above. However, findings in the OBSERVE-4D meta-analysis suggest similarity to reductions observed in randomized clinical studies, and provide added confidence in the results of this meta-analysis.[24]

This meta-analysis (Table 1), as well as real-world data from CVD-REAL studies and the United States (US) Department of Defense Military Health System, support the effect of a an SGLT2i on reducing HF-related hospitalizations; however, they did not seek to differentiate or provide any insight on the effect of these therapies specifically on HF with reduced ejection fraction (HFrEF) and HFpEF phenotypes, defining the need for further investigation.[17]

Table 1: Effects of Sodium-glucose Cotransports-2 Inhibitors (SGLT-2is) on Heart Failure Outcomes

Reference	Comparator groups	HF at baseline	n	Study design	Follow-up (weeks)	Hazard ratio for HHF
EMPA-REG OUTCOME[34]	Empagliflozin vs placebo	10%	7020	RCT	161	0.65 (0.50-0.85
CANVAS[33]	Canagliflozin vs placebo	14%	10 142	RCT	126	0.67 (0.52-0.87)
DECLARE-TIMI-58[35]	Dapagliflozin vs placebo	10%	17 160	RCT	218	0.73 (0.61-0.88)
CVD-REAL[40]	SGLT-2is vs oGLDs	6%	309 056	Retrospective cohort	64	0.61 (0.51-0.73
EASEL Cohort[43]	SGLT-2is vs oGLDs	11%	25 258	Retrospective cohort	83	0.57 (0.45-0.73)

HHF, heart failure hospitalization; oGLDs, other glucose lowering drugs; RCT, randomized controlled trial.
Nassif ME, Kosiborod M. Effects of sodium-glucose co-transporter type 2 inhibitors in heart failure. Diabetes Obes Metab. 2019;21(Suppl. 2):19-23.

HFpEF is well recognized as the predominant type of HF in patients with T2DM and is possibly related to insulin resistance and obesity. Such predisposing factors are thought to be at the crux of ineffectiveness of traditional HF therapies in treating HFpEF patients, making the need for impactful therapies in this patient population even more urgent.[1,3] While more studies are underway, some encouraging data comes from the Canagliflozin for Japanese Patients with Chronic Heart Failure and Type II Diabetes (CANOSSA) study,[25] which assessed the effect of canagliflozin on Japanese patients with T2DM and stable HF. The study enrolled 35 patients, of which 33 had HFpEF, and focused on canagliflozin's effect of weight and adipose tissue reduction on these patients. Beyond reduction in glycated hemoglobin (HbA1c) and fat volumes, the study demonstrated reduction in oxidative stress, improved diastolic disfunction, and decrease in left ventricular mass, potentially linking canagliflozin use to an improved HFpEF condition.

In addition to showing an improvement in HHF, improvement in clinical symptoms, physical functioning and quality of life would be a highly important clinical outcome for patients with HF. It is believed that canagliflozin, through its diuretic, blood pressure, and body weight reduction effects would have a clinically meaningful impact on these outcomes in these patients and represent an advance in the clinical management of these patients.

2.2. Benefit/Risk Assessment

Before randomization and throughout the study, the participant's physician(s) will continue to manage the participant's background medications to achieve goals for controlling the participant's HF and T2DM (if present). Participants enrolled in this study will be managed based on the standards of care for HF and T2DM (if applicable) according to established local and regional guidelines. An Independent Scientific Advisory Committee comprised of experts in the management of patients with HF and sponsor representatives will be commissioned for this study. Other medications in the SGLT2i class of medicines are being studied in patients with HF with or without T2DM as outlined in Section 2, Introduction; however, none of these agents are currently indicated for the clinical improvement in HF symptoms.

The risks of participation in the study include exposure to study intervention, with the potential for side effects. Participant safety will be assured throughout the study by providing participants and their physicians access to a call center to ask questions and to report adverse events. In addition, the adverse effect profile of canagliflozin has been well described and will be outlined

in the informed consent form (ICF). In addition, participant prescribing information with a listing of potential adverse events will be sent to the participant in their welcome kit.

This study was designed based in general accordance with the FDA[4] and European Medicines Agency (EMA) guidance[3] on the development of medications and clinical investigations for the use of canagliflozin for the reduction of HF symptoms of HF and in consultation with Health Authorities. In addition, the design was also based on the FDA's draft guidance to industry on drug development in HF which supports measures of patients' symptoms and physical function as endpoints.[5] This study will be conducted under FDA Investigational New Drug (IND) regulations. More detailed information about the known and expected benefits and risks of JNJ-28431754 (canagliflozin) may be found in the Investigator's Brochure, Canagliflozin.[8]

3. OBJECTIVES AND ENDPOINTS

OBJECTIVES

Primary Objective

The primary objective is:

- to determine the superiority of the effectiveness of canagliflozin 100 mg daily versus placebo in participants with symptomatic HF in improving the overall KCCQ TSS.

Secondary Objectives

The first key secondary objective is:

- to determine the superiority of the effectiveness of canagliflozin 100 mg daily versus placebo in improving the total daily step count.

The second key secondary objective is:

- to determine the superiority of the effectiveness of canagliflozin 100 mg daily versus placebo in improving the KCCQ individual domain scores (physical limitation, quality of life, clinical summary, and overall).

Exploratory Objectives

The exploratory objectives are to assess the effectiveness of canagliflozin 100 mg daily in participants with symptomatic HF by:

- responses on the Patient Global Impression of Change (PGIC) and the Patient Global Impression of Severity (PGI-S)
- healthcare resource utilization (HRU) and health economics data
- associations (correlations and/or categorical association measures) between digital markers (step count and stairs climbed), and clinical events such as need for outpatient intravenous therapies, emergency department (ED) visits, and hospitalizations
- daily stairs climbed

- time to first hospitalization/readmission for heart failure
- time to first hospitalization/readmission for other events
- time to death
- impact of elective use of the interventional therapy class (SGLT2i) by staged data disclosure
- the participant's satisfaction with their experience with the virtual design at the end of the study.

ENDPOINTS

The primary effectiveness endpoint is the change in KCCQ TSS from baseline to Month 3.

The key secondary effectiveness endpoints are 1) change in the daily step count from baseline to Month 3, and 2) changes in KCCQ individual domain scores (physical limitation, quality of life, clinical summary, and overall) from baseline to Month 3.

The exploratory endpoints are the following:

- mean PGIC scores over time
- changes in PGI-S scores from baseline to Month 3
- change in number of daily stairs climbed
- change in HRU and health economics data
- number of outpatient intravenous therapies, ED visits, and hospitalizations
- time to first hospitalization/readmission for heart failure
- time to first hospitalization/readmission for other events
- time to death
- change in elective use of the interventional therapy class (SGLT2i) by staged data disclosure.

Refer to Section 8, Study Assessments and Procedures for evaluations related to endpoints.

Adverse Event Data Collection

Due to the virtual method of data collection for this study, there will be 2 types of adverse event data collection. One will be done by participants instructed to self-report any adverse events from the study intervention to a call center, and the other by an analysis of medical claims data from the participant’s provider to their insurance system. Self-reported events will be captured from the date of the first dose of study intervention through the end of treatment (Month 3) plus 30 days. Medical claims data will be reviewed and evaluated in aggregate at the end of the 3-month, double-blind treatment period when the unblinded data are available.

HYPOTHESIS

The primary hypothesis is that canagliflozin is superior to placebo as assessed by improvement in the TSS on the KCCQ scale during the 3-month, double-blind treatment period.

4. STUDY DESIGN

4.1. Overall Design

This is a randomized, double-blind, placebo-controlled, decentralized, virtual, interventional, superiority study conducted in the US in participants with symptomatic HF (stratified by HFrEF and HFpEF) to examine the improvement in the KCCQ TSS after 3 months of treatment with canagliflozin 100 mg or placebo. The study will continue for an additional 6 months without any study intervention to collect participant follow-up in a real-world setting.

The study will enroll participants from large, integrated, health networks and large physician practices in the US with the participants conducting the majority of their study-related activities through an app on a smartphone and actigraphy data from a Fitbit device. There will be no in-person clinic visits required by the study.

The study consists of a screening period and a 3-month, double-blind treatment period. Approximately 1,900 participants will be randomized into the study in a 1:1 ratio to canagliflozin 100 mg daily or placebo groups (950 in each study intervention group). After Month 3 (the end of the double-blind treatment period), the study will continue for an additional 6 months when study intervention will no longer be provided. Data collection from claims, smartphone, Fitbit device, and selected PROs will continue until Month 9 for the purpose of secondary and further exploratory endpoints analyses. In addition, the KCCQ, PGIC, and PGI-S data will be collected at 6 and 9 months. An optional participant satisfaction survey about the virtual nature of the study will be provided to the participant at the end of the study.

Participants will be of any gender, 18 years of age or older, and have clinically stable, symptomatic HF (HFrEF or HFpEF) with or without T2DM with a KCCQ score of >40 and <80 prior to randomization.

Participants should be receiving guideline recommended HF medications as prescribed by their treating physician(s) (such as ACEi, ARB, beta-adrenergic blocking agent or beta blocker [β-blocker], oral diuretics, MRA, angiotensin receptor-neprilysin inhibitor).

Study assessments include the KCCQ, Fitbit device data (step count and stairs climbed), PGIC, PGI-S, participant satisfaction survey, medical and pharmacy claims data (including up to 36 months history for eligibility confirmation), HRU and health economics (including hospitalization/readmission due to HF or other reason, ED visits, the duration of stay at each hospitalization/readmission), review of claims for prohibited medications and new diagnoses that may require discontinuation, and adverse events.

The total duration of study participation for each participant is approximately 9 months. Participants will be randomly assigned to receive canagliflozin 100 mg daily immediate-release over-encapsulated tablets (capsules) taken orally or placebo capsules for the duration of the 3-month, double-blind treatment period, followed by a 6-month, no treatment period during which claims data will continue to be collected.

Participants will be enrolled from large, integrated, health networks or large physician practices who have electronic health records (EHR) to identify participants. The primary and secondary endpoint data collection will be done via an app for a smartphone (being developed specifically for the study) as well as data from the Fitbit device.

Data integrity will be assured by automatic data collection directly from data sources (Fitbit device, Fitbit app on smartphone, structured electronic PROs on smartphone via the study app, and claims data from or to payers) without manual entry. Significant changes in actigraphy mid-study will be used to confirm that a Fitbit device remains associated with the participant to whom it is assigned. The Fitbit app on the participant's phone will collect all data from the Fitbit device, and significant latencies between wearable sensor data uploads via the app will be used to identify any smartphone and Fitbit device dissociation. In addition, the participant will be informed during the consenting process that the study Fitbit device must only be paired to the participant's smartphone and is to be used only by the participant.

There will be virtual study coordinating center support for initial participant onboarding to answer any study-related questions, assist with proper set up and use of the study app with the participant's smartphone and Fitbit device/app as data collection tools, confirming drug receipt and compliance, and to collect participant-reported adverse events and report them to the sponsor. The virtual study coordinating center will help participants appropriately set up their devices for the purposes of the study. In this particular instance, the main requirement for the Fitbit device is maximal wear time, as all of the sensor data will be collected passively. The blinded study intervention will be hand delivered directly to the participants from a drug distribution vendor. No formal on-site monitoring of the study will be performed as this is a decentralized clinical study, however, the sponsors' designee will centrally monitor participant claims, study app data, and Fitbit device data monthly for compliance with the study. For example, monitoring will include detecting non-use of the study app or Fitbit device. Claims data will also be monitored for adverse events, prohibited medications, and new diagnoses that would require discontinuation. In addition, the study app will send reminders to participants of study items to complete assessments (KCCQ, PGI-S, PGIC), and will collect weekly study drug compliance. If lack of participation is detected by any of the monitoring, the virtual study coordinating center will contact the participants for follow-up information.

Return of Results (RoR)

As each randomized participant completes the 3-month, double-blind treatment period of the study, the RoR to participants will begin. Participants will be provided with their treatment allocation, whether they had been randomized to canagliflozin or placebo. After the last randomized participant completes the 3-month, double-blind treatment period, and after the data are analyzed, participants will be provided aggregate interim study results (ie, KCCQ and activity data). At the end of the study and after all data has been analyzed, the participants will be provided with a final participant study report.

The purpose of informing and unblinding participants to this information in a staged and timely manner is 1) to demonstrate the impact of knowledge of which drug they were taking

(canagliflozin or placebo) on their subsequent choice of therapy; and, 2) to demonstrate the impact of knowledge of the study results on participants' health choices and behaviors to simulate a "learning health system" in a randomized, interventional, real-world setting.

A diagram of the study design is provided in Section 1.2, Schema.

4.2. Scientific Rationale for Study Design

Study Population

The study population selected represents participants with HF (HFrEF and HFpEF), with or without T2DM, who would potentially benefit from a drug intervention that could offer improvement in health status outcomes, such as clinical symptoms (eg, dyspnea and fatigue commonly experienced by these participants) and impaired quality of life (eg, physical limitations and emotional dysfunction such as depression and anxiety). Participants who meet the inclusion criteria for these conditions include adults of any gender 18 years of age and older, representing the demographic group in which these conditions are prevalent, and mortality and hospitalization rates are high, despite the recommendations of existing clinical practice guidelines.

Length of Study Periods

The screening period of 3 weeks allows for an appropriate length of time for screening procedures to determine study eligibility. The 3-month, double-blind treatment period provides sufficient timepoints for effectiveness assessments to demonstrate treatment differences between canagliflozin and placebo.

Placebo Control, Randomization, Blinding, Intervention Groups

A placebo control will be used to establish the frequency and magnitude of changes in clinical endpoints that may occur in the absence of active intervention. Randomization will be used to minimize bias in the assignment of participants to intervention groups, to increase the likelihood that known and unknown participant attributes (eg, demographic and baseline characteristics) are evenly balanced across intervention groups, and to enhance the validity of statistical comparisons across intervention group. Blinded intervention will be used to reduce potential bias during data collection and evaluation of clinical endpoints.

Dose and Dosage Administration Regimen

In the CANVAS program, canagliflozin reduced the relative risk of the composite of CV death or HHF compared with placebo, with a HR of canagliflozin versus placebo of 0.78 (95% CI: 0.67, 0.91). The HRs were similar in the canagliflozin 100 and 300 mg groups. The HR for HHF in the combined canagliflozin group versus placebo was 0.67 (95% CI: 0.52, 0.87), with similar effects results seen in the canagliflozin 100 and 300 mg groups. These HR results resulted in choosing the lower dose of canagliflozin 100 mg daily for this study. In addition, because few participants required titration to 300 mg/d in the CANVAS-R (DIA4003) study, and the treatment period in this study is relatively short, titration to the 300 mg/d dose is not necessary.

Kansas City Cardiomyopathy Questionnaire (KCCQ)

Patient-reported outcomes (PROs) are a measurement tool for the symptom burden and functional limitations in patients with HF and have demonstrated greater reproducibility than other clinical study measures like ejection fraction (EF) valve gradients,[10] 6-minute walk test, and a biomarker (B-type natriuretic peptide [BNP]).[26] The KCCQ (Section 10.3, Appendix 3) is a well-established PRO for use in patients with HF, with published validity and reliability. This instrument was developed and validated by Dr. John Spertus, the Lauer/Missouri Endowed Chair and Tenured Professor at the University of Missouri – Kansas City and the Clinical Director of Outcomes Research at Saint Luke's Mid America Heart Institute. After reviewing its extensive psychometric profile, the KCCQ was recently endorsed by the Center for Devices and Radiological Health as a Certified Outcome Assessment through its Medical Device Development Tools Program and is currently under review at the Center for Drug Evaluation and Research.[14]

The KCCQ is a 23-item, self-administered questionnaire and requires, on average, 4 to 6 minutes to complete, with a resulting score of 0 to 100, and higher scores indicating better health. It was developed to measure the patient's perception of their health status, including their HF symptoms, impact on physical and social function and how their HF impacts the quality of life. The KCCQ has been repeatedly used as a clinically meaningful outcome measure in CV research, patient management, and quality assessment. It has been extensively tested for its validity, reliability, and responsiveness improvement in participants with HF, and is well suited as the primary endpoint for this study. In addition, the intent of the study is to enroll participants with New York Heart Association (NYHA) class II and III. As it would be difficult to verify NYHA class with claims and EHR data, the sponsor will use a KCCQ score at baseline of >40 and <80, which is highly correlated to class II and III in HFrEF and HFpEF based on the research published by Joseph, et al.[9]

Patient Global Impression of Change (PGIC)

The PGIC (Section 10.4, Appendix 4) is a commonly accepted, validated outcome measure used in clinical studies to assess the overall change in the participant's status as it relates to the participant's HF symptoms since starting the study as rated by the participant.

Patient Global Impression of Severity (PGI-S)

The PGI-S (Section 10.5, Appendix 5) is a commonly accepted, validated outcome measure used in clinical studies to assess the change in the severity of the participant's HF symptoms since starting the study as rated by the participant.

Health Resource Utilization and Health Economics Data Collection

Treatment of patients with symptomatic HF with JNJ-28431754 (canagliflozin) versus placebo may result in lower utilization of any incident of hospitalization/readmission due to HF or other reason, ED visits, the duration of stay at each hospitalization/readmission, and discharge destination; therefore, comparison will be done across study intervention groups.

Participant Satisfaction Survey

Participants will be provided with an optional participant satisfaction survey at the end of the study to provide feedback on this virtual study and the use of the Fitbit device and smartphone to collect their information. This feedback may be important to consider when designing studies that use technology and devices.

4.2.1. Study-Specific Ethical Design Considerations

Potential participants will be fully informed of the risks and requirements of the study and, during the study, participants will be given any new information that may affect their decision to continue participation, including availability of an approved medicine for HF in the same SGLT2i class of medicines as canagliflozin. They will be told that their consent to participate in the study is voluntary and may be withdrawn at any time with no reason given and without penalty or loss of benefits to which they would otherwise be entitled. Only participants who are fully able to understand the risks, benefits, and potential adverse events of the study, and provide their consent voluntarily will be enrolled.

4.3. Justification for Dose

Canagliflozin 100 mg daily taken orally is the dose selected based on data from a similar study population in the CANVAS program that demonstrated a significant treatment effect, with no differences observed between the 100 and 300 mg doses. In addition, there are fewer dose-dependent adverse events such as volume-related adverse events associated with this dose. Refer to Section 4.2, Study Design Rationale for further details.

4.4. End of Study Definition

A participant will be considered to have completed the treatment period of the study (at 3 months), regardless of whether the participant is on the study intervention, if he or she has completed assessments at Month 3 of the double-blind treatment period or withdrew earlier from the treatment or at the time of death for participants who died prior to the Month 3 visit.

The end of study (at 9 months) is considered as the last visit shown in the Schedule of Activities for the last participant in the study. The final data from the study partner will be sent to the sponsor (or designee) after completion of the collection of the study data from the Fitbit device and the claims in the time frame specified in the Trial Agreement.

5. STUDY POPULATION

Screening for eligible participants will be performed within 21 days before randomization into the study.

The inclusion and exclusion criteria for enrolling participants in this study are described below. If there is a question about these criteria, the participant or designee must consult with the call center to resolve any issues before enrolling in the study.

For a discussion of the statistical considerations of participant selection, refer to Section 9.2, Sample Size Determination.

5.1. Inclusion Criteria

Each potential participant must satisfy all of the following criteria to be enrolled in the study:

1. any gender

2. 18 (or the legal age of consent in the jurisdiction in which the study is taking place) years of age or older

3. have clinically stable symptomatic HF (HFrEF or HFpEF):

   HFrEF a) EF <40% b) a primary diagnosis of HF or 2 outpatient visits for HF in the past 1 year

   HFpEF: a) EF >40% b) a primary diagnosis of HF or 2 outpatient visits for HF c) on a loop diuretic in the past 1 year

4. have a baseline KCCQ score of >40 and <80 prior to randomization

5. be able to read and understand English

6, possess and have sole use (eg, not shared with other users) of smartphone compatible with the Fitbit device

7. willing/able to wear the Fitbit device on a regular basis for the 9-month study period

8. must sign an electronic informed consent form (eICF) indicating that he or she understands the purpose of, and procedures required for, the study and is willing to participate in the study, including follow-up.

5.2. Exclusion Criteria

Any potential participant who meets any of the following criteria will be excluded from participating in the study:

1. currently taking an SGLT2i or within 3 months prior to enrollment

2. history of diabetic ketoacidosis or have type 1 diabetes mellitus (T1DM)

3. have acute decompensated HF (exacerbation of symptomatic HF) requiring intravenous diuretics, inotropes, or vasodilators within 4 weeks prior to enrollment

4. have stage 4 or 5 Chronic Kidney Disease (ie, eGFR <30ml/min on dialysis)

5. have a history of atraumatic amputation within past 12 months of screening, or an active skin ulcer, osteomyelitis, gangrene, or critical ischemia of the lower extremity within 6 months prior to enrollment

6. have a diagnosis of hypotension within 30 days of the screening visit

7. had major surgery within 3 months of consent or have any surgery, ie, cardiac surgery, planned during the 3-month treatment (except for minor surgery, ie, outpatient surgery under local anesthesia)

8. have a life expectancy of <6 months or current immobility

9. have known allergies, hypersensitivity, or intolerance to JNJ-28431754 (canagliflozin) or its excipients (refer to Investigator's Brochure, Canagliflozin[5])

10. be a woman participant who is pregnant, or breastfeeding, or planning to become pregnant while enrolled in this study

11. are legally incompetent

12. currently enrolled in an investigational study.

NOTE: The required source documentation to support meeting the enrollment criteria are noted in Section 10.1, Appendix 2: Regulatory, Ethical, and Study Oversight Considerations.

5.3. Prohibitions and Restrictions

Potential participants must be willing and able to adhere to the following lifestyle restrictions during the course of the study to be eligible for participation:

1. Refer to details in Section 6.5, Prohibited Medications.

2. Agree to follow all requirements that must be met during the study as noted in the Inclusion and Exclusion Criteria.

5.4. Screen Failures

Participant Identification

All potential participants who electronically sign the remote e-consent will receive a unique identifier in the study database held by a third-party representative. All data entered and collected via the study app with the participant’s smartphone and Fitbit device are time and date stamped for completeness. Those participants who electronically sign the remote e-consent but do not enroll in the study can be tracked up to the point that they no longer interact with the app. Reports will be available to permit a detailed listing of each participant from remote e consent through the study.

6. STUDY INTERVENTION

6.1. Study Interventions Administered

The study intervention, JNJ-28431754 (canagliflozin), will be provided as immediate-release, over-encapsulated tablets (capsules) or placebo capsules taken orally once daily before the first meal of the day for the duration of the 3-month, double-blind treatment period. The capsules should be swallowed intact and participants should not attempt to dissolve them in water. The following table provides a description of the study interventions.

Description of Study Interventions

**Group Name**	Group 1	Group 2
**Study Intervention Name**	canagliflozin	placebo
**Type**	drug	placebo
**Dose Formulation**	over-encapsulated tablets (capsules)	capsule
**Unit Dose Strength**	100 mg	0 mg
**Dosage Level**	100 mg	0 mg
**Route of Administration**	oral	oral
**Use**	experimental	placebo-comparator
**Sourcing**	Provided centrally by the sponsor	Provided centrally by the sponsor
**Packaging and Labeling**	Individual participant bottles	Individual participant bottles
	Child resistant	Child resistant
**Brand Name**	INVOKANA®	Not applicable

JNJ-28431754 (canagliflozin) will be manufactured and provided under the responsibility of the sponsor. Refer to the Investigator's Brochure, Canigliflozin[8] for a list of excipients.

6.2. Preparation/Handling/Storage/Accountability

All study intervention must be stored at controlled temperatures ranging from 20°C to 25°C (68°F to 77°F); excursions permitted between 15°C to 30°C (59°F to 86°F) and kept out of the reach of children.

The drug depot can refer to the pharmacy manual investigational product and procedures manual for additional guidance on study intervention preparation, handling, and storage.

The drug depot is responsible for ensuring that all study intervention received at the depot is inventoried and accounted for throughout the study. The dispensing of study intervention to the participant must be documented at the drug depot.

Study intervention must be handled in strict accordance with the protocol and the container label and must be stored at the drug depot in a secure area under appropriate environmental conditions.

Study intervention will be delivered to the participant by a qualified member of the drug depot/vendor. Study intervention will be supplied only to participants participating in the study. Participants will be instructed on how to store study intervention and how to destroy any unused study intervention at the end of the 3-month, double-blind treatment period.

6.3. Measures to Minimize Bias: Randomization and Blinding

Intervention Allocation

Procedures for Randomization and Stratification

Central randomization will be implemented in this study. Participants will be randomly assigned to 1 of 2 study intervention groups based on a computer-generated randomization schedule prepared before the study by or under the supervision of the sponsor before initiation of the study.

Randomization will be balanced by using randomly permuted blocks and will be stratified by EF (HFrEF or HFpEF) at study entry as recorded in the participant's EHR.

The interactive web response system (IxR) will generate a randomization code, participant randomization number, and kit number once consent is completed and the participant is confirmed eligible by the virtual principal investigator. The kit number will dictate the study intervention assignment and the matching study intervention bottle(s) to be shipped to the participant directly from the drug distribution vendor.

Blinding

The IxR will provide the kit number of the study intervention bottle(s) to be dispensed for each randomly assigned participant on Day 1 of the double-blind treatment period. Based on these

randomization codes, study intervention will be packaged and labeled in a manner that maintains the double-blinded nature of the study.

The drug depot will not be provided with randomization codes. The codes will be maintained within the IxR, which has the functionality to allow the virtual principal investigator or designee to break the blind for an individual participant.

Under normal circumstances, the blind should not be broken until participants have completed the 3-month, double-blind treatment period. The virtual principal investigator or designee may in an emergency determine the identity of the study intervention by contacting the IxR. While the responsibility to break the study intervention code in emergency situations resides solely with the virtual principal investigator, it is recommended that the virtual principal investigator contact the sponsor or its designee if possible, to discuss the particular situation, before breaking the blind. Telephone contact with the sponsor or its designee will be available 7 days per week. In the event the blind is broken, the sponsor must be informed as soon as possible. The date and reason for the unblinding must be documented in the study database. The documentation received from the IxR indicating the code break must be retained with the participant's source documents in a secure manner. All randomization codes will be released after completion of the study. The translation of randomization codes into treatment and control groups will be disclosed only to those authorized. Participants who have had their intervention assignment unblinded should continue to perform their scheduled evaluations.

6.4. Study Intervention Compliance

Participants will be instructed and notified by the study app weekly to confirm the number of study intervention capsules that they took that week. Study intervention compliance data will be monitored bi-weekly and if compliance is not >80%, the participant will be contacted by the call center who will re-educate the participant on the importance of taking study intervention daily.

6.5. Prohibited Medications

Prohibited medications include other SGLT2i medications (including commercially available canagliflozin); participants must not take any other investigational agents during the study.

Medications will be reviewed monthly through claims data by the sponsor or designee. The sponsor must be notified as soon as possible of any instances in which prohibited therapies are administered.

7. DISCONTINUATION OF STUDY INTERVENTION AND PARTICIPANT DISCONTINUATION/WITHDRAWAL

7.1. Discontinuation of Study Intervention

A participant's study intervention must be discontinued during the 3-month, double-blind treatment period if:

- the participant has started another SGLT2i

- the participant has been diagnosed with T1DM
- for safety reasons or tolerability reasons (eg, adverse event), it is in the best interest of the participant to discontinue study intervention
- the participant develops severe renal impairment or End Stage Renal Disease, or is on dialysis
- the participant becomes pregnant
- the participant develops diabetic ketoacidosis
- the participant develops a new active skin ulcer, osteomyelitis, gangrene, or critical ischemia of the lower extremity or has an amputation.

The participant may choose to discontinue the study intervention only and remain in the study. In this case, the participant will continue to perform assessments (KCCQ, PGI-S, PGIC) and allow continued passive data collection from the app, Fitbit, and claims. If contacted during the study, the call center or designee will inform the participant of the importance to the study and results to have ongoing ascertainment of assessments and vital status.

Additional information on informed consent for collection of vital status can be found in Section 10.1, Appendix 2: Regulatory, Ethical, and Study Oversight Considerations.

7.1.1. Temporary Discontinuation of Study Intervention

Study intervention may be temporarily discontinued; however, these interruptions should be kept to a minimum. Study intervention can be resumed when the participant, the virtual principal investigator, or the participant’s physician deems it is appropriate to do so.

7.2. Participant Discontinuation/Withdrawal From the Study

A participant will be withdrawn from the study for any of the following reasons:

- lost to follow-up
- withdrawal of consent
- death.

In the event a participant withdraws consent and does not agree to any kind of follow-up and specifically refuses any further contact with the call center or designee, this must be documented in the study database. If applicable, vital status will be obtained at study end through the participant’s physician, medical claims, or public information according to local guidelines and as allowed by local regulations.

If a participant is lost to follow-up, every reasonable effort must be made by the call center or designee to contact the participant to determine vital status (eg, alive or dead) and the reason for discontinuation/withdrawal. This should include repeated telephone calls, certified letters, email requests, etc. The study screeners and app will obtain both primary and secondary telephone contact numbers (eg, home, work, and smartphone numbers), as well as other contact information (eg, email addresses) from participants before randomization. In addition, the call

center should emphasize the importance of follow-up information to the participant before randomization. The measures taken to obtain follow-up information must be documented.

Unless consent is specifically withdrawn, participants are expected to be followed up through 1 of the alternative follow-up mechanisms described below.

Before withdrawing from the study, participants who request to withdraw from the study should be asked if they agree to be contacted to obtain follow-up information.

8. STUDY ASSESSMENTS AND PROCEDURES

Overview

The Schedule of Activities (SoA) summarizes the frequency and timing of effectiveness, medical resource utilization, health economic, and safety measurements applicable to this study.

Health Resource Utilization (HRU) and health economics data will be collected. Refer to Section 8.2.4, Healthcare Resource Utilization and Health Economics for details.

Study-Specific Materials

The participants will be provided with the following supplies prior to starting the study:

- study intervention supply
- Fitbit device
- instruction manual(s) and patient drug information handouts.

8.1. Effectiveness Assessments

The primary effectiveness endpoint (KCCQ TSS) will be assessed at baseline and Weeks 2, 4, 6, and 12 during the double-blind treatment period (first 3 months) and afterwards at 6 and 9 months when no study intervention is administered. The following sensor data will be obtained from the Fitbit: step count and stairs climbed.

8.1.1. KCCQ Scores

The KCCQ is a 23-item, self-administered questionnaire on the study app and requires, on average, 4 to 6 minutes to complete. It was developed to measure the patient’s perception of their health status, including their HF symptoms, impact on physical and social function and how their HF impacts the quality of life. It scored by assigning a number for each response beginning with 1 that implies the lowest level of functioning and summing items within each domain, dividing by the range and multiplying by 100.[7] Missing values within each domain are assigned the average of the answered items within that same domain, presuming that at least half of the items within that domain are completed. Scale scores are transformed into values ranging from 0 to 100, with higher scores indicating more favorable health status.[7]

The answers provided by participants to the KCCQ’s questions are used to calculate scores for the following 10 scales:

- Physical Limitation: a measure of how much a patient's routine activities are hampered by symptoms of HF
- Symptom Stability: a measure of whether a participant's symptoms have changed over the past 2 weeks
- Symptom Frequency: a measure of how often a participant has HF symptoms
- Symptom Burden: a measure of how much the participant's symptoms bother them
- Total Symptom: a combined measure of the symptom frequency and burden scales
- Social Limitation: a measure of how much a participant's social and work activities are limited by their HF symptoms
- Self-Efficacy: a measure of how well a participant can manage her care, find answers and help
- Quality of Life: a measure of the overall impact of a participant's HF on their perceived quality of life
- Clinical Summary: a combined measure of symptoms and physical limitations, congruent with the considerations a physician uses in assigning their NYHA classification
- Overall Summary: a combined measure of the participant's physical limitation, total symptom, social limitation, and quality of life scores.

8.2. Exploratory Assessments

8.2.1. Patient Global Impression of Change (PGIC) Scores

The PGIC is a global index that is used to rate the overall status of the participant related to the participant's condition. It is rated by the participant and is based on the single question, "Since the start of the treatment you've received in this study, your heart failure symptoms are", where 1=very much improved, 2=somewhat improved, 3=a little improved, 4=no change, 5=a little worse, 6=somewhat worse, and 7=very much worse. Refer to Section 10.4, Appendix 4: Patient Global Impression of Change (PGIC).

8.2.2. Patient Global Impression of Severity (PGI-S) Scores

The PGI-S is a global index that is used to rate the severity of a specific condition. It is rated by the participant and is based on the single question, "Considering all aspects of your heart failure symptoms right now, would you say your heart failure symptoms are", where 1=none, 2=mild, 3=moderate, 4=severe, and 5=very severe. Refer to Section 10.5, Appendix 5: Patient Global Impression of Severity (PGI-S).

8.2.3. Participant Satisfaction Survey

An optional participant satisfaction survey to assess the participant's satisfaction with the virtual design of the study and use of the Fitbit device in the study will be provided to the participant at the end of study. Refer to Section 10.6, Appendix 6: Participant Satisfaction Survey.

8.2.4. Healthcare Resource Utilization and Health Economics

Healthcare Resource Utilization (HRU) and health economics data associated with medical encounters will be collected for all participants during the study. Protocol-mandated procedures, tests, and encounters are excluded. The data collected may be used to conduct exploratory economic analyses and will include:

- number and duration of first hospitalizations/readmissions (overall inpatient visit length of stay) for HF or any other reason
- number and duration of first hospitalizations/readmissions (overall inpatient visit length of stay) for any other events
- number of emergency room visits.

8.3. Adverse Events and Serious Adverse Events and Special Situations

Participants will be instructed to self-report any adverse events from the study intervention to a call center. In addition, this information may be discovered by the call center during interaction with the participant or those listed above. Once identified, the call center will complete and submit a solicited safety reporting form to Janssen Global Medical Safety (GMS) per the sponsor's standard operating procedure. Queries for these cases will go to the call center/and virtual principal investigator as appropriate. If follow-up is needed with the participant's treating physician, it will be done either through the virtual principal investigator or directly with the treating physician. Participants will agree to provide their treating physician contact information in the ICF. All SAEs will be assessed by Janssen GMS for potential suspected unexpected serious adverse reactions (SUSARs) for expedited regulatory reporting.

Medical claims data will be reviewed and evaluated including potential SUSAR assessment and regulatory reporting in aggregate at the end of the 3-month, double-blind treatment period when the unblinded data are available. Adverse events will be coded according to the Medical Dictionary for Regulatory Activities (MedDRA) and will be reported by preferred term and analyzed and reported by T2DM and non-T2DM. Adverse events collected from claims will be aggregated based on diagnosis codes for the incidences of non-endpoint SAEs (eg, SAEs leading to hospitalizations) and adverse events of interest between groups reported by T2DM and non-T2DM by treatment arms. Discontinuations will be summarized based on any available data.

8.3.1. Time Period and Frequency for Collecting Adverse Event and Serious Adverse Event Information

All Adverse Events

All participant-reported adverse events suspected to be related to the study intervention, whether serious or non-serious, will be collected from the time a signed and dated ICF is obtained until the participant completes their participation in the 3-month treatment period plus 30 days. In addition, the sponsor will evaluate any safety information that is spontaneously reported by the call center beyond the time frame specified in the protocol.

8.3.2. Regulatory Reporting Requirements for Serious Adverse Events

The sponsor assumes responsibility for appropriate reporting of adverse events to the regulatory authorities. The sponsor will also report to the virtual principal investigator (and the head of the investigational institute where required) all SUSARs. The virtual principal investigator (or sponsor where required) must report SUSARs to the appropriate Independent Ethics Committee/Institutional Review Board (IEC/IRB) that approved the protocol unless otherwise required and documented by the IEC/IRB. A SUSAR will be reported to regulatory authorities unblinded. The virtual principal investigator and IEC/IRB will receive a blinded SUSAR summary, unless otherwise specified.

8.3.3. Pregnancy

Female participants and male participants with a partner becoming pregnant will be instructed to call the call center for any pregnancy. The call center will report the pregnancy to the sponsor within 24 hours of their knowledge of the event using the appropriate pregnancy notification form. Abnormal pregnancy outcomes (eg, spontaneous abortion, fetal death, stillbirth, congenital anomalies, ectopic pregnancy) are considered adverse events and must also be self-reported by the participants to the call center. Any participant who becomes pregnant during the study must be promptly withdrawn from the study and discontinue further study intervention.

Follow-up information regarding the outcome of the pregnancy and any postnatal sequelae in the infant will be required.

8.3.4. Adverse Events of Interest

Adverse events of interest include all severe hypoglycemic events, acute kidney injury, fractures, diabetic ketoacidosis, and lower limb amputation.

9. STATISTICAL CONSIDERATIONS

Statistical analysis will be done by the sponsor or under the authority of the sponsor. A general description of the statistical methods to be used to analyze the effectiveness and safety data is outlined below. Specific details will be provided in the SAP.

The primary study objective will be addressed by comparing the mean change in KCCQ TSS from baseline to Month 3 between the study intervention groups. The key secondary study objective will involve a comparison between the 2 randomized groups in terms of the mean difference in daily step counts from baseline to Month 3. For analyzing the primary, secondary, and exploratory objectives, an intent-to-treat (ITT) population comprising of all randomized participants will be used. Specific imputation rules for missing data (eg, missing KCCQ TSS, daily step count) will be applied, if warranted, and will be specified in the SAP before the final database lock. Descriptive statistics such as mean, median, standard deviation, interquartile range, minimum and maximum will be used to summarize continuous variables. Counts and proportions will be used to summarize categorical variables. Graphical data displays (eg, box plots) may be employed to summarize data. For time to event variables, Kaplan-Meier estimates over time will be plotted. All statistical tests will be two-sided and performed at the 5%

significance level unless otherwise specified. No multiplicity adjustment to the overall Type I error rate will be applied when analyzing the exploratory variables.

9.1. Statistical Hypotheses

For the primary effectiveness endpoint, the null hypothesis is that the canagliflozin 100 mg is not different from placebo with respect to the mean KCCQ TSS change from baseline at Month 3. For the first key secondary effectiveness endpoint of daily step count, the null hypothesis is that canagliflozin 100 mg is not different from placebo with respect to the mean daily step count.

9.2. Sample Size Determination

The primary effectiveness endpoint is the change from baseline in the KCCQ TSS at the end of the 3-month double-blind treatment period. Although a 5-point improvement in the KCCQ TSS is generally viewed as clinically meaningful,[2,26] recent studies[13,18] have shown that improvements in the range of 3 to 5 points on the KCCQ TSS are equally meaningful in the overall HF-related health status measured by the KCCQ over a 12-week time period. Based on the evidence from these studies, the current study is powered to detect a mean change from baseline of 3 points in the KCCQ TSS between canagliflozin 100 mg and placebo groups at 12 weeks. Assuming a significance level of 5% and a standard deviation of 18 points for mean change in KCCQ TSS, a total of 1,900 participants will be randomized in this study providing approximately 95% power. This sample size is adequate to account for a potential 5% dropout rate.

9.3. Populations for Analyses

For purposes of analysis, the following populations are defined:

Table 2: Analysis Populations

Population	Description
Enrolled	All participants who sign the ICF
Intent to Treat (ITT)	All randomized participants
Per Protocol (PP)	A subset of the ITT population. Participants with major protocol deviations will be excluded from the PP population. Major protocol deviations will be defined in the SAP.

9.4. Statistical Analyses

9.4.1. Handling of Missing Data

Missing data affecting the primary endpoint change in KCCQ TSS will be addressed via the MMRM method that assumes missingness at random (MAR). Specific imputation rules including statistical modeling approaches may be employed when such an assumption is not valid. In cases where individual items in the KCCQ domains are missing or not completed at required times, appropriate rules to impute missing item scores will be specified based on the KCCQ manual. Sensitivity analyses will be conducted to assess impact of missing data on key study results. Further details about the imputation rules and sensitivity analyses will be provided in the SAP.

9.4.2. Effectiveness Analyses

9.4.2.1. Primary and Secondary Effectiveness Analyses

The primary and key secondary effectiveness analyses will be based on the ITT analysis population (using all randomized participants).

As KCCQ TSS is measured repeatedly over time (ie, at baseline, Week 2, Week 4, Week 6, and Week 12 respectively), the primary effectiveness endpoint (change in KCCQ TSS from baseline to Month 3) will be analyzed by a mixed effect model repeated measures (MMRM) method simultaneously adjusting for study intervention group, stratification factor (HFrEF versus HFpEF), time, time by study intervention group, and baseline KCCQ TSS value as covariates. An unstructured covariance structure will be assumed across study intervention groups to model the within-participant errors. The primary comparison will be based on the difference in least squares between treatments at Month 3. The treatment difference in the least-squares means and their 2-sided 95% confidence interval (CI) will be provided. An MMRM model accounts for data missing at random.

A responder analysis will also be performed when comparing proportions of participants with a 5- (or 10-) point improvement in KCCQ TSS from baseline between the study intervention groups. This will be based on a two-sample difference in proportions test along with a 95% confidence interval for the difference in 2 proportions. Additionally, empirical cumulative distribution functions of the KCCQ TSS and other domain scores will be presented.

The primary effectiveness endpoint will also be analyzed by subgroups defined by age, gender, race and key baseline disease/demographic characteristics using the MMRM approach described above. Further details will be provided in the SAP. Quantitative and/or qualitative interactions for each subgroup and treatment will be identified. Qualitative interactions will be investigated further by the Gail and Simon test[6] and interpreted accordingly.

The first key secondary endpoint of daily step count will be tested once the primary endpoint is shown to be significant at the 5% significance level using a two-sided t-test. This approach will control for the overall Type I error rate at 5%.

For the second key effectiveness endpoint, an MMRM model (like that used in the analysis of the primary endpoint) will be employed to summarize changes in KCCQ individual domain scores (physical limitation, quality of life, clinical summary, and overall) from baseline to Month 3. This hypothesis will be independently tested at 5% without any control for overall Type I error rate.

9.4.2.2. Exploratory Analyses

Exploratory analyses will be performed with no adjustment for multiplicity and include the following:

- summary statistics of PGIC and PGI-S scores

- associations (correlations and/or categorical association measures) between digital markers (step count and stairs climbed) and clinical events such as need for outpatient intravenous therapies, ED visits, and hospitalizations
- tabulation and comparison of total HRU and health economics data across groups.
- descriptive statistics (eg, mean, median, and standard deviation) of daily stairs climbed
- association between the KCCQ change from baseline to Month 3 and the PGIC at Month 3 will be examined and summarized
- descriptive statistics of the participant's satisfaction with his/her experience with the virtual design at the end of the study.

9.4.3. Adverse Events Analysis

Adverse events reported to the call center will be assessed by Janssen GMS for causality and meeting SUSAR reporting requirements. Adverse events will be coded according to MedDRA and will be reported by preferred term by T2DM and non-T2DM and by treatment arm. Adverse events collected from claims will be aggregated based on diagnosis codes for the incidences of non-endpoint SAEs (eg, SAEs leading to hospitalizations) and adverse events of interest between groups reported by T2DM and non-T2DM participants by treatment arm. Discontinuations will be summarized based on any available data. Adverse events reported from the call center will be reconciled with those from the claims data to avoid duplicate reports.

9.4.4. Healthcare Resource Utilization and Health Economics Analyses

Healthcare resource utilization (HRU) and health economics data will be descriptively summarized by intervention group.

9.5. Interim Analysis

No interim analysis is planned for this study.

10. SUPPORTING DOCUMENTATION AND OPERATIONAL CONSIDERATIONS

10.1 Appendix 1: Abbreviations and Trademarks

ACEi	angiotensin-converting enzyme inhibitor
AE	adverse event
AHA	antihyperglycemic agent
ARB	angiotensin II receptor blocker
ARNi	angiotensin receptor-neprilysin inhibitor
BB	beta blocker
CANVAS	short title for study, Canagliflozin Cardiovascular Assessment Study
CANVAS-R	short title for study, Canagliflozin Cardiovascular Assessment Study-Renal
CI	confidence interval
CREDENCE	short title for study, Canagliflozin and Renal Events in Diabetes with Established Nephropathy Clinical Evaluation Trial
CV	cardiovascular
CVD	cardiovascular disease
DKA	diabetic ketoacidosis
DMC	Data Monitoring Committee
ED	emergency department
EHR	electronic health record
EMPA	empagliflozin
ESKD	End Stage Kidney Disease
GCP	Good Clinical Practice
GMS	Global Medical Safety
HF	heart failure
HFpEF	heart failure with preserved ejection fraction
HFrEF	heart failure with reduced ejection fraction
HHF	hospitalization for heart failure
HR	hazard ratio
HRU	healthcare resource utilization
ICF	informed consent form
ICH	International Conference on Harmonisation
IEC	Independent Ethics Committee
INVOKANA	canagliflozin
IRB	Institutional Review Board
ITT	intent-to-treat
IxR	interactive web response system
KCCQ	Kansas City Cardiomyopathy Questionnaire
MACE	major adverse cardiovascular events
MAR	missing at random
MDDT	Medical Device Development Tools
MedDRA	Medical Dictionary for Regulatory Activities
MI	myocardial infarction
MMRM	mixed effect model repeated measure
NYHA	New York Heart Association
PGIC	Patient Global Impression of Change
PGI-S	Patient Global Impression of Severity
PQC	Product Quality Complaint
PRO	patient-reported outcome(s) (paper)
SAP	statistical analysis plan
SGLT2	sodium-glucose co-transporter 2
SGLT2i	sodium-glucose co-transporter 2 inhibitor
SoA	schedule of activities

SUSAR	suspected unexpected serious adverse reaction
T1DM	type 1 diabetes mellitus
T2DM	type 2 diabetes mellitus
TSS	Total Symptom Score
US	United States

10.2 Appendix 2: Regulatory, Ethical, and Study Oversight Considerations

REGULATORY AND ETHICAL CONSIDERATIONS

Virtual Principal Investigator Responsibilities

The virtual principal investigator or designee is responsible for ensuring that the study is performed in accordance with the protocol, current ICH guidelines on Good Clinical Practice (GCP), and applicable regulatory and country-specific requirements.

Good Clinical Practice is an international ethical and scientific quality standard for designing, conducting, recording, and reporting studies that involve the participation of human participants. Compliance with this standard provides public assurance that the rights, safety, and well-being of study participants are protected, consistent with the principles that originated in the Declaration of Helsinki, and that the study data are credible.

Protocol Amendments

The sponsor will not modify this protocol without a formal amendment by the sponsor. All protocol amendments must be issued by the sponsor. Protocol amendments must not be implemented without prior IEC/IRB approval, or when the relevant competent authority has raised any grounds for non-acceptance, except when necessary to eliminate immediate hazards to the participants, in which case the amendment must be promptly submitted to the IEC/IRB and relevant competent authority. When the change(s) involve only logistic or administrative aspects of the study, the IEC/IRB (where required) only needs to be notified.

During the course of the study, in situations where a departure from the protocol is unavoidable, the virtual principal investigator or designee will contact the appropriate sponsor representative listed in the Contact Information page(s), which will be provided as a separate document. Except in emergency situations, this contact should be made before implementing any departure from the protocol. In all cases, contact with the sponsor must be made as soon as possible to discuss the situation and agree on an appropriate course of action. The data recorded in the source documents will reflect any departure from the protocol, and the source documents will describe this departure and the circumstances requiring it.

Regulatory Approval/Notification

This protocol and any amendment(s) must be submitted to the appropriate regulatory authorities in each respective country, if applicable. A study may not be initiated until all local regulatory requirements are met.

Required Prestudy Documentation

The following documents must be provided to the sponsor before shipment of study intervention to the drug depot:

- protocol and amendment(s), if any, signed and dated by the principal investigator
- a copy of the dated and signed (or sealed, where appropriate per local regulations), written IEC/IRB approval of the protocol, amendments, ICF, any recruiting materials, and if applicable, participant compensation programs. This approval must clearly identify the specific protocol by title and number and must be signed (or sealed, where appropriate per local regulations) by the chairman or authorized designee.
- name and address of the IEC/IRB, including a current list of the IEC/IRB members and their function, with a statement that it is organized and operates according to GCP and the applicable laws and regulations. If accompanied by a letter of explanation, or equivalent, from the IEC/IRB, a general statement may be substituted for this list. If the principal investigator is a member of the IEC/IRB, documentation must be obtained to state that this person did not participate in the deliberations or in the vote/opinion of the study.
- regulatory authority approval or notification, if applicable
- signed and dated statement of virtual principal investigator (eg, Form FDA 1572) and current curriculum vitae (CV), if applicable
- documentation of virtual principal investigator or designee qualifications (eg, curriculum vitae)
- completed virtual principal investigator or designee financial disclosure form, where required
- signed and dated clinical trial agreement, which includes the financial agreement
- any other documentation required by local regulations.

The following documents must be provided to the sponsor before enrollment of the first participant:

- completed virtual principal investigator or designee financial disclosure forms
- documentation of virtual principal investigator or designee qualifications (eg, curriculum vitae).

Independent Ethics Committee or Institutional Review Board

Before the start of the study, the virtual principal investigator or designee (or sponsor where required) will provide the IEC/IRB with current and complete copies of the following documents (as required by local regulations):

- final protocol and, if applicable, amendments
- sponsor-approved ICF (and any other written materials to be provided to the participants)
- Investigator's Brochure, Canigliflozin[8] (or equivalent information) and amendments/addenda

- sponsor-approved participant recruiting materials
- information on compensation for study-related injuries or payment to participants for participation in the study, if applicable
- virtual principal investigator or designee curriculum vitae or equivalent information (unless not required, as documented by the IEC/IRB)
- information regarding funding, name of the sponsor, institutional affiliations, other potential conflicts of interest, and incentives for participants
- any other documents that the IEC/IRB requests to fulfill its obligation.

This study will be undertaken only after the IEC/IRB has given full approval of the final protocol, amendments (if any, excluding the ones that are purely administrative, with no consequences for participants, data or study conduct, unless required locally), the ICF, applicable recruiting materials, and participant compensation programs, and the sponsor has received a copy of this approval. This approval letter must be dated and must clearly identify the IEC/IRB and the documents being approved.

During the study, the principal investigator (or sponsor where required) will send the following documents and updates to the IEC/IRB for their review and approval, where appropriate:

- protocol amendments (excluding the ones that are purely administrative, with no consequences for participants, data or study conduct)
- revision(s) to ICF and any other written materials to be provided to participants
- if applicable, new or revised participant recruiting materials approved by the sponsor
- new edition(s) of the Investigator's Brochure, Canigliflozin[8] and amendments/addenda
- summaries of the status of the study at intervals stipulated in guidelines of the IEC/IRB (at least annually)
- reports of adverse events that are of interest, special interest, serious, unlisted/unexpected, and associated with the study intervention
- new information that may adversely affect the safety of the participants or the conduct of the study
- deviations from or changes to the protocol to eliminate immediate hazards to the participants
- report of deaths of participants under the virtual principal investigator's care
- notification if a new virtual principal investigator is responsible for the study
- development Safety Update Report and Line Listings, where applicable
- any other requirements of the IEC/IRB.

For all protocol amendments (excluding the ones that are purely administrative, with no consequences for participants, data or study conduct), the amendment and applicable ICF revisions must be submitted promptly to the IEC/IRB for review and approval before implementation of the change(s).

At least once a year, the IEC/IRB will be asked to review and reapprove this study, where required.

At the end of the study, the virtual principal investigator or designee (or sponsor where required) will notify the IEC/IRB about the study completion (if applicable, the notification will be submitted through the head of investigational institution).

Other Ethical Considerations

For study-specific ethical design considerations, refer to Section 4.2.1.

FINANCIAL DISCLOSURE

The virtual principal investigator or designee will provide the sponsor with sufficient, accurate financial information in accordance with local regulations to allow the sponsor to submit complete and accurate financial certification or disclosure statements to the appropriate regulatory authorities. The virtual principal investigator is responsible for providing information on financial interests during the course of the study and for 1 year after completion of the study.

Refer to Required Prestudy Documentation (above) and contracts for details on financial disclosure.

INFORMED CONSENT (REMOTE E-CONSENT) PROCESS

Each participant must give remote e-consent according to local requirements after the nature of the study has been fully explained. The remote e-consent must be electronically signed within the study app before the performance of any study-related activity. The remote e-consent that is/are used must be approved by both the sponsor and by the reviewing IRB and be in a language that the participant can read and understand. The remote e-consent should be in accordance with principles that originated in the Declaration of Helsinki, current ICH and GCP guidelines, applicable regulatory requirements, and sponsor policy. After having obtained the consent, a copy of the remote e-consent must be sent to the participant.

In this study, a remote e-consent process will be utilized. Before enrollment in the study, the participant will have an opportunity to read through the remote e-consent which will detail the aims, methods, reasonably anticipated benefits, and potential hazards of the study, and any discomfort participation in the study may entail. The remote e-consent will explain that their participation is voluntary and that they may withdraw consent to participate at any time. They will be informed that choosing not to participate will not affect the care they will receive. Finally, they will be told that their records may be accessed by health authorities and authorized sponsor personnel without violating their confidentiality, to the extent permitted by the applicable law(s) or regulations. By electronically signing the remote e-consent the participant is authorizing such access, which includes permission to obtain information about his or her survival status. It also denotes that they are agreeing to allow recontact by the study sponsor for the purpose of obtaining consent for additional evaluations, if needed.

If the participant has any questions about the study related to the remote e-consent process prior to providing their electronic signature, they will be provided with an opportunity to discuss these question with the virtual principal investigator via phone and/or a contact at the study call center. Once the participant understands all aspects of the remote e-consent, consent should be appropriately recorded by means of the participant's personally dated signature via electronic signature. After having obtained the consent, the participant will receive a copy of the remote e-consent via email (after the participant has verified his or her email address) for their records.

DATA PROTECTION

Privacy of Personal Data

The collection and processing of personal data from participants enrolled in this study will be limited to those data that are necessary to fulfill the objectives of the study.

These data must be collected and processed with adequate precautions to ensure confidentiality and compliance with applicable data privacy protection laws and regulations. Appropriate technical and organizational measures to protect the personal data against unauthorized disclosures or access, accidental or unlawful destruction, or accidental loss or alteration must be put in place. Sponsor personnel whose responsibilities require access to personal data agree to keep the identity of participants confidential.

The informed consent obtained from the participant includes explicit consent for the processing of personal data and for the investigator/institution to allow direct access to his or her original medical records (source data/documents) for study-related monitoring, audit, IEC/IRB review, and regulatory inspection. This consent also addresses the transfer of the data to other entities.

The participant has the right to request through the virtual principal investigator access to his or her personal data and the right to request rectification of any data that are not correct or complete. Reasonable steps will be taken to respond to such a request, taking into consideration the nature of the request, the conditions of the study, and the applicable laws and regulations.

COMMITTEES STRUCTURE

An Independent Scientific Advisory Committee comprised of experts in the management of patients with HF and sponsor representatives will be commissioned for this study. Committee membership and responsibilities will be documented in the committee charter(s).

PUBLICATION POLICY/DISSEMINATION OF CLINICAL STUDY DATA

All information, including but not limited to information regarding JNJ-28431754 (canagliflozin) or the sponsor's operations (eg, patent application, formulas, manufacturing processes, basic scientific data, prior clinical data, formulation information) supplied by the sponsor to the investigator and not previously published, and any data, generated as a result of this study, are considered confidential and remain the sole property of the sponsor. The virtual principal investigator agrees to maintain this information in confidence and use this information only to

accomplish this study, and will not use it for other purposes without the sponsor's prior written consent.

The virtual principal investigator understands that the information developed in the study will be used by the sponsor in connection with the continued development of JNJ-28431754 (canagliflozin), and thus may be disclosed as required to other clinical investigators or regulatory agencies. To permit the information derived from the clinical studies to be used, the virtual principal investigator is obligated to provide the sponsor with all data obtained in the study.

The results of the study will be reported in a Clinical Study Report generated by the sponsor and will contain data as per protocol. Recruitment performance or specific expertise related to the nature and the key assessment parameters of the study will be used to determine a coordinating investigator for the study, if appropriate.

Study participant identifiers will not be used in publication of results. Any work created in connection with performance of the study and contained in the data that can benefit from copyright protection (except any publication by the investigator as provided for below) shall be the property of the sponsor as author and owner of copyright in such work.

Consistent with Good Publication Practices and International Committee of Medical Journal Editors guidelines, the sponsor shall have the right to publish such primary (multicenter) data and information without approval from the virtual principal investigator. The virtual principal investigator has the right to publish data after the primary data are published. If the virtual principal investigator wishes to publish information from the study, a copy of the manuscript must be provided to the sponsor for review at least 60 days before submission for publication or presentation. Expedited reviews will be arranged for abstracts, poster presentations, or other materials. If requested by the sponsor in writing, the investigator will withhold such publication for up to an additional 60 days to allow for filing of a patent application. In the event that issues arise regarding scientific integrity or regulatory compliance, the sponsor will review these issues with the investigator. The sponsor will not mandate modifications to scientific content and does not have the right to suppress information. For multicenter study designs and substudy approaches, secondary results generally should not be published before the primary endpoints of a study have been published. Similarly, the principal investigator will recognize the integrity of the study by not submitting data for publication data until the results from the completed study have been submitted for publication, within 18 months after the study end date. Authorship of publications resulting from this study will be based on the guidelines on authorship, such as those described in the International Committee of Medical Journal Editors Recommendations for the Conduct, Reporting, Editing and Publication of Scholarly Work in Medical Journals, which state that the named authors must have made a significant contribution to the conception or design of the work; or the acquisition, analysis, or interpretation of the data for the work; and drafted the work or revised it critically for important intellectual content; and given final approval of the version to be published; and agreed to be accountable for all aspects of the work in ensuring that questions related to the accuracy or integrity of any part of the work are appropriately investigated and resolved.

Registration of Clinical Studies and Disclosure of Results

The sponsor will register and disclose the existence and the results of clinical studies as required by law.

DATA QUALITY ASSURANCE

SOURCE DOCUMENTS

At a minimum, source documents consistent in the type and level of detail with that commonly recorded as a basis for standard medical care must be available for the following: participant identification, eligibility, and study identification; record of all adverse events and follow-up of adverse events; and date of study completion and reason for early discontinuation of study intervention or withdrawal from the study, if applicable. Many of the assessments in this study (date of signed informed consent, activity data, KCCQ data) will be collected directly from the participant and therefore the data received is from a direct source.

The author of an entry in the source documents should be identifiable.

Specific details required as source data for the study and source data collection methods will be reviewed with the virtual principal investigator or designee before the study.

Inclusion and exclusion criteria not requiring documented medical history must be verified at a minimum by participant interview or other protocol required assessment (eg, baseline KCCQ) and documented in the source documents.

RECORD RETENTION

In compliance with the ICH/GCP guidelines, the virtual principal investigator/call center will maintain all source documents that support the data collected from each participant, as well as all study documents as specified in ICH/GCP Section 8, Essential Documents for the Conduct of a Clinical Trial, and all study documents as specified by the applicable regulatory requirement(s). The virtual principal investigator/call center will take measures to prevent accidental or premature destruction of these documents.

Essential documents must be retained until at least 2 years after the last approval of a marketing application in an ICH region and until there are no pending or contemplated marketing applications in an ICH region or until at least 2 years have elapsed since the formal discontinuation of clinical development of the investigational product. These documents will be retained for a longer period if required by the applicable regulatory requirements or by an agreement with the sponsor. It is the responsibility of the sponsor to inform the virtual principal investigator/call center as to when these documents no longer need to be retained.

If the virtual principal investigator retires, relocates, or for other reasons withdraws from the responsibility of keeping the study records, custody must be transferred to a person who will accept the responsibility. The sponsor must be notified in writing of the name and address of the new custodian. Under no circumstance shall the virtual principal investigator relocate or dispose of any study documents before having obtained written approval from the sponsor.

If it becomes necessary for the sponsor or the appropriate regulatory authority to review any documentation relating to this study, the virtual principal investigator/call center must permit access to such reports.

STUDY AND SITE CLOSURE

Study Termination

The sponsor reserves the right to terminate the study at any time for any reason at the sole discretion of the sponsor.

10.3 Appendix 3: Kansas City Cardiomyopathy Questionnaire (KCCQ)

Appendix 3 provides a representative example of the scale questions that will be used in this study.

The KC Cardiomyopathy Questionnaire

The following questions refer to your **heart failure** and how it may affect your life. Please read and complete the following questions. There are no right or wrong answers. Please mark the answer that best applies to you.

1. **Heart failure** affects different people in different ways. Some feel shortness of breath while others feel fatigue. Please indicate how much you are limited by **heart failure** (shortness of breath or fatigue) in your ability to do the following activities over the past 2 weeks.

Place an **X** in one box on each line

Activity	Extremely Limited	Quite a bit Limited	Moderately Limited	Slightly Limited	Not at all Limited	Limited for other reasons or did not do the activity
Dressing yourself	❑	❑	❑	❑	❑	❑
Showering/Bathing	❑	❑	❑	❑	❑	❑
Walking 1 block on level ground	❑	❑	❑	❑	❑	❑
Doing yardwork, housework or carrying groceries	❑	❑	❑	❑	❑	❑
Climbing a flight of stairs without stopping	❑	❑	❑	❑	❑	❑
Hurrying or jogging (as if to catch a bus)	❑	❑	❑	❑	❑	❑

2. Compared with 2 weeks ago, have your symptoms of **heart failure** (shortness of breath, fatigue, or ankle swelling) changed?

My symptoms of **heart failure** have become…

Much worse	Slightly worse	Not changed	Slightly better	Much better	I've had no symptoms over the last 2 weeks
❑	❑	❑	❑	❑	❑

Copyright ©1992 –2005 John Spertus, MD, MPH Original US English

3. Over the past 2 weeks, how many times did you have **swelling** in your feet, ankles or legs when you woke up in the morning?

Every morning	3 or more times a week, but not every day	1-2 times a week	Less than once a week	Never over the past 2 weeks
❑	❑	❑	❑	❑

4. Over the past 2 weeks, how much has **swelling** in your feet, ankles or legs bothered you?

It has been ...

**Extremely** bothersome	**Quite a bit** bothersome	**Moderately** bothersome	**Slightly** bothersome	**Not at all** bothersome	I've had **no swelling**
❑	❑	❑	❑	❑	❑

5. Over the past 2 weeks, on average, how many times has **fatigue** limited your ability to do what you want?

All of the time	Several times per day	At least once a day	3 or more times per week but not every day	1-2 times per week	Less than once a week	Never over the past 2 weeks
❑	❑	❑	❑	❑	❑	❑

6. Over the past 2 weeks, how much has your **fatigue** bothered you?

It has been ...

**Extremely** bothersome	**Quite a bit** bothersome	**Moderately** bothersome	**Slightly** bothersome	**Not at all** bothersome	I've had **no fatigue**
❑	❑	❑	❑	❑	❑

7. Over the past 2 weeks, on average, how many times has **shortness of breath** limited your ability to do what you wanted?

All of the time	Several times per day	At least once a day	3 or more times per week but not every day	1-2 times per week	Less than once a week	Never over the past 2 weeks
❑	❑	❑	❑	❑	❑	❑

Copyright ©1992 –2005 John Spertus, MD, MPH Original US English

8. Over the past 2 weeks, how much has your **shortness of breath** bothered you?

It has been ...

**Extremely** bothersome	**Quite a bit** bothersome	**Moderately** bothersome	**Slightly** bothersome	**Not at all** bothersome	I've had **no shortness of breath**
❑	❑	❑	❑	❑	❑

9. Over the past 2 weeks, on average, how many times have you been forced to sleep sitting up in a chair or with at least 3 pillows to prop you up because of **shortness of breath**?

Every night	3 or more times a week, but not every day	1-2 times a week	Less than once a week	Never over the past 2 weeks
❑	❑	❑	❑	❑

10. **Heart failure** symptoms can worsen for a number of reasons. How sure are you that you know what to do, or whom to call, if your **heart failure** gets worse?

**Not at all** sure	**Not very** sure	**Somewhat** sure	**Mostly** sure	**Completely** sure
❑	❑	❑	❑	❑

11. How well do you understand what things you are able to do to keep your **heart failure** symptoms from getting worse? (for example, weighing yourself, eating a low salt diet etc.)

Do not understand at all	Do not understand very well	Somewhat understand	Mostly understand	Completely understand
❑	❑	❑	❑	❑

12. Over the past 2 weeks, how much has your **heart failure** limited your enjoyment of life?

It has **extremely** limited my enjoyment of life	It has limited my enjoyment of life **quite a bit**	It has **moderately** limited my enjoyment of life	It has **slightly** limited my enjoyment of life	It has **not limited** my enjoyment of life at all
❑	❑	❑	❑	❑

13. If you had to spend the rest of your life with your **heart failure** the way it is right now, how would you feel about this?

Not at all satisfied	Mostly dissatisfied	Somewhat satisfied	Mostly satisfied	Completely satisfied
❑	❑	❑	❑	❑

Copyright ©1992 –2005 John Spertus, MD, MPH Original US English

14. Over the past 2 weeks, how often have you felt discouraged or down in the dumps because of your **heart failure**?

I felt that way **all of the time**	I felt that way **most of the time**	I **occasionally** felt that way	I **rarely** felt that way	I **never** felt that way
❑	❑	❑	❑	❑

15. How much does your **heart failure** affect your lifestyle? Please indicate how your **heart failure** may have limited your participation in the following activities over the past 2 weeks.

Please place an **X** in one box on each line

**Activity**	**Severely** limited	Limited **quite a bit**	**Moderately** limited	**Slightly** limited	**Did not** limit at all	Does not apply or did not do for other reasons
Hobbies, recreational activities	❑	❑	❑	❑	❑	❑
Working or doing household chores	❑	❑	❑	❑	❑	❑
Visiting family or friends out of your home	❑	❑	❑	❑	❑	❑
Intimate relationships with loved ones	❑	❑	❑	❑	❑	❑

Copyright ©1992 –2005 John Spertus, MD, MPH
Original US English

10.4 Appendix 4: Patient Global Impression of Change (PGIC)

Appendix 4 provides a representative example of the scale questions that will be used in this study.

Since the start of the treatment you’ve received in this study, your heart failure symptoms are: (select one response)

1. Very much improved
2. Somewhat improved
3. A little improved
4. No change
5. A little worse
6. Somewhat worse
7. Very much worse

10.5 Appendix 5: Patient Global Impression of Severity (PGI-S)

Appendix 5 provides a representative example of the scale question that will be used in this study.

Considering all aspects of your heart failure symptoms right now, would you say your heart failure symptoms are: (select one response)

1. None
2. Mild
3. Moderate
4. Severe
5. Very Severe

10.6 Appendix 6: Participant Satisfaction Survey

Appendix 6 provides a representative example of the questions that will be provided to the participant.

For each of the statements below, please indicate your level of agreement:	Strongly Disagree	Disagree	Neutral	Agree	Strongly Agree
Trying something new to possibly treat my heart failure was important to me.					
The virtual study was easy for me to sign up for and get started.					
The Fitbit was easy to set up and use.					
The study app was easy to set up on my smartphone.					
The questionnaires were easy to complete on my smartphone.					
The reminders to complete the questionnaires were helpful.					
The study Fitbit provided helpful information to me.					
It was easy to reach the study team when I had questions or concerns.					
I would recommend participating in a study like this to my friends and family.					

10.7 Appendix 7: Adverse Events: Definitions and Procedures for Recording, Evaluating, Follow-up, and Reporting

Adverse Event

An adverse event is any untoward medical occurrence in a clinical study participant administered a medicinal (investigational or non-investigational) product. An adverse event does not necessarily have a causal relationship with the intervention. An adverse event can therefore be any unfavorable and unintended sign (including an abnormal finding), symptom, or disease temporally associated with the use of a medicinal (investigational or non-investigational) product, whether or not related to that medicinal (investigational or non-investigational) product. (Definition per International Conference on Harmonisation [ICH]).

This includes any occurrence that is new in onset or aggravated in severity or frequency from the baseline condition, or abnormal results of diagnostic procedures, including laboratory test abnormalities.

Note: The sponsor collects adverse events starting with the signing of the ICF (refer to All Adverse Events under Section 8.3.1, Time Period and Frequency for Collecting Adverse Events and Serious Adverse Events Information, for time of last adverse event recording).

Serious Adverse Event

A serious adverse event based on ICH and EU Guidelines on Pharmacovigilance for Medicinal Products for Human Use is any untoward medical occurrence that at any dose:

- results in death
- is life-threatening
  (The participant was at risk of death at the time of the event. It does not refer to an event that hypothetically might have caused death if it were more severe.)
- requires inpatient hospitalization or prolongation of existing hospitalization
- results in persistent or significant disability/incapacity
- is a congenital anomaly/birth defect
- is a suspected transmission of any infectious agent via a medicinal product.

*Medical and scientific judgment should be exercised in deciding whether expedited reporting is also appropriate in other situations, such as important medical events that may not be immediately life-threatening or result in death or hospitalization but may jeopardize the participant or may require intervention to prevent one of the other outcomes listed in the definition above. These should usually be considered serious.

If a serious and unexpected adverse event occurs for which there is evidence suggesting a causal relationship between the study intervention and the event (eg, death from anaphylaxis), the event must be reported as a serious and unexpected suspected adverse reaction even if it is a component of the study endpoint (eg, all-cause mortality).

Unlisted (Unexpected) Adverse Event/Reference Safety Information

An adverse event is considered unlisted if the nature or severity is not consistent with the applicable product reference safety information. For JNJ-28431754 (canagliflozin), the expectedness of an adverse event will be determined by whether or not it is listed in the IB.

ATTRIBUTION DEFINITIONS

Assessment of Causality

The causal relationship to study treatment is determined by the virtual principal investigator. The following selection should be used to assess all adverse events (AE).

Related

There is a reasonable causal relationship between study treatment administration and the AE.

Not Related

There is not a reasonable causal relationship between study treatment administration and the AE.

The term "reasonable causal relationship" means there is evidence to support a causal relationship.

SEVERITY CRITERIA

An assessment of severity grade will be made using the following general categorical descriptors:

Mild: Awareness of symptoms that are easily tolerated, causing minimal discomfort and not interfering with everyday activities.

Moderate: Sufficient discomfort is present to cause interference with normal activity.

Severe: Extreme distress, causing significant impairment of functioning or incapacitation. Prevents normal everyday activities.

The virtual principal investigator should use clinical judgment in assessing the severity of events not directly experienced by the participant (eg, laboratory abnormalities).

SPECIAL REPORTING SITUATIONS

Safety events of interest on a sponsor study intervention in an interventional study that may require expedited reporting or safety evaluation include, but are not limited to:

- overdose of a sponsor study intervention
- suspected abuse/misuse of a sponsor study intervention
- accidental or occupational exposure to a sponsor study intervention
- any failure of expected pharmacologic action (ie, lack of effect) of a sponsor study intervention

- unexpected therapeutic or clinical benefit from use of a sponsor study intervention
- medication error, intercepted medication error, or potential medication error involving a Johnson & Johnson medicinal product (with or without patient exposure to the Johnson & Johnson medicinal product, eg, product name confusion, product label confusion, intercepted prescribing or dispensing errors)
- exposure to a sponsor study intervention from breastfeeding.

Special reporting situations should be reported. Any special reporting situation that meets the criteria of a serious adverse event should also be reported.

PROCEDURES

All Adverse Events

Adverse events, regardless of seriousness, severity, or presumed relationship to study intervention, must be recorded using medical terminology on the solicited AE form. Whenever possible, diagnoses should be given when signs and symptoms are due to a common etiology (eg, cough, runny nose, sneezing, sore throat, and head congestion should be reported as "upper respiratory infection").

For all studies with an outpatient phase, including open-label studies, the participant must be provided with a "wallet (study) card" and instructed to carry this card with them for the duration of the study indicating the following:

- study number
- statement, in the local language(s), that the participant is participating in a clinical study
- call center name and 24-hour contact telephone number
- site number
- participant number
- any other information that is required to do an emergency breaking of the blind.

Serious Adverse Events

All serious adverse events that have not resolved by the end of the study, or that have not resolved upon discontinuation of the participant's participation in the study, must be followed until any of the following occurs:

- the event resolves
- the event stabilizes
- the event returns to baseline, if a baseline value/status is available
- the event can be attributed to agents other than the study intervention or to factors unrelated to study conduct

- it becomes unlikely that any additional information can be obtained (participant or health care practitioner refusal to provide additional information, lost to follow-up after demonstration of due diligence with follow-up efforts)
- suspected transmission of an infectious agent by a medicinal product will be reported as a serious adverse event. Any event requiring hospitalization (or prolongation of hospitalization) that occurs during the course of a participant's participation in a study must be reported as a serious adverse event, except hospitalizations for the following:
- hospitalizations not intended to treat an acute illness or adverse event (eg, social reasons such as pending placement in long-term care facility)
- surgery or procedure planned before entry into the study (must be documented). Note: hospitalizations that were planned before the signing of the ICF, and where the underlying condition for which the hospitalization was planned has not worsened, will not be considered serious adverse events. Any adverse event that results in a prolongation of the originally planned hospitalization is to be reported as a new serious adverse event.
- for convenience the virtual principal investigator may choose to hospitalize the participant for the duration of the intervention period.

The cause of death of a participant in a study[within 24 hours of the last dose of study intervention, whether or not the event is expected or associated with the study intervention, is considered a serious adverse event.

CONTACTING SPONSOR REGARDING SAFETY

The names (and corresponding telephone numbers) of the individuals who should be contacted regarding safety issues or questions regarding the study are listed in the Contact Information page(s), which will be provided as a separate document.

PRODUCT QUALITY COMPLAINT HANDLING

A product quality complaint (PQC) is defined as any suspicion of a product defect related to manufacturing, labeling, or packaging, ie, any dissatisfaction relative to the identity, quality, durability, or reliability of a product, including its labeling or package integrity. A PQC may have an impact on the safety and efficacy of the product. Timely, accurate, and complete reporting and analysis of PQC information from studies are crucial for the protection of participants, investigators, and the sponsor, and are mandated by regulatory agencies worldwide. The sponsor has established procedures in conformity with regulatory requirements worldwide to ensure appropriate reporting of PQC information; all studies conducted by the sponsor or its affiliates will be conducted in accordance with those procedures.

Procedures

All initial PQCs must be reported by the call center to the sponsor within 24 hours after being made aware of the event.

If the defect is combined with a serious adverse event, the study-site personnel must report the PQC to the sponsor according to the serious adverse event reporting timelines (refer to Section 8.3.1, Time Period and Frequency for Collecting Adverse Event and Serious Adverse

Event Information). A sample of the suspected product should be maintained for further investigation if requested by the sponsor.

Contacting Sponsor Regarding Product Quality

The names (and corresponding telephone numbers) of the individuals who should be contacted regarding product quality issues are listed in the Contact Information page(s), which will be provided as a separate document.

10.8 Appendix 8: Protocol Amendment History

This is an original protocol.

11. REFERENCES

1. Butler J (2017), Hamo CE, Filippatos G, et al. The potential role and rationale for treatment of heart failure with sodium-glucose co-transporter 2 inhibitors. Eur J Heart Fail. 2017.

2. Dreyer RP (2016), Jones PG, Kutty S, Spertus JA. Quantifying clinical change: discrepancies between patients' and providers' perspectives. Qual Life Res. 2016 Sep;25(9): 2213-2220.

3. European Medicines Agency (EMEA) (2012). Guideline on clinical investigation of medicinal products in the treatment or prevention of diabetes mellitus. 14 May 2012; CPMP/EWP/1080/00 Rev. 1. Committee for Medicinal Products for Human Use (CHMP).

4. Food and Drug Administration (FDA) (2008). Guidance for industry: Diabetes Mellitus: Developing drugs and therapeutic biologics for treatment and prevention. U.S. Department of Health and Human Services, Food and Drug Administration, Center for Drug Evaluation and Research (CDER), February 2008, Clinical/Medical.

5. Food and Drug Administration (FDA) (2019). Treatment for heart failure: Endpoints for drug development, Guidance for Industry. Draft guidance un 2019; US Department of Health and Human Service, Food and Drug Administration.

6. Gail M (1985), Simon R. Testing for qualitative interactions between treatment effects and patient subsets. Biometrics. 1985 Jun;41(2):361-372.

7. Green CP (2000), Porter CB, Bresnahan DR, et al. Development and evaluation of the Kansas City Cardiomyopathy Questionnaire: A new health status measure for heart failure. J Am Coll Cardiol 2000;35:1245-1255.

8. Investigator's Brochure: JNJ-28431754 (Canagliflozin) Edition 15. Janssen Research and Development, LLC (9 Nov 2017).

9. Joseph SM (2013), Novak E, Arnold SV, et al. Comparable performance of the Kansas City Cardiomyopathy Questionnaire in patients with heart failure with preserved and reduced ejection fraction Circ Heart Fail. 2013 Nov;6(6):1139-1146.

10. Kelkar AA (2016), Spertus J, Pang P, et al. Utility of patient-reported outcome instruments in heart failure. JACC Heart Failure 2016;4(3)165-175.

11. Mahaffey KW (2019), Jardine MJ, Bompoint S, et al. Canagliflozin and cardiovascular and renal outcomes in type 2 diabetes and chronic kidney disease in primary and secondary cardiovascular prevention groups: results from the randomized CREDENCE trial. Circulation. 2019 Aug 27;140(9):739-750.

12. Mahaffey KW (2018), Neal B, Perkovic B, et al. Canagliflozin for primary and secondary prevention of cardiovascular events: results from the CANVAS program (Canagliflozin Cardiovascular Assessment Study). Circulation. 2018 Jan 23;137(4):323-334.

13. McMurray JJV (2019), Solomon SD, Inzucchi SE, et al; DAPA-HF Trial Committees and Investigators. Dapagliflozin in patients with heart failure and reduced ejection fraction. N Engl J Med. 2019 Sep 19.

14. Medical Device Development Tools Program (2016). Medical Development Tool Qualification Decision Summary for Kansas City Cardiomyopathy Questionnaire (KCCQ). Available from: https://www.fda.gov/downloads/MedicalDevices/ScienceandResearch/MedicalDeviceDevelopmentToolsMDDT/UCM581761.pdf. Accessed on 13 November 2017.

15. Monami M (2014), Dicembrini I, Mannucci E. Dipeptidyl peptidase-4 inhibitors and heart failure: a meta-analysis of randomized clinical trials. Nutr Metab Cardiovasc Dis. 2014;24(7):689-697.

16. Mosenzon O (2019), Wiviott SD, Cahn A, et. al. Effects of dapagliflozin on development and progression of kidney disease in patients with type 2 diabetes: an analysis from the DECLARE-TIMI 58 randomised trial. Lancet Diabetes Endocrinol. 2019 Aug;7(8):606-617.

17. Nassif ME (2019), Kosiborod M. Effects of sodium-sodglucose cotransporter type 2 inhibitors in heart failure. Diabetes Obes Metab. 2019;21(Suppl. 2):19-23].

18. Nassif ME (2019), Windsor SL, Tang F, et al. Dapagliflozin Effects on Biomarkers, Symptoms, and Functional Status in Patients With Heart Failure With Reduced Ejection Fraction: The DEFINE-HF Trial. Circulation. 2019 Oct 29;140(18):1463-1476.

19. National Kidney Foundation (2012). KDOQI Clinical Practice Guideline for Diabetes and CKD: 2012 Update. Am J Kidney DIS. 2012;60(5):850-886.

20. Nichols GA (2001), Hillier TA, Erbey JR, Brown JB. Congestive heart failure in type 2 diabetes: prevalence, incidence, and risk factors. Diabetes Care. 2001;24(9):1614-1619.

21. Nichols GA (2004), Gullion CM, Koro CE, Ephross SA, Brown JB. The incidence of congestive heart failure in type 2 diabetes. Diabetes Care. 2004;27(8):1879-1884.

22. Packer M (2017), Anker SD, Butler J, Filippatos G, Zannad F. Effects of sodium-glucose cotransporter 2 inhibitors for the treatment of patients with heart failure: proposal of a novel mechanism of action. JAMA Cardiol. 2017;2(9):1025-1029.

23. Radholm K (2018), Figtree G, Perkovic V, et al. Canagliflozin and Heart Failure in Type 2 Diabetes Mellitus. Results From the CANVAS Program. Circ. 2018;138:458–468.

24. Ryan PB (2018), Buse JB, Schuemie MJ, et al. Comparative effectiveness of canagliflozin, SGLT2 inhibitors and non-SGLT2 inhibitors on the risk of hospitalization for heart failure and amputation in patients with type 2 diabetes mellitus: A real-world meta-analysis of 4 observational databases (OBSERVE-4D). Diabetes Obes Metab. 2018;20(11):2585–2597.

25. Sezai A (2019), Sekino H, Unosawa S, et al. Canagliflozin for Japanese patients with chronic heart failure and type II diabetes. Cardiovasc Diabetol. (2019) Jun 5;18(1):76.

26. Spertus J (2005), Peterson E, Conard MW, et al. Monitoring clinical changes in patients with heart failure: a comparison of methods. Am Heart J 2005;150(4):707-715.

27. Zinman B (2015), Wanner C, Lachin JM, et. al. Empagliflozin, cardiovascular outcomes and mortality in type 2 diabetes. N Engl J Med 2015;373:2117-2128.

INVESTIGATOR AGREEMENT

JNJ-28431754 (canagliflozin) (INVOKANA)

Clinical Protocol 28431754HFA3002

INVESTIGATOR AGREEMENT

I have read this protocol and agree that it contains all necessary details for carrying out this study. I will conduct the study as outlined herein and will complete the study within the time designated.

I will provide copies of the protocol and all pertinent information to all individuals responsible to me who assist in the conduct of this study. I will discuss this material with them to ensure that they are fully informed regarding the study intervention, the conduct of the study, and the obligations of confidentiality.

Coordinating Investigator (where required):

Name (typed or printed): ____________________

Institution and Address: ____________________

____

____

____

Signature: ____________________ Date: ____________________

(Day Month Year)

Principal (Site) Investigator:

Name (typed or printed): ____________________

Institution and Address: ____________________

____

____

____

Telephone Number: ____________________

Signature: ____________________ Date: ____________________

(Day Month Year)

Sponsor's Responsible Medical Officer:

Name (typed or printed): Paul Burton, MD

Institution: Janssen Scientific Affairs

Signature: 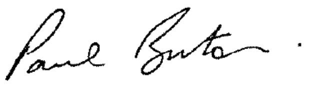 Date: 07/November/2019

(Day Month Year)

Note: If the address or telephone number of the investigator changes during the course of the study, written notification will be provided by the investigator to the sponsor, and a protocol amendment will not be required.

65

Approved, Date: 7 November 2019

Final Protocol

Janssen Research & Development*

Clinical Protocol

Protocol Title
Canagliflozin: Impact on Health Status, Quality of Life, and Functional Status in Heart Failure

CHIEF-HF

Protocol 28431754HFA3002; Phase 3B
AMENDMENT 3

JNJ-28431754 (canagliflozin) (INVOKANA)

*Janssen Research & Development is a global organization that operates through different legal entities in various countries. Therefore, the legal entity acting as the sponsor for Janssen Research & Development studies may vary, such as, but not limited to Janssen Biotech, Inc.; Janssen Products, LP; Janssen Biologics, BV; Janssen-Cilag International NV; Janssen, Inc; Janssen Pharmaceutica NV; Janssen Sciences Ireland UC; or Janssen Research & Development, LLC. The term "sponsor" is used throughout the protocol to represent these various legal entities; the sponsor is identified on the Contact Information page that accompanies the protocol.

US sites of this study will be conducted under US Food & Drug Administration IND regulations (21 CFR Part 312).

Status: Approved
Date: 23 February 2021
Prepared by: Janssen Scientific Affairs
EDMS number: EDMS-ERI-154820237, 6.0

GCP Compliance: This study will be conducted in compliance with Good Clinical Practice, and applicable regulatory requirements.

Confidentiality Statement
The information provided herein contains Company trade secrets, commercial or financial information that the Company customarily holds close and treats as confidential. The information is being provided under the assurance that the recipient will maintain the confidentiality of the information under applicable statutes, regulations, rules, protective orders or otherwise.

PROTOCOL AMENDMENT SUMMARY OF CHANGES TABLE

DOCUMENT HISTORY	
**Document**	**Date**
Amendment 3	23 February 2021
Amendment 2	02 June 2020
Amendment 1	07 February 2020
Original Protocol	07 November 2019

Amendment 3 (23 February 2021)

Overall Rationale for the Amendment: The overall rationale for the amendment is to clarify the adverse event (AE) reporting requirements and time frames for the study.

Section number and Name	Description of Change	Brief Rationale
Synopsis (Adverse Event Data Collection, Adverse Event Evaluations, Adverse Event Analyses);<br>3. Objectives and Endpoints (Adverse Event Data Collection);<br>8.3. Adverse Events and Serious Adverse Events and Special Situations;<br>8.3.1. Time Period and Frequency for Collecting Adverse Event and Serious Adverse Event Information;<br>9.4.3 Adverse Event Analysis	The following text was modified:<br>"Due to the virtual method of data collection for this study, there will be 2 types of adverse event data collection**,** one ~~will be done~~ by participants instructed to self-report any adverse events ~~from the study intervention~~ to a call center, and the other by an analysis of medical claims data from the participant's provider to their insurance system. **Participant-reported adverse events to the call center will be captured from the time of informed consent until 30 days after the end of treatment (ie, the last dose of study intervention) and adverse events from medical claims data from the time of informed consent to the end of study.** ~~Adverse events will be captured from the time of informed consent through the end of treatment (Month 3) plus 30 days.~~ **After the treatment period plus 30 days, serious adverse events will be identified solely through medical claims data. Adverse events that occur during the treatment period plus 30 days identified from medical claims will be aggregated by treatment assignment when unblinding is available. In addition, for this time period, self-reported adverse events from the call center will be summarized in a listing. Serious adverse event data from medical claims for the 9-month study period will be aggregated by treatment assignment.** ~~Medical claims adverse event data will be reviewed and evaluated in aggregate at the end of the 3-month, double-blind treatment period when the unblinded data are available.~~"	To clarify that<br>1. Adverse events will be collected from medical claims as well as from the call center, from the time of informed consent through the end of treatment plus 30 days.<br>2. After the treatment period plus 30 days, serious adverse events will be identified from medical claims data.<br>3. Aggregated adverse events from medical claims data will be presented for the treatment period plus 30 days. In addition, for the same period, a listing will be provided for the self-reported adverse events from the call center.<br>4. Serious adverse events from medical claims will be summarized at the end of the 9-month study period.
1.3 Schedule of Activities (SoA)	Reporting of adverse events is extended from end of treatment to end of study in SoA table.<br><br>Footnote 'e' revised to '**Participant-reported adverse events to the call center occurs** ~~Lasts~~ from the time of informed consent until 30 days after the end of treatment (ie, the last dose of	To clarify the duration of self-reported adverse events from call center and adverse events collected from medical claim data.

Section number and Name	Description of Change	Brief Rationale
	study intervention). **Adverse events from medical claims data occurs from the time of informed consent to the end of study.**'	
10.1 Appendix 1: Abbreviations	Abbreviation list updated for an 'SAE'.	Minor errors were noted.
Throughout the protocol	Minor formatting and consistency changes were made.	

TABLE OF CONTENTS

PROTOCOL AMENDMENT SUMMARY OF CHANGES TABLE **2**

TABLE OF CONTENTS **4**

LIST OF IN-TEXT TABLES AND FIGURES **5**

1. PROTOCOL SUMMARY **6**
1.1. Synopsis 6
1.2. Schema 11
1.3. Schedule of Activities (SoA) 12

2. INTRODUCTION **14**
2.1. Background and Study Rationale 18
2.2. Benefit/Risk Assessment 19

3. OBJECTIVES AND ENDPOINTS **20**

4. STUDY DESIGN **22**
4.1. Overall Design 22
4.2. Scientific Rationale for Study Design 24
4.2.1. Study-Specific Ethical Design Considerations 26
4.3. Justification for Dose 26
4.4. End of Study Definition 26

5. STUDY POPULATION **27**
5.1. Inclusion Criteria 27
5.2. Exclusion Criteria 28
5.3. Prohibitions and Restrictions 29
5.4. Screen Failures 29

6. STUDY INTERVENTION **30**
6.1. Study Interventions Administered 30
6.2. Preparation/Handling/Storage/Accountability 32
6.3. Measures to Minimize Bias: Randomization and Blinding 32
6.4. Study Intervention Compliance 33
6.5. Prohibited Medications 33

7. DISCONTINUATION OF STUDY INTERVENTION AND PARTICIPANT DISCONTINUATION/WITHDRAWAL **33**
7.1. Discontinuation of Study Intervention 33
7.1.1. Temporary Discontinuation of Study Intervention 34
7.2. Participant Discontinuation/Withdrawal From the Study 34

8. STUDY ASSESSMENTS AND PROCEDURES **35**
8.1. Effectiveness Assessments 35
8.1.1. KCCQ Scores 35
8.1.2. Actigraphy Measurements 36
8.2. Exploratory Assessments 36
8.2.1. Patient Global Impression of Change (PGIC) Scores 36
8.2.2. Patient Global Impression of Severity (PGI-S) Scores 36
8.2.3. Participant Satisfaction Survey 37
8.2.4. Healthcare Resource Utilization and Health Economics 37
8.3. Adverse Events and Serious Adverse Events and Special Situations 37
8.3.1. Time Period and Frequency for Collecting Adverse Event and Serious Adverse Event Information 38
8.3.2. Regulatory Reporting Requirements for Serious Adverse Events 38
8.3.3. Pregnancy 38

8.3.4. Adverse Events of Interest 38

9. STATISTICAL CONSIDERATIONS 39
9.1. Statistical Hypotheses 39
9.2. Sample Size Determination 39
9.3. Populations for Analyses 40
9.4. Statistical Analyses 40
9.4.1. Handling of Missing Data 40
9.4.2. Effectiveness Analyses 40
9.4.2.1. Primary and Secondary Effectiveness Analyses 40
9.4.2.2. Exploratory Analyses 41
9.4.3. Adverse Events Analysis 41
9.4.4. Healthcare Resource Utilization and Health Economics Analyses 42
9.5. Interim Analysis 42

10. SUPPORTING DOCUMENTATION AND OPERATIONAL CONSIDERATIONS 43
10.1 Appendix 1: Abbreviations 43
10.2 Appendix 2: Regulatory, Ethical, and Study Oversight Considerations 45
10.3 Appendix 3: Kansas City Cardiomyopathy Questionnaire (KCCQ) 53
10.4 Appendix 4: Patient Global Impression of Change (PGIC) 57
10.5 Appendix 5: Patient Global Impression of Severity (PGI-S) 58
10.6 Appendix 6: Participant Satisfaction Survey 59
10.7 Appendix 7: Adverse Events: Definitions and Procedures for Recording, Evaluating, Follow-up, and Reporting 60
10.8 Appendix 8: Protocol Amendment History 65

11. REFERENCES 72

INVESTIGATOR AGREEMENT 74

LIST OF IN-TEXT TABLES AND FIGURES

TABLES

Table 1: Effects of Sodium-glucose Cotransports-2 Inhibitors (SGLT-2is) on Heart Failure Outcomes 19
Table 2: Description of Study Interventions 31
Table 3: Analysis Populations 40

FIGURES

Figure 1: Schematic Overview of the Study 11

1. PROTOCOL SUMMARY

1.1. Synopsis

Canagliflozin: Impact on Health Status, Quality of Life, and Functional Status in Heart Failure

Canagliflozin (JNJ-28431754) is an inhibitor of sodium-glucose co-transporter 2 (SGLT2) that has been developed as an oral antihyperglycemic agent (AHA) for the treatment of patients with type 2 diabetes mellitus (T2DM). The full chemical name for canagliflozin is (1S)-1,5-anhydro-1-[3-[[5-(4-fluorophenyl)-2-thienyl]methyl]-4-methylphenyl]-D-glucitol hemihydrate.

Canagliflozin therapy has been shown to improve clinical outcomes including hospitalizations for heart failure (HHF) in patients with T2DM and HF based on results from the CANVAS program (CANVAS [DIA3008]/CANVAS-Renal [DIA4003]) and CREDENCE [DNE3001] study. An improvement in patient's symptoms of HF would be an important advance in the clinical management of HF. This randomized study is designed to assess whether canagliflozin therapy improves HF symptoms as assessed by the Total Symptom Score (TSS) of the Kansas City Cardiomyopathy Questionnaire (KCCQ) patient-reported outcome (PRO) scale in participants with HF and with or without T2DM in a real-world setting.

OBJECTIVES AND ENDPOINTS

Primary Objective

The primary objective is:

- to determine the superiority of the effectiveness of canagliflozin 100 mg daily versus placebo in participants with symptomatic HF in improving the overall KCCQ TSS.

Secondary Objectives

The first key secondary objective is:

- to determine the superiority of the effectiveness of canagliflozin 100 mg daily versus placebo in improving the total daily step count.

The second key secondary objective is:

- to determine the superiority of the effectiveness of canagliflozin 100 mg daily versus placebo in improving the KCCQ individual domain scores (physical limitation, quality of life, clinical summary, and overall).

Exploratory Objectives

The exploratory objectives are to assess the effectiveness of canagliflozin 100 mg daily in participants with symptomatic HF by:

- responses on the Patient Global Impression of Change (PGIC) and the Patient Global Impression of Severity (PGI-S)
- healthcare resource utilization (HRU) and health economics data
- associations (correlations and/or categorical association measures) between digital markers (step count and floors climbed), and clinical events such as need for outpatient intravenous therapies, emergency department (ED) visits, and hospitalizations
- daily floors climbed

- time to first hospitalization/readmission for heart failure
- time to first hospitalization/readmission for other events
- time to death
- the participant's satisfaction with their experience with the virtual design at the end of the study.

Endpoints

The primary effectiveness endpoint is the change in KCCQ TSS from baseline to Month 3.

The key secondary effectiveness endpoints are 1) change in total daily step count from baseline to Month 3, and 2) changes in KCCQ individual domain scores (physical limitation, quality of life, clinical summary, and overall) from baseline to Month 3.

The exploratory endpoints are the following:

- mean PGIC scores over time
- changes in PGI-S scores from baseline to Month 3
- change in number of daily floors climbed
- change in HRU and health economics data
- number of outpatient intravenous therapies, ED visits, and hospitalizations
- time to first hospitalization/readmission for heart failure
- time to first hospitalization/readmission for other events
- time to death

Adverse Event Data Collection

Due to the virtual method of data collection for this study, there will be 2 types of adverse event data collection, one by participants instructed to self-report any adverse events to a call center, and the other by an analysis of medical claims data from the participant's provider to their insurance system. Participant-reported adverse events to the call center will be captured from the time of informed consent until 30 days after the end of treatment (ie, the last dose of study intervention) and adverse events from medical claims data from the time of informed consent to the end of study. After the treatment period plus 30 days, serious adverse events will be identified solely through medical claims data. Adverse events that occur during the treatment period plus 30 days identified from medical claims will be aggregated by treatment assignment when unblinding is available. In addition, for this time period, self-reported adverse events from the call center will be summarized in a listing. Serious adverse event data from medical claims for the 9-month study period will be aggregated by treatment assignment.

Hypotheses

The primary hypothesis is that canagliflozin is superior to placebo as assessed by improvement in the TSS on the KCCQ scale during the 3-month, double-blind treatment period.

OVERALL DESIGN

This is a randomized, double-blind, placebo-controlled, decentralized, virtual, interventional, superiority study conducted in the United States (US) in participants with symptomatic HF (stratified by heart failure with reduced ejection fraction [HFrEF] and heart failure with preserved ejection fraction [HFpEF]) to examine the improvement in the KCCQ TSS after 3 months of treatment with canagliflozin 100 mg or

placebo. The study will continue for an additional 6 months without any study intervention to collect participant follow-up in a real-world setting.

The study will enroll participants from large, integrated, health networks and large physician practices in the US with the participants conducting the majority of their study-related activities through an app on a smartphone and actigraphy data from a Fitbit device. There will be no in-person clinic visits required by the study.

The study consists of a screening period and a 3-month, double-blind treatment period. Approximately 1,900 participants will be randomized into the study in a 1:1 ratio to canagliflozin 100 mg daily or placebo groups (950 in each study intervention group). After Month 3 (the end of the double-blind treatment period), the study will continue for an additional 6 months when study intervention will no longer be provided. Data collection from medical claims, smartphone, Fitbit device, and selected PROs will continue until Month 9 for the purpose of secondary and further exploratory endpoints analyses. In addition, the KCCQ, PGIC, and PGI-S data will be collected at 6 and 9 months. An optional participant satisfaction survey about the virtual nature of the study will be provided to the participant at end of the study.

Participants will be of any gender, 18 years of age or older, and have clinically stable, symptomatic HF with or without T2DM with a KCCQ baseline overall summary score of ≤80 prior to randomization.

Study assessments include the KCCQ, Fitbit device data (step count and floors climbed), PGIC, PGI-S, participant satisfaction survey, medical and pharmacy claims data (including up to 36 months history for eligibility confirmation), HRU and health economics (including hospitalization/readmission due to HF or other reason, ED visits, the duration of stay at each hospitalization/readmission), review of medical claims for prohibited medications and new diagnoses that may require discontinuation, dates of deaths, and adverse events.

NUMBER OF PARTICIPANTS

Approximately 1,900 participants will be randomized in the study in a 1:1 ratio to canagliflozin 100 mg daily or placebo. Randomization of participants into the study will be stratified by the type of ejection fraction (ie, HFrEF or HFpEF) at study entry as recorded in the participant's electronic health record (EHR).

INTERVENTION GROUPS AND DURATION

The total duration of study participation for each participant is approximately 9 months. Participants will be randomly assigned to receive canagliflozin 100 mg daily immediate-release over-encapsulated tablets (capsules) taken orally or placebo capsules for the duration of the 3-month, double-blind treatment period, followed by a 6-month, no treatment period during which data will continue to be collected.

Description of Interventions

The study intervention, JNJ-28431754 (canagliflozin), will be provided as immediate-release, over-encapsulated tablets (as a capsule) or a placebo capsule taken orally once daily for the duration of the 3-month, double-blind treatment period.

Participant Unblinding

After each randomized participant completes the 3-month, double-blind treatment period of the study, they will be provided with their treatment allocation, whether they had been randomized to canagliflozin or placebo.

EFFECTIVENESS EVALUATIONS

Effectiveness evaluations include the KCCQ and daily step count.

SECONDARY AND EXPLORATORY EVALUATIONS

Secondary and exploratory evaluations include KCCQ individual domains, PGIC, PGI-S, participant satisfaction survey, the HRU and health economics data collected in this study, time to death, and associations (correlations and/or categorical association measures) between digital markers (step count and floors climbed) and clinical events such as need for outpatient intravenous therapies, ED visits, and hospitalizations.

ADVERSE EVENT EVALUATIONS

Adverse events will be captured through medical claims (from the time of informed consent to the end of study) and by the call center (from the time of informed consent until 30 days after the end of treatment). After the treatment period plus 30 days, serious adverse events will be identified solely through medical claims data. Adverse events that occur during the treatment period plus 30 days identified from medical claims will be aggregated by treatment assignment when unblinding is available. In addition, for this time period, self-reported adverse events from the call center will be summarized in a listing. Serious adverse event data from medical claims for the 9-month study period will be aggregated by treatment assignment.

STATISTICAL METHODS

Sample Size Determination

The primary effectiveness endpoint is the change from baseline in the KCCQ TSS at the end of the 3-month, double-blind treatment period. Although a 5-point improvement in the KCCQ TSS is generally viewed as clinically meaningful, recent studies have shown that improvements in the range of 3 to 5 points on the KCCQ TSS are equally meaningful in the overall HF-related health status measured by the KCCQ over a 12-week time period. Based on the evidence from these studies, the current study is powered to detect a mean change from baseline of 3 points in the KCCQ TSS between canagliflozin 100 mg and placebo groups at 12 weeks. Assuming a significance level of 5% and a standard deviation of 18 points for mean change in KCCQ TSS, a total of 1,900 participants will be randomized in this study providing approximately 95% power. This sample size is adequate to account for a potential 5% dropout rate.

Effectiveness Analyses

Primary and Secondary Effectiveness Analysis

The primary and key secondary effectiveness analyses will be based on the intent-to-treat (ITT) analysis population (using all randomized participants).

As the KCCQ TSS is measured repeatedly over time (ie, at baseline, Weeks 4, 6, and 12 respectively), the primary effectiveness endpoint (change in KCCQ TSS from baseline to Month 3) will be analyzed by a mixed effect model repeated measures (MMRM) method simultaneously adjusting for study intervention group, stratification factor (HFrEF versus HFpEF), time, time by study intervention group, and baseline KCCQ TSS value as covariates. An unstructured covariance structure will be assumed across study intervention groups to model the within-participant errors. The primary comparison will be based on the difference in least squares means between treatments at Month 3. The treatment difference in the least-squares means and their 2-sided 95% confidence interval (CI) will be provided. An MMRM model accounts for data missing at random.

A responder analysis will also be performed when comparing proportions of participants with a 5- (or 10-) point improvement in KCCQ TSS from baseline between the groups. This will be based on a two-sample difference in proportions test along with a 95% confidence interval for the difference in 2 proportions. Additionally, empirical cumulative distribution functions of the KCCQ TSS and other domain scores will be presented.

Subgroups defined by age, gender, race and baseline disease/demographic characteristics will be pre-specified in the Statistical Analysis Plan (SAP) for the primary effectiveness analysis. Details about the imputation of any missing data will be provided in the SAP. Each subgroup will be analyzed following a similar MMRM model used in the analysis of the primary effectiveness endpoint (change in KCCQ TSS from baseline to Month 3). The treatment difference in the least-squares means and their 2-sided 95% CI will be provided from each subgroup analysis. Quantitative and/or qualitative interactions will be identified. Qualitative interactions will be investigated further by the Gail and Simon test and interpreted accordingly.

The first key secondary endpoint of daily step count will be tested once the primary endpoint is shown to be significant at the 5% significance level using a two-sided t-test. This approach will control for the overall Type I error rate at 5%.

For the second key effectiveness endpoint, an MMRM model (like that used in the analysis of the primary endpoint) will be employed to summarize changes in KCCQ individual domain scores (physical limitation, quality of life, clinical summary, and overall) from baseline to Month 3. This hypothesis will be independently tested at 5% without any control for overall Type I error rate.

Exploratory Analyses

Exploratory analyses include the following:

- summary statistics of PGIC and PGI-S scores
- associations (correlations and/or categorical association measures) between digital markers (step count and floors climbed) and clinical events such as need for outpatient intravenous therapies, ED visits, and hospitalizations
- tabulation and comparison of total HRU and health economics data across groups
- descriptive statistics (eg, mean, median, and standard deviation) of daily floors climbed
- association between the KCCQ change from baseline to Month 3 and the PGIC at Month 3 will be examined and summarized
- time to death
- descriptive statistics of the participant's satisfaction with his/her experience with the virtual design at the end of the study.

Adverse Event Analyses

Adverse events reported to the call center will be assessed by Janssen Global Medical Safety (GMS) for causality and meeting suspected unexpected serious adverse reaction (SUSAR) reporting requirements. Self-reported adverse events will be coded according to the Medical Dictionary for Regulatory Activities (MedDRA) and will be reported by preferred term by T2DM and non-T2DM. Adverse events collected from medical claims will be categorized by diagnosis code and reported by T2DM and non-T2DM. Discontinuations will be summarized based on any available data. Self-reported adverse events from the call center will be summarized in a listing.

Healthcare Resource Utilization (HRU) and Health Economics

Healthcare Resource Utilization and health economics data will be descriptively summarized by study intervention group.

1.2. Schema

Figure 1: Schematic Overview of the Study

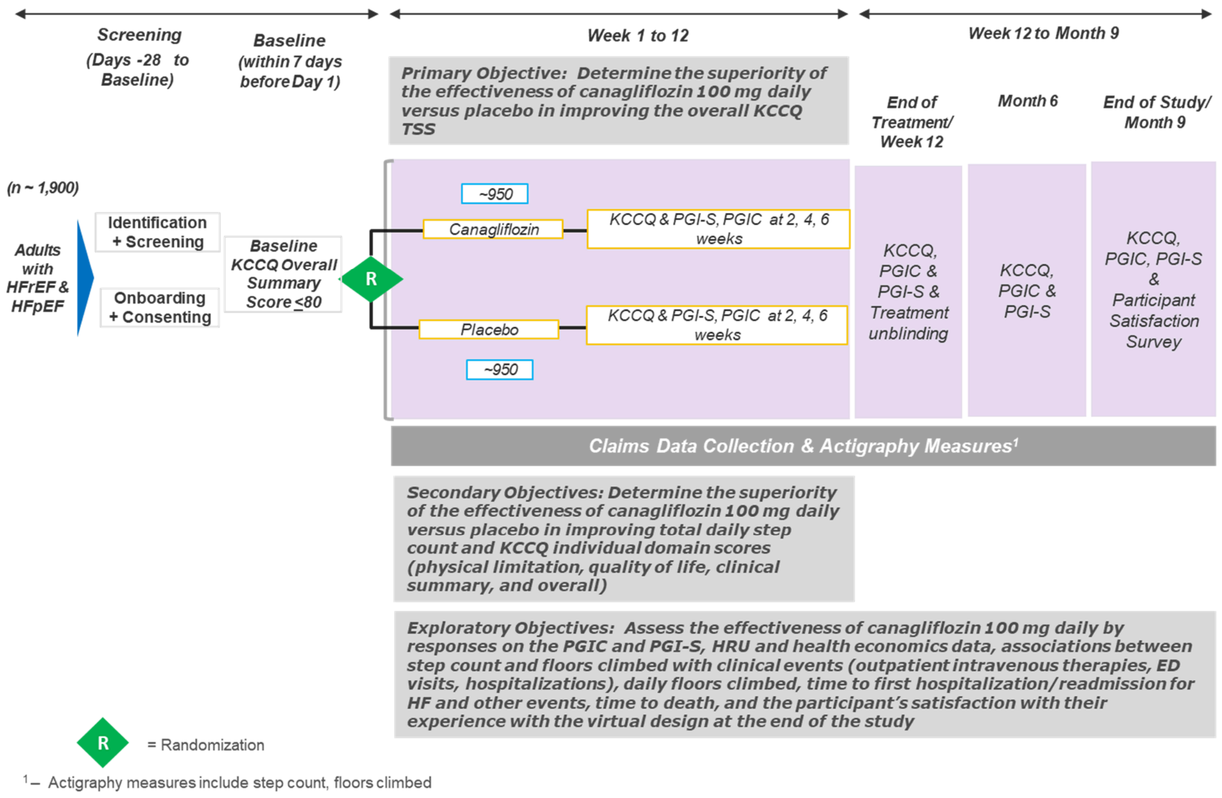

1.3. Schedule of Activities (SoA)

Period	Screening	Baseline	Double-Blind Treatment					No Treatment		Notes
Day	Within 28 days before Baseline	Within 7 days before Day 1	1 (first dose)	14+3	28±3	42±3	84±3 End of Treatment	85-251-±7	252-±7 End of Study	Day –28 to 0=Screening; Day 1 = first dose, Day 84= end of treatment; Day 252=End of Study
Week			1	2	4	6	12	13-36		Week 12 is equivalent to Day 84 of treatment
Month		0					3	6	9	Month 3 is equivalent to Day 84 of treatment
**Study Procedure**										
Screening/Administrative										
Electronic Informed consent[a]	X									
Review medical history	X									
Inclusion/exclusion criteria[b]	X	X								All inclusion/exclusion verification should be completed within the 28-day screening period and before randomization
KCCQ baseline overall summary score ≤80		X								
Study Intervention Administration										
Randomization		X								
Dispense study intervention		X								
Administer study intervention		←———	———	———	———	———	———→			
Participant-reported adherence via app - weekly		←———	———	———	———	———	———→			
Assessments										
KCCQ				X	X	X	X	X[c]	X	Kansas City Cardiomyopathy Questionnaire
PGIC				X	X	X	X	X[c]	X	Patient Global Impression of Change
PGI-S		X		X	X	X	X	X[c]	X	Patient Global Impression of Severity
Step count and floors climbed		←———	———	———	———	———	———	———	———→	
Medical claims data collection[d]		←———	———	———	———	———	———	———	———→	
Participant Satisfaction Survey (optional)									X	

Period	Screening	Baseline	Double-Blind Treatment					No Treatment		Notes
Day	Within 28 days before Baseline	Within 7 days before Day 1	1 (first dose)	14+3	28±3	42±3	84±3 End of Treatment	85-251-±7	252-±7 End of Study	Day −28 to 0=Screening; Day 1 = first dose, Day 84= end of treatment; Day 252=End of Study
Week			1	2	4	6	12	13-36		Week 12 is equivalent to Day 84 of treatment
Month		0					3	6	9	Month 3 is equivalent to Day 84 of treatment
**Health Resource Utilization and Health Economics**										
HHF for any reason and duration		←————————————————————→								Hospitalizations for heart failure
Emergency department visits		←————————————————————→								
Readmissions and duration		←————————————————————→								
**Ongoing Review of Participant Data**										
Prohibited medications and new diagnoses		←————————————————————→								
Reporting of adverse events[e]	←————————————————————→									

Footnotes:

a. Must be signed before first study-related activity.

b. Minimum criteria for the availability of documentation supporting the eligibility criteria are described in Source Documentation in Section 10.2, Appendix 2: Regulatory, Ethical, and Study Oversight Considerations.

c. To be completed at Month 6.

d. Includes up to 36 months of historical medical and pharmacy claims data for eligibility confirmation. This data collection will begin upon signing of the informed consent.

e. Participant-reported adverse events to the call center occurs from the time of informed consent until 30 days after the end of treatment (ie, the last dose of study intervention). Adverse events from medical claims data occurs from the time of informed consent to the end of study.

2. INTRODUCTION

Canagliflozin (JNJ-28431754) is an inhibitor of sodium-glucose co-transporter 2 (SGLT2) that has been developed as an oral antihyperglycemic agent (AHA) for the treatment of patients with type 2 diabetes mellitus (T2DM). The full chemical name for JNJ-28431754 is (1S)-1,5-anhydro-1-[3-[[5-(4-fluorophenyl)-2-thienyl]methyl]-4-methylphenyl]-D-glucitol hemihydrate.

The development program for canagliflozin has investigated the compound's efficacy and safety profile both as a monotherapy and in combination with other AHAs as adjunctive treatment to diet and exercise to improve glycemic control in the treatment of adult participants with T2DM. Three of the Phase 3 and Phase 4 studies evaluated canagliflozin in special populations, including older adults with T2DM and participants with T2DM who had moderate renal impairment. Recently completed cardiovascular (CV) safety studies have evaluated canagliflozin as a treatment to reduce the risk of major adverse CV events (MACE - CV death, nonfatal myocardial infarction (MI), and nonfatal stroke) in adults with T2DM who have established cardiovascular disease (CVD) or at least 2 risk factors for CVD. Canagliflozin therapy has been shown to be associated with improved clinical outcomes in patients with T2DM and heart failure (HF) based on results from the CANVAS program (CANVAS [DIA3008] /CANVAS-Renal [DIA4003]) and CREDENCE [DNE3001] studies.

Proposed mechanisms of action of sodium-glucose co-transporter 2 inhibitors (SGLT2is) and recent information from the DAPA-HF and DEFINE-HF studies[16,21] suggest that the effects of the SGLT2i class of medicines in patients with HF may extend to HF patients with or without T2DM.

This randomized study is designed to assess whether canagliflozin therapy improves HF symptoms as assessed by the Total Symptom Score (TSS) of the Kansas City Cardiomyopathy Questionnaire (KCCQ) patient-reported outcome (PRO) scale in participants with HF and with or without T2DM in a real-world setting.

CANVAS Heart Failure Data

Approximately 14.4% of all participants in the CANVAS Program had a prior history of HF. While the CANVAS Program met its primary endpoint,[15] the principal secondary endpoint (superiority of canagliflozin with respect to reducing all-cause mortality) did not meet statistical significance, hence the results of the subsequent analyses examining endpoints including hospitalization for HF (HHF) must be viewed as exploratory. The hazard ratio (HR) for HHF in the combined canagliflozin group versus placebo was 0.67 (95% CI: 0.52, 0.87), with similar effects seen in the canagliflozin 100 mg and 300 mg groups in the CANVAS study.[26] These HR results resulted in choosing the lower dose of canagliflozin 100 mg for this study.

Canagliflozin lowered the risk of HHF in both patient groups: primary (participants with only risk factors for CV disease) (HR: 0.64; 95% CI: 0.35 to 1.15) and secondary (participants with a history of CV disease) (HR: 0.68; 95% CI: 0.51 to 0.90) with no evidence of statistical heterogeneity (p=0.91).

An analysis of CANVAS[26] program demonstrated that canagliflozin decreased the risk of HHF in those with pre-existing history of HF (HR: 0.51; 95% CI: 0.33 to 0.78), and in those without a history of HF (HR: 0.79; 95% CI: 0.57 to 1.09) with no evidence of statistical heterogeneity ($p = 0.47$). This effect of reducing the risk of HHF in those with or without a history of HF was consistent irrespective of established treatments for the prevention or management of HF (ie, inhibitors of the renin-angiotensin-aldosterone system, beta-blockers, and diuretics).

Therefore, a study to demonstrate that canagliflozin improves patient symptoms of HF would be an important advance in the clinical management of HF patients and would offer an important and valuable therapeutic option.

CREDENCE Study

The CREDENCE study (NDA 204042 S-032, approved 27 September 2019, was a large, international, randomized, double-blind, event-driven study undertaken to formally test whether canagliflozin 100 mg, administered once daily, reduces the risk of kidney failure and CV events in participants with Stage 2 or 3 chronic kidney disease (eGFR ≥30 to <90 mL/min/1.73 m^2) and macroalbuminuria (urinary ACR >300 to ≤5,000 mg/g) and T2DM who were receiving Standard of Care (SoC) therapy. SoC was defined in the National Kidney Foundation Kidney Disease Outcomes Quality Initiative consensus guidelines[22] and included a maximum tolerated labeled daily dose of an angiotensin-converting enzyme inhibitor (ACEi) or angiotensin II receptor blocker (ARB). This study was conducted between 21 February 2014 and 30 October 2018.

The renal protective effect of canagliflozin relative to placebo in a population with established CKD and T2DM was measured using a composite endpoint of doubling of serum creatinine, progression to end-stage kidney disease (ESKD) (defined as dialysis, renal transplantation, or sustained eGFR <15 L/min/1.73 m^2), and renal or CV death. The study also assessed the effects of canagliflozin on several adverse CV outcomes (CV death, HHF, nonfatal MI, nonfatal stroke, and hospitalization for unstable angina), and all-cause mortality. The results demonstrated clinical benefit of the 100 mg dose of canagliflozin in reducing important cardiovascular and renal clinical endpoints in patients with T2DM and impaired renal function.[14] Specifically, HHF (which was a secondary endpoint) was significantly decreased in the canagliflozin versus placebo group (HR=0.61 [0.47-0.80]). Additionally, the rate of amputation was not shown to be statistically significantly increased with canagliflozin treatment in the CREDENCE study in the overall population (HR=1.11 [0.79-1.37]).

Recently, INVOKANA® was approved as the only diabetes medicine indicated to reduce the risk of ESKD, worsening of kidney function, cardiovascular death, and HHF in adults with type 2 diabetes and diabetic kidney disease (nephropathy) with a certain amount of protein in the urine.

Use of SGLT2is in Heart Failure With or Without T2DM

A mechanistic basis has been proposed for the SGLT2i class of medicines to exert beneficial effects in HF irrespective of the diabetic state and effects on glycemic control or osmotic diuresis.[25] Inhibition of sodium-hydrogen (Na/H) exchange in the kidney by SGLT2 inhibitors may reduce diuretic and endogenous natriuretic peptide resistance, and similar inhibition in the myocardium

may lead to a reduction in cardiac injury, hypertrophy, fibrosis, remodeling, and systolic dysfunction. Furthermore, the major pathophysiological derangements of HF and a preserved ejection fraction may be mitigated by the actions of SGLT2 inhibitors to reduce blood pressure, body weight, and fluid retention as well as to improve renal function.[25]

The use of canagliflozin in patients without T2DM has been studied in approximately 1,200 healthy subjects in multiple Phase 1 studies. A thorough QT/QTc study in healthy subjects (study DIA1010 NDA 204042 Sequence 0000 Module 5.3.4.1) demonstrated that canagliflozin at single therapeutic (300 mg) or supra-therapeutic (1,200 mg) doses does not lead to QT/QTc prolongation. At the 1,200 mg dose, canagliflozin C_{max} was approximately 1.4 times that for steady-state C_{max} at a 300 mg QD dose in T2DM subjects. This is important in light of the proarrhythmic myocardial substrate present in HF, in particular HFrEF, which is more prevalent in the non-type 2 diabetes population than in the type 2 diabetes population who experiences a higher incidence of HFpEF. For this study, the proposed dose of canagliflozin is 100 mg daily.

Additional evidence for the safety of canagliflozin in patients with HF comes from sub-group analyses of patients with T2DM in the CANVAS Program and in the CREDENCE study, in which there was no signal of increased arrhythmic death, or hyperkalemia – the latter of which is reassuring since many HF patients are on medications that are potassium-sparing, such as ACEis, ARBs, or mineralocorticoid receptor antagonists (MRAs), and diabetics are more predisposed to hyperkalemia with these agents than non-diabetics. CREDENCE in particular, which studied a population with a greater prevalence of kidney disease and a higher likelihood of hyperkalemia than the population in the CANVAS Program, demonstrated no increased incidence of hyperkalemia with canagliflozin versus placebo. In addition, in the CANVAS Program, there was no evidence of proportional differences in the risk of volume depletion, fracture, amputation, osmotic diuresis, acute kidney injury events, adverse events leading to discontinuation, and all serious adverse events (SAEs) between patients with or without HF at baseline (p interaction >0.160).[26]

A growing body of evidence is being generated for use of the SGLT2i class in HF. A randomized clinical study, DAPA-HF, in 4,774 HFrEF patients with (42%) and without (58%) T2DM demonstrated safety consistent with the known profile of dapagliflozin.[16] Ninety-four percent of patients were on ACEis or ARBs, and the mean eGFR was 66 mL/min/1.73m^2. In the study, dapagliflozin met the primary composite endpoint with a statistically significant and clinically meaningful reduction of cardiovascular death or the worsening of HF (defined as hospitalization or an urgent HF visit), compared to placebo. Based on these data, dapagliflozin was recently approved by the Food and Drug Administration (FDA) to reduce the risk of CV death and hospitalization for heart failure in adults with heart failure (NYHA class II-IV) with reduced ejection fraction with or without T2DM. This further supports more evaluations of this class of drugs in additional clinical trials in HFrEF and HFpEF, including measurement of symptomatic and functional improvement with tools such as the KCCQ.

Another recent randomized clinical study with dapagliflozin, DEFINE-HF in HFrEF, was completed in 510 patients with (62%) and without (36%) T2DM.[21] Results were consistent among

patients with or without T2DM, including safety analyses. Mean eGFR was 69 mL/min/1.73m^2. In the study, patients treated with dapagliflozin vs. placebo had clinically meaningful improvement in health status (KCCQ Overall Summary score ≥5 points).

Finally, there are multiple ongoing randomized studies with SGLT2 inhibitors in HF with or without T2DM (see below). As such, there is ample precedent to justify starting another study in HF patients irrespective of a diagnosis of T2DM.

Dapagliflozin:

- DEFINE-HF: biomarkers, symptoms, health status and quality of life in HFrEF patients (listed in clinicaltrials.gov March 2016)
- DELIVER: Outcomes study in HFpEF patients (listed in clinicaltrials.gov Aug. 2018)
- DETERMINE-REDUCED: Exercise capacity study in HFrEF patients (listed in clinicaltrials.gov March 2019)
- DETERMINE-PRESERVED: Exercise capacity study in HFpEF patients (listed in clinicaltrials.gov March 2019)

Empagliflozin:

- EMPEROR-Reduced: Outcomes study in HFrEF patients (listed in clinicaltrials.gov Feb. 2017)
- EMPEROR-Preserved: Outcomes study in HFpEF patients (listed in clinicaltrials.gov Feb. 2017)
- EMPERIAL-Reduced: Exercise capacity study in HFrEF patients (listed in clinicaltrials.gov Feb. 2018)
- EMPERIAL-Preserved: Exercise capacity study in HFpEF patients (listed in clinicaltrials.gov Feb. 2018)

Safety

The safety profile of canagliflozin is well established having been broadly evaluated for safety in several well-designed, randomized, clinical studies. The broader Phase 3/4 canagliflozin program was conducted in 22,645 patients, including 13,278 patients treated with canagliflozin and 9,367 patients treated with a comparator, in 15 double-blind, controlled, clinical studies; among them, more than 1,100 patients reported a history of HF at baseline. In addition, there is an extensive, real-world base of adverse event reporting based on approximately 1,475,000,000 person days treated worldwide. The SGLT2 inhibitor, dapagliflozin, was studied in HFrEF patients both with and without diabetes in the DAPA-HF and the DEFINE-HF studies[16,21] (those with HFrEF and type 2 diabetes). In those studies, the side effect profile was not different between those with or without T2DM or HF. Taken together, the evidence indicates that the safety profile of SGLT2 inhibitors, of which canagliflozin is a member, is well described and broadly understood in the study population included in this study.

For the most comprehensive nonclinical and clinical information regarding canagliflozin, refer to the latest version of the Investigator's Brochure[11] and Addenda for JNJ-28431754 (canagliflozin).

The term "sponsor" used throughout this document refers to the entities listed in the Contact Information page(s), which will be provided as a separate document.

The term "study intervention" refers to canagliflozin or placebo.

2.1. Background and Study Rationale

Outcomes in studies of antidiabetic agents improving HF outcomes include canagliflozin, empagliflozin, and dapagliflozin CV that have shown a strong signal for improvement in HF endpoints with SGLT2i, but further studies are needed to validate these findings. However, very recently, the FDA approved dapagliflozin to reduce the risk of HHF in adults with T2DM and established CV disease or multiple CV risk factors. Based on results of the DECLARE-TIMI 58, the possible role that SGTL2i may play in HF has been hypothesized but evaluation of the various pathways has not been performed.[30]

Heart failure contributes to one in nine deaths and is a leading cause of hospitalization in the US, yet there are limited treatment options for people living with this debilitating disease. It is highly prevalent and despite best available therapy, is associated with poor patient outcomes.[23,24] The CANVAS Program demonstrated that canagliflozin treatment in patients at cardiovascular (CV) risk was associated with improved clinical outcomes, including a reduction in the composite of CV death and HHF. A similar finding was observed with empagliflozin in the EMPA-REG cardiovascular outcomes study and dapagliflozin in DECLARE-TIMI 58.[19]

A meta-analysis of EMPA-REG OUTCOME, CANVAS PROGRAM, and DECLARE-TIMI 58 looked at a total of 20,060 T2DM patients and the CV outcomes of SGLT2i therapies. Enrolling 3,891 HF patients at baseline, this meta-analysis demonstrated similar significant reduction in HHF, regardless of the patient's baseline HF status (HR 0.68 vs HR 0.71) for those with or without HF. The benefit for these groups was affected only by renal function, with greater benefit observed in patients with lower estimated glomerular filtration rate (eGFR).[18]

In addition, the OBSERVE-4D real-world meta-analysis is the largest retrospective observational study of effects of SGLT2i in this patient population. It compared the experience of over 140,000 new canagliflozin patients to over 100,000 empagliflozin and dapagliflozin new users, as well as 460,000 new users of non-SGLT2i. Patients treated with canagliflozin showed comparable HHF reduction to other SGLT2is on the market, and greater reduced risk for HHF and below knee lower extremity amputations when compared to non-SGLT2is. This held true even when comparing patients with established CV disease to the overall study population. While limitations exist in OBSERVE-4D pertaining to the length of treatment duration with canagliflozin, this may not be entirely comparable to the randomized clinical study data discussed above. However, findings in the OBSERVE-4D meta-analysis suggest similarity to reductions observed in randomized clinical studies and provide added confidence in the results of this meta-analysis.[27]

This meta-analysis (Table 1), as well as real-world data from CVD-REAL studies and the United States (US) Department of Defense Military Health System, support the effect of an SGLT2i on reducing HF-related hospitalizations; however, they did not seek to differentiate or provide any insight on the effect of these therapies specifically on HF with reduced ejection fraction (HFrEF) and HFpEF phenotypes, defining the need for further investigation.[20]

Table 1: Effects of Sodium-glucose Cotransports-2 Inhibitors (SGLT-2is) on Heart Failure Outcomes

Reference	Comparator groups	HF at baseline	n	Study design	Follow-up (weeks)	Hazard ratio for HHF
EMPA-REG OUTCOME[34]	Empagliflozin vs placebo	10%	7020	RCT	161	0.65 (0.50-0.85
CANVAS[33]	Canagliflozin vs placebo	14%	10 142	RCT	126	0.67 (0.52-0.87)
DECLARE-TIMI-58[35]	Dapagliflozin vs placebo	10%	17 160	RCT	218	0.73 (0.61-0.88)
CVD-REAL[40]	SGLT-2is vs oGLDs	6%	309 056	Retrospective cohort	64	0.61 (0.51-0.73
EASEL Cohort[43]	SGLT-2is vs oGLDs	11%	25 258	Retrospective cohort	83	0.57 (0.45-0.73)

HHF, heart failure hospitalization; oGLDs, other glucose lowering drugs; RCT, randomized controlled trial.
Nassif ME, Kosiborod M. Effects of sodium-glucose co-transporter type 2 inhibitors in heart failure. Diabetes Obes Metab. 2019;21(Suppl. 2):19-23.

HFpEF is well recognized as the predominant type of HF in patients with T2DM and is possibly related to insulin resistance and obesity. Such predisposing factors are thought to be at the crux of ineffectiveness of traditional HF therapies in treating HFpEF patients, making the need for impactful therapies in this patient population even more urgent.[2,6] While more studies are underway, some encouraging data comes from the Canagliflozin for Japanese Patients with Chronic Heart Failure and Type II Diabetes (CANOSSA) study,[28] which assessed the effect of canagliflozin on Japanese patients with T2DM and stable HF. The study enrolled 35 patients, of which 33 had HFpEF, and focused on canagliflozin's effect of weight and adipose tissue reduction on these patients. Beyond reduction in glycated hemoglobin (HbA1c) and fat volumes, the study demonstrated reduction in oxidative stress, improved diastolic disfunction, and decrease in left ventricular mass, potentially linking canagliflozin use to an improved HFpEF condition.

In addition to showing an improvement in HHF, improvement in clinical symptoms, physical functioning and quality of life would be a highly important clinical outcome for patients with HF. It is believed that canagliflozin, through its diuretic, blood pressure, and body weight reduction effects would have a clinically meaningful impact on these outcomes in these patients and represent an advance in the clinical management of these patients. This study is designed to assess whether canagliflozin can impact these important patient-centric outcomes not only in HFrEF patients but also in HFpEF patients for which there is no approved SGLT2i for treatment.

2.2. Benefit/Risk Assessment

Before randomization and throughout the study, the participant's physician(s) will continue to manage the participant's background medications to achieve goals for controlling the participant's HF and T2DM (if present). Participants enrolled in this study will be managed based on the standards of care for HF and T2DM (if applicable) according to established local and regional guidelines. An Independent Scientific Advisory Committee comprised of experts in the management of patients with HF and sponsor representatives will be commissioned for this study. Other medications in the SGLT2i class of medicines are being studied in patients with HF with or

without T2DM as outlined in Section 2, Introduction; however, none of these agents are currently indicated for the clinical improvement in HF symptoms or for use in patients with HFpEF.

The risks of participation in the study include exposure to study intervention, with the potential for side effects. Participant safety will be assured throughout the study by providing participants and their physicians access to a call center to ask questions and to report adverse events. In addition, the adverse effect profile of canagliflozin has been well described and will be outlined in the informed consent form (ICF). In addition, participant prescribing information with a listing of potential adverse events will be sent to the participant in their welcome kit.

This study was designed based in general accordance with the FDA[7] and European Medicines Agency (EMA) guidance[6] on the development of medications and clinical investigations for the use of canagliflozin for the reduction of HF symptoms of HF and in consultation with Health Authorities. In addition, the design was also based on the FDA's draft guidance to industry on drug development in HF which supports measures of patients' symptoms and physical function as endpoints.[8] This study will be conducted under FDA Investigational New Drug (IND) regulations. More detailed information about the known and expected benefits and risks of JNJ-28431754 (canagliflozin) may be found in the Investigator's Brochure, Canagliflozin.[11]

3. OBJECTIVES AND ENDPOINTS

OBJECTIVES

Primary Objective

The primary objective is:

- to determine the superiority of the effectiveness of canagliflozin 100 mg daily versus placebo in participants with symptomatic HF in improving the overall KCCQ TSS.

Secondary Objectives

The first key secondary objective is:

- to determine the superiority of the effectiveness of canagliflozin 100 mg daily versus placebo in improving the total daily step count.

The second key secondary objective is:

- to determine the superiority of the effectiveness of canagliflozin 100 mg daily versus placebo in improving the KCCQ individual domain scores (physical limitation, quality of life, clinical summary, and overall).

Exploratory Objectives

The exploratory objectives are to assess the effectiveness of canagliflozin 100 mg daily in participants with symptomatic HF by:

- responses on the Patient Global Impression of Change (PGIC) and the Patient Global Impression of Severity (PGI-S)
- healthcare resource utilization (HRU) and health economics data
- associations (correlations and/or categorical association measures) between digital markers (step count and floors climbed), and clinical events such as need for outpatient intravenous therapies, emergency department (ED) visits, and hospitalizations
- daily floors climbed
- time to first hospitalization/readmission for heart failure
- time to first hospitalization/readmission for other events
- time to death
- the participant's satisfaction with their experience with the virtual design at the end of the study.

ENDPOINTS

The primary effectiveness endpoint is the change in KCCQ TSS from baseline to Month 3.

The key secondary effectiveness endpoints are 1) change in the daily step count from baseline to Month 3, and 2) changes in KCCQ individual domain scores (physical limitation, quality of life, clinical summary, and overall) from baseline to Month 3.

The exploratory endpoints are the following:

- mean PGIC scores over time
- changes in PGI-S scores from baseline to Month 3
- change in number of daily floors climbed
- change in HRU and health economics data
- number of outpatient intravenous therapies, ED visits, and hospitalizations
- time to first hospitalization/readmission for heart failure
- time to first hospitalization/readmission for other events
- time to death

Refer to Section 8, Study Assessments and Procedures for evaluations related to endpoints.

Adverse Event Data Collection

Due to the virtual method of data collection for this study, there will be 2 types of adverse event data collection, one by participants instructed to self-report any adverse events to a call center, and the other by an analysis of medical claims data from the participant’s provider to their insurance system. Participant-reported adverse events to the call center will be captured from the time of informed consent until 30 days after the end of treatment (ie, the last dose of study intervention) and adverse events from medical claims data from the time of informed consent to the end of study.

After the treatment period plus 30 days, serious adverse events will be identified solely through medical claims data. Adverse events that occur during the treatment period plus 30 days identified from medical claims will be aggregated by treatment assignment when unblinding is available. In addition, for this time period, self-reported adverse events from the call center will be summarized in a listing. Serious adverse event data from medical claims for the 9-month study period will be aggregated by treatment assignment.

HYPOTHESIS

The primary hypothesis is that canagliflozin is superior to placebo as assessed by improvement in the TSS on the KCCQ scale during the 3-month, double-blind treatment period.

4. STUDY DESIGN

4.1. Overall Design

This is a randomized, double-blind, placebo-controlled, decentralized, virtual, interventional, superiority study conducted in the US in participants with symptomatic HF (stratified by HFrEF and HFpEF) to examine the improvement in the KCCQ TSS after 3 months of treatment with canagliflozin 100 mg or placebo. The study will continue for an additional 6 months without any study intervention to collect participant follow-up in a real-world setting.

The study will enroll participants from large, integrated, health networks and large physician practices in the US with the participants conducting the majority of their study-related activities through an app on a smartphone and actigraphy data from a Fitbit device. There will be no in-person clinic visits required by the study.

The study consists of a screening period and a 3-month, double-blind treatment period. Approximately 1,900 participants will be randomized into the study in a 1:1 ratio to canagliflozin 100 mg daily or placebo groups (950 in each study intervention group). After Month 3 (the end of the double-blind treatment period), the study will continue for an additional 6 months when study intervention will no longer be provided. Data collection from medical claims, smartphone, Fitbit device, and selected PROs will continue until Month 9 for the purpose of secondary and further exploratory endpoints analyses. In addition, the KCCQ, PGIC, and PGI-S data will be collected at 6 and 9-months. An optional participant satisfaction survey about the virtual nature of the study will be provided to the participant at the end of the study.

Participants will be of any gender, 18 years of age or older, and have clinically stable, symptomatic HF (HFrEF or HFpEF) with or without T2DM with a KCCQ baseline overall summary score of ≤80 prior to randomization.

Participants should be receiving guideline recommended HF medications as prescribed by their treating physician(s) (such as ACEi, ARB, beta-adrenergic blocking agent or beta blocker [β-blocker], oral diuretics, MRA, angiotensin receptor-neprilysin inhibitor).

Study assessments include the KCCQ, Fitbit device data (step count and floors climbed), PGIC, PGI-S, participant satisfaction survey, medical and pharmacy claims data (including up to

36 months history for eligibility confirmation), HRU and health economics (including hospitalization/readmission due to HF or other reason, ED visits, the duration of stay at each hospitalization/readmission), review of medical claims for prohibited medications and new diagnoses that may require discontinuation, dates of deaths, and adverse events.

The total duration of study participation for each participant is approximately 9 months. Participants will be randomly assigned to receive canagliflozin 100 mg daily immediate-release over-encapsulated tablets (capsules) taken orally or placebo capsules for the duration of the 3-month, double-blind treatment period, followed by a 6-month, no treatment period during which data will continue to be collected.

Participants will be enrolled from large, integrated, health networks or large physician practices who have electronic health records (EHR) to identify participants. The primary and secondary endpoint data collection will be done via an app for a smartphone (being developed specifically for the study) as well as data from the Fitbit device.

Data integrity will be assured by automatic data collection directly from data sources (Fitbit device, Fitbit app on smartphone, structured electronic PROs on smartphone via the study app, and medical claims data from or to payers) without manual entry. Significant changes in actigraphy mid-study will be used to confirm that a Fitbit device remains associated with the participant to whom it is assigned. The Fitbit app on the participant's phone will collect all data from the Fitbit device, and significant latencies between wearable sensor data uploads via the app will be used to identify any smartphone and Fitbit device dissociation. In addition, the participant will be informed during the consenting process that the study Fitbit device must only be paired to the participant's smartphone and is to be used only by the participant.

There will be virtual study coordinating center support for initial participant onboarding to answer any study-related questions, assist with proper set up and use of the study app with the participant's smartphone and Fitbit device/app as data collection tools, confirming drug receipt and compliance, and to collect participant-reported adverse events and report them to the sponsor. The virtual study coordinating center will help participants appropriately set up their devices for the purposes of the study. In this particular instance, the main requirement for the Fitbit device is maximal wear time, as all of the sensor data will be collected passively. The blinded study intervention will be hand delivered directly to the participants from a drug distribution vendor. No formal on-site monitoring of the study will be performed as this is a decentralized clinical study, however, the sponsors' designee will centrally monitor participant medical claims, study app data, and Fitbit device data monthly for compliance with the study. For example, monitoring will include detecting non-use of the study app or Fitbit device. Medical claims data will also be monitored for inclusion/exclusion criteria, adverse events, prohibited medications, HRU, relevant concomitant medications, and new diagnoses that would require discontinuation. In addition, the study app will send reminders to participants of study items to complete assessments (KCCQ, PGI-S, PGIC), and will collect weekly study drug compliance. If lack of participation is detected by any of the monitoring, the virtual study coordinating center will contact the participants for follow-up information.

Participant Unblinding

After each randomized participant completes the 3-month, double-blind treatment period of the study, they will be provided with their treatment allocation, whether they had been randomized to canagliflozin or placebo.

A diagram of the study design is provided in Section 1.2, Schema.

4.2. Scientific Rationale for Study Design

Study Population

The study population selected represents participants with HF (HFrEF and HFpEF), with or without T2DM, who would potentially benefit from a drug intervention that could offer improvement in health status outcomes, such as clinical symptoms (eg, dyspnea and fatigue commonly experienced by these participants) and impaired quality of life (eg, physical limitations and emotional dysfunction such as depression and anxiety). Participants who meet the inclusion criteria for these conditions include adults of any gender 18 years of age and older, representing the demographic group in which these conditions are prevalent, and mortality and hospitalization rates are high, despite the recommendations of existing clinical practice guidelines.

Length of Study Periods

The screening period of 28 days allows for an appropriate length of time for screening procedures to determine study eligibility (with EHR reviews, app set-up, completion of the KCCQ, and consenting). The baseline period of 7 days begins upon randomization and allows for shipment of the study drug and Fitbit device to the participant. The 3-month, double-blind treatment period provides sufficient timepoints for effectiveness assessments to demonstrate treatment differences between canagliflozin and placebo.

Placebo Control, Randomization, Blinding, Intervention Groups

A placebo control will be used to establish the frequency and magnitude of changes in clinical endpoints that may occur in the absence of active intervention. Randomization will be used to minimize bias in the assignment of participants to intervention groups, to increase the likelihood that known and unknown participant attributes (eg, demographic and baseline characteristics) are evenly balanced across intervention groups, and to enhance the validity of statistical comparisons across intervention group. Blinded intervention will be used to reduce potential bias during data collection and evaluation of clinical endpoints.

Dose and Dosage Administration Regimen

In the CANVAS program, canagliflozin reduced the relative risk of the composite of CV death or HHF compared with placebo, with a HR of canagliflozin versus placebo of 0.78 (95% CI: 0.67, 0.91). The HRs were similar in the canagliflozin 100 and 300 mg groups. The HR for HHF in the combined canagliflozin group versus placebo was 0.67 (95% CI: 0.52, 0.87), with similar effects results seen in the canagliflozin 100 and 300 mg groups. These HR results resulted in choosing the lower dose of canagliflozin 100 mg daily for this study. In addition, because few participants

required titration to 300 mg/d in the CANVAS-R (DIA4003) study, and the treatment period in this study is relatively short, titration to the 300 mg/d dose is not necessary.

Kansas City Cardiomyopathy Questionnaire (KCCQ)

Patient-reported outcomes (PROs) are a measurement tool for the symptom burden and functional limitations in patients with HF and have demonstrated greater reproducibility than other clinical study measures like ejection fraction (EF) valve gradients,[13] 6-minute walk test, and a biomarker (B-type natriuretic peptide [BNP]).[29] The KCCQ (Section 10.3, Appendix 3) is a well-established PRO for use in patients with HF, with published validity and reliability. This instrument was developed and validated by Dr. John Spertus, the Lauer/Missouri Endowed Chair and Tenured Professor at the University of Missouri – Kansas City and the Clinical Director of Outcomes Research at Saint Luke’s Mid America Heart Institute. After reviewing its extensive psychometric profile, the KCCQ has been endorsed by the Center for Devices and Radiological Health as a Certified Outcome Assessment through its Medical Device Development Tools Program.[17] In addition, the KCCQ was recently qualified by the Center for Drug Evaluation and Research as a PRO instrument for use in clinical investigations in heart failure.[4]

The KCCQ is a 23-item, self-administered questionnaire and requires, on average, 4 to 6 minutes to complete, with a resulting score of 0 to 100, and higher scores indicating better health. It was developed to measure the patient’s perception of their health status, including their HF symptoms, impact on physical and social function and how their HF impacts the quality of life. The KCCQ has been repeatedly used as a clinically meaningful outcome measure in CV research, patient management, and quality assessment. It has been extensively tested for its validity, reliability, and responsiveness improvement in participants with HF, and is well suited as the primary endpoint for this study. In addition, the intent of the study is to enroll participants with New York Heart Association (NYHA) class II to IV. As it would be difficult to verify NYHA class with medical claims and EHR data, the sponsor will use a KCCQ overall summary score at baseline of ≤80, which is highly correlated to class II to IV in HFrEF and HFpEF based on the research published by Joseph, et al.[12]

Actigraphy Measurements

Total step count and total floors climbed will be collected continuously from the Fitbit device from each participant for the entire duration of the study. These actigraphy measures will be used to assess patient activity as it relates to treatment effect on the participant’s daily activity.

Patient Global Impression of Change (PGIC)

The PGIC (Section 10.4, Appendix 4) is a commonly accepted, validated outcome measure used in clinical studies to assess the overall change in the participant’s status as it relates to the participant’s HF symptoms since starting the study as rated by the participant.

Patient Global Impression of Severity (PGI-S)

The PGI-S (Section 10.5, Appendix 5) is a commonly accepted, validated outcome measure used in clinical studies to assess the change in the severity of the participant's HF symptoms since starting the study as rated by the participant.

Health Resource Utilization and Health Economics Data Collection

Treatment of patients with symptomatic HF with JNJ-28431754 (canagliflozin) versus placebo may result in lower utilization of any incident of hospitalization/readmission due to HF or other reason, ED visits, the duration of stay at each hospitalization/readmission, and discharge destination; therefore, comparison will be done across study intervention groups.

Participant Satisfaction Survey

Participants will be provided with an optional participant satisfaction survey at the end of the study to provide feedback on this virtual study and the use of the Fitbit device and smartphone to collect their information. This feedback may be important to consider when designing studies that use technology and devices.

4.2.1. Study-Specific Ethical Design Considerations

Potential participants will be fully informed of the risks and requirements of the study and, during the study, participants will be given any new information that may affect their decision to continue participation, including availability of an approved medicine for HF in the same SGLT2i class of medicines as canagliflozin, which now includes the recent approval of dapagliflozin for HFrEF. They will be told that their consent to participate in the study is voluntary and may be withdrawn at any time with no reason given and without penalty or loss of benefits to which they would otherwise be entitled. Only participants who are fully able to understand the risks, benefits, and potential adverse events of the study, and provide their consent voluntarily will be enrolled.

4.3. Justification for Dose

Canagliflozin 100 mg daily taken orally is the dose selected based on data from a similar study population in the CANVAS program that demonstrated a significant treatment effect, with no differences observed between the 100 and 300 mg doses. In addition, there are fewer dose-dependent adverse events such as volume-related adverse events associated with this dose. Refer to Section 4.2, Study Design Rationale for further details.

4.4. End of Study Definition

A participant will be considered to have ended the treatment period of the study (at 3 months), regardless of whether the participant has completed the study intervention, if he or she has completed assessments at Month 3 of the double-blind treatment period, withdrew earlier from the treatment, or died prior to the Month 3 assessment.

The end of study (at 9 months) is considered as the last visit shown in the Schedule of Activities for the last participant in the study. The final data from the study clinical research organization

partner will be sent to the sponsor (or designee) after completion of the collection of the study data from the Fitbit device and the claims in the time frame specified in the Trial Agreement.

5. STUDY POPULATION

Screening for eligible participants will be performed within 28 days before randomization into the study.

The inclusion and exclusion criteria for enrolling participants in this study are described below. If there is a question about these criteria, the participant or designee must consult with the call center to resolve any issues before enrolling in the study.

For a discussion of the statistical considerations of participant selection, refer to Section 9.2, Sample Size Determination.

5.1. Inclusion Criteria

Each potential participant must satisfy all of the following criteria to be enrolled in the study at enrollment (consent date):

1. any gender
2. 18 (or the legal age of consent in the jurisdiction in which the study is taking place) years of age or older
3. Criterion modified per Amendment 2

   3.1. have clinically stable symptomatic HF (HFrEF or HFpEF)

   For HFrEF:

   a) EF ≤40% AND

   b) a primary diagnosis of HF OR 2 medical visits (including virtual) with a HF diagnosis code in any position in the past 18 months.

   For HFpEF:

   a) EF >40% AND

   b) a primary diagnosis of HF OR 2 medical visits (including virtual) with a HF diagnosis code in any position in the past 18 months, AND

   c) on a loop diuretic or spironolactone or eplerenone (mineralocorticoid receptor antagonists)[1,3] in the past 18 months.
4. Criterion modified per Amendment 2

4.1. have a KCCQ baseline overall summary score of ≤80 prior to randomization

5. be able to read and understand English

6, possess and have sole use (eg, not shared with other users) of smartphone compatible with the Fitbit device

7. willing/able to wear the Fitbit device on a regular basis for the 9-month study period

8. must sign an electronic informed consent form (eICF) indicating that he or she understands the purpose of, and procedures required for, the study and is willing to participate in the study, including follow-up.

5.2. Exclusion Criteria

Any potential participant who meets any of the following criteria prior to enrollment (consent date) will be excluded from participating in the study:

1. Criterion modified per Amendment 2

   1.1. currently taking an SGLT2i or within the last 3 months

2. history of diabetic ketoacidosis or have type 1 diabetes mellitus (T1DM)

3. Criterion modified per Amendment 2

   3.1. have acute decompensated HF (exacerbation of symptomatic HF) requiring intravenous diuretics, inotropes, or vasodilators within the last 4 weeks

4. Criterion modified per Amendment 2

   4.1. have stage 4 or 5 Chronic Kidney Disease (ie, eGFR <30ml/min on dialysis) from the most recent assessment

5. Criterion modified per Amendment 2

   5.1. have a history of atraumatic amputation within past 12 months of screening, or an active skin ulcer, osteomyelitis, gangrene, or critical ischemia of the lower extremity within 6 months

6. Criterion modified per Amendment 2

   6.1. have a diagnosis of hypotension within 30 days

7. Criterion modified per Amendment 2

7.1. had major surgery within 3 months or have any surgery, ie, cardiac surgery, planned during the 3-month treatment (except for minor surgery, ie, outpatient surgery under local anesthesia)

8. Criterion modified per Amendment 2

8.1. any condition for which, in the opinion of the investigator, participation would not be in the best interest of the participant (eg, compromise the well-being) or that could prevent, limit, or confound the protocol-specified assessments or have a life expectancy of <6 months or current immobility

9. have known allergies, hypersensitivity, or intolerance to JNJ-28431754 (canagliflozin) or its excipients (refer to Investigator's Brochure, Canagliflozin[5])

10. be a woman participant who is pregnant, or breastfeeding, or planning to become pregnant while enrolled in this study

11. are legally incompetent

12. Criterion modified per Amendment 2

12.1. currently enrolled in an investigational study receiving an investigational study medication

13. has a left ventricular assist device

14. patient identity or association with enrolling network cannot be verified

NOTE: The required source documentation to support meeting the enrollment criteria are noted in Section 10.2, Appendix 2: Regulatory, Ethical, and Study Oversight Considerations.

5.3. Prohibitions and Restrictions

Potential participants must be willing and able to adhere to the following lifestyle restrictions during the course of the study to be eligible for participation:

1. Refer to details in Section 6.5, Prohibited Medications.

2. Agree to follow all requirements that must be met during the study as noted in the Inclusion and Exclusion Criteria.

5.4. Screen Failures

Individuals who do not meet the criteria for participation in this study may be rescreened. Generally, a participant may only be rescreened once, but an additional screening may be allowed with concurrence by the sponsor.

Participant Identification

All potential participants who opt in to participate in the study on the website will receive a unique identifier in the study database held by a third-party representative. All data entered and collected via the study app with the participant's smartphone and Fitbit device are time and date stamped for completeness. Those participants who electronically sign the remote e-consent but do not enroll in the study can be tracked up to the point that they no longer interact with the app. Reports will be available to permit a detailed listing of each participant from remote e-consent through the study.

6. STUDY INTERVENTION

6.1. Study Interventions Administered

The study intervention, JNJ-28431754 (canagliflozin), will be provided as immediate-release, over-encapsulated tablets (capsules) or placebo capsules taken orally once daily before the first meal of the day for the duration of the 3-month, double-blind treatment period. The capsules should be swallowed intact and participants should not attempt to dissolve them in water. The following table provides a description of the study interventions.

Table 2: Description of Study Interventions

**Group Name**	Group 1	Group 2
**Study Intervention Name**	canagliflozin	placebo
**Type**	drug	placebo
**Dose Formulation**	over-encapsulated tablets (capsules)	capsule
**Unit Dose Strength**	100 mg	0 mg
**Dosage Level**	100 mg	0 mg
**Route of Administration**	oral	oral
**Use**	experimental	placebo-comparator
**Sourcing**	Provided centrally by the sponsor	Provided centrally by the sponsor
**Packaging and Labeling**	Individual participant bottles	Individual participant bottles
	Child resistant	Child resistant
**Brand Name**	INVOKANA®	Not applicable

JNJ-28431754 (canagliflozin) will be manufactured and provided under the responsibility of the sponsor. Refer to the Investigator's Brochure, Canigliflozin[11] for a list of excipients.

6.2. Preparation/Handling/Storage/Accountability

All study intervention must be stored at controlled temperatures ranging from 20°C to 25°C (68°F to 77°F); excursions permitted between 15°C to 30°C (59°F to 86°F) and kept out of the reach of children.

The drug depot can refer to the pharmacy manual investigational product and procedures manual for additional guidance on study intervention preparation, handling, and storage.

The drug depot is responsible for ensuring that all study intervention received at the depot is inventoried and accounted for throughout the study. The dispensing of study intervention to the participant must be documented at the drug depot.

Study intervention must be handled in strict accordance with the protocol and the container label and must be stored at the drug depot in a secure area under appropriate environmental conditions.

Study intervention will be delivered to the participant by a qualified member of the drug depot/vendor. Study intervention will be supplied only to participants participating in the study. Participants will be instructed on how to store study intervention and how to destroy any unused study intervention at the end of the 3-month, double-blind treatment period.

6.3. Measures to Minimize Bias: Randomization and Blinding

Intervention Allocation

Procedures for Randomization and Stratification

Central randomization will be implemented in this study. Participants will be randomly assigned to 1 of 2 study intervention groups based on a computer-generated randomization schedule prepared before the study by or under the supervision of the sponsor before initiation of the study.

Randomization will be balanced by using randomly permuted blocks and will be stratified by EF (HFrEF or HFpEF) at study entry as recorded in the participant's EHR.

The interactive web response system (IxR) will generate a randomization code, participant randomization number, and kit numbers once consent is completed and the participant is confirmed eligible by the virtual principal investigator. The kit number will dictate the study intervention assignment and the matching study intervention bottle(s) to be shipped to the participant directly from the drug distribution vendor.

Blinding

The IxR will provide the kit number of the study intervention bottle(s) to be dispensed for each randomly assigned participant to begin dosing on Day 1 of the double-blind treatment period. Based on these randomization codes, study intervention will be packaged and labeled in a manner that maintains the double-blinded nature of the study.

The drug depot will not be provided with randomization codes. The codes will be maintained within the IxR, which has the functionality to allow the virtual principal investigator or designee to break the blind for an individual participant.

Under normal circumstances, the blind should not be broken until participants have completed the 3-month, double-blind treatment period. The Study Responsible Physician or other sponsor physician designee may in an emergency determine the identity of the study intervention by contacting the IxR. Telephone contact with the sponsor or its designee will be available 7 days per week. The date and reason for the unblinding must be documented in the study database. The documentation received from the IxR indicating the code break must be retained. All randomization codes will be released after completion of the study. The translation of randomization codes into treatment and control groups will be disclosed only to those authorized. Participants who have had their intervention assignment unblinded should continue to perform their scheduled evaluations.

6.4. Study Intervention Compliance

Participants will be instructed and notified by the study app weekly to confirm the number of study intervention capsules that they took that week. Study intervention compliance data will be monitored bi-weekly and if compliance is not >80%, the participant will be contacted by the call center who will re-educate the participant on the importance of diary completion and taking study intervention daily.

6.5. Prohibited Medications

Prohibited medications include other SGLT2i medications (including commercially available canagliflozin); participants must not take any other investigational agents during the study.

Medications will be reviewed monthly through medical claims data by the sponsor or designee. The sponsor must be notified as soon as possible of any instances in which prohibited therapies are administered.

7. DISCONTINUATION OF STUDY INTERVENTION AND PARTICIPANT DISCONTINUATION/WITHDRAWAL

7.1. Discontinuation of Study Intervention

A participant's study intervention must be discontinued during the 3-month, double-blind treatment period if:

- the participant has started another SGLT2i
- the participant has been diagnosed with T1DM

- for safety reasons or tolerability reasons (eg, adverse event), it is in the best interest of the participant to discontinue study intervention
- the participant develops severe renal impairment or End Stage Renal Disease, or is on dialysis
- the participant becomes pregnant
- the participant develops diabetic ketoacidosis
- the participant develops a new active skin ulcer, osteomyelitis, gangrene, or critical ischemia of the lower extremity or has an amputation.

The participant may choose to discontinue the study intervention only and remain in the study. In this case, the participant will continue to perform assessments (KCCQ, PGI-S, PGIC) and allow continued passive data collection from the app, Fitbit, and medical claims. If contacted during the study, the call center or designee will inform the participant of the importance to the study and results to have ongoing ascertainment of assessments and vital status.

Additional information on informed consent for collection of vital status can be found in Section 10.2, Appendix 2: Regulatory, Ethical, and Study Oversight Considerations.

7.1.1. Temporary Discontinuation of Study Intervention

Study intervention may be temporarily discontinued; however, these interruptions should be kept to a minimum. Study intervention can be resumed when the participant, the virtual principal investigator, or the participant's physician deems it is appropriate to do so.

7.2. Participant Discontinuation/Withdrawal From the Study

A participant will be withdrawn from the study for any of the following reasons:

- lost to follow-up
- withdrawal of consent
- death

In the event a participant withdraws consent and does not agree to any kind of follow-up and specifically refuses any further contact with the call center or designee, this must be documented in the study database. If a participant is lost to follow-up, vital status will still be attempted for collection at study end through the participant's physician, medical claims, or public information according to local guidelines and as allowed by local regulations.

If a participant is lost to follow-up, every reasonable effort must be made by the call center or designee to contact the participant to determine vital status (eg, alive or dead) and the reason for discontinuation/withdrawal. This should include repeated telephone calls, certified letters, email requests, etc. The study screeners and app will obtain primary telephone contact number (eg, smartphone numbers), as well as other contact information (eg, email addresses) from participants before randomization. In addition, the call center should emphasize the importance of follow-up information to the participant before randomization. Follow-up can also be done with the help of the study site personnel. The measures taken to obtain follow-up information must be documented.

Unless consent is specifically withdrawn, participants are expected to be followed up through 1 of the alternative follow-up mechanisms described above.

Before withdrawing from the study, participants who request to withdraw from the study should be asked if they agree to be contacted to obtain follow-up information.

8. STUDY ASSESSMENTS AND PROCEDURES

Overview

The Schedule of Activities (SoA) summarizes the frequency and timing of effectiveness, medical resource utilization, health economic, and safety measurements applicable to this study.

Study-Specific Materials

The participants will be provided with the following supplies prior to starting the study:

- study intervention supply
- Fitbit device
- instruction manual(s) and patient drug information handouts.

8.1. Effectiveness Assessments

The primary effectiveness endpoint (KCCQ TSS) will be assessed at baseline and Weeks 2, 4, 6, and 12 during the double-blind treatment period (first 3 months) and afterwards at 6 and 9 months when no study intervention is administered. The following sensor data will be obtained from the Fitbit: step count and floors climbed.

8.1.1. KCCQ Scores

The KCCQ is a 23-item, self-administered questionnaire on the study app and requires, on average, 4 to 6 minutes to complete. It was developed to measure the patient's perception of their health status, including their HF symptoms, impact on physical and social function and how their HF impacts the quality of life. It scored by assigning a number for each response beginning with 1 that implies the lowest level of functioning and summing items within each domain, dividing by the range and multiplying by 100.[10] Missing values within each domain are assigned the average of the answered items within that same domain, presuming that at least half of the items within that domain are completed. Scale scores are transformed into values ranging from 0 to 100, with higher scores indicating more favorable health status.[10]

The answers provided by participants to the KCCQ's questions are used to calculate scores for the following 10 scales:

- Physical Limitation: a measure of how much a patient's routine activities are hampered by symptoms of HF
- Symptom Stability: a measure of whether a participant's symptoms have changed over the past 2 weeks

- Symptom Frequency: a measure of how often a participant has HF symptoms
- Symptom Burden: a measure of how much the participant's symptoms bother them
- Total Symptom: a combined measure of the symptom frequency and burden scales
- Social Limitation: a measure of how much a participant's social and work activities are limited by their HF symptoms
- Self-Efficacy: a measure of how well a participant can manage her care, find answers and help
- Quality of Life: a measure of the overall impact of a participant's HF on their perceived quality of life
- Clinical Summary: a combined measure of symptoms and physical limitations, congruent with the considerations a physician uses in assigning their NYHA classification
- Overall Summary: a combined measure of the participant's physical limitation, total symptom, social limitation, and quality of life scores.

In this study, at screening/baseline, KCCQ overall summary score will be evaluated to determine participant eligibility into the study. All other KCCQ domains and summary scores will also be calculated at baseline and at subsequent post-baseline time points respectively.

8.1.2. Actigraphy Measurements

Once randomized into the study, data from the Fitbit device on each participant will be collected continuously pertaining to the total step counts and total floors climbed for the entire duration of the study. Actigraphy measures (ie, step counts and floors climbed) will be examined in relation to the PRO measures and clinical outcomes to better characterize treatment effect on daily activity.

8.2. Exploratory Assessments

8.2.1. Patient Global Impression of Change (PGIC) Scores

The PGIC is a global index that is used to rate the overall status of the participant related to the participant's condition. It is rated by the participant and is based on the single question, "Since the start of the treatment you've received in this study, your heart failure symptoms are", where 1=very much improved, 2=somewhat improved, 3=a little improved, 4=no change, 5=a little worse, 6=somewhat worse, and 7=very much worse. Refer to Section 10.4, Appendix 4: Patient Global Impression of Change (PGIC).

8.2.2. Patient Global Impression of Severity (PGI-S) Scores

The PGI-S is a global index that is used to rate the severity of a specific condition. It is rated by the participant and is based on the single question, "Considering all aspects of your heart failure symptoms right now, would you say your heart failure symptoms are", where 1=none, 2=mild, 3=moderate, 4=severe, and 5=very severe. Refer to Section 10.5, Appendix 5: Patient Global Impression of Severity (PGI-S).

8.2.3. Participant Satisfaction Survey

An optional participant satisfaction survey to assess the participant's satisfaction with the virtual design of the study and use of the Fitbit device in the study will be provided to the participant at the end of study. Refer to Section 10.6, Appendix 6: Participant Satisfaction Survey.

8.2.4. Healthcare Resource Utilization and Health Economics

Healthcare Resource Utilization (HRU) and health economics data associated with medical encounters will be collected for all participants during the study. Protocol-mandated procedures, tests, and encounters are excluded. The data collected may be used to conduct exploratory economic analyses and will include:

- number and duration of first hospitalizations/readmissions (overall inpatient visit length of stay) for HF or any other reason
- number and duration of first hospitalizations/readmissions (overall inpatient visit length of stay) for any other events
- number of emergency room visits
- discharge destination

8.3. Adverse Events and Serious Adverse Events and Special Situations

Participants will be instructed to self-report any adverse events to a call center. In addition, this information may be discovered by the call center during interaction with the participant. Once identified, the call center will complete and submit a solicited safety reporting form to Janssen Global Medical Safety (GMS) per the sponsor's standard operating procedure. Queries for these cases will go to the call center/and virtual principal investigator as appropriate. If follow-up is needed with the participant's treating physician, it will be done either through the virtual principal investigator, who may contact the treating physician directly, as agreed upon in the ICF. All SAEs will be assessed by Janssen GMS for potential suspected unexpected serious adverse reactions (SUSARs) for expedited regulatory reporting.

Medical claims adverse event data will be reviewed and evaluated including potential SUSAR assessment and regulatory reporting in aggregate at the end of the 3-month, double-blind treatment period when the unblinded data are available. In addition, for this time period, self-reported adverse events from the call center will be summarized in a listing. Serious adverse event data from medical claims for the 9-month study period will be aggregated by treatment assignment. Self-reported adverse events will be coded according to the Medical Dictionary for Regulatory Activities (MedDRA) and will be reported by preferred term and by T2DM and non-T2DM. Adverse events collected from medical claims will be aggregated based on diagnosis codes for the incidences of non-endpoint SAEs (ie, SAEs leading to hospitalizations, ED visits) and adverse events of interest between groups reported by T2DM and non-T2DM. Discontinuations will be summarized based on any available data.

8.3.1. Time Period and Frequency for Collecting Adverse Event and Serious Adverse Event Information

All Adverse Events

Adverse events will be captured through medical claims (from the time of informed consent to the end of study) and by the call center (from the time of informed consent until 30 days after the end of treatment). After the treatment period plus 30 days, serious adverse events will be identified solely through medical claims data. Adverse events that occur during the treatment period plus 30 days identified from medical claims will be aggregated by treatment assignment when unblinding is available. In addition, for this time period, self-reported adverse events from the call center will be summarized in a listing. Serious adverse event data from medical claims for the 9-month study period will be aggregated by treatment assignment.

8.3.2. Regulatory Reporting Requirements for Serious Adverse Events

The sponsor assumes responsibility for appropriate reporting of adverse events to the regulatory authorities. The sponsor will also report to the virtual principal investigator (and the head of the investigational institute where required) all SUSARs. The virtual principal investigator (or sponsor where required) must report SUSARs to the appropriate Independent Ethics Committee/Institutional Review Board (IEC/IRB) that approved the protocol unless otherwise required and documented by the IEC/IRB. A SUSAR will be reported to regulatory authorities unblinded. The virtual principal investigator and IEC/IRB will receive a blinded SUSAR summary, unless otherwise specified.

8.3.3. Pregnancy

Female participants and male participants with a partner becoming pregnant will be instructed to call the call center for any pregnancy. The call center will report the pregnancy to the sponsor within 24 hours of their knowledge of the event using the appropriate pregnancy notification form. Abnormal pregnancy outcomes (eg, spontaneous abortion, fetal death, stillbirth, congenital anomalies, ectopic pregnancy) are considered adverse events and must also be self-reported by the participants to the call center. Any participant who becomes pregnant during the study must be promptly discontinued from the study intervention.

Follow-up information regarding the outcome of the pregnancy and any postnatal sequelae in the infant will be required.

8.3.4. Adverse Events of Interest

Adverse events of interest include all severe hypoglycemic events, acute kidney injury, fractures, diabetic ketoacidosis, and lower limb amputation.

In addition, the following adverse events will be examined: hypotension; Fournier’s gangrene; genital mycotic infections; urinary tract infections (including urosepsis and pyelonephritis); increased urination; and hypersensitivity reactions including angioedema and anaphylaxis.

9. STATISTICAL CONSIDERATIONS

Statistical analysis will be done by the sponsor or under the authority of the sponsor. A general description of the statistical methods to be used to analyze the effectiveness and safety data is outlined below. Specific details will be provided in the SAP.

The primary study objective will be addressed by comparing the mean change in KCCQ TSS from baseline to Month 3 between the study intervention groups. The key secondary study objective will involve a comparison between the 2 randomized groups in terms of the mean difference in daily step counts from baseline to Month 3. For analyzing the primary, secondary, and exploratory objectives, an intent-to-treat (ITT) population comprising of all randomized participants will be used. Specific imputation rules for missing data (eg, missing KCCQ TSS, daily step count) will be applied, if warranted, and will be specified in the SAP before the final database lock. Descriptive statistics such as mean, median, standard deviation, interquartile range, minimum and maximum will be used to summarize continuous variables. Counts and proportions will be used to summarize categorical variables. Graphical data displays (eg, box plots) may be employed to summarize data. For time to event variables, Kaplan-Meier estimates over time will be plotted. All statistical tests will be two-sided and performed at the 5% significance level unless otherwise specified. No multiplicity adjustment to the overall Type I error rate will be applied when analyzing the exploratory variables.

9.1. Statistical Hypotheses

For the primary effectiveness endpoint, the null hypothesis is that the canagliflozin 100 mg is not different from placebo with respect to the mean KCCQ TSS change from baseline at Month 3. For the first key secondary effectiveness endpoint of daily step count, the null hypothesis is that canagliflozin 100 mg is not different from placebo with respect to the mean daily step count.

9.2. Sample Size Determination

The primary effectiveness endpoint is the change from baseline in the KCCQ TSS at the end of the 3-month double-blind treatment period. Although a 5-point improvement in the KCCQ TSS is generally viewed as clinically meaningful,[5,29] recent studies[16,21] have shown that improvements in the range of 3 to 5 points on the KCCQ TSS are equally meaningful in the overall HF-related health status measured by the KCCQ over a 12-week time period. Based on the evidence from these studies, the current study is powered to detect a mean change from baseline of 3 points in the KCCQ TSS between canagliflozin 100 mg and placebo groups at 12 weeks. Assuming a significance level of 5% and a standard deviation of 18 points for mean change in KCCQ TSS, a total of 1,900 participants will be randomized in this study providing approximately 95% power. This sample size is adequate to account for a potential 5% dropout rate.

9.3. Populations for Analyses

For purposes of analysis, the following populations are defined:

Table 3: Analysis Populations

Population	Description
Enrolled	All participants who sign the ICF
Intent-to-Treat (ITT)	All randomized participants
Per Protocol (PP)	A subset of the ITT population. Randomized participants who received study drug and with major protocol deviations will be excluded from the PP population. Major protocol deviations will be defined in the SAP.

9.4. Statistical Analyses

9.4.1. Handling of Missing Data

Missing data affecting the primary endpoint change in KCCQ TSS will be addressed via the MMRM method that assumes missingness at random (MAR). Specific imputation rules including statistical modeling approaches may be employed when such an assumption is not valid. In cases where individual items in the KCCQ domains are missing or not completed at required times, appropriate rules to impute missing item scores will be specified based on the KCCQ manual. Sensitivity analyses will be conducted to assess impact of missing data on key study results. Further details about the imputation rules and sensitivity analyses will be provided in the SAP.

9.4.2. Effectiveness Analyses

9.4.2.1. Primary and Secondary Effectiveness Analyses

The primary and key secondary effectiveness analyses will be based on the ITT analysis population (using all randomized participants).

As KCCQ TSS is measured repeatedly over time (ie, at baseline, Week 2, Week 4, Week 6, and Week 12 respectively), the primary effectiveness endpoint (change in KCCQ TSS from baseline to Month 3) will be analyzed by a mixed effect model repeated measures (MMRM) method simultaneously adjusting for study intervention group, stratification factor (HFrEF versus HFpEF), time, time by study intervention group, and baseline KCCQ TSS value as covariates. An unstructured covariance structure will be assumed across study intervention groups to model the within-participant errors. The primary comparison will be based on the difference in least squares between treatments at Month 3. The treatment difference in the least squares means and their 2-sided 95% confidence interval (CI) will be provided. An MMRM model accounts for data missing at random.

A responder analysis will also be performed when comparing proportions of participants with a 5- (or 10-) point improvement in KCCQ TSS from baseline between the study intervention groups. This will be based on a two-sample difference in proportions test along with a 95% confidence interval for the difference in 2 proportions. Additionally, empirical cumulative distribution functions of the KCCQ TSS and other domain scores will be presented.

The primary effectiveness endpoint will also be analyzed by subgroups defined by age, gender, race and key baseline disease/demographic characteristics using the MMRM approach described above. Further details will be provided in the SAP. Quantitative and/or qualitative interactions for each subgroup and treatment will be identified. Qualitative interactions will be investigated further by the Gail and Simon test[9] and interpreted accordingly.

The first key secondary endpoint of daily step count will be tested once the primary endpoint is shown to be significant at the 5% significance level using a two-sided t-test. This approach will control for the overall Type I error rate at 5%.

For the second key effectiveness endpoint, an MMRM model (like that used in the analysis of the primary endpoint) will be employed to summarize changes in KCCQ individual domain scores (physical limitation, quality of life, clinical summary, and overall) from baseline to Month 3. This hypothesis will be independently tested at 5% without any control for overall Type I error rate.

9.4.2.2. Exploratory Analyses

Exploratory analyses will be performed with no adjustment for multiplicity and include the following:

- summary statistics of PGIC and PGI-S scores
- associations (correlations and/or categorical association measures) between digital markers (step count and floors climbed) and clinical events such as need for outpatient intravenous therapies, ED visits, and hospitalizations
- tabulation and comparison of total HRU and health economics data across groups.
- descriptive statistics (eg, mean, median, and standard deviation) of daily floors climbed
- association between the KCCQ change from baseline to Month 3 and the PGIC at Month 3 will be examined and summarized
- time to death
- descriptive statistics of the participant's satisfaction with his/her experience with the virtual design at the end of the study.

9.4.3. Adverse Events Analysis

Adverse events reported to the call center will be assessed by Janssen GMS for causality and meeting SUSAR reporting requirements. Self-reported adverse events will be coded according to MedDRA and will be reported by preferred term by T2DM and non-T2DM. Adverse events collected from medical claims will be aggregated based on diagnosis codes for the incidences of non-endpoint SAEs (ie, SAEs leading to hospitalizations, ED visits) and adverse events of interest between groups reported by T2DM and non-T2DM. Discontinuations will be summarized based on any available data. Adverse events reported from the call center will be reported separately from those obtained from the medical claims data. Self-reported adverse events from the call center will be summarized in a listing.

9.4.4. Healthcare Resource Utilization and Health Economics Analyses

Healthcare resource utilization (HRU) and health economics data will be descriptively summarized by intervention group.

9.5. Interim Analysis

No interim analysis is planned for this study.

10. SUPPORTING DOCUMENTATION AND OPERATIONAL CONSIDERATIONS

10.1 Appendix 1: Abbreviations

ACEi	angiotensin-converting enzyme inhibitor
AE	adverse event
AHA	antihyperglycemic agent
ARB	angiotensin II receptor blocker
ARNi	angiotensin receptor-neprilysin inhibitor
BB	beta blocker
CANVAS	short title for study, Canagliflozin Cardiovascular Assessment Study
CANVAS-R	short title for study, Canagliflozin Cardiovascular Assessment Study-Renal
CI	confidence interval
CREDENCE	short title for study, Canagliflozin and Renal Events in Diabetes with Established Nephropathy Clinical Evaluation Trial
CV	cardiovascular
CVD	cardiovascular disease
DKA	diabetic ketoacidosis
DMC	Data Monitoring Committee
ED	emergency department
EHR	electronic health record
EMPA	empagliflozin
ESKD	End Stage Kidney Disease
GCP	Good Clinical Practice
GMS	Global Medical Safety
HF	heart failure
HFpEF	heart failure with preserved ejection fraction
HFrEF	heart failure with reduced ejection fraction
HHF	hospitalization for heart failure
HR	hazard ratio
HRU	healthcare resource utilization
ICF	informed consent form
ICH	International Conference on Harmonisation
IEC	Independent Ethics Committee
INVOKANA	canagliflozin
IRB	Institutional Review Board
ITT	intent-to-treat
IxR	interactive web response system
KCCQ	Kansas City Cardiomyopathy Questionnaire
MACE	major adverse cardiovascular events
MAR	missing at random
MDDT	Medical Device Development Tools
MedDRA	Medical Dictionary for Regulatory Activities
MI	myocardial infarction
MMRM	mixed effect model repeated measure
NYHA	New York Heart Association
PGIC	Patient Global Impression of Change
PGI-S	Patient Global Impression of Severity
PQC	Product Quality Complaint
PRO	patient-reported outcome(s) (paper)
SAE	serious adverse event
SAP	statistical analysis plan
SGLT2	sodium-glucose co-transporter 2
SGLT2i	sodium-glucose co-transporter 2 inhibitor
SoA	schedule of activities

SUSAR	suspected unexpected serious adverse reaction
T1DM	type 1 diabetes mellitus
T2DM	type 2 diabetes mellitus
TSS	Total Symptom Score
US	United States

10.2 Appendix 2: Regulatory, Ethical, and Study Oversight Considerations

REGULATORY AND ETHICAL CONSIDERATIONS

Virtual Principal Investigator Responsibilities

The virtual principal investigator or designee is responsible for ensuring that the study is performed in accordance with the protocol, current ICH guidelines on Good Clinical Practice (GCP), and applicable regulatory and country-specific requirements.

Good Clinical Practice is an international ethical and scientific quality standard for designing, conducting, recording, and reporting studies that involve the participation of human participants. Compliance with this standard provides public assurance that the rights, safety, and well-being of study participants are protected, consistent with the principles that originated in the Declaration of Helsinki, and that the study data are credible.

Protocol Amendments

The sponsor will not modify this protocol without a formal amendment by the sponsor. All protocol amendments must be issued by the sponsor. Protocol amendments must not be implemented without prior IEC/IRB approval, or when the relevant competent authority has raised any grounds for non-acceptance, except when necessary to eliminate immediate hazards to the participants, in which case the amendment must be promptly submitted to the IEC/IRB and relevant competent authority. When the change(s) involve only logistic or administrative aspects of the study, the IEC/IRB (where required) only needs to be notified.

During the course of the study, in situations where a departure from the protocol is unavoidable, the virtual principal investigator or designee will contact the appropriate sponsor representative listed in the Contact Information page(s), which will be provided as a separate document. Except in emergency situations, this contact should be made before implementing any departure from the protocol. In all cases, contact with the sponsor must be made as soon as possible to discuss the situation and agree on an appropriate course of action. These will be recorded by the sponsor.

Regulatory Approval/Notification

This protocol and any amendment(s) must be submitted to the appropriate regulatory authorities in each respective country, if applicable. A study may not be initiated until all local regulatory requirements are met.

Required Prestudy Documentation

The following documents must be provided to the sponsor before shipment of study intervention to the drug depot:

- protocol and amendment(s), if any, signed and dated by the principal investigator
- a copy of the dated and signed (or sealed, where appropriate per local regulations), written IEC/IRB approval of the protocol, amendments, ICF, any recruiting materials, and if applicable, participant compensation programs. This approval must clearly identify the

specific protocol by title and number and must be signed (or sealed, where appropriate per local regulations) by the chairman or authorized designee.

- name and address of the IEC/IRB, including a current list of the IEC/IRB members and their function, with a statement that it is organized and operates according to GCP and the applicable laws and regulations. If accompanied by a letter of explanation, or equivalent, from the IEC/IRB, a general statement may be substituted for this list. If the principal investigator is a member of the IEC/IRB, documentation must be obtained to state that this person did not participate in the deliberations or in the vote/opinion of the study.
- regulatory authority approval or notification, if applicable
- signed and dated statement of virtual principal investigator (eg, Form FDA 1572) and current curriculum vitae (CV), if applicable
- documentation of virtual principal investigator or designee qualifications (eg, curriculum vitae)
- completed virtual principal investigator or designee financial disclosure form, where required
- signed and dated clinical trial agreement, which includes the financial agreement
- any other documentation required by local regulations.

The following documents must be provided to the sponsor before enrollment of the first participant:

- completed virtual principal investigator or designee financial disclosure forms
- documentation of virtual principal investigator or designee qualifications (eg, curriculum vitae).

Independent Ethics Committee or Institutional Review Board

Before the start of the study, the virtual principal investigator or designee (or sponsor where required) will provide the IEC/IRB with current and complete copies of the following documents (as required by local regulations):

- final protocol and, if applicable, amendments
- sponsor-approved ICF (and any other written materials to be provided to the participants)
- Investigator's Brochure, Canigliflozin[11] (or equivalent information) and amendments/addenda
- sponsor-approved participant recruiting materials
- information on compensation for study-related injuries or payment to participants for participation in the study, if applicable
- virtual principal investigator or designee curriculum vitae or equivalent information (unless not required, as documented by the IEC/IRB)
- information regarding funding, name of the sponsor, institutional affiliations, other potential conflicts of interest, and incentives for participants
- any other documents that the IEC/IRB requests to fulfill its obligation.

This study will be undertaken only after the IEC/IRB has given full approval of the final protocol, amendments (if any, excluding the ones that are purely administrative, with no consequences for participants, data or study conduct, unless required locally), the ICF, applicable recruiting materials, and participant compensation programs, and the sponsor has received a copy of this approval. This approval letter must be dated and must clearly identify the IEC/IRB and the documents being approved.

During the study, the principal investigator (or sponsor where required) will send the following documents and updates to the IEC/IRB for their review and approval, where appropriate:

- protocol amendments (excluding the ones that are purely administrative, with no consequences for participants, data or study conduct)
- revision(s) to ICF and any other written materials to be provided to participants
- if applicable, new or revised participant recruiting materials approved by the sponsor
- new edition(s) of the Investigator's Brochure, Canigliflozin[11] and amendments/addenda
- summaries of the status of the study at intervals stipulated in guidelines of the IEC/IRB (at least annually)
- reports of adverse events that are of interest, special interest, serious, unlisted/unexpected, and associated with the study intervention
- new information that may adversely affect the safety of the participants or the conduct of the study
- deviations from or changes to the protocol to eliminate immediate hazards to the participants
- report of deaths of participants under the virtual principal investigator's care
- notification if a new virtual principal investigator is responsible for the study
- development Safety Update Report and Line Listings, where applicable
- any other requirements of the IEC/IRB.

For all protocol amendments (excluding the ones that are purely administrative, with no consequences for participants, data or study conduct), the amendment and applicable ICF revisions must be submitted promptly to the IEC/IRB for review and approval before implementation of the change(s).

At least once a year, the IEC/IRB will be asked to review and reapprove this study, where required.

At the end of the study, the virtual principal investigator or designee (or sponsor where required) will notify the IEC/IRB about the study completion (if applicable, the notification will be submitted through the head of investigational institution).

Other Ethical Considerations

For study-specific ethical design considerations, refer to Section 4.2.1.

FINANCIAL DISCLOSURE

The virtual principal investigator or designee will provide the sponsor with sufficient, accurate financial information in accordance with local regulations to allow the sponsor to submit complete and accurate financial certification or disclosure statements to the appropriate regulatory authorities. The virtual principal investigator is responsible for providing information on financial interests during the course of the study and for 1 year after completion of the study.

Refer to Required Prestudy Documentation (above) and contracts for details on financial disclosure.

INFORMED CONSENT (REMOTE E-CONSENT) PROCESS

Each participant must give remote e-consent according to local requirements after the nature of the study has been fully explained. The remote e-consent must be electronically signed within the study app before the performance of any study-related activity. The remote e-consent that is/are used must be approved by both the sponsor and by the reviewing IRB and be in a language that the participant can read and understand. The remote e-consent should be in accordance with principles that originated in the Declaration of Helsinki, current ICH and GCP guidelines, applicable regulatory requirements, and sponsor policy. After having obtained the consent, a copy of the remote e-consent must be sent to the participant.

In this study, a remote e-consent process will be utilized. Before enrollment in the study, the participant will have an opportunity to read through the remote e-consent which will detail the aims, methods, reasonably anticipated benefits, and potential hazards of the study, and any discomfort participation in the study may entail. The remote e-consent will explain that their participation is voluntary and that they may withdraw consent to participate at any time. They will be informed that choosing not to participate will not affect the care they will receive. Finally, they will be told that their records may be accessed by health authorities and authorized sponsor personnel without violating their confidentiality, to the extent permitted by the applicable law(s) or regulations. By electronically signing the remote e-consent the participant is authorizing such access, which includes permission to obtain information about his or her survival status. It also denotes that they are agreeing to allow recontact by the study sponsor for the purpose of obtaining consent for additional evaluations, if needed.

If the participant has any questions about the study related to the remote e-consent process prior to providing their electronic signature, they will be provided with an opportunity to discuss these questions with the virtual principal investigator via phone and/or a contact at the study call center. Once the participant understands all aspects of the remote e-consent, consent should be appropriately recorded by means of the participant’s personally dated signature via electronic signature. After having obtained the consent, the participant will receive a copy of the remote e-consent via email (after the participant has verified his or her email address) for their records.

DATA PROTECTION

Privacy of Personal Data

The collection and processing of personal data from participants enrolled in this study will be limited to those data that are necessary to fulfill the objectives of the study.

These data must be collected and processed with adequate precautions to ensure confidentiality and compliance with applicable data privacy protection laws and regulations. Appropriate technical and organizational measures to protect the personal data against unauthorized disclosures or access, accidental or unlawful destruction, or accidental loss or alteration must be put in place. Sponsor personnel whose responsibilities require access to personal data agree to keep the identity of participants confidential.

The informed consent obtained from the participant includes explicit consent for the processing of personal data and for the investigator/institution to allow direct access to his or her original medical records (source data/documents) for study-related monitoring, audit, IEC/IRB review, and regulatory inspection. This consent also addresses the transfer of the data to other entities.

The participant has the right to request through the virtual principal investigator access to his or her personal data and the right to request rectification of any data that are not correct or complete. Reasonable steps will be taken to respond to such a request, taking into consideration the nature of the request, the conditions of the study, and the applicable laws and regulations.

COMMITTEES STRUCTURE

An Independent Scientific Advisory Committee comprised of experts in the management of patients with HF and sponsor representatives will be commissioned for this study. Committee membership and responsibilities will be documented in the committee charter(s).

PUBLICATION POLICY/DISSEMINATION OF CLINICAL STUDY DATA

All information, including but not limited to information regarding JNJ-28431754 (canagliflozin) or the sponsor's operations (eg, patent application, formulas, manufacturing processes, basic scientific data, prior clinical data, formulation information) supplied by the sponsor to the investigator and not previously published, and any data, generated as a result of this study, are considered confidential and remain the sole property of the sponsor. The virtual principal investigator agrees to maintain this information in confidence and use this information only to accomplish this study and will not use it for other purposes without the sponsor's prior written consent.

The virtual principal investigator understands that the information developed in the study will be used by the sponsor in connection with the continued development of JNJ-28431754 (canagliflozin), and thus may be disclosed as required to other clinical investigators or regulatory agencies. To permit the information derived from the clinical studies to be used, the virtual principal investigator is obligated to provide the sponsor with all data obtained in the study.

The results of the study will be reported in a Clinical Study Report generated by the sponsor and will contain data as per protocol. Recruitment performance or specific expertise related to the nature and the key assessment parameters of the study will be used to determine a coordinating investigator for the study, if appropriate.

Study participant identifiers will not be used in publication of results. Any work created in connection with performance of the study and contained in the data that can benefit from copyright protection (except any publication by the investigator as provided for below) shall be the property of the sponsor as author and owner of copyright in such work.

Consistent with Good Publication Practices and International Committee of Medical Journal Editors guidelines, the sponsor shall have the right to publish such primary (multicenter) data and information without approval from the virtual principal investigator. The virtual principal investigator has the right to publish data after the primary data are published. If the virtual principal investigator wishes to publish information from the study, a copy of the manuscript must be provided to the sponsor for review at least 60 days before submission for publication or presentation. Expedited reviews will be arranged for abstracts, poster presentations, or other materials. If requested by the sponsor in writing, the investigator will withhold such publication for up to an additional 60 days to allow for filing of a patent application. In the event that issues arise regarding scientific integrity or regulatory compliance, the sponsor will review these issues with the investigator. The sponsor will not mandate modifications to scientific content and does not have the right to suppress information. For multicenter study designs and substudy approaches, secondary results generally should not be published before the primary endpoints of a study have been published. Similarly, the principal investigator will recognize the integrity of the study by not submitting data for publication data until the results from the completed study have been submitted for publication, within 18 months after the study end date. Authorship of publications resulting from this study will be based on the guidelines on authorship, such as those described in the International Committee of Medical Journal Editors Recommendations for the Conduct, Reporting, Editing and Publication of Scholarly Work in Medical Journals, which state that the named authors must have made a significant contribution to the conception or design of the work; or the acquisition, analysis, or interpretation of the data for the work; and drafted the work or revised it critically for important intellectual content; and given final approval of the version to be published; and agreed to be accountable for all aspects of the work in ensuring that questions related to the accuracy or integrity of any part of the work are appropriately investigated and resolved.

Registration of Clinical Studies and Disclosure of Results

The sponsor will register and disclose the existence and the results of clinical studies as required by law.

DATA QUALITY ASSURANCE

SOURCE DOCUMENTS

At a minimum, source documents consistent in the type and level of detail with that commonly recorded as a basis for standard medical care must be available for the following: participant identification, eligibility, and study identification; record of all adverse events and follow-up of adverse events; and date of study completion and reason for early discontinuation of study intervention or withdrawal from the study, if applicable. Many of the assessments in this study (date of signed informed consent, activity data, KCCQ data) will be collected directly from the participant and therefore the data received is from a direct source.

The author of an entry in the source documents should be identifiable.

Specific details required as source data for the study and source data collection methods will be reviewed with the virtual principal investigator or designee before the study.

Inclusion and exclusion criteria not requiring documented medical history must be verified at a minimum by participant interview or other protocol required assessment (eg, baseline KCCQ) and documented in the source documents.

RECORD RETENTION

In compliance with the ICH/GCP guidelines, the virtual principal investigator/call center will maintain all source documents that support the data collected from each participant, as well as all study documents as specified in ICH/GCP Section 8, Essential Documents for the Conduct of a Clinical Trial, and all study documents as specified by the applicable regulatory requirement(s). The virtual principal investigator/call center will take measures to prevent accidental or premature destruction of these documents.

Essential documents must be retained until at least 2 years after the last approval of a marketing application in an ICH region and until there are no pending or contemplated marketing applications in an ICH region or until at least 2 years have elapsed since the formal discontinuation of clinical development of the investigational product. These documents will be retained for a longer period if required by the applicable regulatory requirements or by an agreement with the sponsor. It is the responsibility of the sponsor to inform the virtual principal investigator/call center as to when these documents no longer need to be retained.

If the virtual principal investigator retires, relocates, or for other reasons withdraws from the responsibility of keeping the study records, custody must be transferred to a person who will accept the responsibility. The sponsor must be notified in writing of the name and address of the new custodian. Under no circumstance shall the virtual principal investigator relocate or dispose of any study documents before having obtained written approval from the sponsor.

If it becomes necessary for the sponsor or the appropriate regulatory authority to review any documentation relating to this study, the virtual principal investigator/call center must permit access to such reports.

STUDY AND SITE CLOSURE

Study Termination

The sponsor reserves the right to terminate the study at any time for any reason at the sole discretion of the sponsor.

10.3 Appendix 3: Kansas City Cardiomyopathy Questionnaire (KCCQ)

Appendix 3 provides a representative example of the scale questions that will be used in this study.

The KC Cardiomyopathy Questionnaire

The following questions refer to your **heart failure** and how it may affect your life. Please read and complete the following questions. There are no right or wrong answers. Please mark the answer that best applies to you.

1. **Heart failure** affects different people in different ways. Some feel shortness of breath while others feel fatigue. Please indicate how much you are limited by **heart failure** (shortness of breath or fatigue) in your ability to do the following activities over the past 2 weeks.

Place an **X** in one box on each line

**Activity**	**Extremely Limited**	**Quite a bit Limited**	**Moderately Limited**	**Slightly Limited**	**Not at all Limited**	**Limited for other reasons or did not do the activity**
Dressing yourself	❑	❑	❑	❑	❑	❑
Showering/Bathing	❑	❑	❑	❑	❑	❑
Walking 1 block on level ground	❑	❑	❑	❑	❑	❑
Doing yardwork, housework or carrying groceries	❑	❑	❑	❑	❑	❑
Climbing a flight of stairs without stopping	❑	❑	❑	❑	❑	❑
Hurrying or jogging (as if to catch a bus)	❑	❑	❑	❑	❑	❑

2. Compared with 2 weeks ago, have your symptoms of **heart failure** (shortness of breath, fatigue, or ankle swelling) changed?

My symptoms of **heart failure** have become…

Much worse	Slightly worse	Not changed	Slightly better	Much better	I've had no symptoms over the last 2 weeks
❑	❑	❑	❑	❑	❑

Copyright ©1992 –2005 John Spertus, MD, MPH Original US English

3. Over the past 2 weeks, how many times did you have **swelling** in your feet, ankles or legs when you woke up in the morning?

Every morning	3 or more times a week, but not every day	1-2 times a week	Less than once a week	Never over the past 2 weeks
❑	❑	❑	❑	❑

4. Over the past 2 weeks, how much has **swelling** in your feet, ankles or legs bothered you?

It has been ...

**Extremely** bothersome	**Quite a bit** bothersome	**Moderately** bothersome	**Slightly** bothersome	**Not at all** bothersome	I've had **no swelling**
❑	❑	❑	❑	❑	❑

5. Over the past 2 weeks, on average, how many times has **fatigue** limited your ability to do what you want?

All of the time	Several times per day	At least once a day	3 or more times per week but not every day	1-2 times per week	Less than once a week	Never over the past 2 weeks
❑	❑	❑	❑	❑	❑	❑

6. Over the past 2 weeks, how much has your **fatigue** bothered you?

It has been ...

**Extremely** bothersome	**Quite a bit** bothersome	**Moderately** bothersome	**Slightly** bothersome	**Not at all** bothersome	I've had **no fatigue**
❑	❑	❑	❑	❑	❑

7. Over the past 2 weeks, on average, how many times has **shortness of breath** limited your ability to do what you wanted?

All of the time	Several times per day	At least once a day	3 or more times per week but not every day	1-2 times per week	Less than once a week	Never over the past 2 weeks
❑	❑	❑	❑	❑	❑	❑

Copyright ©1992 –2005 John Spertus, MD, MPH Original US English

8. Over the past 2 weeks, how much has your **shortness of breath** bothered you?

It has been ...

**Extremely** bothersome	**Quite a bit** bothersome	**Moderately** bothersome	**Slightly** bothersome	**Not at all** bothersome	I've had **no shortness of breath**
❑	❑	❑	❑	❑	❑

9. Over the past 2 weeks, on average, how many times have you been forced to sleep sitting up in a chair or with at least 3 pillows to prop you up because of **shortness of breath**?

Every night	3 or more times a week, but not every day	1-2 times a week	Less than once a week	Never over the past 2 weeks
❑	❑	❑	❑	❑

10. **Heart failure** symptoms can worsen for a number of reasons. How sure are you that you know what to do, or whom to call, if your **heart failure** gets worse?

**Not at all** sure	**Not very** sure	**Somewhat** sure	**Mostly** sure	**Completely** sure
❑	❑	❑	❑	❑

11. How well do you understand what things you are able to do to keep your **heart failure** symptoms from getting worse? (for example, weighing yourself, eating a low salt diet etc.)

Do not understand at all	Do not understand very well	Somewhat understand	Mostly understand	Completely understand
❑	❑	❑	❑	❑

12. Over the past 2 weeks, how much has your **heart failure** limited your enjoyment of life?

It has **extremely** limited my enjoyment of life	It has limited my enjoyment of life **quite a bit**	It has **moderately** limited my enjoyment of life	It has **slightly** limited my enjoyment of life	It has **not limited** my enjoyment of life at all
❑	❑	❑	❑	❑

13. If you had to spend the rest of your life with your **heart failure** the way it is right now, how would you feel about this?

Not at all satisfied	Mostly dissatisfied	Somewhat satisfied	Mostly satisfied	Completely satisfied
❑	❑	❑	❑	❑

Copyright ©1992 –2005 John Spertus, MD, MPH

Original US English

14. Over the past 2 weeks, how often have you felt discouraged or down in the dumps because of your **heart failure**?

I felt that way **all of the time**	I felt that way **most of the time**	I **occasionally** felt that way	I **rarely** felt that way	I **never** felt that way
❑	❑	❑	❑	❑

15. How much does your **heart failure** affect your lifestyle? Please indicate how your **heart failure** may have limited your participation in the following activities over the past 2 weeks.

Please place an **X** in one box on each line

**Activity**	**Severely** limited	Limited **quite a bit**	**Moderately** limited	**Slightly** limited	**Did not** limit at all	Does not apply or did not do for other reasons
Hobbies, recreational activities	❑	❑	❑	❑	❑	❑
Working or doing household chores	❑	❑	❑	❑	❑	❑
Visiting family or friends out of your home	❑	❑	❑	❑	❑	❑
Intimate relationships with loved ones	❑	❑	❑	❑	❑	❑

Copyright ©1992 –2005 John Spertus, MD, MPH Original US English

10.4 Appendix 4: Patient Global Impression of Change (PGIC)

Appendix 4 provides a representative example of the scale questions that will be used in this study.

Since the start of the treatment you've received in this study, your heart failure symptoms are: (select one response)

1. Very much improved
2. Somewhat improved
3. A little improved
4. No change
5. A little worse
6. Somewhat worse
7. Very much worse

10.5 Appendix 5: Patient Global Impression of Severity (PGI-S)

Appendix 5 provides a representative example of the scale question that will be used in this study.

Considering all aspects of your heart failure symptoms right now, would you say your heart failure symptoms are: (select one response)

1. None
2. Mild
3. Moderate
4. Severe
5. Very Severe

10.6 Appendix 6: Participant Satisfaction Survey

Appendix 6 provides a representative example of the questions that will be provided to the participant.

For each of the statements below, please indicate your level of agreement:	Strongly Disagree 1	Disagree 2	Neutral 3	Agree 4	Strongly Agree 5
Trying something new to possibly treat my heart failure was important to me.					
The virtual study was easy for me to sign up for and get started.					
The Fitbit was easy to set up and use.					
The study app was easy to set up on my smartphone.					
The questionnaires were easy to complete on my smartphone.					
The reminders to complete the questionnaires were helpful.					
The study Fitbit provided helpful information to me.					
It was easy to reach the study team when I had questions or concerns.					
I would recommend participating in a study like this to my friends and family.					

10.7 Appendix 7: Adverse Events: Definitions and Procedures for Recording, Evaluating, Follow-up, and Reporting

Adverse Event

An adverse event is any untoward medical occurrence in a clinical study participant administered a medicinal (investigational or non-investigational) product. An adverse event does not necessarily have a causal relationship with the intervention. An adverse event can therefore be any unfavorable and unintended sign (including an abnormal finding), symptom, or disease temporally associated with the use of a medicinal (investigational or non-investigational) product, whether or not related to that medicinal (investigational or non-investigational) product. (Definition per International Conference on Harmonisation [ICH]).

This includes any occurrence that is new in onset or aggravated in severity or frequency from the baseline condition, or abnormal results of diagnostic procedures, including laboratory test abnormalities.

Note: The sponsor collects adverse events starting with the signing of the ICF (refer to All Adverse Events under Section 8.3.1, Time Period and Frequency for Collecting Adverse Events and Serious Adverse Events Information, for time of last adverse event recording).

Serious Adverse Event

A serious adverse event based on ICH and EU Guidelines on Pharmacovigilance for Medicinal Products for Human Use is any untoward medical occurrence that at any dose:

- results in death
- is life-threatening
  (The participant was at risk of death at the time of the event. It does not refer to an event that hypothetically might have caused death if it were more severe.)
- requires inpatient hospitalization or prolongation of existing hospitalization
- results in persistent or significant disability/incapacity
- is a congenital anomaly/birth defect
- is a suspected transmission of any infectious agent via a medicinal product.

*Medical and scientific judgment should be exercised in deciding whether expedited reporting is also appropriate in other situations, such as important medical events that may not be immediately life-threatening or result in death or hospitalization but may jeopardize the participant or may require intervention to prevent one of the other outcomes listed in the definition above. These should usually be considered serious.

If a serious and unexpected adverse event occurs for which there is evidence suggesting a causal relationship between the study intervention and the event (eg, death from anaphylaxis), the event must be reported as a serious and unexpected suspected adverse reaction even if it is a component of the study endpoint (eg, all-cause mortality).

Unlisted (Unexpected) Adverse Event/Reference Safety Information

An adverse event is considered unlisted if the nature or severity is not consistent with the applicable product reference safety information. For JNJ-28431754 (canagliflozin), the expectedness of an adverse event will be determined by whether or not it is listed in the IB.

ATTRIBUTION DEFINITIONS

Assessment of Causality

The causal relationship to study treatment is determined by Janssen Global Medical Safety (GMS). The following selection should be used to assess all adverse events (AE).

Related

There is a reasonable causal relationship between study treatment administration and the AE.

Not Related

There is not a reasonable causal relationship between study treatment administration and the AE.

The term "reasonable causal relationship" means there is evidence to support a causal relationship.

SEVERITY CRITERIA

An assessment of severity grade will be made using the following general categorical descriptors:

Mild: Awareness of symptoms that are easily tolerated, causing minimal discomfort and not interfering with everyday activities.

Moderate: Sufficient discomfort is present to cause interference with normal activity.

Severe: Extreme distress, causing significant impairment of functioning or incapacitation. Prevents normal everyday activities.

SPECIAL REPORTING SITUATIONS

Safety events of interest on a sponsor study intervention in an interventional study that may require expedited reporting or safety evaluation include, but are not limited to:

- overdose of a sponsor study intervention
- suspected abuse/misuse of a sponsor study intervention
- accidental or occupational exposure to a sponsor study intervention
- any failure of expected pharmacologic action (ie, lack of effect) of a sponsor study intervention
- unexpected therapeutic or clinical benefit from use of a sponsor study intervention
- medication error, intercepted medication error, or potential medication error involving a Johnson & Johnson medicinal product (with or without patient exposure to the Johnson &

Johnson medicinal product, eg, product name confusion, product label confusion, intercepted prescribing or dispensing errors)

- exposure to a sponsor study intervention from breastfeeding.

Special reporting situations should be reported. Any special reporting situation that meets the criteria of a serious adverse event should also be reported.

PROCEDURES

All Adverse Events

Adverse events, regardless of seriousness, severity, or presumed relationship to study intervention, must be recorded using medical terminology on the solicited AE form. Whenever possible, diagnoses should be given when signs and symptoms are due to a common etiology (eg, cough, runny nose, sneezing, sore throat, and head congestion should be reported as "upper respiratory infection").

For all studies with an outpatient phase, including open-label studies, the participant must be provided with a "wallet (study) card" and instructed to carry this card with them for the duration of the study indicating the following:

- study number
- statement, in the local language(s), that the participant is participating in a clinical study
- call center name and 24-hour contact telephone number
- site number
- participant number
- any other information that is required to do an emergency breaking of the blind.

Serious Adverse Events

All serious adverse events that have not resolved by the end of the study, or that have not resolved upon discontinuation of the participant's participation in the study, must be followed until any of the following occurs:

- the event resolves
- the event stabilizes
- the event returns to baseline, if a baseline value/status is available
- the event can be attributed to agents other than the study intervention or to factors unrelated to study conduct
- it becomes unlikely that any additional information can be obtained (participant or health care practitioner refusal to provide additional information, lost to follow-up after demonstration of due diligence with follow-up efforts)

- suspected transmission of an infectious agent by a medicinal product will be reported as a serious adverse event. Any event requiring hospitalization (or prolongation of hospitalization) that occurs during the course of a participant's participation in a study must be reported as a serious adverse event, except hospitalizations for the following:
- hospitalizations not intended to treat an acute illness or adverse event (eg, social reasons such as pending placement in long-term care facility)
- surgery or procedure planned before entry into the study (must be documented). Note: hospitalizations that were planned before the signing of the ICF, and where the underlying condition for which the hospitalization was planned has not worsened, will not be considered serious adverse events. Any adverse event that results in a prolongation of the originally planned hospitalization is to be reported as a new serious adverse event.

The cause of death of a participant in a study within 24 hours of the last dose of study intervention, whether or not the event is expected or associated with the study intervention, is considered a serious adverse event.

CONTACTING SPONSOR REGARDING SAFETY

The names (and corresponding telephone numbers) of the individuals who should be contacted regarding safety issues or questions regarding the study are listed in the Contact Information page(s), which will be provided as a separate document.

PRODUCT QUALITY COMPLAINT HANDLING

A product quality complaint (PQC) is defined as any suspicion of a product defect related to manufacturing, labeling, or packaging, ie, any dissatisfaction relative to the identity, quality, durability, or reliability of a product, including its labeling or package integrity. A PQC may have an impact on the safety and efficacy of the product. Timely, accurate, and complete reporting and analysis of PQC information from studies are crucial for the protection of participants, investigators, and the sponsor, and are mandated by regulatory agencies worldwide. The sponsor has established procedures in conformity with regulatory requirements worldwide to ensure appropriate reporting of PQC information; all studies conducted by the sponsor or its affiliates will be conducted in accordance with those procedures.

Procedures

All initial PQCs must be reported by the call center to the sponsor within 24 hours after being made aware of the event.

If the product defect is combined with a serious adverse event, the call center must report the PQC to the sponsor according to the serious adverse event reporting timelines (refer to Section 8.3.1, Time Period and Frequency for Collecting Adverse Event and Serious Adverse Event Information). A sample of the suspected product should be maintained for further investigation if requested by the sponsor.

Contacting Sponsor Regarding Product Quality

The names (and corresponding telephone numbers) of the individuals who should be contacted regarding product quality issues are listed in the Contact Information page(s), which will be provided as a separate document.

10.8 Appendix 8: Protocol Amendment History

The Protocol Amendment Summary of Changes Table for the current amendment is located directly before the Table of Contents (TOC).

PROTOCOL AMENDMENT SUMMARY OF CHANGES TABLE

**DOCUMENT HISTORY**	
**Document**	**Date**
Amendment 2	02 June 2020
Amendment 1	07 February 2020
Original Protocol	07 November 2019

Amendment 2 (02 June 2020)

Overall Rationale for the Amendment: The overall reasons for the amendment are to 1) clarify inclusion/exclusion criteria for recruitment centers, 2) more accurately identify patients with heart failure in inclusion/exclusion criteria, and 3) represent patients with heart failure in medical claims over time and between sites of care.

Section Number and Name	Description of Change	Brief Rationale
1.1. Synopsis (Objectives and Endpoints, Overall Design, Secondary and Exploratory Evaluations, Statistical Methods);<br>1.3. Schedule of Activities (SoA);<br>3. Objectives and Endpoints;<br>4.1. Overall Design;<br>8.1. Effectiveness Assessments;<br>9.4.2.2. Exploratory Analyses	The term 'stairs' was changed to 'floors'.	To align with the data obtained from the Fitbit device, which reports this metric as 'floors' climbed not 'stairs' climbed.
1.1. Synopsis (Adverse Event Data Collection, Adverse Event Evaluations);<br>3. Objectives and Endpoints	The word 'self-reported' was replaced by 'adverse' and 'date of the first dose of study intervention' was replaced by 'time of informed consent'.	To ensure consistency for adverse event (AE) collection and timepoints across the protocol.
1.1. Synopsis (Adverse Event Data Collection, Adverse Event Evaluations);<br>3. Objectives and Endpoints	The word 'adverse event' was added with respect to medical claims data.	To clarify that medical claims data include AE data.
1.1. Synopsis (Overall Design);<br>1.3. Schedule of Activities (SoA);<br>4.1. Overall Design;<br>4.2. Scientific Rationale for Study Design;<br>5.1. Inclusion Criteria (Criterion #4)	The upper threshold of the Kansas City Cardiomyopathy Questionnaire (KCCQ) for baseline overall summary score was changed from <80 to ≤80.	To include participants with KCCQ summary score of 80.
1.1. Synopsis (Overall Design);<br>1.3. Schedule of Activities (SoA);<br>4.1. Overall Design;<br>4.2. Scientific Rationale for Study Design;	The lower threshold (<40) of the KCCQ for baseline overall summary score was removed.	To ensure that a larger number of participants have an opportunity to experience measurable health status benefit from treatment as there is no *a*

**Section Number and Name**	**Description of Change**	**Brief Rationale**
5.1. Inclusion Criteria (Criterion #4)		*priori* reason to presume that more symptomatic participants can't benefit from treatment.
4.2. Scientific Rationale for Study Design	The participant eligibility was extended to include those with New York Heart Association (NYHA) Class IV.	
1.1. Synopsis (Overall Design);<br>4.1. Overall Design	'Dates of deaths' was added to the list of study assessments.	To correlate with the exploratory endpoint 'time to death' and also for consistency.
1.1. Synopsis (Secondary and Exploratory Evaluations, Statistical Methods);<br>9.4.2.2. Exploratory Analyses	The 'time to death' evaluation was added to the list of secondary and exploratory evaluations.	To maintain consistency across the protocol. The term was omitted by mistake in the previous versions.
1.1. Synopsis (Intervention Groups and Duration);<br>4.1. Overall Design	The term 'claims' was deleted from collection of data during the no treatment period.	To explain the wider scope of data collection during the no treatment period (not just claims data).
1.1. Synopsis (Adverse Event Analyses);<br>8.3. Adverse Events and Serious Adverse Events and Special Situations;<br>9.4.3. Adverse Events Analysis	The term 'self-reported' was added.	To clarify AE analysis process
1.2. Schema	The study schema was updated.	To align with the changes in the study design and Schedule of Activities.
1.3. Schedule of Activities (SoA)	Kept Screening and Baseline in separate columns.	To clarify that Screening and Baseline are 2 distinct periods in this study.
1.3. Schedule of Activities (SoA);<br>4.2. Scientific Rationale for Study Design;<br>5. Study Population	Screening window was increased from 21 days to 28 days.	To increase the screening period from 21 days to 28 days to accommodate various study set up activities like electronic health record (EHR) reviews, app set-up and coordination of consenting, participants discussing with their physicians, families, etc and to keep the 7-day baseline period and specify the baseline activities.
4.2. Scientific Rationale for Study Design (Length of Study Periods)	Provided clarity on requirement of baseline period of 7 days. The text was modified as: The screening period of ~~3 weeks~~ **28 days** allows for an appropriate length of time for screening procedures to determine study eligibility **(with EHR reviews, app set-up, completion of the KCCQ, and consenting). The baseline period of 7 days begins upon randomization and allows for shipment of the study drug and Fitbit device to the participant**.	
2. Introduction;<br>4.2.1. Study-Specific Ethical Design Considerations	Information on Food and Drug Administration approval of dapagliflozin was added.	To explain the dapagliflozin information and to illustrate the benefit of including participants with heart failure with preserved ejection fraction (HFpEF) in this study.

Section Number and Name	Description of Change	Brief Rationale
2.2. Benefit/Risk Assessment	The text 'or for use in patients with HFpEF ' was added.	To indicate that there may be a benefit in HFpEF participants where dapagliflozin doesn't have an indication.
4.1. Overall Design	Specified that inclusion/exclusion criteria, healthcare resource utilization, and relevant concomitant medications will be included in the claims data monitoring.	To maintain consistency with the other sections of the protocol.
4.2. Scientific Rationale for Study Design;	Information of KCCQ was updated.	To update that KCCQ was recently qualified by Center for Drug Evaluation and Research as a patient reported outcome instrument for use in clinical trials in heart failure.
4.2. Scientific Rationale for Study Design;<br>8.1.2. Actigraphy Measurements	Added description on actigraphy measurements.	To add the description on Fitbit data collection that was missed in the previous protocol version.
4.4. End of Study Definition	The following text was modified as: A participant will be considered to have **ended** ~~completed~~ the treatment period of the study (at 3 months), regardless of whether the participant **has completed** ~~is on~~ the study intervention, if he or she has completed assessments at Month 3 of the double-blind treatment period, ~~or~~ withdrew earlier from the treatment, or ~~at the time of death for participants who~~ died prior to the Month 3 ~~visit~~ assessment.	To clarify that the participant could have ended the treatment period but not have completed the study intervention.
4.4. End of Study Definition	The term 'clinical research organization' was added.	To clarify that the study partner prefers to a clinical research organization partner and not participant.
5.1. Inclusion Criteria;<br>5.2. Exclusion Criteria;<br>5.2. Exclusion Criteria (Criteria #1, 3, 5, 6, and 7)	Clarified that each potential participant was required to satisfy the inclusion/exclusion criteria 'prior to enrollment (consent date)'.<br>The extra term 'prior to enrollment ' was deleted from exclusion criteria 1, 3, and 5.<br>The extra term 'of the screening visit' was deleted from exclusion criterion 6.<br>The extra term 'of consent' was deleted from exclusion criterion 7.	To clarify the timing for fulfillment of inclusion/exclusion criteria.
5.1. Inclusion Criteria (Criterion #3)	Definitions of heart failure with reduced ejection fraction (HFrEF) and HFpEF were updated. Added spironolactone and eplerenone to the definition of HFpEF.	To clarify and align with the information received from site principal investigators and Steering Committee on definitions of HFrEF and HFpEF for this virtual study and diagnosis confirmation within 18 months instead of 1 year.

Section Number and Name	Description of Change	Brief Rationale
5.2. Exclusion Criteria (Criterion #4)	The text 'from the most recent assessment' was added.	To clarify the timing of assessment of stage 4 or 5 chronic kidney disease for exclusion of participants from the study.
5.2. Exclusion Criteria (Criterion #8)	The text 'any condition for which, in the opinion of the investigator, participation would not be in the best interest of the participant (eg, compromise the well-being) or that could prevent, limit, or confound the protocol-specified assessments' was added.	To explicitly describe the exclusion of participants with compromised well-being or whose participation could prevent, limit, or confound the protocol-specified assessments.
5.2. Exclusion Criteria (Criterion #12)	The text 'receiving an investigational study medication' was added.	To clarify that participants with ongoing treatment with an investigational agent should be excluded from the study, but those in studies without an investigational agent are eligible to participate.
5.2. Exclusion Criteria (Criterion #13)	A new exclusion criterion #13 was added: 'has a left ventricular assist device'.	To exclude the factors that can alter the health status and would confound the ability to define a benefit from treatment with the study drug.
5.2. Exclusion Criteria (Criterion #14)	A new exclusion criterion # 14 was added: 'patient identity or association with enrolling network cannot be verified'.	To exclude those participants whose identity cannot be verified.
5.4. Screen Failures	Updated the criteria for rescreening of participants.	To allow and outline for the rescreening of participants.
5.4. Screen Failures	The following text was modified as: All potential participants who ~~electronically sign the remote e-consent~~ **opt in to participate in the study on the website** will receive a unique identifier in the study database held by a third-party representative.	To emphasize that the participants will receive a unique study number when they opt in to participate in the study which is prior to enrollment (signing of consent).
6.3. Measures to Minimize Bias: Randomization and Blinding	The following text was modified as: The IxR will provide the kit number of the study intervention bottle(s) to be dispensed for each randomly assigned participant **to begin dosing** on Day 1 of the double-blind treatment period.	To indicate that the kit numbers are not assigned on Day 1 but that study intervention dosing should begin on Day 1.
6.4. Study Intervention Compliance	Re-education of the participant on importance of dairy completion was added.	To indicate that diary should be completed to track the study intervention compliance data.
7.2. Participant Discontinuation/Withdrawal from the Study	The following text was modified as: If ~~applicable~~, **the participant is lost to follow-up**, vital status **will still be attempted** ~~to be obtained~~ **for collection** at study end through the participant's physician, medical claims, or	To specify that vital status should be obtained, if possible, in case participant is lost to follow-up.

Section Number and Name	Description of Change	Brief Rationale
	public information according to local guidelines and as allowed by local regulations	
7.2. Participant Discontinuation/Withdrawal from the Study	The information on home and work contact numbers were removed.	To indicate that the app can only store smartphone contact number.
7.2. Participant Discontinuation/Withdrawal from the Study	The following text was added: Follow-up can also be done with the help of the study site personnel.	To specify that besides the call center, study site can also help with follow-up.
8. Study Assessments and Procedures (Overview)	The following text was deleted: Health Resource Utilization (HRU) and health economics data will be collected. Refer to Section 8.2.4, Healthcare Resource Utilization and Health Economics for details.	To remove duplicate information.
8.2.4. Healthcare resource Utilization and Health Economics	Added 'discharge destination' information to the list of healthcare resource utilization and health economics data	To maintain consistency with text in the other sections of the protocol.
8.3. Adverse Events and Serious Adverse Events and Special Situations	The text was modified to indicate that the principal investigator may contact the treating physician as agreed in the informed consent form.	To update how the principal investigator may contact the participant's treating physician based on the confirmed process.
8.3. Adverse Events and Serious Adverse Events and Special Situations;<br>9.4.3. Adverse Events Analysis	Emergency department visits was added as a criteria for aggregation of AEs collection from claims.	To be consistent by including criteria for aggregation of AEs collection from claims in this section.
8.3.1. Time Period and Frequency for Collecting Adverse Event and Serious Adverse Event Information	The following text was modified as: All ~~participant-reported~~ adverse events ~~suspected to be related to the study intervention~~, whether serious or non-serious, will be collected from the time a signed and dated ICF is obtained until the participant completes their participation in the 3-month treatment period plus 30 days.	To clarify that all AEs (not just those suspected to be related to study intervention) should be collected.
8.3.4. Adverse Events of Interest	Additional adverse events of interest were specified.	To expand the scope of AEs of interest to be collected in the study.
9.3. Populations for Analyses (Table 2)	Updated the definition of 'per protocol' population.	To clarify the definition of per protocol population.
10.2. Appendix 2: Regulatory, Ethical, and Study Oversight Considerations (Regulatory and Ethical Considerations [Protocol Amendments])	The following text was deleted: The data recorded in the source documents will reflect any departure from the protocol, and the source documents will describe this departure and the circumstances requiring it.	To remove non-applicable data source in this virtual study.
10.2. Appendix 2: Regulatory, Ethical, and Study Oversight Considerations (Regulatory and Ethical Considerations [Protocol Amendments])	Specified that the departure from the protocol information will be recorded by the sponsor.	To provide clarity on who will record the departure from protocol information.
10.6. Appendix 6: Participant Satisfaction Survey	Added numeric grading of 1 to 5.	To provide the scoring used on the participant satisfaction survey which was omitted in previous versions.

Section Number and Name	Description of Change	Brief Rationale
10.7. Appendix 7: Adverse Events: Definitions and Procedures for Recording, Evaluating, Follow-up, and Reporting (Product Quality Complaint Handling)	Modified the text to indicate that instead of study site personnel, the call center will report the product quality complaint to the sponsor in case a product defect is combined with serious AE.	To provide clarity and for consistency across the study.
References	New references (#1, 3, 14) were added. Other reference numbers cited within the protocol were updated.	References added to support the study.
6.1. Study Interventions Administered	Assigned table number to ‘Description of Study Interventions’ table. Table numbering of other tables were updated.	Minor errors were noted.
8.3.3. Pregnancy	The following text was modified as ‘Any participant who becomes pregnant during the study must be promptly ~~withdrawn~~ **discontinued** from the study ~~and discontinue further study~~ intervention’.	
Throughout the protocol	Minor grammatical, formatting, or consistency changes were made.	

Amendment 1 (07 February 2020)

Overall Rationale for the Amendment: The overall reason for the amendment is to remove Return of Results (RoR) to align with Janssen standard operating procedure (SOP), update the study overview schema to align with the protocol, and to define that the Kansas City Cardiomyopathy Questionnaire (KCCQ) overall summary score will be used for the baseline assessment.

Section Number and Name	Description of Change	Brief Rationale
Synopsis Overall Design; 1.3 Schedule of activities (SoA); 4.1. Overall Design; 4.2. Scientific Rationale for Study Design; 5.1. Inclusion Criteria; 8.1.1. KCCQ Scores	Clarified that KCCQ overall summary score will be evaluated at Screening/Baseline to determine participant eligibility and other KCCQ domains and summary scores will be calculated at baseline and subsequent postbaseline timepoints, respectively.	To clarify that KCCQ baseline overall summary score will be used to determine participant eligibility at screening/baseline.
Synopsis Objectives and Endpoints; Synopsis Intervention Groups and Duration; 3. Objectives and Endpoints; 4.1 Overall Design	The term of “Return of Results (RoR)” was removed and term “Participant Unblinding” was added.<br>Text related to RoR including exploratory objective and endpoint was deleted.	Janssen SOP does not allow for “RoR” to participants for an approved drug.
1.2 Schema	Schematic Overview of the Study was replaced with an updated figure (1).	To align with protocol.
1.3 Schedule of activities (SoA)	SoA was updated to clarify study day for date of first dose and other timepoints. Also, footnote “d” related to claims data collection and reporting of adverse events were updated.	To specify the timepoints (in days) for clarity and added a baseline period of up to 7 days before Day 1.
Title Page	EudraCT number deleted	EudraCT number is not applicable for the current study.
6.3 Measures to Minimize Bias: Randomization and Blinding	Information related to blind breaking was updated. Clarified that “Study Responsible Physician “or “other sponsor physician” designee may in an emergency determine the	To update appropriate process for study unblinding.

Section Number and Name	Description of Change	Brief Rationale
	identity of the study intervention by contacting the IxR.	
9.4.3 Adverse event analysis	Clarified that adverse events reported from call center will be reported separately from those obtained from claims data.	To clarify the adverse event reporting
10.7 Appendix 7: Adverse Events: Definitions and Procedures for Recording, Evaluating, Follow-up, and Reporting	Clarified that the causal relationship to the study treatment is determined by Janssen Global Medical Safety.<br>Text related to responsibility of virtual principal investigator was deleted.	To align with the protocol and the design of the study and to remove incorrect text.<br>Clarified the responsibility of virtual principal investigator.
Throughout the protocol	Minor grammatical, formatting, or spelling changes were made.	Minor errors were noted.

11. REFERENCES

1. Bertram Pitt, Pfeffer MA, Assmann SF, et al. Spironolactone for Heart Failure with Preserved Ejection Fraction. N Engl J Med. 2014;370:1383-1392.
2. Butler J (2017), Hamo CE, Filippatos G, et al. The potential role and rationale for treatment of heart failure with sodium-glucose co-transporter 2 inhibitors. Eur J Heart Fail. 2017;19(11):1390-1400.
3. Yancy CW, Jessup M, Bozkurt B, et al. 2017 ACC/AHA/HFSA Focused Update of the 2013 ACCF/AHA Guideline for the Management of Heart Failure. JACC. 2017;70(6):776-803.
4. DDT COA #000084: Kansas City Cardiomyopathy Questionnaire (KCCQ). https://www.fda.gov/drugs/ddt-coa-000084-kansas-city-cardiomyopathy-questionnaire-kccq.
5. Dreyer RP (2016), Jones PG, Kutty S, Spertus JA. Quantifying clinical change: discrepancies between patients' and providers' perspectives. Qual Life Res. 2016 Sep;25(9):2213-2220.
6. European Medicines Agency (EMEA) (2012). Guideline on clinical investigation of medicinal products in the treatment or prevention of diabetes mellitus. 14 May 2012; CPMP/EWP/1080/00 Rev. 1. Committee for Medicinal Products for Human Use (CHMP).
7. Food and Drug Administration (FDA) (2008). Guidance for industry: Diabetes Mellitus: Developing drugs and therapeutic biologics for treatment and prevention. U.S. Department of Health and Human Services, Food and Drug Administration, Center for Drug Evaluation and Research (CDER), February 2008, Clinical/Medical.
8. Food and Drug Administration (FDA) (2019). Treatment for heart failure: Endpoints for drug development, Guidance for Industry. Draft guidance un 2019; US Department of Health and Human Service, Food and Drug Administration.
9. Gail M (1985), Simon R. Testing for qualitative interactions between treatment effects and patient subsets. Biometrics. 1985 Jun;41(2):361-372.
10. Green CP (2000), Porter CB, Bresnahan DR, et al. Development and evaluation of the Kansas City Cardiomyopathy Questionnaire: A new health status measure for heart failure. J Am Coll Cardiol 2000;35:1245-1255.
11. Investigator's Brochure: JNJ-28431754 (Canagliflozin) Edition 15. Janssen Research and Development, LLC (9 Nov 2017).
12. Joseph SM (2013), Novak E, Arnold SV, et al. Comparable performance of the Kansas City Cardiomyopathy Questionnaire in patients with heart failure with preserved and reduced ejection fraction Circ Heart Fail. 2013 Nov;6(6):1139-1146.
13. Kelkar AA (2016), Spertus J, Pang P, et al. Utility of patient-reported outcome instruments in heart failure. JACC Heart Failure 2016;4(3):165-175.
14. Mahaffey KW (2019), Jardine MJ, Bompoint S, et al. Canagliflozin and cardiovascular and renal outcomes in type 2 diabetes and chronic kidney disease in primary and secondary cardiovascular prevention groups: results from the randomized CREDENCE trial. Circulation. 2019 Aug 27;140(9):739-750.
15. Mahaffey KW (2018), Neal B, Perkovic B, et al. Canagliflozin for primary and secondary prevention of cardiovascular events: results from the CANVAS program (Canagliflozin Cardiovascular Assessment Study). Circulation. 2018 Jan 23;137(4):323-334.
16. McMurray JJV (2019), Solomon SD, Inzucchi SE, et al; DAPA-HF Trial Committees and Investigators. Dapagliflozin in patients with heart failure and reduced ejection fraction. N Engl J Med. 2019 Sep 19.
17. Medical Device Development Tools Program (2016). Medical Development Tool Qualification Decision Summary for Kansas City Cardiomyopathy Questionnaire (KCCQ). Available from: https://www.fda.gov/downloads/MedicalDevices/ScienceandResearch/MedicalDeviceDevelopmentToolsMDDT/UCM581761.pdf. Accessed on 13 November 2017.
18. Monami M (2014), Dicembrini I, Mannucci E. Dipeptidyl peptidase-4 inhibitors and heart failure: a meta-analysis of randomized clinical trials. Nutr Metab Cardiovasc Dis. 2014;24(7):689-697.

19. Mosenzon O (2019), Wiviott SD, Cahn A, et. al. Effects of dapagliflozin on development and progression of kidney disease in patients with type 2 diabetes: an analysis from the DECLARE-TIMI 58 randomised trial. Lancet Diabetes Endocrinol. 2019 Aug;7(8):606-617.

20. Nassif ME (2019), Kosiborod M. Effects of sodium-sodglucose cotransporter type 2 inhibitors in heart failure. Diabetes Obes Metab. 2019;21(Suppl. 2):19-23.

21. Nassif ME (2019), Windsor SL, Tang F, et al. Dapagliflozin Effects on Biomarkers, Symptoms, and Functional Status in Patients With Heart Failure With Reduced Ejection Fraction: The DEFINE-HF Trial. Circulation. 2019 Oct 29;140(18):1463-1476.

22. National Kidney Foundation (2012). KDOQI Clinical Practice Guideline for Diabetes and CKD: 2012 Update. Am J Kidney DIS. 2012;60(5):850-886.

23. Nichols GA (2001), Hillier TA, Erbey JR, Brown JB. Congestive heart failure in type 2 diabetes: prevalence, incidence, and risk factors. Diabetes Care. 2001;24(9):1614-1619.

24. Nichols GA (2004), Gullion CM, Koro CE, Ephross SA, Brown JB. The incidence of congestive heart failure in type 2 diabetes. Diabetes Care. 2004;27(8):1879-1884.

25. Packer M (2017), Anker SD, Butler J, Filippatos G, Zannad F. Effects of sodium-glucose cotransporter 2 inhibitors for the treatment of patients with heart failure: proposal of a novel mechanism of action. JAMA Cardiol. 2017;2(9):1025-1029.

26. Radholm K (2018), Figtree G, Perkovic V, et al. Canagliflozin and Heart Failure in Type 2 Diabetes Mellitus. Results From the CANVAS Program. Circ. 2018;138:458-468.

27. Ryan PB (2018), Buse JB, Schuemie MJ, et al. Comparative effectiveness of canagliflozin, SGLT2 inhibitors and non-SGLT2 inhibitors on the risk of hospitalization for heart failure and amputation in patients with type 2 diabetes mellitus: A real-world meta-analysis of 4 observational databases (OBSERVE-4D). Diabetes Obes Metab. 2018;20(11):2585-2597.

28. Sezai A (2019), Sekino H, Unosawa S, et al. Canagliflozin for Japanese patients with chronic heart failure and type II diabetes. Cardiovasc Diabetol. (2019) Jun 5;18(1):76.

29. Spertus J (2005), Peterson E, Conard MW, et al. Monitoring clinical changes in patients with heart failure: a comparison of methods. Am Heart J 2005;150(4):707-715.

30. Zinman B (2015), Wanner C, Lachin JM, et. al. Empagliflozin, cardiovascular outcomes and mortality in type 2 diabetes. N Engl J Med 2015;373:2117-2128.

INVESTIGATOR AGREEMENT

JNJ-28431754 (canagliflozin) (INVOKANA)
Clinical Protocol 28431754HFA3002 Amendment 3

INVESTIGATOR AGREEMENT

I have read this protocol and agree that it contains all necessary details for carrying out this study. I will conduct the study as outlined herein and will complete the study within the time designated.

I will provide copies of the protocol and all pertinent information to all individuals responsible to me who assist in the conduct of this study. I will discuss this material with them to ensure that they are fully informed regarding the study intervention, the conduct of the study, and the obligations of confidentiality.

Coordinating Investigator (where required):

Name (typed or printed): ______________________

Institution and Address: ______________________

Signature: ______________________ Date: ______________________
(Day Month Year)

Principal (Site) Investigator:

Name (typed or printed): ______________________

Institution and Address: ______________________

Telephone Number: ______________________

Signature: ______________________ Date: ______________________
(Day Month Year)

Sponsor's Responsible Medical Officer:

Name (typed or printed): John Whang, MD, FACC

Institution: Janssen Scientific Affairs

Signature: [signature] Date: 24 February 2021
(Day Month Year)

Note: If the address or telephone number of the investigator changes during the course of the study, written notification will be provided by the investigator to the sponsor, and a protocol amendment will not be required.

Summary Of Changes in Statistical Analysis Plan

Canagliflozin: Impact on Health Status, Quality of Life, and Functional Status in Heart Failure
CHIEF-HF

Protocol 28431754HFA3002; Phase 3B

PROTOCOL AMENDMENT SUMMARY OF CHANGES TABLE

MAJOR CHANGES – MINOR AND OTHER CLARIFICATIONS ALL LISTED WITHIN EACH PROTOCOL AMENDMENT

**DOCUMENT HISTORY**	
**Document**	**Date**
Original Protocol	07 November 2019
Amendment 1	07 February 2020
	To remove Return of Results (RoR) to align with Janssen standard operating procedure (SOP), update the study overview schema to align with the protocol, and to define that the Kansas City Cardiomyopathy Questionnaire (KCCQ) overall summary score (OSS) is used for the baseline assessment.
Amendment 2	02 June 2020
	1) updated inclusion/exclusion criteria – removed the lower threshold of the KCCQ OSS to also include those with scores <40, added spironolactone or eplerenone as a diuretic for inclusion for HFpEF patients, increased diagnosis confirmation of HF from 12 months to 18 months.<br><br>2) specified that vital status to be obtained if participant is lost to follow-up (LTFU)
Amendment 3	23 February 2021
	Clarified and made consistent the adverse event (AE) reporting requirements and time frames for the study.

The original sample size was to include 1,900 randomized participants, but shifting priorities of the sponsors (Janssen Scientific Affairs and PRA Health Sciences), led to closing the study to enrollment on February 1, 2021.

Original Statistical Analysis Plan

Janssen Research & Development, LLC

Statistical Analysis Plan

Canagliflozin: Impact on Health Status, Quality of Life, and Functional Status in Heart Failure)

Protocol 28431754HFA3002; Phase 3B

JNJ-28431754 (canagliflozin) (INVOKANA)

**Status:**	FINAL
**Date:**	10 April 2020
**Prepared by:**	Janssen Research & Development, LLC
**Document No.:**	EDMS-ERI-204506194, 1.0

Compliance: The study described in this report was performed according to the principles of Good Clinical Practice (GCP).

Confidentiality Statement

The information in this document contains trade secrets and commercial information that are privileged or confidential and may not be disclosed unless such disclosure is required by applicable law or regulations. In any event, persons to whom the information is disclosed must be informed that the information is privileged or confidential and may not be further disclosed by them. These restrictions on disclosure will apply equally to all future information supplied to you that is indicated as privileged or confidential.

TABLE OF CONTENTS

TABLE OF CONTENTS **2**

LIST OF IN-TEXT TABLES **3**

LIST OF IN-TEXT FIGURES **3**

AMENDMENT HISTORY **4**

ABBREVIATIONS **4**

1. INTRODUCTION **5**
1.1. Objectives 5
1.2. Study Design 6
1.3. Statistical Hypotheses for Trial Objectives 7
1.4. Sample Size Justification 8
1.5. Randomization and Blinding 8
1.6. Study Data Flow 9

2. GENERAL ANALYSIS DEFINITIONS **10**
2.1. Time Windows 10
2.2. Pooling Algorithm for Analysis Centers 12
2.3. Analysis Sets 12
2.3.1. All Randomized Analysis Set 12
2.3.2. Per Protocol Analysis Set 12
2.3.3. Safety Analysis Set 13
2.4. Definition of Subgroups 13
2.5. Study Day and Relative Day 13
2.6. Baseline, End of Treatment, and End of Study 13
2.7. Imputation Rules for Missing AE Date/Time of Onset/Resolution 14

3. INTERIM ANALYSIS AND DATA MONITORING COMMITTEE REVIEW **14**

4. SUBJECT INFORMATION **14**
4.1. Demographics and Baseline Characteristics 14
4.2. Disposition Information 15
4.3. Treatment Compliance 15
4.4. Extent of Exposure 16
4.5. Protocol Deviations 16
4.6. Prior and Concomitant Medications 17

5. EFFICACY **17**
5.1. Analysis Specifications 17
5.1.1. Level of Significance 17
5.1.2. Data Handling Rules 17
5.2. Primary Efficacy Endpoint 19
5.2.1. Definition 19
5.2.2. Estimand 19
5.2.3. Analysis Methods 19
5.3. Secondary Endpoints 22
5.3.1. Definition 22
5.3.2. Estimand 22
5.3.3. Analysis Methods 23
5.4. Exploratory Variables 23
5.4.1. Definition 23
5.4.2. Analysis Methods 24

6. SAFETY ... 26
6.1. Adverse Events ... 26
6.2. Clinical Laboratory Tests ... 27
6.3. Vital Signs and Physical Examination Findings ... 27
6.4. Electrocardiogram ... 27
6.5. Other Safety Parameters ... 28

7. PHARMACOKINETICS/PHARMACODYNAMICS ... 28

8. BIOMARKERS ... 28

9. HEALTH ECONOMICS ... 28

REFERENCES ... 29

APPENDICES ... 31
Appendix 1: The Kansas City Cardiomyopathy Questionnaire (KCCQ) ... 31
Appendix 2: Participant Satisfaction Survey ... 36
Appendix 3: Schedule of Activities ... 37
Appendix 4: Usage of the Analysis Sets ... 39
Appendix 5: CHIEF-HF Study Data Flow Diagram ... 40
Appendix 6: Patient Global Impression of Change (PGIC) ... 42
Appendix 7: Patient Global Impression of Severity (PGI-S) ... 43
Appendix 8: KCCQ Score Instructions ... 44

LIST OF IN-TEXT TABLES

Table 1: Measurement Windows ... 11
Table 2: Demographic Variables ... 14

LIST OF IN-TEXT FIGURES

Figure 1: Schematic Overview of the Study ... 7

AMENDMENT HISTORY

Not applicable.

ABBREVIATIONS

AE	adverse event
API	application program interface
ANOVA	analysis of variance
CDF	cumulative distribution function
CI	confidence interval
CSR	clinical study report
ED	emergency department
EF	ejection fraction
EHR	electronic health record
EOS	end of study
EOT	end of treatment
ES	effect size
FDA	Food and Drug Administration
HF	heart failure
HFpEF	heart failure preserved ejection fraction
HFrEF	heart failure reduced ejection fraction
HRU	healthcare resource utilization
ICD	International Statistical Classification of Diseases and Related Health Problems
ICH	International Conference on Harmonisation
IHN	integrated health networks
ITT	intent to treat
IxR	interactive web response system
KCCQ	Kansas City Cardiomyopathy Questionnaire
KCCQ-TSS	Kansas City Cardiomyopathy Questionnaire – Total Symptom Score
LOCF	last observation carried forward
LS	least squares
MedDRA	Medical Dictionary for Regulatory Activities
MMRM	mixed model of repeated measurement
MI	multiple imputation
MID	minimum detectable
NYHA	New York Heart Association
PGIC	Patient Global Impression of Change
PGI-S	Patient Global Impression of Severity
PI	principal investigator
PP	per protocol
SAE	serious adverse event
SAP	statistical analysis plan
SAS	Statistical Analysis System
SD	standard deviation
SE	standard error
SGLT2	sodium glucose cotransporter-2 inhibitor
SUSAR	suspected unexpected serious adverse reaction
T2DM	type 2 diabetes mellitus
TEAE	treatment-emergent adverse event
UGT	UDP-Glucuronosyltransferase
US	United States

1. INTRODUCTION

This Statistical Analysis Plan (SAP) describes the planned analysis and reporting for the 28431754HFA3002 **(Canagliflozin: Impact on Health Status, Quality of Life, and Functional Status in Heart Failure – CHIEF-HF)** study. This Phase 3b study is being conducted in the United States (US) in participants with symptomatic HF stratified by HF (reduced ejection fraction [HFrEF] or preserved ejection fraction [HFpEF]) and compares the effectiveness of canagliflozin 100 mg versus placebo for improvement in HF symptoms measured by the Kansas City Cardiomyopathy Questionnaire - Total Symptom Score (KCCQ-TSS) (see Appendix 1: The Kansas City Cardiomyopathy Questionnaire (KCCQ)) after 12 weeks. This document contains details of the relevant definitions, data handling conventions and methods for analyses. The document will be finalized prior to database lock.

This SAP is based on the clinical study protocol (finalized first on 7 November, and subsequently amended on 7 February 2020 (Protocol **28431754HFA3002 Amendment 1**, dated **07 February 2020** - **EDMS-ERI-154820237, version 3.0)** by Janssen Scientific Affairs, LLC) that provides details on the conduct of this study and operational aspects of clinical assessments and timing for completing a participant in this study.

1.1. Objectives

Primary Objective

The primary objective is to determine the superiority of the effectiveness of canagliflozin 100 mg daily versus placebo in participants with symptomatic HF in improving the overall KCCQ-TSS.

Secondary Objectives

The first key secondary objective is:

- to determine the superiority of the effectiveness of canagliflozin 100 mg daily versus placebo in improving the total daily step count.

The second key secondary objective is:

- to determine the superiority of the effectiveness of canagliflozin 100 mg daily versus placebo in improving the KCCQ individual domain scores (physical limitation, quality of life, clinical summary, and overall).

Exploratory Objectives

The exploratory objectives are to assess the effectiveness of canagliflozin 100 mg daily versus placebo in participants with symptomatic HF by:

- responses on the Patient Global Impression of Change (PGIC) and the Patient Global Impression of Severity (PGI-S)
- healthcare resource utilization (HRU) and health economics data

- associations (correlations and/or categorical association measures) between digital markers (step count and floors climbed), and clinical events such as need for outpatient intravenous therapies, emergency department (ED) visits, and hospitalizations
- daily stairs/floors climbed
- time to first hospitalization/readmission for HF
- time to first hospitalization/readmission for other events
- time to death
- the participant's satisfaction with their experience with the virtual design at the end of the study (see Appendix 2: Participant Satisfaction Survey).

1.2. Study Design

OVERALL DESIGN

This is a randomized, double-blind, placebo-controlled, decentralized, virtual, superiority study conducted in the US in participants with symptomatic HF (stratified by heart failure with HFrEF or HFpEF). This study examines the improvement in the KCCQ-TSS after 12 weeks of treatment with canagliflozin 100 mg or placebo. The study will continue for an additional 6 months without further study intervention to collect participant follow-up data from the smartphone, Fitbit, and claims in a real-world setting. At the end of the 12-week, double-blind treatment period, participants will be informed about their actual treatment allocation, whether they had been randomized to canagliflozin or placebo.

The study will enroll participants from large integrated health networks (IHNs) and physician practices in the US with the participants conducting many of their study-related activities through an app on a smartphone and will provide daily activity data recorded by using a Fitbit device. There will be no in-person clinic visits required by the study (see Appendix 3: Schedule of Activities).

The study consists of a screening period, a 12-week double-blind treatment period, and a 6-month follow-up (no intervention) observational period for each participant. Approximately 1,900 participants will be randomized into the study in a 1:1 ratio to canagliflozin 100 mg daily or placebo groups (950 in each study intervention group). After Week 12 (i.e., the end of the double-blind treatment period), the study will continue for an additional 6 months when study intervention will no longer be provided. Randomization will be performed using the interactive web response system (IxR). Participants will be stratified based on HF ejection fraction (EF) type – HFrEF or HFpEF.

Data collection from the smartphone, Fitbit device, and claims will continue until Month 9 for all endpoints. Specifically, study assessments include the following: KCCQ, PGIC, PGI-S, and an optional participant satisfaction survey at end of study (via smartphone), step count and floors climbed (via the Fitbit device), medical and pharmacy claims data (including up to 36 months history for eligibility confirmation), HRU and health economics (including hospitalization/readmission due to HF or other reason, ED visits, the duration of stay at each

hospitalization/readmission), review of claims for prohibited medications and new diagnoses that may require discontinuation, and adverse events. In addition, information related to outpatient IV diuretics, inotropes and vasodilators and in-patient IV therapies will be considered relevant. Note that claims data involves up to 36 months history to confirm eligibility into this study will not be part of the study database for analysis.

Participants will be of any gender, 18 years of age or older, and will have clinically stable, symptomatic HF with or without type 2 diabetes mellitus (T2DM) with a KCCQ overall summary score of >40 and <80 prior to randomization, which represents New York Heart Association (NYHA) II and III participants.

Figure 1: Schematic Overview of the Study

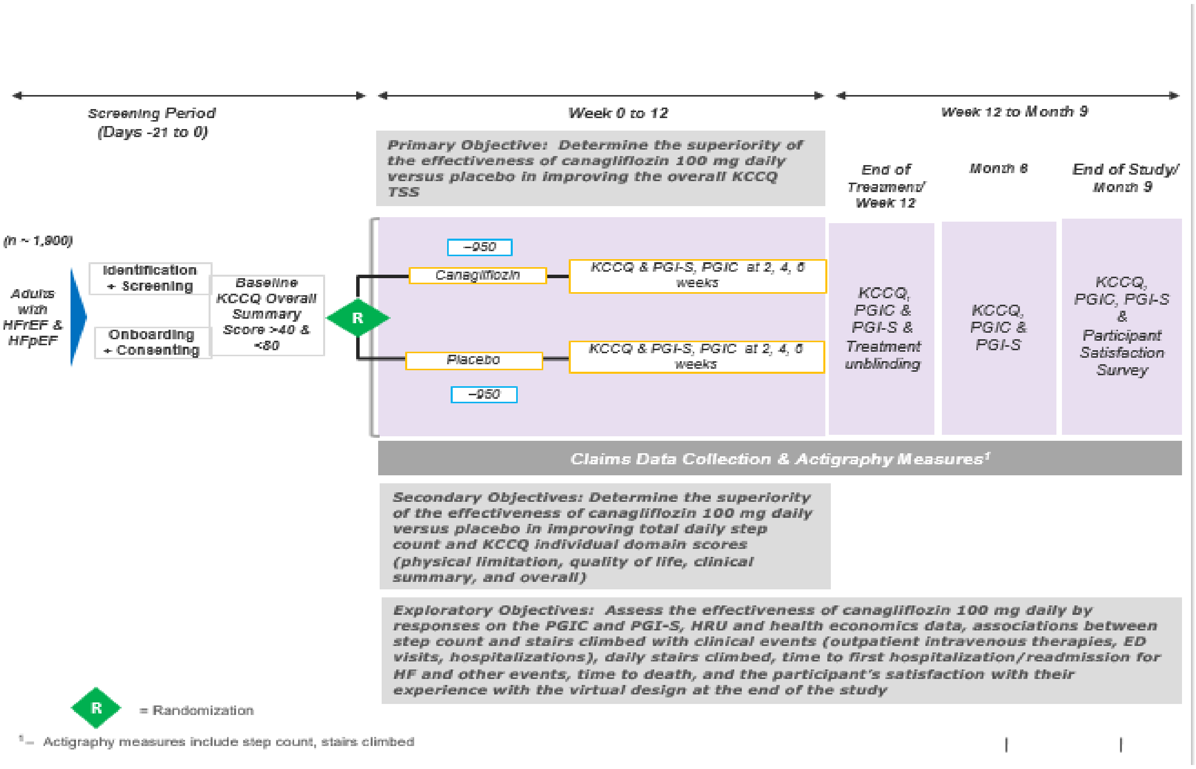

1.3. Statistical Hypotheses for Trial Objectives

The primary effectiveness endpoint is the change in the KCCQ-TSS from baseline to Week 12. The null hypothesis to be tested here is that there is no difference between canagliflozin 100 mg and placebo in the treatment of symptomatic HF based on the primary effectiveness endpoint. For the first key secondary effectiveness endpoint of change in daily step count from baseline to Week 12, the null hypothesis to be tested is that canagliflozin 100 mg is not different from placebo with respect to the mean daily step count.

The 2 hypotheses described above will be tested using a fixed-sequence (hierarchical) testing procedure to control the familywise type I error rate at 5% as follows:

1. (primary effectiveness endpoint): change from baseline to Week 12 in KCCQ-TSS for canagliflozin 100 mg versus the placebo

2. (first key secondary endpoint): change from baseline to Week 12 in daily step count for canagliflozin 10 mg versus placebo.

If the result of the 2-sided p-value from the first hypothesis (i.e., primary effectiveness endpoint) test is ≤0.05, then the second hypothesis test result will be interpreted inferentially at the 5% significance level. This sequential testing approach will control the familywise type I error rate at 5%.

Hypotheses related to the secondary effectiveness endpoints (i.e., for KCCQ individual domain scores defined by changes in physical limitation, quality of life, clinical summary, and overall summary from baseline to Week 12) as well as other exploratory endpoints will be tested at the nominal significance level (5%) without adjustment for multiplicity (or controlling type I error rate).

1.4. Sample Size Justification

The primary effectiveness endpoint is the change from baseline in the KCCQ-TSS at the end of the 12-week double-blind treatment period. Although a 5-point improvement in the KCCQ-TSS is generally viewed as clinically meaningful,[2,12] recent studies[7,8] have shown that improvements in the range of 3 to 5 points on the KCCQ-TSS are equally meaningful in the overall HF-related health status measured by the KCCQ over a 12-week time period. In considering the clinical significance of scores, it is important to separate mean changes for groups of participants, as compared with intra-individual changes. On the TSS, there are 7 items (4 for symptom frequency and 3 for severity/burden).[3,11] Shifting a response on KCCQ questions 5 and 7 shifts the TSS score by 2.083 points on a 100-point scale. A shift in response for questions 3 and 9 shifts the score by 3.125 points and for items 4, 6, and 8, shifts the score by 4.166 points, assuming no missing responses. Thus, an intra-individual change of at least 2 categories (3 if the only change is in items 5 and 7) would exceed the 5-point threshold that has the been shown to be clinically significant. When analyzing groups of participants, the mean difference across the entire population includes a distribution of participants with different magnitudes of individual change and a 3- to 5-point difference between groups reflects a significantly larger proportion of participants in one group, as compared with the other, who have clinically important improvements in their TSS. The mean differences between the groups will be augmented with both responder analyses and empirical cumulative distribution and probability density function curves. Thus, based on the totality of evidence evaluating the clinical significance of the KCCQ,[5,6,9,14] the current study is powered to detect a mean change from baseline of 3 points in the KCCQ-TSS between canagliflozin 100 mg and placebo groups at Week 12. Assuming a significance level of 5% and a standard deviation (SD) of 18 points for mean change in KCCQ-TSS, a total of 1,900 participants will be randomized in this study providing approximately 95% power. This sample size is adequate to account for a potential 5% to 10% dropout rate - that is, with a dropout as high as 10% [n=1,710], the statistical power remains >90%.

1.5. Randomization and Blinding

Central randomization will be implemented in this study. Participants will be randomly assigned to 1 of 2 study intervention groups based on a computer-generated randomization schedule prepared before the study by or under the supervision of the sponsor before initiation of the study.

Randomization will be balanced by using randomly permuted blocks and will be stratified by EF (HFrEF or HFpEF) at study entry as recorded in the participant's electronic health record (EHR).

The interactive web response system (IxR) will generate a randomization code, participant randomization number, and kit number once consent is completed and the participant is confirmed eligible by the virtual principal investigator. The kit number will dictate the study intervention assignment and the matching study intervention bottle(s) to be shipped to the participant directly from the drug distribution vendor.

The IxR will provide the kit number of the study intervention bottle(s) to be dispensed for each randomly assigned participant of the double-blind treatment period. Based on these randomization codes, study intervention will be packaged and labeled in a manner that maintains the double-blinded nature of the study.

The drug depot will not be provided with randomization codes. The codes will be maintained within the IxR, which has the functionality to allow the virtual principal investigator or designee to break the blind for an individual participant.

Under normal circumstances, the blind should not be broken until participants have completed the 12-week, double-blind treatment period. The virtual principal investigator or designee may in an emergency determine the identity of the study intervention by contacting the IxR. In the event the blind is broken, the sponsor must be informed as soon as possible. The date and reason for the unblinding must be documented in the study database. All randomization codes will be released after completion of the study. Participants who have had their intervention assignment unblinded should continue to perform their scheduled evaluations.

1.6. Study Data Flow

Data flow in this study is facilitated by an automated integrated process that extracts clinical data from 3 sources: PRA mobile health platform [PRA mHP], Care Innovations, and Symphony. This data is loaded into PRA's Common Clinical Data Store (CCDS) where the data is accessible by JReview for data validation and analysis, as well as for extract into RAW and SDTM datasets. This automated Extract-Transform-Load (ETL) process will utilize a data container on Amazon Web Services (AWS), referred to as the "Data Analytics Container" or "DAC", which is managed by PRA. A more detailed data flow diagram is provided in Appendix 5 of this SAP.

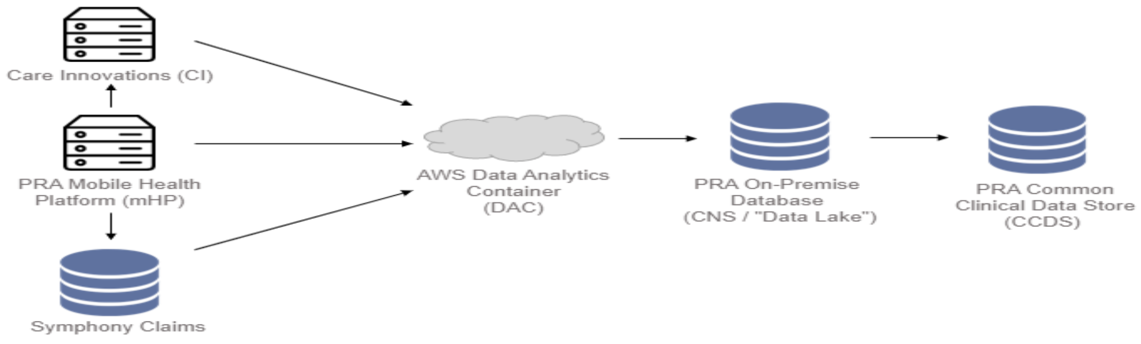

The PRA mHP platform serves as the main hub for source data capture on the CHIEF-HF study. It captures eConsent, information entered by Health Network Systems (HNS) from a participant's electronic medical records (EMRs), participant status overseen by Care Innovations (CI), randomization through integration with Endpoint IRT, drug shipment through integration with Marken, primary and secondary endpoint data from ePROs (KCCQ, PGI-S, PGIC), dosing compliance, as well as activity measured by the Fitbit watch. PRA mHP data will go into the DAC via a direct database connection. Medical and pharmacy claims data is contained within Symphony Health Solutions and exists as completely de-identified data. This includes data used in the study to review and analyze adverse events, healthcare resource utilization (HRU) and health economics (including hospitalization/readmission due to Heart Failure [HF] or other reason, Emergency Department (ED) visits, the duration of stay at each hospitalization/readmission), as well as review of prohibited medications and new diagnoses that may require discontinuation.

Symphony claims data will be pulled into the DAC for participants randomized into the study via PRA mHP eConsent and will be mapped to SDTM as detailed in the SDTM specifications relevant to the study protocol and SAP. The collection timeframe for claims associated with AEs of special interest will begin on the Informed Consent Date through the End of Treatment + 30 days. The collection timeframe for Pharmacy claims of prohibited concomitant medications (SGLT2i) will begin on the ICF date through End of Treatment. The collection timeframe for claims associated with Health Resource Utilization will begin on the ICF Date through the 9-month observation period.

2. GENERAL ANALYSIS DEFINITIONS

As this is a virtual study, there are no planned in-person clinic visits for measuring key endpoints on the study participants. Data collection via smartphone, Fitbit device, and claims will continue throughout the 9-month duration of the study. While the focus of the first 12-week, double-blind treatment duration is to establish the primary and key secondary objectives related to primary and secondary effectiveness endpoints, the latter 6 months (i.e., Month 3 until Month 9) of the study (when no study intervention is provided) are aimed to explore multiple objectives by collecting additional follow-up data on PROs, daily activity, and HRUs and generate hypotheses of interest for future studies.

Unless stated otherwise, following the intent-to-treat (ITT) principle, analyses of the primary and secondary endpoints will be based on all randomized participants and include data collected in the 12-week, double-blind treatment period. Where meaningful, these analyses may also be repeated using the per protocol analysis set. Analysis based on all randomized set will also be extended to the full study data period (up to the end of the 9-month long study). In a similar way, exploratory endpoints will be analyzed according to the randomized intervention groups by using data from both the 12-week, double-blind treatment period and the 9-month study duration, respectively.

2.1. Time Windows

In this virtual study, the randomization date will refer to the day of randomization of an eligible participant. Once randomized, study intervention will be shipped and delivered to the randomized participant directly, and therefore, the first study intervention may be started by a participant a few

days after the actual randomization date. This will be considered the date of starting study intervention, or, a reference day (Study Day 1). Scheduled study intervention will end after 12 weeks. After Week 12, participants will be followed up to Month 9 without receiving any further study intervention. Total study days will be numbered relative to the Study Day 1 for each participant up to the end of the 9-month long study. All measurements will be arranged in a chronological order relative to Study Day 1. Participants will be instructed and notified by the study app on their smartphone to confirm taking the study intervention on schedule and completing all necessary patient reported outcome (PRO) measurements. Study app is programmed to allow PRO measurements to be recorded only within a pre-specified window of days around each scheduled time point.

The primary, secondary and exploratory variables (ie, KCCQ and its individual domains, daily step count, daily floors climbed, PGIC and PGI-S, participant satisfaction survey, etc.) will be measured at specific time points as identified in the protocol, using either a smartphone-based mobile app or, the Fitbit device, as is relevant, registered by the study participant. Measurements taken closest to the planned (target) time points will be considered for the endpoint analyses and will be appropriately assigned to a time window to allow reasonable variations around the target day. Endpoint for analyses and summaries is the last recorded postbaseline data.

Daily activity measurements via the Fitbit device will be collected continuously from the time study started (ie, when both the smartphone and the Fitbit are linked by a participant). Fitbit daily activity data will be aggregated into averages over 2-week intervals starting with a baseline period (ie, Baseline = average of Study Day 1 to Study Day 14) and similarly over the entire post-baseline study duration for each participant. This is to allow capturing a robust length of physical activity for each participant using the Fitbit device and minimizing variability associated with measurements in shorter time spans. The end-of-treatment (EOT) average value of the step count will be the average of the last 14 days prior to the last dose of study intervention, regardless of when the last dose is taken.

Claims data will be extracted as access to participant-level information becomes available. This will include clinical outcomes and adverse events (AEs) (including hospitalizations for HF and/or other reasons) coded using International Statistical Classification of Diseases and Related Health Problems (ICD)-9 and ICD-10 as appropriate.

Table 1 presents the time windows and the target days for each time of measurement defined in the protocol.

Table 1: Measurement Windows

Parameter	Time Interval (label on output)	Time Interval (Day)*	Target Time Point (Day)
KCCQ (and its individual domains); PGIC and PGI-S; participant satisfaction survey	Screening/Baseline	-21 to <1	1
	Week 2	11 to 17	14
	Week 4	25 to 31	28
	Week 6	39 to 45	42
	Week 12	81 to 87	84

Parameter	Time Interval (label on output)	Time Interval (Day)*	Target Time Point (Day)
	Month 6	161 to 175	168
	Month 9	245 to 259	252
Daily Step Count / Daily Floors Climbed **	Baseline to Week 2	1 to 14	
	Week 3 & Week 4	15 to 28	
	Week 5 & Week 6	29 to 42	
	Week 7 & Week 8	43 to 56	
	Week 9 & Week 10	57 to 70	
	Week 11 & Week 12	71 to 84	
	…	…	
	Week 35 & Week 36	238 to 252	

*Relative to Study Day 1
** Measurements over a 2-week interval with ≥ 100 daily steps to allow capture of physical activity

2.2. Pooling Algorithm for Analysis Centers

This is a virtual study with multiple IHNs competitively enrolling HF patients to meet the overall study enrollment target. Therefore, from the analytical perspective, no separate sites exist for pooling to be applicable. It will be considered a single center study.

2.3. Analysis Sets

An overview of the different analysis sets and their usage is presented in Appendix 4: Usage of the Analysis Sets.

2.3.1. All Randomized Analysis Set

The all randomized analysis set includes all participants who are randomly assigned to a study treatment. In order to adhere to the ITT principle, analyses based on the all randomized analysis set will use the following rules:

- participants will be evaluated according to the study intervention they have been assigned to (which may be different from the study intervention they have received)
- all available data will be included (e.g., including assessment after study intervention discontinuation).

Analyses of the primary, secondary, and exploratory endpoints are based on the all randomized analysis set.

2.3.2. Per Protocol Analysis Set

The per protocol analysis set (PP) will consist of all participants who received study intervention and who complied with the protocol to allow assessment of the treatment effects. Specifically, this includes participants who comply with the study intervention, have all key measurements available, and do not have major protocol deviations that have an impact on the treatment effect. The effectiveness analysis for the primary and secondary endpoints will be repeated for the PP analysis set.

2.3.3. Safety Analysis Set

The safety analysis set includes all randomized participants who received at least 1 dose of study intervention. Summary of available safety data from all sources (claims and patient reported) will be based on this analysis set.

2.4. Definition of Subgroups

Analysis for the primary effectiveness endpoint will be performed for the subgroups identified below.

**Subgroup**	**Definition**
Region**	US - East, West, North and South
Age Group	• 18-25<br>• 26-50<br>• 51-64<br>• >65
Gender	• Male<br>• Female
Race	• White<br>• Non-White
Diabetes type	• T2DM<br>• No-T2DM
HF – Ejection Fraction type	• pEF (preserved ejection fraction)<br>• rEF (reduced ejection fraction)

**Information on region will be based on details available through IHNs.

Additional subgroups may be identified (including those based on combinations of T2DM and HF ejection fraction types) before finalizing the database.

2.5. Study Day and Relative Day

Study Day 1 refers to the start of the first study intervention administration. All assessments recorded via the smartphone/mobile app and/or Fitbit device will be assigned a day relative to this date.

Study Day 1 or relative day for a specific time point of measurement is defined as:

- measurement date - (date of Study Day 1) +1, if measurement date is ≥ date of Study Day 1
- measurement date - date of Study Day 1, if measurement date < date of Study Day 1.

2.6. Baseline, End of Treatment, and End of Study

Baseline is defined as the last observation made prior to the start of the first study intervention administration. End of Treatment (EOT) observation will refer to the last observation made prior to and including the day of the last dose of study intervention, regardless of when the last dose is taken. For the PRO measurements, EOT observation will include the last observed value prior to the last dose of study intervention, and/or, within a measurement window allowed by the app that may overlap the last dose date (i.e., may extend the last dose date by a few days). For the daily

activity data, it will represent average from the last 14 days prior to the last dose taken, regardless of when the dose is taken.

The EOS value is defined as the observation that is taken on the date of very last available postbaseline result in the study period. Unscheduled measurement results are included in this definition and will be considered as the last timepoint value if the unscheduled measurement result is the last postbaseline result available within the analysis period.

2.7. Imputation Rules for Missing AE Date/Time of Onset/Resolution

A treatment emergent adverse event (TEAE) in this study is defined as an event that is reported in the claims database along with a related service date that occurs after the first dose of study intervention (Study Day 1) and during the double-blind treatment period plus 30 days. Data from the claims database includes reported AEs and dates of post-baseline services (procedure dates, office visits, hospital admissions, etc.) but not start/end dates of these events when claims are filed. Therefore, no imputation of start/end dates for AEs or clinical outcomes are possible. Dates of service(s) rendered to participants for AEs cover the period starting from the time their informed consent is signed until the end of their double-blind treatment period plus 30 days which will be pulled into the final safety database. For clinical outcomes, information collected until the end of the study will be included.

3. INTERIM ANALYSIS AND DATA MONITORING COMMITTEE REVIEW

No interim analysis is planned in this study.

Data from the 12-week double-blind treatment period will be analyzed/summarized first and reported out as soon as the last randomized participant has completed the double-blind treatment period. This may be used in facilitating an early regulatory review of the main findings (effectiveness/safety results) as appropriate. Data from the full 9-month study period will be reported later once the remainder of the follow-up with no study intervention is completed on all study participants and will be included in the final clinical study report (CSR).

4. SUBJECT INFORMATION

The number of participants in each analysis set will be summarized and listed by intervention group, and overall. In addition, the distribution of participants by region within the US and recruiting networks will be presented, unless otherwise noted.

4.1. Demographics and Baseline Characteristics

Table 2 presents a list of the demographic variables that will be summarized by intervention group, and overall for the all randomized analysis set. Demographics will also be summarized by various IHNs (or regions) using the all randomized analysis set.

Table 2: Demographic Variables

**Continuous Variables:**	**Summary Type**
Age (years)	Descriptive statistics (N, mean, standard deviation [SD], median and range [minimum and maximum], and IQ range).
**Categorical Variables**	

Age (18-25 years, 26-50 years, 51-64 years, and ≥65 years)	Frequency distribution with the number and percentage of participants in each category.
Sex (male, female)	
Race[a] (American Indian or Alaska Native, Asian, Black or African American, Native Hawaiian, or, other Pacific Islander, White, Other)	
Diabetes status at baseline (T2DM or no T2DM)	
HF ejection fraction type (HFrEF or HFpEF)	

a If multiple race categories are indicated the Race is recorded as 'Multiple'.

4.2. Disposition Information

Total number of screened participants and number of screen failures will be provided. The total number of participants randomized into the study will be provided. Randomized participants who complete the 12-week, double-blind treatment period and who do not discontinue study intervention prematurely are considered study intervention completers.

The number of participants in the following disposition categories will be provided for the 12-week, double-blind treatment period by intervention group and overall:

- participants randomized
- participants who were randomized yet did not receive/initiate study intervention
- participants receiving study intervention
- participants completing the double-blind treatment period
- participants who discontinued study intervention
- participants who terminated study prematurely
- reasons for discontinuation of study intervention and termination of study
- participants who were unblinded during the double-blind treatment period.

The distribution of the time to study termination for the 12-week, double-blind treatment period will be displayed with Kaplan-Meier curves. Participants who terminate study participation prematurely at any time will be considered an 'Event' and their date of study termination will be used in the time to study termination calculation. In this analysis, participants who complete the 12-week, double-blind treatment period will be considered 'censored' and the date of double-blind treatment period completion will serve as the time of censoring.

4.3. Treatment Compliance

Participants will be instructed and notified by the study app weekly to confirm the number of study intervention capsules that they took that week. Study intervention compliance data will be monitored bi-weekly for ensuring compliance is >80%.

Compliance with the study intervention is defined in this study in terms of the total number of doses received during the total treatment period of 12 weeks and not in terms of maintained drug concentration while participating in the study. Participants who take study intervention as instructed >80% of the total study intervention duration will thus be considered compliant.

For the double-blind study intervention period, compliance (%) = 100 x number of days taking study intervention (pills)/total study intervention duration (including days off study intervention).

The number and percentage of participants who have >80% study intervention compliance through Week 12 (end of double-blind treatment period) will be provided by randomized group.

4.4. Extent of Exposure

The number and percentage of participants who receive study intervention will be summarized by randomized group.

Descriptive statistics for study intervention duration (N, mean, SD, median, and range [minimum, maximum]) will be presented by randomized group based on the safety analysis set. Person-years of exposure to study intervention are calculated [as days of intervention/365.25] and will be presented by randomized group.

Duration of study intervention will be summarized in the following duration categories: [<1 week, 1-<2 weeks, 2-<3 weeks, …, 11-≤12 weeks] by randomized group and presented graphically in a histogram.

Cumulative study intervention exposure [≥1 week, ≥2 weeks, …, ≥12 weeks] will also be summarized.

Study intervention duration is defined as (date of last dose of study intervention – date of first dose of study intervention) +1.

4.5. Protocol Deviations

In general, the following list of major protocol deviations may have the potential to impact participants' rights, safety, or well-being, or the integrity and/or result of the clinical study. Participants with major protocol deviations will be identified prior to database lock and summarized by following categories:

- developed withdrawal criteria but not withdrawn
- entered but did not satisfy inclusion criteria
- received a disallowed concomitant/prohibited intervention (eg, another SGLT2i other than canagliflozin)
- received the wrong study intervention or incorrect dose
- other.

4.6. Prior and Concomitant Medications

In this pragmatic virtual study, other than history on prior medications up to 36 months, no additional information including specific start/end dates of using prior medications will be available. However, dates of prescription-fills for specific concomitant/prohibited medications collected through the claims/pharmacy database for the duration of the study may be available. The concomitant medication use during study may be categorized into broad classes of **glucose-lowering agents** (such as insulin, metformin, sulfonylureas, DPP-4 inhibitors, GLP-1 receptor agonists), **renal and cardiovascular (CV) protective agents** (such as RAAS inhibitors [including sac/val], statins, antithrombotics, beta-blockers, diuretics, IV in-patient diuretics, vasodilators, inotropes, anti-infectives) and **UGT inducers** (such as carbamazepine, phenytoin, phenobarbital, tiprnavir, rifampicin, testosterone, propionate, nelfinavir, ritonavir, efavirenz, lamotrigine) .

5. EFFICACY

5.1. Analysis Specifications

Descriptive statistics for continuous variables include count (n), mean, SD, standard error (SE), median, minimum, and maximum. Descriptive statistics for categorical variables include participant counts and percentages.

5.1.1. Level of Significance

A 5% level of significance will be used for all analyses. All statistical tests and 95% confidence intervals presented in the analyses of endpoints will be 2-sided.

5.1.2. Data Handling Rules

This study will obtain mainly 3 types of data for statistical analysis: the smartphone/app-based PRO data, the activity data collected from the Fitbit device and the claims data (including adverse events, clinical outcomes, prescription fills, and related HRU). In addition, participant's self-reported AE data collected via a Call Center will be directly sent over to Janssen Safety separately and is described below as well. All data will be received and processed by PRA (3rd party vendor, see Appendix 5: CHIEF-HF Study Data Flow Diagram) into analysis files based on specifications provided by the sponsor/study SAP unless specified otherwise.

Smartphone Data - PRO Measures

PRO data (ie, KCCQ, PGIC and PGI-S, participant satisfaction survey) will be collected via the smartphone apps available with participants. Based on the administration of the KCCQ at screening/baseline through an app, an overall summary score will be produced to decide eligibility of individual participant into the study. The individual domains and summary scores of KCCQ will be calculated using the scoring algorithm provided in the KCCQ manual at specified time points during the study. Missingness in these PRO measures may result from participants not completing the questionnaires on the smartphone app at the scheduled times during the study period for various reasons including non-compliance with the protocol, discontinuation due to AE, loss to follow up, etc.

Missing and incomplete data in KCCQ PRO measure may be of 2 types: missing at the form-level and at the item-level. Form-level missing data refers to a participant missing an entire PRO assessment for a scheduled time point. If not otherwise specified, analyses will be conducted on all available data and form-level missing data will not be imputed. For the KCCQ, derivation of individual domain scores in the presence of missing item-level responses will follow algorithms specified in the KCCQ manual. PRA will calculate baseline KCCQ overall summary score based on the KCCQ app and will include this in the analysis database. Janssen will calculate of all other KCCQ domain scores (including the KCCQ-TSS) at baseline and at all post-randomization time points, using the scoring instructions available per the KCCQ manual (see Appendix 8). For the PGIC and PGI-S measures, a single imputation (ie, last observed scores carried forward) for missing post-baseline scores will be used as a conservative rule. Vertical file formats will be prepared for all PRO data, reading one record per patient for each measurement time (ie, baseline, Weeks 2, 4, 6, and 12, and Months 6 and 9, respectively).

Fitbit Data - Clinical (Activity) Measures

Data from the Fitbit device pertaining to the daily total step counts and daily total floors climbed will be aggregated over 2-week intervals for the entire study duration (see Table 1). Daily stairs climbed information from the Fitbit will be considered as equivalent to daily floors climbed in the analysis. Due to the general sedentary nature of HF patients, Fitbit recorded <100 steps taken in a day will not be considered in the calculation of averages for daily step counts and daily floors climbed. If missing >7 days (50%) in a 2-week interval, the corresponding 2-week interval average will be set to missing.

In case of premature discontinuation of the study intervention, the average of the last non-missing 14-day window prior to the last dose of study intervention will always be used as an EOT value. If the EOT value corresponding to the period (EOT-14, EOT-1) is missing (not valid), the EOT period will be rolled back by one day, ie, (EOT-15, EOT-2), (EOT-16, EOT-3), until a non-missing (valid) value is available. This corresponds to a last observation carried forward (LOCF) up to Week 12/EOT approach. If no post-baseline daily activity data have been collected for at least 7 days over a 14-day time window, no EOT value will be computed, and the post-baseline value will be set to missing.

Baseline for daily step count and daily floors climbed variables will be defined by averaging the daily values over a 14-day interval starting with the first dose date. These data will be organized into a vertical file format to read one record per participant for averages over each 2-week interval during the entire study (including the EOT/EOS values, as applicable).

Claims Data – AEs, HRUs, Clinical Outcomes

Claims data pertaining to AEs of interest (as defined in the protocol), number of prescription fills, HRUs, and clinical outcomes reported with appropriate ICD codes applied at the individual participant level will be organized with one record for each incident (ie, hospitalization, etc.) date.

Self-reported AE data – Data from participants self-reporting adverse events to a Call Center (set up by Care Innovations) will be manually submitted directly to the Sponsor (i.e. Janssen Safety

[GMS]) for appropriate evaluation and reporting requirements. Janssen Safety (GMS) will extract participant-reported AEs and transfer data to PRA for upload into the CCDS for CSR reporting purposes. Self-reported events will be captured from the date of the first dose of study intervention through the end of treatment (Month 3) plus 30 days for each participant.

5.2. Primary Efficacy Endpoint

5.2.1. Definition

The primary effectiveness endpoint is the change in the KCCQ-TSS from baseline to Week 12.

5.2.2. Estimand

The primary estimand, the main clinical quantity of interest to be estimated in the study, is defined by the following 5 attributes (ICH E9 [R1]).[4]

- Population: all randomized participants (i.e., randomized participants with HF who either have HFrEF or HFpEF, regardless of T2DM status)
- Treatment: canagliflozin 100 mg or placebo
- Variable: change from baseline to Week 12 post first dose in the KCCQ-TSS
- Intercurrent events: treatment discontinuations (either due to AE or other reasons) or treatment switching; use treatment policy strategy to address intercurrent events (i.e., use all available measurements collected during study, regardless of the occurrence of the intercurrent event); events of “loss to follow up” or “study withdrawal” or “death” are not considered intercurrent events but rather missing data.
- Population-level summary: difference in mean change from baseline to Week 12 in the KCCQ-TSS between randomized groups.

In other words, the main quantity to estimate for the primary effectiveness endpoint is the mean difference between the 2 treatment groups (canagliflozin 100 mg and placebo) with respect to the change from baseline to Week 12 in the KCCQ-TSS.

5.2.3. Analysis Methods

The primary effectiveness endpoint analysis will be based on the all randomized analysis set.

Considering the repeated measurements of the KCCQ-TSS in the double-blind treatment period (ie, at baseline, Weeks 2, 4, 6, and 12 respectively), the primary effectiveness endpoint (change in the KCCQ-TSS from baseline to Week 12) will be analyzed by a MMRM method simultaneously adjusting for treatment, stratification factor (HFrEF or HFpEF), time, time by treatment, and baseline KCCQ-TSS value as covariates. Time is repeated within participants and an unstructured covariance structure will be assumed across treatment groups to model the within-participant errors. Fixed effects tests will be computed using the Kenward-Rogers method to determine the degrees of freedom of the denominator. In case of convergence issues, alternative variance-covariance structures will be evaluated in the following order, with the first structure in the following list that converges being used in the analysis: heterogeneous Toeplitz, standard Toeplitz, and AR (1) with separate participant random effect. The primary comparison will be based on the

difference in the LS mean changes from baseline to Week 12 between treatments. The treatment difference in the LS means, corresponding 95% confidence intervals (CI) and associated p-value will be calculated. The LS means ±SE of change from baseline values estimated by MMRM will be plotted by each timepoint for each intervention group.

```
PROC MIXED DATA=analysis_dataset;
CLASS treatment time ptid;
MODEL change = treatment time treatment*time baseline strat_var/DDFM=KR;
REPEATED time / SUBJECT=ptid TYPE=UN;
LSMEANS treatment;
LSMEANS treatment*time/pdiff cl;
RUN;
```

An MMRM model assumes the KCCQ-TSS data missing at random (MAR). A sensitivity analysis will be performed involving multiple imputation (MI) modeling approach for missing KCCQ-TSS values when the MAR assumption is considered not valid. Implementing the MI process requires 3 steps: 1) each missing value is imputed multiple times, based on statistical modeling, resulting in repetitions of the original dataset, along with the newly imputed values (implemented in the Statistical Analysis System [SAS] using PROC MI); 2) each dataset is analyzed using an MMRM model (implemented in SAS using PROC MIXED). The LS means and SEs from each analysis will be output to a SAS dataset; 3) results from the analyses performed on multiple datasets in step #2 are combined to provide valid statistical inference (implemented in SAS using PROC MIANALYZE).[1]

A complete SAS code for MMRM model and implementation of the MI approach described above will be added to the SAP appendix.

An exploratory responder analysis will be performed when comparing proportions of participants with a 5- (or 10-) point improvement in the KCCQ-TSS from baseline to Week 12 between the groups. This will be based on a 2-sample difference in proportions test along with a 95% confidence interval for the difference in 2 proportions.

In addition, the empirical cumulative distribution function (CDF) (a continuous plot of each individual participant’s change scores) of the KCCQ-TSS will be generated. CDF shows the proportion of the population scoring less than or equal to each possible change score. CDFs are useful as they graphically characterize the treatment effect or differences between groups. It also enables a ready assessment of different magnitudes of clinical change (e.g., 5, 10, and ≥20-point changes, corresponding with small to moderate, moderate to large, and large to very large clinical changes). CDFs and histograms will be shown for changes from baseline to Week 12 in the KCCQ TSS. For all CDF plots, the x-axis is the score change from baseline to final time point (i.e., Week 12) for the KCCQ-TSS. The y-axis is the cumulative proportion of the participants in each of the corresponding categories that reach the score change on the x-axis.

According to the Food and Drug Administration’s (FDA’s) guidance on PRO measures, a responder is defined by the empirically determined score change in a measure, experienced by an individual patient over a predetermined time period that has been demonstrated in the target

population to have a significant treatment benefit. This is also often referred to as responsiveness or ability of the PRO measure to capture the true underlying change in the patient health status over time. Assessing the responsiveness of the PRO measure using an anchor-based approach involves comparing the changes in the PRO scores to other clinically meaningful markers or anchors that represent a valid measure of clinical change.

For the anchor-based analysis of the KCCQ-TSS endpoint, participants will be characterized based on the PGIC scores at Week 12. The mean change in KCCQ-TSS endpoint observed in the smallest improvement category of the PGIC will be examined as a key anchor-based indicator of a responder. The definition for a small improvement in PGIC is given as those who improve by one category (i.e., from “a little improved” to “somewhat improved”). Specifically, the retrospective PGIC responses of “3=a little improved” will be selected to represent time points where participants register meaningful changes in their HF symptoms. Each time a participant responds with a PGIC score of “3=a little improved”, a KCCQ-TSS change score from the preceding visit is computed. For the double-blind treatment period, the PGIC will be collected at 4 time points (i.e., Weeks 2, 4, 6, and 12 respectively) which can yield between zero and 4 KCCQ-TSS change scores by each participant. For each participant with at least one PGIC response of “3=a little improved”, a mean of the individual participant’s KCCQ-TSS change scores is computed. A summary of KCCQ-TSS change scores across PGIC categories[16] will then be presented.

The KCCQ-TSS change scores may also be standardized into effect size (ES) statistics by dividing them by their SD at baseline. The following cutoff values will be used to interpret effect size: small ES = 0.2, moderate ES =0.5, and large ES = 0.8. Together with the CDF, results from the anchor-based methods will be examined and a single responder definition threshold will be determined, if the results from the anchor-based assessments in this study differ substantially from prior studies examining the clinical meaningfulness of changes in the KCCQ.

In addition, a distribution-based change (i.e., minimum detectable [MID] change) will be calculated, for which the KCCQ-TSS can be derived using the baseline SD. Generally, a value of half the baseline SD has been frequently found to correspond to the MID obtained via the anchor-based method. A value of 0.2*SD may also be examined.

Subgroup analyses for the change from baseline to Week 12 for the primary effectiveness endpoint will be conducted for subgroups (listed in Section 2.4) based on the all randomized analysis set. These analyses will assess consistency of the overall treatment effect across subgroup variables.

The main MMRM analyses will be repeated with each subgroup tested separately. Intervention group differences of LS means as well as corresponding 95% CIs within each subgroup level will be presented.

The intervention-by-subgroup interaction p-value will be estimated using a separate MMRM model including intervention, baseline KCCQ value, stratification factor (HFrEF/HFpEF), subgroup, and intervention-by-subgroup interaction term(s). Interactions with p-value <0.1 will be investigated further to determine the nature of interaction (quantitative or qualitative) and the

association with other subgroups. For qualitative interactions, Gail and Simon (1985) approach[15] may be considered.

Results will be presented in a summary table and in a forest plot. The forest plot will display the "overall" treatment effect, based on the main MMRM analysis, as a reference line. LS means of the intervention group differences, the corresponding 95% CIs, and the numbers of participants in the subgroup levels will be presented in the forest plot.

5.3. Secondary Endpoints

5.3.1. Definition

The first key secondary effectiveness endpoint is change in the daily step count from baseline to Week 12. Even though evidence of a meaningful change in daily step count in chronic HF patients via Fitbit device is not readily available, there are several studies that have conducted six-minute walk tests (6MWT) among HF patients in general and examined a minimum clinically important difference in daily step counts under different conditions. A change of 30 meters[10] (approximately 1000 steps) may be considered in the present study to be of clinical interest to benchmark the expected mean change in daily step count in this study over the double-blind treatment period. This expected mean change in daily step count will also be examined in relation to variables such as PRO measures (as anchors) and clinical outcomes to better characterize treatment effect on daily activity.

As HF patients are generally not consistent in their daily walk patterns, there will be variations in levels of their daily activity (whether it is a weekday or a weekend) as well as variability in the total wear time of the Fitbit device. As a rule, we will consider Fitbit recorded ≥100 steps[13] taken in a day in the calculations of averages. This will ensure capturing any meaningful level of physical activity while avoiding even smaller variations in daily steps taken by a participant wearing a Fitbit device. For each participant, baseline daily step count will be calculated by averaging daily step counts from Study Day 1 through Study Day 14. All subsequent post-baseline daily step count data will also be averaged in 2-week intervals. The change from baseline to Week 12 in daily step count will be based on subtracting the average of daily step count for Week 11 and Week 12 from the baseline average for each participant. Additionally, daily step count data will be averaged in 3-days, 5-days and 7-days (1-week) intervals at baseline and throughout the treatment period respectively and corresponding changes from baseline will be calculated. Daily floors climbed will also be aggregated over 3-days, 5-days, 1-week, and 2-week intervals in a similar fashion.

5.3.2. Estimand

The first key secondary estimand to be estimated in the study, is defined by the following -

- Population: all randomized participants (i.e., randomized participants with HF who either have HFrEF or HFpEF, regardless of T2DM status)
- Treatment: canagliflozin 100 mg or placebo
- Variable: change from baseline to Week 12 in the daily step count averaged over 2-week intervals

- Intercurrent event: treatment discontinuations (either due to AE or other reasons) or treatment switching or temporary disruption in daily activity; use treatment policy strategy to address intercurrent events (i.e., use all available measurements collected during study, regardless of the occurrence of the intercurrent event); events of “loss to follow up” or “study withdrawal” or “death” are not considered intercurrent events but rather missing data.
- Population-level summary: difference in mean change from baseline to Week 12 in the daily step count averaged over 2-week intervals (i.e., difference is based on subtracting the average of daily step count for Week 11 and Week 12 from the baseline 14-day average daily step count) between two randomized groups.

Other secondary endpoints refer to changes in the KCCQ individual domain scores (physical limitation, quality of life), and the KCCQ clinical summary and overall summary scores from baseline to Week 12. The individual KCCQ domain scores as well as the summary scores (clinical summary and overall summary scores) will be produced at each time point (based on the KCCQ algorithm) with changes from baseline calculated at each specified post-baseline time point.

5.3.3. Analysis Methods

Daily step counts and daily floors climbed data at baseline will be presented as 14-day averages of daily counts starting with the first dose date and ending with Day 14 during the double-blind treatment period. This method of averaging will continue until end of Month 9 (Day 252) or, until the time of end of study (EOS) observation is available.

The first key secondary endpoint is calculated as the change from baseline to Week 12 in daily step count using the corresponding 14-day averages at baseline and Week 12 respectively. The mean change is calculated for each randomized intervention group and difference in mean changes between the 2 randomized intervention groups will be compared using a 2-sided (paired) t-test.

As stated in Section 1.3, the first key secondary endpoint of daily step count will be tested once the primary endpoint is shown to be statistically significant at 5% significance level.

Longitudinal changes over time in daily step counts using a 3-, 5-, 7- and 14-days averages will also be explored using an MMRM model, if necessary. This model will adjust for baseline covariates including treatment, stratification factor, and baseline average step count. For other secondary effectiveness endpoints, an MMRM model (like that specified for the analysis of the primary endpoint) will be employed to summarize changes in the KCCQ individual domain scores (physical limitation, quality of life, clinical summary, and overall) from baseline to Week 12. Hypotheses related to these domains will be independently tested at 5% without any control for overall type I error rate.

5.4. Exploratory Variables

5.4.1. Definition

The exploratory endpoints are the following:

- mean PGIC scores over time
- changes in PGI-S scores from baseline to Week 12

- change in number of daily stairs/floors climbed
- change in HRU and health economics data
- number of outpatient intravenous therapies, ED visits, and hospitalizations
- time to first hospitalization/readmission for heart failure
- time to first hospitalization/readmission for other events
- time to death

5.4.2. Analysis Methods

The exploratory endpoints listed below will be presented using descriptive statistics at each time point measurements are taken. Where specified, 95% confidence intervals (CIs) and p-values will be presented but no multiplicity adjustment will be made. Analyses of mean changes from baseline to Week 12 and month 9 (Day 252) respectively will be conducted, as described below.

- The PGIC scale (see

• Step	Description
1A	Health Network sends email to prospective study participants.
1B	Pre-consent Participant clicks link to PRA Patient Experience Website and completes Technology Screening form and contact info submission to "opt in".
1C	PRA Patient Experience opt in triggers Patient ID creation in PRA mHP.
1D	Health Network + VI utilize the PRA mHP Web Application to update Pre-Consent Participant demographic info and assess eligibility.
1E	Care Innovations Zendesk Ticketing System automatically pulls information from PRA mHP for Pre-Consent Participants assessed as "Eligible" and creates an assigned open ticket.
1F	Care Innovations interacts with Zendesk Ticketing System to pick up assigned open tickets.
1G	Care Innovations interacts with PRA mHP Web Application to access Pre-Consent Participant contact information to schedule eConsent c
2A	Care Innovations initiates communication with Pre-consent Participant to schedule eConsent call and verify contact information (mobile number and address).
2B	Pre-consent Participant interacts with PRA mHP Participant Web Application and Mobile Application to complete pre-consent review.
2C	Care Innovations interacts with VI to coordinate and facilitate completion of eConsent call. Post-consent Participant moves to ePRO completion post eConsent call.
3A	PRA mHP sends questionnaire completion details to SCOUT for payment to participants.
3B	SCOUT sends reloadable payment card and payment to consented participants upon completion of study questionnaires.
3C	PRA mHP interacts with Endpoint IRT at Randomization via a two-way database integration.
3D	PRA mHP interacts with Marken's system via a two-way database integration.
3E	Marken schedules directly with Participant to deliver drug to Participant's home. Care Innovations sends study supplies Welcome Kit to Participant.
3F	During Welcome Call with Care Innovations, Participant is guided through the supplies in the Welcome Kit, which includes the Fitbit watch Participant interacts with Fitbit Mobile Application for Fitbit set-up and wears Fitbit to collect Biodata / ADL data.
3G	Once authorized within the PRA mHP Mobile Application, Fitbit data is retrieved by PRA mHP automatically through Fitbit API.
4A	PRA mHP sends Symphony encrypted, tokenized PII to match consented Participant in PRA mHP with their available medical claims data in Symphony.
4B	Synoma ID Matches identified in Symphony are sent to DAC
5A	Care Innovations manually submits participant-reported AEs directly to Janssen Safety (GMS).
5B	Janssen Safety (GMS) extracts participant-reported AEs and transfers data to PRA for upload into CCDS.
6A	Symphony Claims data is pulled into DAC.
6B	Care Innovations Zendesk ticketing data is sent to DAC via JSON payloads.
6C	PRA mHP data is pulled into DAC.
7A	DAC data is staged and moved into consolidation (CNS) layer, aka "PRA Data Lake".
7B	Consolidated DAC data tables are loaded into CCDS.
8A	Data Transfer from Endpoint is manually loaded into CCDS.
8B	Data Transfer from Marken is manually loaded into CCDS.

9A	RAW SAS datasets are extracted from CCDS for each data delivery.
9B	SDTM Datasets are generated from the RAW data tables for each data delivery.
9C	RAW SAS datasets are manually posted to LSAF for delivery to Janssen.
9D	SDTM SAS datasets are manually posted to LSAF for delivery to Janssen.
10A	Care Innovations manually enters drug resupply request into Endpoint IRT (if applicable)
10B	Endpoint notifies Marken of need for drug resupply (if applicable)

- Appendix 6: Patient Global Impression of Change (PGIC)) is a validated generic tool for assessment of overall change in the severity of illness following treatment. Participants rate how they feel now compared with how they felt before receiving study intervention on a 7-point scale where 1 is “Very much improved” and 7 is “Very much worse”. A mean ± SD PGIC score for each time point will be calculated by randomized intervention group. Distribution of PGIC score at Week 12 will also be presented. Based on the PGIC symmetric scale, a dichotomous scale of “Yes” or “No” will be derived. A favorable change is score of 1-3 = ’Yes’, which means there is significant improvement with the study intervention. If the response is 4-7 = ’No’, it is considered no significant change. Change in the KCCQ-TSS from baseline to Week 12 by PGIC response at Week 12 will be summarized by treatment group, and, also graphically displayed (i.e., x-axis will display categories of PGIC response at Week 12 and y-axis will display mean change scores from baseline to Week 12 in KCCQ-TSS by treatment group).
- Distribution of participants in PGI-S (see Appendix 7: Patient Global Impression of Severity (PGI-S)) response categories will be summarized by randomized intervention group at baseline and by Week 12. Mean change in PGI-S score between baseline and Week 12 will be summarized by randomized group. Change in the KCCQ-TSS from baseline to Week 12 by PGI-S response at Week 12 will be summarized.
- For daily activity measured by daily step counts, total active days (defined as Fitbit recorded ≥100 steps) and percent of active days to total days spent in the study analysis period will be summarized for each intervention group using mean and SD. Level of daily activity will also be described using categories of total step counts per day – 1) <3000 steps per day (low activity/sedentary), 2) 3000-5999 (moderate activity), and 3) ≥ 6000 steps per day (high activity). Associations between daily step count (categorized into activity levels) and baseline characteristics (eg, age, sex, race, T2DM status, EF type, etc.) will be examined using a 1-way ANOVA for continuous data values and the Fisher exact test for categorical data.
- Correlation between total step counts per day and clinical events (eg, # of outpatient intravenous therapies, # ED visits, # hospitalizations, etc.) will be described for the double-blind treatment period and total study duration separately. Relationships between change in step counts/floors climbed and incidence of reported clinical outcomes and PRO measures will be examined at the end of the double-blind treatment period and end of study separately.
- Daily floors climbed will be summarized by each intervention group for the double-blind treatment period and over the entire study duration separately.
- Descriptive statistics (ie, N, mean, SD, minimum and maximum) will be presented for total HRU and health economics data for each intervention group separately for the double-blind

treatment period and the observational (no intervention) period. HRUs and health economics data typically include information regarding participants using health services for managing any clinical events/illnesses, hospitalizations/ED visits, surgeries, medication issues/prescription changes, and costs incurred.

- Descriptive statistics will be presented for participant's satisfaction with his/her experience with the virtual design at the end of the study.
- Using data from claims, the number of outpatient intravenous therapies, ED visits, and hospitalizations by each intervention group will be summarized. These summaries will include descriptive statistics.
- Using data from the claims, time to first hospitalization/readmission for heart failure, time to first hospitalization/readmission for other events, and time to death will each be analyzed using a Cox's regression model both for the double-blind treatment period and the entire study duration and include terms for study intervention and stratification factor (HFrEF/HFpEF). Hazard ratios, corresponding 95% CIs, and associated p-values will be presented. Distribution of the times to first hospitalization/readmission for HF, time to first hospitalization/readmission for other events, and time to death will be displayed with Kaplan-Meier curves. Participants who do not experience any such events of interest during the study analysis period will be considered 'censored' in these analyses.

No adjustment for multiplicity will be performed for the exploratory analyses listed above.

6. SAFETY

Summaries of safety data will be based on the safety analysis set and will use claims data.

6.1. Adverse Events

All AE data summaries will be based on the safety analysis set except where stated otherwise. A treatment emergent AE is defined as an event that occurs after the first dose of study intervention (Study Day 1) and/or worsens in severity during the double-blind treatment period.

Adverse events self-reported to the Call Center will be assessed by Janssen Global Medical Safety (GMS) for start and end dates, causality and meeting SUSAR reporting requirements. If the specific dates pertaining to the self-reported AEs are unknown, there will be limited follow-up for resolution. For any events still ongoing at the end of the study, there will be no follow up (as the Call Center will be closed at that point) possible. Adverse events will be coded according to MedDRA and will be reported by preferred term for each randomized intervention group. Janssen Data Management will request a listing of all AEs/SAEs reported to the Call Center from the Janssen GMS. This listing will be shared with PRA (external vendor) to convert the information into the SDTM format and for inclusion in the final SDTM data package for summary by various subgroups (e.g., T2DM and non-T2DM).

Adverse events collected through the claims database will have dates of service provided (procedure dates, office visits, hospital admissions, etc.) but not actual start/end dates of events prior to claims being filed. Therefore, no imputation of AE start/end dates will be feasible on the claims data. Dates of service(s) rendered to participants during the study (i.e., starting from the time informed consent is signed until the end of double-blind treatment plus 30 days) for each

participant will be available. The claims data will utilize ICD codes to identify the AEs of interest which are defined in the protocol and for the final analysis. The AEs of interest include all severe hypoglycemic events, acute kidney injury, fractures, diabetic ketoacidosis, and lower limb amputation. In addition, hypotension, Fournier's gangrene, genital mycotic infections, urinary tract infections (including urosepsis and pyelonephritis), increased urination and hypersensitivity reactions (including angioedema and anaphylaxis) will be considered for coding and final analysis.

For the prohibited medications, analysis period will include data from the date of informed consent until the end of double-blind treatment period. For all other concomitant mediations, analysis period will include data from the date of informed consent until the end of the study. For the HRU data analysis, information collected from date of informed consent until the end of study will be considered in the final analysis.

Summaries (numbers and percentages) of participants having an AE of interest will be produced, including by participant's diabetes status (T2DM and non-T2DM) and by randomized intervention group. Discontinuations (and reported reasons) will be summarized by randomized intervention group based on available claims data.

There will be no reconciliation/analysis of AE data that are self-reported to the Call Center with the data obtained from the claims database.

A listing will be presented for treatment emergent hospitalizations and deaths for each randomized intervention group. AEs occurring between informed consent and the day prior to the first dose of study intervention will also be listed. This listing will be based on participants with informed consent into the study. Depending on the claims/pharmacy data, AEs occurring in participants who used prohibited (commercially available SGLT2is other than the study intervention, canagliflozin) will also be listed.

Based on pharmacy profiles of the participants after-consent in this study, the total number of participants concomitantly receiving UGT inducers during the double-blind treatment period will be summarized combined across both study intervention groups. If the number of such participants is >2% of the total 1,900 targeted for enrollment in this study, the users and non-users of UGT inducers will be summarized by each study intervention group as well and examined further (as necessary) for any relationship to key study outcomes (eg, PRO endpoints and clinical outcomes reported in claims).

6.2. Clinical Laboratory Tests

Not Applicable.

6.3. Vital Signs and Physical Examination Findings

Not Applicable.

6.4. Electrocardiogram

Not Applicable.

6.5. Other Safety Parameters

Not Applicable.

7. PHARMACOKINETICS/PHARMACODYNAMICS

Not Applicable.

8. BIOMARKERS

Not Applicable.

9. HEALTH ECONOMICS

As treatment of participants with symptomatic HF with JNJ-28431754 (canagliflozin)JNJ-28431754 (canagliflozin) versus placebo may result in lower utilization of hospitalization/readmissions due to HF or other reason, ED visits, the duration of stay at each hospitalization/readmission, and discharge destination will be summarized by each intervention group. Comparison will be made between groups by analyzing differences in hospitalizations/readmissions and ED visits using the Fisher exact test. Descriptive statistics (eg, mean, median, minimum, and maximum) will be provided for the total length of stay in hospitals for each intervention group. Cumulative distribution function of the time to first occurrence of hospitalization/readmission will be estimated by the Kaplan-Meier method and tested using a log-rank test, stratified by the type of HF ejection fraction (ie, HFrEF or HFpEF). In addition, a Cox proportional hazards mode with intervention and stratification factor as covariates will be fitted. A point-estimate along with 2-sided 95% CI for HR of the intervention effect (in terms of relative risk reduction (RRR) = 100 x [1-HR] %) will be provided. The cumulative event rate derived from Kaplan-Meier estimate will also be displayed graphically to evaluate the timing of event occurrence and the consistency of the treatment effect over time.

REFERENCES

1. Berglund P and Heeringa S. Multiple Imputation of Missing Data Using SAS®. 2014. Cary, NC: SAS Institute Inc. CDISC. “Analysis Data.
2. Dreyer RP, Jones PG, Kutty S, Spertus JA. Quantifying clinical change: discrepancies between patients' and providers' perspectives. Qual Life Res. 2016 Sep;25(9): 2213-2220.
3. Green, C., Porter C., Bresnahan D., and Spertus, J. 2000. Development and evaluation of the Kansas City Cardiomyopathy Questionnaire: a new health status measure for heart failure. Journal of American College of Cardiology. 2000 Apr; 35(5):1245-1255.
4. International Council For Harmonisation (ICH) of Technical Requirement for Pharmaceuticals for Human Use (final version, 2019): ICH Harmonised Guideline – Addendum on estimands and sensitivity analysis in clinical trials (to the guideline on Statistical Principles for Clinical Trials) - E9 (R1).
5. Khariton Y. et al. Association Between Sacubitril/Valsartan Initiation and Health Status Outcomes in Heart Failure with Reduced Ejection Fraction: Findings from the CHAMP-HF Registry. Data presented at the European Society of Cardiology Heart Failure (ESC-HF); 2018 May 26-29; Vienna, Austria.
6. Luo N, et al. Relationship between changing patient-reported outcomes and subsequent clinical events in patients with chronic heart failure: insights from HF-ACTION, European Journal of Heart Failure (2019) 21, 63-70.
7. McMurray JJV, Solomon SD, Inzucchi SE, et al; DAPA-HF Trial Committees and Investigators. Dapagliflozin in patients with heart failure and reduced ejection fraction. N Engl J Med. 2019 Sep 19.
8. Nassif ME, Windsor SL, Tang F, et al. Dapagliflozin Effects on Biomarkers, Symptoms, and Functional Status in Patients With Heart Failure With Reduced Ejection Fraction: The DEFINE-HF Trial. Circulation. 2019 Oct 29;140(18):1463-1476.
9. Pokharel Y, et al. Association of serial Kansas City Cardiomyopathy Questionnaire assessments with death and hospitalization in patients with heart failure with preserved and reduced ejection fraction. A secondary analysis of 2 randomized clinical trials. JAMA Cardiology. 2017 Dec 1; 2912):1315-1321.
10. Shoemaker M, Curtis AB, Vangsnes E, Dickinson, MG. Clinically Meaningful Change Estimates for the Six-Minute Walk Test and Daily Activity in Individuals With Chronic Heart Failure. Cardiopulm Phys Ther J, Sep 2013;24(3), 21-29.
11. Spertus, J, Jones P. Development and validation of a short version of the Kansas City Cardiomyopathy Questionnaire. Circulation: Cardiovascular Quality and Outcomes, 2015; 8:469-476.
12. Spertus J, Peterson E, Conard MW, et al. Monitoring clinical changes in patients with heart failure: a comparison of methods. Am Heart J 2005;150(4):707-715.
13. Thorup C, Hansen J, Gronkjaer M, et al. Cardiac Patients’ Walking Activity Determined by a Step Counter in Cardiac Telerehabilitation: Data From the Intervention Arm of A Randomized Controlled Trial. J Med Internet Res 2016 Apr; 18(4); e69.
14. Vetrovsky T, Siranec M, Parenica J, et al. Effect of a 6-month pedometer-based walking intervention on functional capacity in patients with chronic heart failure with reduced (HFrEF) and with preserved (HFpEF) ejection fraction: study protocol for two multicenter randomized controlled trials, J Transl Med, 2017; 15(1):153.
15. Gail, M. H., and Simon, R. (1985). “Tests for Qualitative Interactions between Treatment Effects and Patient Subsets.” *Biometrics* 41:361–372.
16. Mehta, L. et al. (2015). Identifying an important change estimate for the multiple sclerosis walking scale-12 (MSWS-12v1) for interpreting clinical trial results, Multiple Sclerosis Journal – Experimental, Translational, Clinical” 1:1-9

APPENDICES

Appendix 1: The Kansas City Cardiomyopathy Questionnaire (KCCQ)

The KCCQ (Appendix 1: The Kansas City Cardiomyopathy Questionnaire (KCCQ)) is a well-established PRO for use in patients with HF, with published validity and reliability and was recently endorsed by the Center for Devices and Radiological Health (CDRH, US FDA) as a Certified Outcome Assessment through its Medical Device Development Tools Program and is currently under review at the Center for Drug Evaluation and Research (CDER, US FDA).

The Kansas City Cardiomyopathy Questionnaire (KCCQ) is a 23-item self-administered questionnaire developed to independently measure the patient's perception of their health status, which includes heart failure symptoms, impact on physical and social function, and how their heart failure impacts their quality of life (QOL) within a 2-week recall period. This questionnaire requires, on average, 4 to 6 minutes to complete, with a resulting score of 0 to 100, and higher scores indicating better health.

The intent of the study is to enroll participants with NYHA class II and III. As it would be difficult to verify NYHA class with claims and EHR data, the sponsor will use a KCCQ overall summary score at baseline of >40 and <80, which is highly correlated to class II and III in HFrEF and HFpEF based on the research published by Joseph, et al.[6] In addition to the KCCQ overall summary score, a baseline KCCQ TSS score will also be calculated for each participant.

The 23-item KCCQ quantifies 7 domains of patients' HF-related health status:

- Physical Limitation (6 items)
- Symptom Stability (1 item)
- Symptom Frequency (4 items)
- Symptom Burden (3 items)
- Self-Efficacy (2 items)
- Quality of Life (3 items)
- Social Limitations (4 items)

Item responses are coded sequentially (1, 2, 3, etc.) from worst to best status. Scores are generated for each domain and scaled from 0 to 100, with 0 denoting the worst and 100 the best possible status. In addition, three summary scores are calculated: **Total Symptom score** (average of Symptom Frequency and Symptom Burden), **Clinical Summary score** (average of Physical Limitation and Total Symptoms), and **Overall Summary score** (average of Physical Limitation, Total Symptoms, Quality of Life, and Social Limitation).

Among the 7 domains and 3 summary scores of KCCQ described above, this study will focus on analyzing only two individual domains (physical limitation and quality of life) and three summary scores (clinical summary, overall summary, and total symptom).

Below is a representative example of the scale questions that will be used in this study.

The KC Cardiomyopathy Questionnaire

The following questions refer to your **heart failure** and how it may affect your life. Please read and complete the following questions. There are no right or wrong answers. Please mark the answer that best applies to you.

1. **Heart failure** affects different people in different ways. Some feel shortness of breath while others feel fatigue. Please indicate how much you are limited by **heart failure** (shortness of breath or fatigue) in your ability to do the following activities over the past 2 weeks.

Place an **X** in one box on each line

Activity	Extremely Limited	Quite a bit Limited	Moderately Limited	Slightly Limited	Not at all Limited	Limited for other reasons or did not do the activity
Dressing yourself	❑	❑	❑	❑	❑	❑
Showering/Bathing	❑	❑	❑	❑	❑	❑
Walking 1 block on level ground	❑	❑	❑	❑	❑	❑
Doing yardwork, housework or carrying groceries	❑	❑	❑	❑	❑	❑
Climbing a flight of stairs without stopping	❑	❑	❑	❑	❑	❑
Hurrying or jogging (as if to catch a bus)	❑	❑	❑	❑	❑	❑

2. Compared with 2 weeks ago, have your symptoms of **heart failure** (shortness of breath, fatigue, or ankle swelling) changed?

My symptoms of **heart failure** have become…

Much worse	Slightly worse	Not changed	Slightly better	Much better	I've had no symptoms over the last 2 weeks
❑	❑	❑	❑	❑	❑

Copyright ©1992 –2005 John Spertus, MD, MPH
Original US English

3. Over the past 2 weeks, how many times did you have **swelling** in your feet, ankles or legs when you woke up in the morning?

Every morning	3 or more times a week, but not every day	1-2 times a week	Less than once a week	Never over the past 2 weeks
❑	❑	❑	❑	❑

4. Over the past 2 weeks, how much has **swelling** in your feet, ankles or legs bothered you?

It has been ...

**Extremely** bothersome	**Quite a bit** bothersome	**Moderately** bothersome	**Slightly** bothersome	**Not at all** bothersome	I've had **no swelling**
❑	❑	❑	❑	❑	❑

5. Over the past 2 weeks, on average, how many times has **fatigue** limited your ability to do what you want?

All of the time	Several times per day	At least once a day	3 or more times per week but not every day	1-2 times per week	Less than once a week	Never over the past 2 weeks
❑	❑	❑	❑	❑	❑	❑

6. Over the past 2 weeks, how much has your **fatigue** bothered you?

It has been ...

**Extremely** bothersome	**Quite a bit** bothersome	**Moderately** bothersome	**Slightly** bothersome	**Not at all** bothersome	I've had **no fatigue**
❑	❑	❑	❑	❑	❑

7. Over the past 2 weeks, on average, how many times has **shortness of breath** limited your ability to do what you wanted?

All of the time	Several times per day	At least once a day	3 or more times per week but not every day	1-2 times per week	Less than once a week	Never over the past 2 weeks
❑	❑	❑	❑	❑	❑	❑

Copyright ©1992 –2005 John Spertus, MD, MPH
Original US English

8. Over the <u>past 2 weeks</u>, how much has your **shortness of breath** bothered you?

It has been ...

**Extremely** bothersome	**Quite a bit** bothersome	**Moderately** bothersome	**Slightly** bothersome	**Not at all** bothersome	I've had **no shortness of breath**
❑	❑	❑	❑	❑	❑

9. Over the <u>past 2 weeks</u>, on average, how many times have you been forced to sleep sitting up in a chair or with at least 3 pillows to prop you up because of **shortness of breath**?

Every night	3 or more times a week, but not every day	1-2 times a week	Less than once a week	Never over the past 2 weeks
❑	❑	❑	❑	❑

10. **Heart failure** symptoms can worsen for a number of reasons. How sure are you that you know what to do, or whom to call, if your **heart failure** gets worse?

**Not at all** sure	**Not very** sure	**Somewhat** sure	**Mostly** sure	**Completely** sure
❑	❑	❑	❑	❑

11. How well do you understand what things you are able to do to keep your **heart failure** symptoms from getting worse? (for example, weighing yourself, eating a low salt diet etc.)

Do not understand at all	Do not understand very well	Somewhat understand	Mostly understand	Completely understand
❑	❑	❑	❑	❑

12. Over the <u>past 2 weeks</u>, how much has your **heart failure** limited your enjoyment of life?

It has **extremely** limited my enjoyment of life	It has limited my enjoyment of life **quite a bit**	It has **moderately** limited my enjoyment of life	It has **slightly** limited my enjoyment of life	It has **not limited** my enjoyment of life at all
❑	❑	❑	❑	❑

13. If you had to spend the rest of your life with your **heart failure** the way it is <u>right now</u>, how would you feel about this?

Not at all satisfied	Mostly dissatisfied	Somewhat satisfied	Mostly satisfied	Completely satisfied
❑	❑	❑	❑	❑

Copyright ©1992 –2005 John Spertus, MD, MPH
Original US English

14. Over the past 2 weeks, how often have you felt discouraged or down in the dumps because of your **heart failure**?

I felt that way **all of the time**	I felt that way **most of the time**	I **occasionally** felt that way	I **rarely** felt that way	I **never** felt that way
❑	❑	❑	❑	❑

15. How much does your **heart failure** affect your lifestyle? Please indicate how your **heart failure** may have limited your participation in the following activities over the past 2 weeks.

Please place an **X** in one box on each line

**Activity**	**Severely** limited	Limited **quite a bit**	**Moderately** limited	**Slightly** limited	**Did not** limit at all	Does not apply or did not do for other reasons
Hobbies, recreational activities	❑	❑	❑	❑	❑	❑
Working or doing household chores	❑	❑	❑	❑	❑	❑
Visiting family or friends out of your home	❑	❑	❑	❑	❑	❑
Intimate relationships with loved ones	❑	❑	❑	❑	❑	❑

Copyright ©1992 –2005 John Spertus, MD, MPH
Original US English

Appendix 2: Participant Satisfaction Survey

Appendix 2 provides a representative example of the questions that will be provided to the participant.

For each of the statements below, please indicate your level of agreement:	Strongly Disagree	Disagree	Neutral	Agree	Strongly Agree
Trying something new to possibly treat my heart failure was important to me.					
The virtual study was easy for me to sign up for and get started.					
The Fitbit was easy to set up and use.					
The study app was easy to set up on my smartphone.					
The questionnaires were easy to complete on my smartphone.					
The reminders to complete the questionnaires were helpful.					
The study Fitbit provided helpful information to me.					
It was easy to reach the study team when I had questions or concerns.					
I would recommend participating in a study like this to my friends and family.					

Appendix 3: Schedule of Activities

Period	Screening			Double-Blind Treatment				No Treatment		Notes
	Screening	Baseline								
Day	Within 21 days before Day 1	Within 7 days before Day 1	1 (first dose)	14±3	28±3	42±3	84±3 End of Treatment	85-251-±7	252-±7 End of Study	Day -21 to 0=Screening; Day 1 = first dose, Day 84= end of treatment; Day 252=End of Study
Week				2	4	6	12	13-36		Week 12 is equivalent to Day 84 of treatment
Month			0				3	6	9	Month 3 is equivalent to Day 84 of treatment
**Study Procedure**										
Screening/ Administrative										
Electronic Informed consent[a]	X									
Review medical history	X									
Inclusion/ exclusion criteria[b]	X		X							All inclusion/exclusion verification should be completed within the 21-day screening period and before randomization
KCCQ baseline overall summary score >40 and <80			X							
Study Intervention Administration										
Randomization			X							
Dispense/administer study intervention			←——————————————————→							
Participant-reported adherence via app - weekly			←——————————————————→							
Assessments										
KCCQ				X	X	X	X	X[c]	X	Kansas City Cardiomyopathy Questionnaire
PGIC				X	X	X	X	X[c]	X	Patient Global Impression of Change
PGI-S			X	X	X	X	X	X[c]	X	Patient Global Impression of Severity
Step count and floors climbed			←——————————————————————————→							
Claims data collection[d]			←——————————————————————————→							

Period	Screening			Double-Blind Treatment				No Treatment		Notes
	Screening	Baseline								
Day	Within 21 days before Day 1	Within 7 days before Day 1	1 (first dose)	14±3	28±3	42±3	84±3 End of Treatment	85-251-±7	252-±7 End of Study	Day -21 to 0=Screening; Day 1 = first dose, Day 84= end of treatment; Day 252=End of Study
Week				2	4	6	12	13-36		Week 12 is equivalent to Day 84 of treatment
Month			0				3	6	9	Month 3 is equivalent to Day 84 of treatment
Participant Satisfaction Survey (optional)									X	
Health Resource Utilization and Health Economics										
HHF for any reason and duration			←→							Hospitalizations for heart failure
Emergency department visits			←→							
Readmissions and duration			←→							
Ongoing Review of Participant Data										
Prohibited medications and new diagnoses			←→							
Reporting of adverse events[e]	←→									

Appendix 4: Usage of the Analysis Sets

Appendix 4 presents an overview of the different analysis sets and their usage.

Analyses/data displays	Screened analysis set	All randomized analysis set	Safety analysis set	Per protocol set
Patient disposition	✓			
Demographics		✓		✓
Baseline characteristics		✓		✓
Medical history		✓		
Treatment exposure			✓	✓
Effectiveness: Primary endpoint		✓		✓
Effectiveness: Secondary endpoints		✓		✓
Effectiveness; Exploratory endpoints		✓		
Safety endpoints			✓	
All other patient listings	✓			

Appendix 5: CHIEF-HF Study Data Flow Diagram

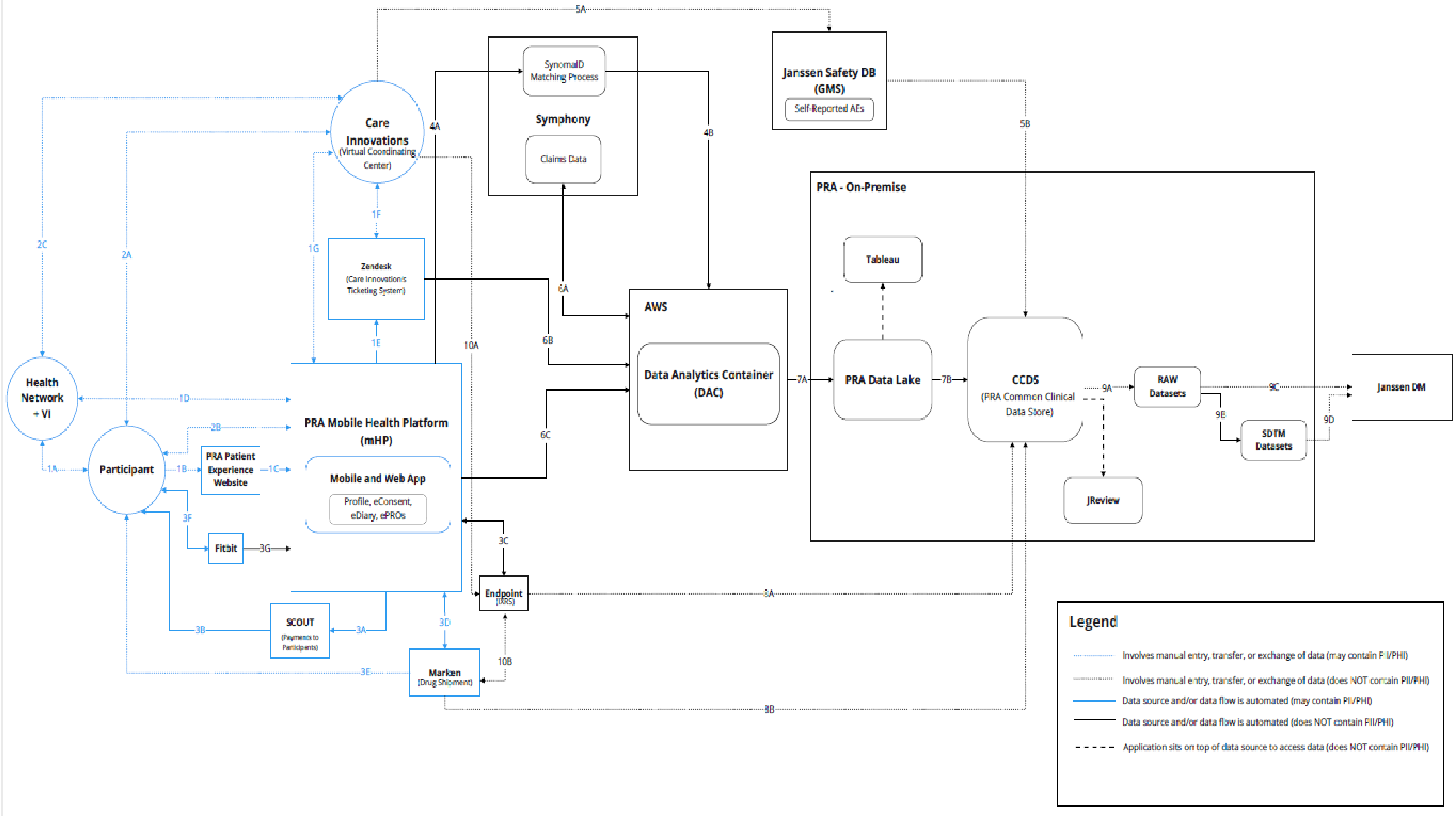

Step	Description
1A	Health Network sends email to prospective study participants.
1B	Pre-consent Participant clicks link to PRA Patient Experience Website and completes Technology Screening form and contact info submission to "opt in".
1C	PRA Patient Experience opt in triggers Patient ID creation in PRA mHP.
1D	Health Network + VI utilize the PRA mHP Web Application to update Pre-Consent Participant demographic info and assess eligibility.
1E	Care Innovations Zendesk Ticketing System automatically pulls information from PRA mHP for Pre-Consent Participants assessed as “Eligible” and creates an assigned open ticket.
1F	Care Innovations interacts with Zendesk Ticketing System to pick up assigned open tickets.
1G	Care Innovations interacts with PRA mHP Web Application to access Pre-Consent Participant contact information to schedule eConsent call.
2A	Care Innovations initiates communication with Pre-consent Participant to schedule eConsent call and verify contact information (mobile number and address).
2B	Pre-consent Participant interacts with PRA mHP Participant Web Application and Mobile Application to complete pre-consent review.
2C	Care Innovations interacts with VI to coordinate and facilitate completion of eConsent call. Post-consent Participant moves to ePRO completion post eConsent call.
3A	PRA mHP sends questionnaire completion details to SCOUT for payment to participants.
3B	SCOUT sends reloadable payment card and payment to consented participants upon completion of study questionnaires.
3C	PRA mHP interacts with Endpoint IRT at Randomization via a two-way database integration.
3D	PRA mHP interacts with Marken's system via a two-way database integration.
3E	Marken schedules directly with Participant to deliver drug to Participant's home. Care Innovations sends study supplies Welcome Kit to Participant.
3F	During Welcome Call with Care Innovations, Participant is guided through the supplies in the Welcome Kit, which includes the Fitbit watch. Participant interacts with Fitbit Mobile Application for Fitbit set-up and wears Fitbit to collect Biodata / ADL data.
3G	Once authorized within the PRA mHP Mobile Application, Fitbit data is retrieved by PRA mHP automatically through Fitbit API.
4A	PRA mHP sends Symphony encrypted, tokenized PII to match consented Participant in PRA mHP with their available medical claims data in Symphony.
4B	Synoma ID Matches identified in Symphony are sent to DAC
5A	Care Innovations manually submits participant-reported AEs directly to Janssen Safety (GMS).
5B	Janssen Safety (GMS) extracts participant-reported AEs and transfers data to PRA for upload into CCDS.
6A	Symphony Claims data is pulled into DAC.
6B	Care Innovations Zendesk ticketing data is sent to DAC via JSON payloads.
6C	PRA mHP data is pulled into DAC.
7A	DAC data is staged and moved into consolidation (CNS) layer, aka "PRA Data Lake".
7B	Consolidated DAC data tables are loaded into CCDS.
8A	Data Transfer from Endpoint is manually loaded into CCDS.
8B	Data Transfer from Marken is manually loaded into CCDS.
9A	RAW SAS datasets are extracted from CCDS for each data delivery.
9B	SDTM Datasets are generated from the RAW data tables for each data delivery.
9C	RAW SAS datasets are manually posted to LSAF for delivery to Janssen.
9D	SDTM SAS datasets are manually posted to LSAF for delivery to Janssen.
10A	Care Innovations manually enters drug resupply request into Endpoint IRT (if applicable)
10B	Endpoint notifies Marken of need for drug resupply (if applicable)

Appendix 6: Patient Global Impression of Change (PGIC)

Appendix 6 provides a representative example of the scale questions that will be used in this study.

Since the start of the treatment you've received in this study, your heart failure symptoms are: (select one response)

1. Very much improved
2. Somewhat improved
3. A little improved
4. No change
5. A little worse
6. Somewhat worse
7. Very much worse

Appendix 7: Patient Global Impression of Severity (PGI-S)

Appendix 7 provides a representative example of the scale question that will be used in this study.

Considering all aspects of your heart failure symptoms right now, would you say your heart failure symptoms are: (select one response)

1. None
2. Mild
3. Moderate
4. Severe
5. Very Severe

Final Statistical Analysis Plan

Janssen Research & Development, LLC

Statistical Analysis Plan

Canagliflozin: Impact on Health Status, Quality of Life, and Functional Status in Heart Failure)

Protocol 28431754HFA3002; Phase 3B

JNJ-28431754 (canagliflozin) (INVOKANA)

Status: FINAL

Date: 19 July 2021

Prepared by: Janssen Research & Development, LLC

Document No.: EDMS-RIM-294497, 1.0

Compliance: The study described in this report was performed according to the principles of Good Clinical Practice (GCP).

Confidentiality Statement

The information in this document contains trade secrets and commercial information that are privileged or confidential and may not be disclosed unless such disclosure is required by applicable law or regulations. In any event, persons to whom the information is disclosed must be informed that the information is privileged or confidential and may not be further disclosed by them. These restrictions on disclosure will apply equally to all future information supplied to you that is indicated as privileged or confidential.

TABLE OF CONTENTS

TABLE OF CONTENTS 2

LIST OF IN-TEXT TABLES 3

LIST OF IN-TEXT FIGURES 3

AMENDMENT HISTORY 4

ABBREVIATIONS 5

1. INTRODUCTION 8
1.1. Objectives 8
1.2. Study Design 9
1.3. Statistical Hypotheses for Trial Objectives 10
1.4. Sample Size Justification 11
1.5. Randomization and Blinding 12
1.6. Study Data Flow 13

2. GENERAL ANALYSIS DEFINITIONS 14
2.1. Time Windows 14
2.2. Pooling Algorithm for Analysis Centers 16
2.3. Analysis Sets 16
2.3.1. All Randomized Analysis Set 16
2.3.2. Full Analysis Set (FAS) 16
2.3.3. Per Protocol Analysis Set 16
2.3.4. Safety Analysis Set 17
2.4. Definition of Subgroups 17
2.5. Study Day and Relative Day 17
2.6. Baseline, End of Treatment, and End of Study 17
2.7. Imputation Rules for Missing AE Date/Time of Onset/Resolution 18

3. INTERIM ANALYSIS AND DATA MONITORING COMMITTEE REVIEW 18

4. SUBJECT INFORMATION 18
4.1. Demographics and Baseline Characteristics 18
4.2. Disposition Information 19
4.3. Treatment Compliance 19
4.4. Extent of Exposure 20
4.5. Protocol Deviations 20
4.6. Prior and Concomitant Medications 20

5. EFFICACY 20
5.1. Analysis Specifications 20
5.1.1. Level of Significance 21
5.1.2. Data Handling Rules 21
5.2. Primary Efficacy Endpoint 22
5.2.1. Definition 22
5.2.2. Estimand 22
5.2.3. Analysis Methods 23
5.3. Secondary Endpoints 26
5.3.1. Definition 26
5.3.2. Estimand 26
5.3.3. Analysis Methods 27
5.4. Exploratory Variables 27
5.4.1. Definition 27
5.4.2. Analysis Methods 28

6. SAFETY **29**
6.1. Adverse Events 29
6.2. Clinical Laboratory Tests 31
6.3. Vital Signs and Physical Examination Findings 31
6.4. Electrocardiogram 31
6.5. Other Safety Parameters 31

7. PHARMACOKINETICS/PHARMACODYNAMICS **31**

8. BIOMARKERS **31**

9. HEALTH ECONOMICS **31**

REFERENCES **32**

APPENDICES **34**
Appendix 1: The Kansas City Cardiomyopathy Questionnaire (KCCQ) 34
Appendix 2: Participant Satisfaction Survey 39
Appendix 3: Schedule of Activities 40
Appendix 4: Usage of the Analysis Sets 42
Appendix 5: CHIEF-HF Study Data Flow Diagram 43
Appendix 6: Patient Global Impression of Change (PGIC) 45
Appendix 7: Patient Global Impression of Severity (PGI-S) 46

1. PHYSICAL LIMITATION **46**

2. SYMPTOM STABILITY **46**

3. SYMPTOM FREQUENCY **47**

4. SYMPTOM BURDEN **48**

5. TOTAL SYMPTOM SCORE **48**

6. SELF-EFFICACY **48**

7. QUALITY OF LIFE **49**

8. SOCIAL LIMITATION **49**

9. OVERALL SUMMARY SCORE **50**

10. CLINICAL SUMMARY SCORE **50**

LIST OF IN-TEXT TABLES

Table 1: Measurement Windows 15
Table 2: Demographic Variables 18

LIST OF IN-TEXT FIGURES

Figure 1: Schematic Overview of the Study 10

AMENDMENT HISTORY

1st Amendment (December 30, 2020)

Final changes (July 1, 2021)

ABBREVIATIONS

AE	adverse event
API	application program interface
ANOVA	analysis of variance
CDF	cumulative distribution function
CI	confidence interval
CSR	clinical study report
ED	emergency department
EF	ejection fraction
EHR	electronic health record
EOS	end of study
EOT	end of treatment
ES	effect size
FAS	Full analysis set
FDA	Food and Drug Administration
HF	heart failure
HFpEF	heart failure preserved ejection fraction
HFrEF	heart failure reduced ejection fraction
HRU	healthcare resource utilization
ICD	International Statistical Classification of Diseases and Related Health Problems
ICH	International Conference on Harmonisation
IHN	integrated health networks
ITT	intent to treat
IxR	interactive web response system
KCCQ	Kansas City Cardiomyopathy Questionnaire
KCCQ-TSS	Kansas City Cardiomyopathy Questionnaire – Total Symptom Score
LOCF	last observation carried forward
LS	least squares
MedDRA	Medical Dictionary for Regulatory Activities
MMRM	mixed model of repeated measurement
MI	multiple imputation
MID	minimum detectable
NYHA	New York Heart Association
PGIC	Patient Global Impression of Change
PGI-S	Patient Global Impression of Severity
PI	principal investigator
PP	per protocol
SAE	serious adverse event
SAP	statistical analysis plan
SAS	Statistical Analysis System
SD	standard deviation
SE	standard error
SGLT2	sodium glucose cotransporter-2 inhibitor
SUSAR	suspected unexpected serious adverse reaction
T2DM	type 2 diabetes mellitus
TEAE	treatment-emergent adverse event
UGT	UDP-Glucuronosyltransferase
US	United States

Amendment Page – Summary of Changes (December 30, 2020)

Section #	Original Text/Figure	Amended Text/Figure	Rationale
1.2	Figure 1	Figure 1	Changed entry criterion for KCCQ <80 incorporated
1.4	Added text	After approximately 10 months, the planned enrollment in this study is not expected to reach the target of 1900 participants by the end of January 30, 2021 (the scheduled end of study enrollment window). Instead, the final anticipated enrollment may be in the range of about 400-480 participants. Consequently, a reduction in total sample size of this magnitude will have impact on statistical power at different effect sizes, as shown by the figure below. A large effect size was reported in the recently concluded DEFINE-HF study[17]. Additional KCCQ domains were also reported in this study with robust improvements over baseline[17,18].	Statement about lower than expected sample size enrollment and its impact on statistical power
2.3.2.	Added full analysis set (FAS) definition	This includes all randomized participants who have received at least one dose of study intervention/medication and have at least one post-baseline KCCQ measurement.<br><br>Analyses of the primary, secondary, and exploratory endpoints will be based on the full analysis set (FAS).	Primary, secondary, and exploratory endpoints will be based on FAS (earlier version stated as based on all randomized set)
5.2.2.	Population: all randomized set	Population: FAS - all randomized participants with HF (who either have HFrEF or HFpEF, regardless of T2DM status) with at least one dose of study intervention and at least 1 post-baseline KCCQ measurement	Changed analysis population to FAS
5.2.3.	Added to the analysis method	Mortality in this short-duration trial is expected to be low and to be comparable between groups and will be ignored in the primary analyses of mean differences in changes in KCCQ Total Symptom scores, but can be included in the responder analyses by assigning them to the lowest response (greatest deterioration) category. Should there be a >5% mortality rate and if it is unequally distributed between treatment arms, then joint modeling of survival and health status will be performed. A win-ratio approach[19,20] may also be considered by analyzing the KCCQ-TSS score as a composite, rank-based outcome, incorporating participant vital status at 12	Added to the analysis method regarding missing data due to deaths

		weeks along with a change in score from baseline to 12 weeks in surviving participants. This essentially is the rank analysis of covariance method, with a corresponding win ratio used to estimate the magnitude of treatment effect.	
5.3.2.	Population: all randomized set	Population: FAS - all randomized participants with HF (who either have HFrEF or HFpEF, regardless of T2DM status) with at least one dose of study intervention and at least 1 post-baseline KCCQ measurement	Changed analysis population to FAS
5.3.3.	Added to analysis method	The first key secondary endpoint will be analyzed following the same approach as employed for the primary effectiveness endpoint (i.e. using an MMRM model).	Added to analysis method regarding daily step counts from two-sample t-test to MMRM analysis
References	Added references	Nassif, M. et al. (2019). Dapagliflozin Effects on Biomarkers, Symptoms, and Functional Status in Patients With Heart Failure With Reduced Ejection Fraction - The DEFINE-HF Trial. *Circulation*, Vol. 140, No. 18, 1463-1476<br><br>Bhatt, D. et al. (2020). Sotagliflozin in Patients with Diabetes and Recent Worsening Heart Failure. *N Engl J Med*, 2020; Nov 16<br><br>Gasparyan, S. et al. (2019). Adjusted Win Ratio with Stratification: Calculation Methods and Interpretation, *Stat.ME*<br><br>Wang, D. and Pocock, S. (2016). A win-ratio approach to comparing continuous non-normal outcomes in clinical trials, *Pharmaceutical Statistics*, online 11 March 2016	Added more references pertaining to sample size section and analysis methods such as win-ratio

1. INTRODUCTION

This Statistical Analysis Plan (SAP) describes the planned analysis and reporting for the 28431754HFA3002 **(Canagliflozin: Impact on Health Status, Quality of Life, and Functional Status in Heart Failure – CHIEF-HF)** study. This Phase 3b study is being conducted in the United States (US) in participants with symptomatic HF stratified by HF (reduced ejection fraction [HFrEF] or preserved ejection fraction [HFpEF]) and compares the effectiveness of canagliflozin 100 mg versus placebo for improvement in HF symptoms measured by the Kansas City Cardiomyopathy Questionnaire - Total Symptom Score (KCCQ-TSS) (see Appendix 1: The Kansas City Cardiomyopathy Questionnaire (KCCQ)) after 12 weeks. This document contains details of the relevant definitions, data handling conventions and methods for analyses. The document will be finalized prior to database lock.

This SAP is based on the clinical study protocol (finalized first on 7 November, and subsequently amended on 7 February 2020 (Protocol **28431754HFA3002 Amendment 1**, dated **07 February 2020 - EDMS-ERI-154820237, version 3.0)** by Janssen Scientific Affairs, LLC) that provides details on the conduct of this study and operational aspects of clinical assessments and timing for completing a participant in this study.

Based on the last protocol amendment, changes were made to this SAP on December 30, 2020 and are listed on the preceding amendment page in this document. A final version of this SAP with editorial changes is posted on 1 July 2021.

1.1. Objectives

Primary Objective

The primary objective is to determine the superiority of the effectiveness of canagliflozin 100 mg daily versus placebo in participants with symptomatic HF in improving the overall KCCQ-TSS.

Secondary Objectives

The first key secondary objective is:

- to determine the superiority of the effectiveness of canagliflozin 100 mg daily versus placebo in improving the total daily step count.

The second key secondary objective is:

- to determine the superiority of the effectiveness of canagliflozin 100 mg daily versus placebo in improving the KCCQ individual domain scores (physical limitation, quality of life, clinical summary, and overall).

Exploratory Objectives

The exploratory objectives are to assess the effectiveness of canagliflozin 100 mg daily versus placebo in participants with symptomatic HF by:

- responses on the Patient Global Impression of Change (PGIC) and the Patient Global Impression of Severity (PGI-S)
- healthcare resource utilization (HRU) and health economics data
- associations (correlations and/or categorical association measures) between digital markers (step count and floors climbed), and clinical events such as need for outpatient intravenous therapies, emergency department (ED) visits, and hospitalizations
- daily floors climbed
- time to first hospitalization/readmission for HF
- time to first hospitalization/readmission for other events
- time to death
- the participant's satisfaction with their experience with the virtual design at the end of the study (see Appendix 2: Participant Satisfaction Survey).

1.2. Study Design

OVERALL DESIGN

This is a randomized, double-blind, placebo-controlled, decentralized, virtual, superiority study conducted in the US in participants with symptomatic HF (stratified by heart failure with HFrEF or HFpEF). This study examines the improvement in the KCCQ-TSS after 12 weeks of treatment with canagliflozin 100 mg or placebo. The study will continue for an additional 6 months without further study intervention to collect participant follow-up data from the smartphone, Fitbit, and claims in a real-world setting. At the end of the 12-week, double-blind treatment period, participants will be informed about their actual treatment allocation, whether they had been randomized to canagliflozin or placebo.

The study will enroll participants from large integrated health networks (IHNs) and physician practices in the US with the participants conducting many of their study-related activities through an app on a smartphone and will provide daily activity data recorded by using a Fitbit device. There will be no in-person clinic visits required by the study (see Appendix 3: Schedule of Activities).

The study consists of a screening period, a 12-week double-blind treatment period, and a 6-month follow-up (no intervention) observational period for each participant. Approximately 1,900 participants are planned for randomization into the study in a 1:1 ratio to canagliflozin 100 mg daily or placebo groups (950 in each study intervention group). After Week 12 (i.e., the end of the double-blind treatment period), the study will continue for an additional 6 months when study intervention will no longer be provided. Randomization will be performed using the interactive web response system (IxR). Participants will be stratified based on HF ejection fraction (EF) type – HFrEF or HFpEF.

Data collection from the smartphone, Fitbit device, and claims will continue until Month 9 for all endpoints. Specifically, study assessments include the following: KCCQ, PGIC, PGI-S, and an

optional participant satisfaction survey at end of study (via smartphone), step count and floors climbed (via the Fitbit device), medical and pharmacy claims data (including up to 36 months history for eligibility confirmation), HRU and health economics (including hospitalization/readmission due to HF or other reason, ED visits, the duration of stay at each hospitalization/readmission), review of claims for prohibited medications and new diagnoses that may require discontinuation, and adverse events. In addition, information related to outpatient IV diuretics, inotropes and vasodilators and in-patient IV therapies as well will be considered relevant. Note that claims data that involves up to 36 months history to confirm eligibility into this study will not be part of the study database for analysis.

Participants will be of any gender, 18 years of age or older, and will have clinically stable, symptomatic HF with or without type 2 diabetes mellitus (T2DM) with a KCCQ overall summary score <80 prior to randomization, which represents New York Heart Association (NYHA) II and III participants.

Figure 1: Schematic Overview of the Study

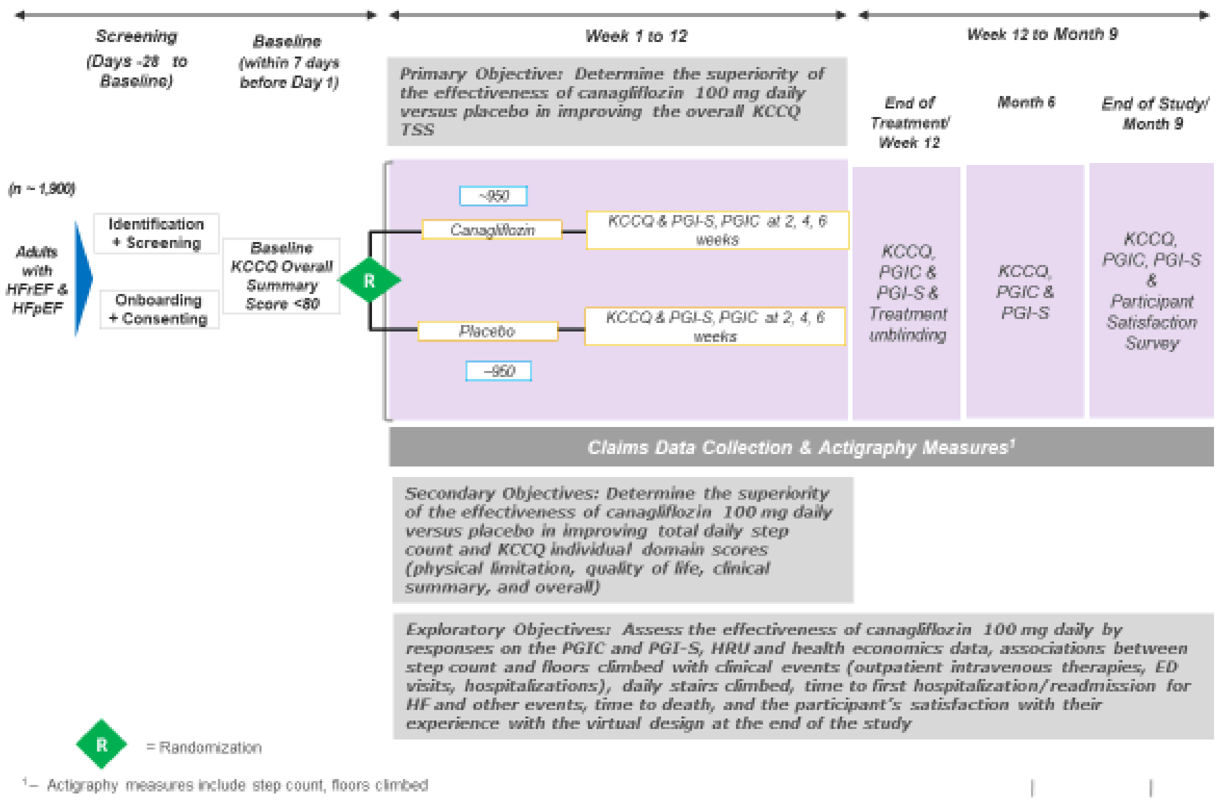

1.3. Statistical Hypotheses for Trial Objectives

The primary effectiveness endpoint is the change in the KCCQ-TSS from baseline to Week 12. The null hypothesis to be tested here is that there is no difference between canagliflozin 100 mg and placebo in the treatment of symptomatic HF based on the primary effectiveness endpoint. For

the first key secondary effectiveness endpoint of change in daily step count from baseline to Week 12, the null hypothesis to be tested is that canagliflozin 100 mg is not different from placebo with respect to the mean change in daily step count.

The two hypotheses described above will be tested using a fixed-sequence (hierarchical) testing procedure to control the familywise type I error rate at 5% as follows:

1. (primary effectiveness endpoint): change from baseline to Week 12 in KCCQ-TSS for canagliflozin 100 mg versus the placebo
2. (first key secondary endpoint): change from baseline to Week 12 in daily step count for canagliflozin 10 mg versus placebo.

If the result of the 2-sided p-value from the first hypothesis (i.e., primary effectiveness endpoint) test is ≤0.05, then the second hypothesis test result will be interpreted inferentially at the 5% significance level. This sequential testing approach will control the familywise type I error rate at 5%.

Hypotheses related to the second key secondary effectiveness endpoint (i.e., KCCQ individual domain scores defined by changes in physical limitation, quality of life, clinical summary, and overall summary from baseline to Week 12) as well as other exploratory endpoints will be tested at the nominal significance level (5%) without adjustment for multiplicity (or controlling type I error rate).

1.4. Sample Size Justification

The primary effectiveness endpoint is the change from baseline in the KCCQ-TSS at the end of the 12-week double-blind treatment period. Although a 5-point improvement in the KCCQ-TSS is generally viewed as clinically meaningful,[2,12] recent studies[7,8] have shown that improvements in the range of 3 to 5 points on the KCCQ-TSS are equally meaningful in the overall HF-related health status measured by the KCCQ over a 12-week time period. In considering the clinical significance of scores, it is important to separate mean changes for groups of participants, as compared with intra-individual changes. On the TSS, there are 7 items (4 for symptom frequency and 3 for severity/burden).[3,11] Shifting a response on KCCQ questions 5 and 7 shifts the TSS score by 2.083 points on a 100-point scale. A shift in response for questions 3 and 9 shifts the score by 3.125 points and for items 4, 6, and 8, shifts the score by 4.166 points, assuming no missing responses. Thus, an intra-individual change of at least 2 categories (3 if the only change is in items 5 and 7) would exceed the 5-point threshold that has the been shown to be clinically significant. When analyzing groups of participants, the mean difference across the entire population includes a distribution of participants with different magnitudes of individual change and a 3- to 5-point difference between groups reflects a significantly larger proportion of participants in one group, as compared with the other, who have clinically important improvements in their TSS.

Thus, based on the totality of evidence evaluating the clinical significance of the KCCQ,[5,6,9,14] a between group difference of 3 points is considered clinically meaningful and the current study is powered to detect a mean change from baseline of 3 points in the KCCQ-TSS between canagliflozin 100 mg and placebo groups at Week 12. Assuming a significance level of 5% and a

standard deviation (SD) of 18 points for mean change in KCCQ-TSS, a total of 1,900 participants will be randomized in this study providing approximately 95% power. This sample size is adequate to account for a potential 5% to 10% dropout rate - that is, with a dropout as high as 10% [n=1,710], the statistical power remains >90%.

After approximately 10 months, the planned enrollment in this study is not expected to reach the target of 1900 participants by the scheduled end of study enrollment window in the first quarter of 2021. Instead, the final anticipated enrollment may only be in the range of about 400-480 participants. Consequently, a reduction in total sample size of this magnitude will have impact on the statistical power for the assumed effect size as well as for various other plausible effect sizes, shown by the figures below. At 50% statistical power, an effect size of 3.5 points in the KCCQ change score (assuming standard deviation is 18) can be detected with a minimum sample size of 400. For power ≥80%, the required sample size has to be at least 445 if the expected mean difference in KCCQ-TSS change score over 12 weeks is as high as 4.8, assuming the pooled standard deviation is 18. An effect size of this magnitude for the KCCQ-TSS change score was reported in the recently concluded DEFINE-HF study[17]. Robust improvements over baseline were also reported for additional KCCQ domains in this study as well as in the SOLOIST study[17,18].

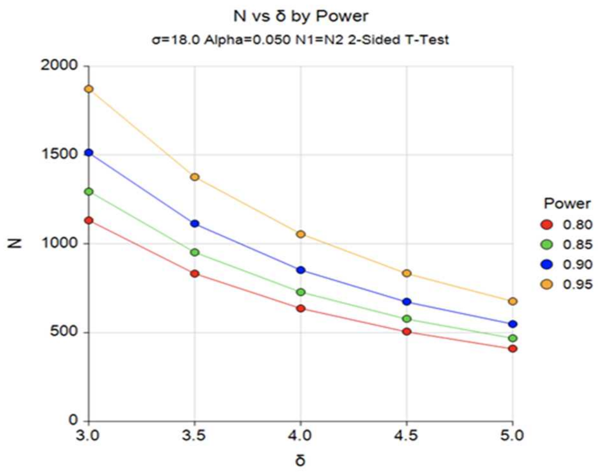

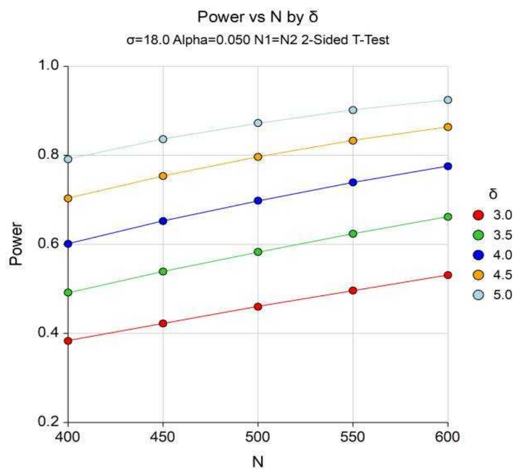

1.5. Randomization and Blinding

Central randomization will be implemented in this study. Participants will be randomly assigned to 1 of 2 study intervention groups based on a computer-generated randomization schedule prepared before the study by or under the supervision of the sponsor before initiation of the study.

Randomization will be balanced by using randomly permuted blocks and will be stratified by EF (HFrEF or HFpEF) at study entry as recorded in the participant's electronic health record (EHR).

The interactive web response system (IxR) will generate a randomization code, participant randomization number, and kit number once consent is completed and the participant is confirmed eligible by the virtual principal investigator. The kit number will dictate the study intervention assignment and the matching study intervention bottle(s) to be shipped to the participant directly from the drug distribution vendor.

The IxR will provide the kit number of the study intervention bottle(s) to be dispensed for each randomly assigned participant of the double-blind treatment period. Based on these randomization codes, study intervention will be packaged and labeled in a manner that maintains the double-blinded nature of the study.

The drug depot will not be provided with randomization codes. The codes will be maintained within the IxR, which has the functionality to allow the virtual principal investigator or designee to break the blind for an individual participant.

Under normal circumstances, the blind should not be broken until participants have completed the 12-week, double-blind treatment period. The virtual principal investigator or designee may in an emergency determine the identity of the study intervention by contacting the IxR. In the event the blind is broken, the sponsor must be informed as soon as possible. The date and reason for the unblinding must be documented in the study database. All randomization codes will be released after completion of the study. Participants who have had their intervention assignment unblinded should continue to perform their scheduled evaluations.

1.6. Study Data Flow

Data flow in this study is facilitated by an automated integrated process that extracts clinical data from 3 sources: PRA mobile health platform [PRA mHP], Care Innovations, and Symphony. These data are loaded into PRA's Common Clinical Data Store (CCDS) where the data is accessible by JReview for data validation and analysis, as well as for extraction into RAW and SDTM datasets. This automated Extract-Transform-Load (ETL) process will utilize a data container on Amazon Web Services (AWS), referred to as the "Data Analytics Container" or "DAC", which is managed by PRA. A more detailed data flow diagram is provided in Appendix 5 of this SAP.

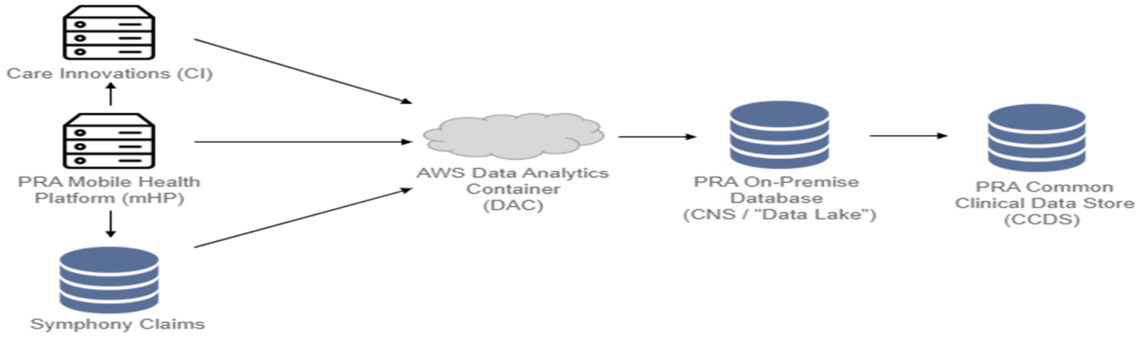

The PRA mHP platform serves as the main hub for source data capture on the CHIEF-HF study. It captures eConsent, information entered by Health Network Systems (HNS) from a participant's electronic medical records (EMRs), participant status overseen by Care Innovations (CI), randomization through integration with Endpoint IRT, drug shipment through integration with Marken, primary and secondary endpoint data from ePROs (KCCQ, PGI-S, PGIC), dosing compliance, as well as activity measured by the Fitbit watch. PRA mHP data will go into the DAC via a direct database connection. Medical and pharmacy claims data is contained within Symphony

Health Solutions and exists as completely de-identified data. This includes data used in the study to review and analyze adverse events, healthcare resource utilization (HRU) and health economics (including hospitalization/readmission due to Heart Failure [HF] or other reason, Emergency Department (ED) visits, the duration of stay at each hospitalization/readmission), as well as review of prohibited medications and new diagnoses that may require discontinuation.

Symphony claims data will be pulled into the DAC for participants randomized into the study via PRA mHP eConsent and will be mapped to SDTM as detailed in the SDTM specifications relevant to the study protocol and SAP. The collection timeframe for claims associated with AEs of special interest will begin on the Informed Consent Date through the End of Treatment + 30 days. The collection timeframe for Pharmacy claims of prohibited concomitant medications (SGLT2i) will begin on the ICF date through End of Treatment. The collection timeframe for claims associated with Health Resource Utilization will begin on the ICF Date through the 9-month observation period.

2. GENERAL ANALYSIS DEFINITIONS

As this is a virtual study, there are no planned in-person clinic visits for measuring key endpoints on the study participants. Data collection via smartphone, Fitbit device, and claims will continue throughout the 9-month duration of the study. While the focus of the first 12-week, double-blind treatment duration is to establish the primary and key secondary objectives related to primary and secondary effectiveness endpoints, the latter 6 months (i.e., Month 3 until Month 9) of the study (when no study intervention is provided) are aimed to explore multiple objectives by collecting additional follow-up data on PROs, daily activity, and HRUs and generate hypotheses of interest for future studies.

Unless stated otherwise, following the intent-to-treat (ITT) principle, analyses of the primary and secondary endpoints will be based on the full analysis set (FAS) and include data collected in the 12-week, double-blind treatment period. Where meaningful, these analyses may also be repeated using the per protocol analysis set. Analysis based on full analysis set (FAS) will also be extended to the full study data period (up to the end of the 9-month long study). In a similar way, exploratory endpoints will be analyzed according to the randomized intervention groups by using data from both the 12-week, double-blind treatment period and the 9-month study duration, respectively.

2.1. Time Windows

In this virtual study, the randomization date will refer to the day of randomization of an eligible participant. Once randomized, study intervention will be shipped and delivered to the randomized participant directly, and therefore, the first study intervention may be started by a participant a few days after the actual randomization date. This will be considered the date of starting study intervention, or, a reference day (Study Day 1). Scheduled study intervention will end after 12 weeks. After Week 12, participants will be followed up to Month 9 without receiving any further study intervention. Total study days will be numbered relative to the Study Day 1 for each participant up to the end of the 9-month long study. All measurements will be arranged in a chronological order relative to Study Day 1. Participants will be instructed and notified by the study app on their smartphone to confirm taking the study intervention on schedule and completing

all necessary patient reported outcome (PRO) measurements. Study app is programmed to allow PRO measurements to be recorded only within a pre-specified window of days around each scheduled time point.

The primary, secondary and exploratory variables (ie, KCCQ, and its individual domains, daily step count, daily floors climbed, PGIC and PGI-S, participant satisfaction survey, etc.) will be measured at specific time points as identified in the protocol, using either a smartphone-based mobile app or, the Fitbit device, as is relevant, registered by the study participant. Measurements taken closest to the planned (target) time points will be considered for the endpoint analyses and will be appropriately assigned to a time window to allow reasonable variations around the target day. Endpoint for analyses and summaries is the last recorded postbaseline data.

Daily activity measurements via the Fitbit device will be collected continuously from the time study started (ie, when both the smartphone and the Fitbit are linked by a participant). Fitbit daily activity data will be aggregated into averages over 2-week intervals starting with a baseline period (ie, Baseline = average of Study Day 1 to Study Day 14) and similarly over the entire post-baseline study duration for each participant. This is to allow capturing a robust length of physical activity for each participant using the Fitbit device and minimizing variability associated with measurements in shorter time spans. The end-of-treatment (EOT) average value of the step count will be the average of the last 14 days prior to the last dose of study intervention, regardless of when the last dose is taken.

Claims data will be extracted as access to participant-level information becomes available. This will include clinical outcomes (including hospitalizations for HF and/or other reasons) coded using International Statistical Classification of Diseases and Related Health Problems (ICD)-9 and ICD-10 as appropriate.

Time windows will not be applied to summarize the safety data collected through claims. Self-reported safety data will be summarized through Week 12 (Day 87).

Table 1 presents the time windows and the target days for each time of measurement defined in the protocol.

Table 1: Measurement Windows

Parameter	Time Interval (label on output)	Time Interval (Day)*	Target Time Point (Day)
KCCQ (and its individual domains); PGIC and PGI-S; participant satisfaction survey	Screening/Baseline	-21 to <1	1
	Week 2	11 to 17	14
	Week 4	25 to 31	28
	Week 6	39 to 45	42
	Week 12	81 to 87	84
	Month 6	161 to 175	168
	Month 9	245 to 259	252
Daily Step Count / Daily Floors Climbed **	Baseline to Week 2	1 to 14	
	Week 3 & Week 4	15 to 28	
	Week 5 & Week 6	29 to 42	
	Week 7 & Week 8	43 to 56	

Parameter	Time Interval (label on output)	Time Interval (Day)*	Target Time Point (Day)
	Week 9 & Week 10	57 to 70	
	Week 11 & Week 12	71 to 84	
	…	…	
	Week 35 & Week 36	238 to 252	

*Relative to Study Day 1

** Measurements over a 2-week interval with ≥ 100 daily steps to allow capture of physical activity

2.2. Pooling Algorithm for Analysis Centers

This is a virtual study with multiple IHNs competitively enrolling HF patients to meet the overall study enrollment target. Therefore, from the analytical perspective, no separate sites exist for pooling to be applicable. It will be considered a single center study.

2.3. Analysis Sets

An overview of the different analysis sets and their usage is presented in Appendix 4: Usage of the Analysis Sets.

2.3.1. All Randomized Analysis Set

The all randomized analysis set includes all participants who are randomly assigned to a study treatment. In order to adhere to the ITT principle, analyses based on all randomized analysis set will use the following rules:

- participants will be evaluated according to the study intervention they have been assigned to (which may be different from the study intervention they have received)
- all available data will be included (e.g., including assessment after study intervention discontinuation).

2.3.2. Full Analysis Set (FAS)

This includes all randomized participants who have received at least one dose of study intervention/medication and have at least one post-baseline KCCQ measurement.

- Randomized participants in this analysis set will be considered regardless of when they received/started their first dose of study intervention/medication.
- all available data will be included (e.g., including assessment after study intervention discontinuation).

Analyses of the primary, secondary, and exploratory endpoints in this study will be based on the full analysis set (FAS).

2.3.3. Per Protocol Analysis Set

The per protocol analysis set (PP) will consist of all participants who received study intervention and who complied with the protocol to allow assessment of the treatment effects. Specifically, this includes randomized participants who comply with the study intervention, have at least the key (ePRO) measurement of KCCQ completed, and do not experience major protocol deviations that may have an impact on estimating the treatment effect. The effectiveness analysis for the primary and secondary endpoints will be repeated for the PP analysis set.

2.3.4. Safety Analysis Set

The safety analysis set includes all randomized participants who received at least 1 dose of study intervention. Summary of available safety data from all sources (claims and patient reported) will be based on this analysis set.

2.4. Definition of Subgroups

Analysis for the primary effectiveness endpoint will be performed for the subgroups identified below.

Subgroup	Definition
Region**	US - East, West, North and South
Age Group	• 18-25<br>• 26-50<br>• 51-64<br>• >65
Gender	• Male<br>• Female
Race	• White<br>• Non-White
Diabetes type	• T2DM<br>• No-T2DM
HF – Ejection Fraction type	• pEF (preserved ejection fraction)<br>• rEF (reduced ejection fraction)

**Information on region will be based on details available through IHNs.

Additional subgroups may be identified (including those based on combinations of T2DM and HF ejection fraction types) before finalizing the database.

2.5. Study Day and Relative Day

Study Day 1 refers to the start of the first study intervention administration. All assessments recorded via the smartphone/mobile app and/or Fitbit device will be assigned a day relative to this date.

Relative day for a specific time-point of measurement is defined as:

- measurement date - (date of Study Day 1) +1, if measurement date is ≥ date of Study Day 1
- measurement date - date of Study Day 1, if measurement date < date of Study Day 1.

2.6. Baseline, End of Treatment, and End of Study

Baseline is defined as the last observation made prior to the start of the first study intervention administration. End of Treatment (EOT) observation will refer to the last observation made prior to and including the day of the last dose of study intervention, regardless of when the last dose is taken. For the PRO measurements, EOT observation will include the last observed value prior to the last dose of study intervention, and/or, within a measurement window allowed by the app that may overlap the last dose date (i.e., may extend the last dose date by a few days). For the daily

activity data, it will represent average from the last 14 days prior to the last dose taken, regardless of when the dose is taken.

The EOS value is defined as the observation that is taken on the date of very last available postbaseline data in the study period. Unscheduled measurement results are included in this definition and will be considered as the last timepoint value if the unscheduled measurement result is the last postbaseline result available within the analysis period.

2.7. Imputation Rules for Missing AE Date/Time of Onset/Resolution

Claims data are generally based on service dates and not on start and end dates for adverse events. Consequently, no imputation rule for missing AE dates can be applied.

3. INTERIM ANALYSIS AND DATA MONITORING COMMITTEE REVIEW

No interim analysis is planned in this study. However, data from the 12-week double-blind treatment period will be summarized/analyzed and reported out first as soon as the last randomized participant has completed the treatment period. Data from the full 9-month study period will be reported after the follow-up is completed on all study participants.

4. SUBJECT INFORMATION

The number of participants in each analysis set will be summarized and listed by intervention group, and overall. In addition, the distribution of participants by region within the US and recruiting networks will be presented, unless otherwise noted.

4.1. Demographics and Baseline Characteristics

Table 2 presents a list of the demographic variables that will be summarized by each intervention group, and both combined for the full analysis set (FAS). Demographics will also be summarized by various IHNs (or regions) using the FAS as well as the all randomized analysis set.

Table 2: Demographic Variables

**Continuous Variables:**	**Summary Type**
Age (years)	Descriptive statistics (N, mean, standard deviation [SD], median and range [minimum and maximum], and IQ range).
**Categorical Variables**	
Age (18-25 years, 26-50 years, 51-64 years, and ≥65 years)	Frequency distribution with the number and percentage of participants in each category.
Sex (male, female)	
Race[a] (American Indian or Alaska Native, Asian, Black or African American, Native Hawaiian, or, other Pacific Islander, White, Other)	
Diabetes status at baseline (T2DM or no T2DM)	
HF ejection fraction type (HFrEF or HFpEF)	

[a] If multiple race categories are indicated the Race is recorded as 'Multiple'.

4.2. Disposition Information

Total number of screened participants and number of screen failures will be provided. The total number of participants randomized into the study will be provided. Randomized participants who complete the 12-week, double-blind treatment period and who do not discontinue study intervention prematurely are considered study intervention completers.

The number of participants in the following disposition categories will be provided for the 12-week, double-blind treatment period by intervention group and overall:

- participants randomized
- Participants treated with study agent.
- participants receiving study intervention
- participants completing the double-blind treatment period
- participants who discontinued study intervention
- participants who terminated study prematurely
- reasons for discontinuation of study intervention and termination of study
- participants who were unblinded during the double-blind treatment period.

The distribution of the time to study termination for the 12-week, double-blind treatment period will be displayed with Kaplan-Meier curves. Participants who terminate study participation prematurely at any time will be considered an 'Event' and their date of study termination will be used in the time to study termination calculation. In this analysis, participants who complete the 12-week, double-blind treatment period will be considered 'censored' and the date of double-blind treatment period completion will serve as the time of censoring.

4.3. Treatment Compliance

Participants will be instructed and notified by the study app weekly to confirm the number of study intervention capsules that they took that week. Study intervention compliance data will be monitored bi-weekly for ensuring compliance is >80%.

Compliance with the study intervention is defined in this study in terms of the total number of doses received during the total treatment period of 12 weeks and not in terms of maintained drug concentration while participating in the study. Participants who take study intervention as instructed >80% of the total study intervention duration will thus be considered compliant.

For the double-blind study intervention period, compliance (%) = 100 x number of days taking study intervention (pills)/total study intervention duration (including days off study intervention).

The number and percentage of participants who have >80% study intervention compliance through Week 12 (end of double-blind treatment period) will be provided by randomized group.

4.4. Extent of Exposure

The number and percentage of participants who receive study intervention will be summarized by randomized group.

Descriptive statistics for study intervention duration (N, mean, SD, median, and range [minimum, maximum]) will be presented by randomized group based on the safety analysis set. Person-years of exposure to study intervention are calculated [as days of intervention/365.25] and will be presented by randomized group.

Duration of study intervention will be summarized in the following duration categories: [<1 week, 1-<2 weeks, 2-<3 weeks, …, 11-≤12 weeks] by randomized group and presented graphically in a histogram.

Cumulative study intervention exposure [≥1 week, ≥2 weeks, …, ≥12 weeks] will also be summarized.

Study intervention duration is defined as (date of last dose of study intervention – date of first dose of study intervention) +1.

4.5. Protocol Deviations

In general, a list of major protocol deviations will be produced for the final clinical study report (CSR) and these major deviations from protocol may have the potential to impact participants' rights, safety, or well-being, or the integrity and/or result of the clinical study. Participants with major protocol deviations will be identified prior to database lock and summarized

4.6. Prior and Concomitant Medications

In this pragmatic virtual study, other than history on prior medications up to 36 months, no additional information including specific start/end dates of using prior medications will be available. However, dates of prescription-fills for specific concomitant/prohibited medications collected through the claims/pharmacy database for the duration of the study may be available. The concomitant medication use during study may be categorized into broad classes of **glucose-lowering agents** (such as insulin, metformin, sulfonylurea, DPP-4 inhibitor, GLP-1 receptor agonist), **renal and cardiovascular (CV) protective agents** (such as RAAS inhibitor [including sac/val], statin, antithrombotics, beta-blockers, diuretics, IV in-patient diuretics, vasodilators, inotropes, anti-infectives) and **UGT inducers** (such as carbamazepine, phenytoin, phenobarbital, tiprnavir, rifampicin, testosterone, propionate, nelfinavir, ritonavir, efavirenz, lamotrigine) respectively.

5. EFFICACY

5.1. Analysis Specifications

Descriptive statistics for continuous variables include count (n), mean, SD, standard error (SE), median, minimum, and maximum. Descriptive statistics for categorical variables include participant counts and percentages.

5.1.1. Level of Significance

A 5% level of significance will be used for all analyses. All statistical tests and 95% confidence intervals presented in the analyses of endpoints will be 2-sided.

5.1.2. Data Handling Rules

This study will obtain mainly 3 types of data for statistical analysis: the smartphone/app-based PRO data, the activity data collected from the Fitbit device and the claims data on adverse events (including clinical outcomes, prescription fills, and related HRU). In addition, participant's self-reported AE data collected via a Call Center will be directly sent over to Janssen Safety separately and is described below as well. All data will be received and processed by PRA (3rd party vendor, see Appendix 5: CHIEF-HF Study Data Flow Diagram) into analysis files based on specifications provided by the sponsor/study SAP unless specified otherwise.

Smartphone Data - PRO Measures

PRO data (ie, KCCQ, PGIC and PGI-S, participant satisfaction survey) will be collected via the smartphone apps available with participants. Based on the administration of the KCCQ at screening/baseline through an app, an overall summary score will be produced to decide eligibility of individual participant into the study. The individual domains and summary scores of KCCQ will be calculated using the scoring algorithm provided in the KCCQ manual at specified time points during the study. Missingness in these PRO measures may result from participants not completing the questionnaires on the smartphone app at the scheduled times during the study period for various reasons including non-compliance with the protocol, discontinuation due to AE, loss to follow up, etc.

Missing and incomplete data in KCCQ PRO measure may be of 2 types: missing at the form-level and at the item-level. Form-level missing data refers to a participant missing an entire PRO assessment for a scheduled time point. If not otherwise specified, analyses will be conducted on all available data and form-level missing data will not be imputed. For the KCCQ, derivation of individual domain scores in the presence of missing item-level responses will follow algorithms specified in the KCCQ manual. PRA will calculate baseline KCCQ overall summary score based on the KCCQ app and include in the analysis database. For calculation of all other KCCQ domain scores (including the KCCQ-TSS) at baseline and at all post-randomization time points, scoring instructions available per the KCCQ manual with Janssen will be followed (see Appendix 8). For the PGIC and PGI-S measures, a single imputation (ie, last observed scores carried forward) for missing post-baseline scores will be used as a conservative rule. Vertical file formats will be prepared for all PRO data, reading one record per patient for each measurement time (ie, baseline, Weeks 2, 4, 6, and 12, and Months 6 and 9, respectively).

Fitbit Data - Clinical (Activity) Measures

Data from the Fitbit device pertaining to the daily total step counts and daily total floors climbed will be aggregated over 2-week intervals for the entire study duration (see Table 1). Due to the general sedentary nature of HF patients, Fitbit recorded <100 steps taken in a day will not be

considered in the calculation of averages for daily step counts . If missing >7 days (50%) in a 2-week interval, the corresponding 2-week interval average will be set to missing.

In case of premature discontinuation of the study intervention, the average of the last non-missing 14-day window prior to the last dose of study intervention will always be used as an EOT value. If the EOT value corresponding to the period (EOT-14, EOT-1) is missing (not valid), the EOT period will be rolled back by one day, ie, (EOT-15, EOT-2), (EOT-16, EOT-3), until a non-missing (valid) value is available. This corresponds to a last observation carried forward (LOCF) up to Week 12/EOT approach. If no post-baseline daily activity data have been collected for at least 7 days over a 14-day time window, no EOT value will be computed, and the post-baseline value will be set to missing.

Baseline for daily step count and daily floors climbed variables will be defined by averaging the daily values over a 14-day interval starting with the first dose date. These data will be organized into a vertical file format to read one record per participant for averages over each 2-week interval during the entire study (including the EOT/EOS values, as applicable).

Claims Data – AEs, HRUs, Clinical Outcomes

Claims data pertaining to AEs of interest (as defined in the protocol), number of prescription fills, HRUs, and clinical outcomes reported with appropriate ICD codes applied at the individual participant level will be organized with one record for each incident (ie, hospitalization, etc.) date. Adverse events of interest include all severe hypoglycemic events, acute kidney injury, fractures, diabetic ketoacidosis, and lower limb amputation.

In addition, the following adverse events will be examined: hypotension; Fournier's gangrene; genital mycotic infections; urinary tract infections (including urosepsis and pyelonephritis); increased urination; and hypersensitivity reactions including angioedema and anaphylaxis.

Self-reported AE data – Data from participants self-reporting adverse events (AEs) to a Call Center (set up by Care Innovations) will be manually submitted directly to the Sponsor (i.e. Janssen Safety [GMS]) for appropriate evaluation and analysis. Janssen Safety (GMS) will extract participant-reported AEs and transfer data to PRA for upload into the CCDS. Self-reported events will be captured from the date of the first dose of study intervention through the end of treatment (Month 3) plus 30 days. A listing of these self-reported AE may be produced based on available data.

5.2. Primary Efficacy Endpoint

5.2.1. Definition

The primary effectiveness endpoint is the change in the KCCQ-TSS from baseline to Week 12.

5.2.2. Estimand

The primary estimand, the main clinical quantity of interest to be estimated in the study, is defined by the following 5 attributes (ICH E9 [R1]).[4]

- Population: FAS: all randomized participants with HF (who either have HFrEF or HFpEF, regardless of T2DM status) with at least one dose of study intervention and at least 1 post-baseline KCCQ measurement
- Treatment: canagliflozin 100 mg or placebo
- Variable: change from baseline to Week 12 post first dose in the KCCQ-TSS
- Intercurrent events: treatment discontinuations (either due to AE or other reasons) or treatment switching; use treatment policy strategy to address intercurrent events (i.e., use all available measurements collected during Week 12 , regardless of the occurrence of the intercurrent event); events of "loss to follow up" or "study withdrawal" or "death" are not considered intercurrent events but rather missing data.
- Population-level summary: difference in mean change from baseline to Week 12 in the KCCQ-TSS between randomized groups.

In other words, the main quantity to estimate for the primary effectiveness endpoint is the mean difference between the 2 treatment groups (canagliflozin 100 mg and placebo) of the change from baseline to Week 12 in the KCCQ-TSS.

5.2.3. Analysis Methods

The primary effectiveness endpoint analysis will be based on the full analysis set (FAS).

Considering the repeated measurements of the KCCQ-TSS in the double-blind treatment period (ie, at baseline, Weeks 2, 4, 6, and 12 respectively), the primary effectiveness endpoint (change in the KCCQ-TSS from baseline to Week 12) will be analyzed by a MMRM method simultaneously adjusting for treatment, stratification factor (HFrEF or HFpEF), time, time by treatment, and baseline KCCQ-TSS value as covariates. Time is repeated within participants and an unstructured covariance structure will be assumed across treatment groups to model the within-participant errors. Fixed effects tests will be computed using the Kenward-Roger method to determine the degrees of freedom of the denominator. In case of convergence issues, alternative variance-covariance structures will be evaluated in the following order, with the first structure in the following list that converges being used in the analysis: heterogeneous Toeplitz, standard Toeplitz, and AR (1) with separate participant random effect. The primary comparison will be based on the difference in the LS mean changes from baseline to Week 12 between treatments. The treatment difference in the LS means, corresponding 95% confidence intervals (CI) and associated p-value will be calculated. The LS means ±SE of change from baseline values estimated by MMRM will be plotted by each timepoint for each intervention group.

```
PROC MIXED DATA=analysis_dataset;
CLASS treatment time ptid;
MODEL change = treatment time treatment*time baseline strat_var/DDFM=KR;
REPEATED time / SUBJECT=ptid TYPE=UN;
LSMEANS treatment;
LSMEANS treatment*time/pdiff cl;
RUN;
```

An MMRM model assumes the KCCQ-TSS data missing at random (MAR). A sensitivity analysis may be performed involving multiple imputation (MI) modeling approach for missing KCCQ-TSS values when the MAR assumption is considered not valid. Implementing the MI process requires 3 steps: 1) each missing value is imputed multiple times, based on statistical modeling, resulting in repetitions of the original dataset, along with the newly imputed values (implemented in the Statistical Analysis System [SAS] using PROC MI); 2) each dataset is analyzed using an MMRM model (implemented in SAS using PROC MIXED). The LS means and SEs from each analysis will be output to a SAS dataset; 3) results from the analyses performed on multiple datasets in step #2 are combined to provide valid statistical inference (implemented in SAS using PROC MIANALYZE).[1]

A complete SAS code for MMRM model and implementation of the MI approach described above will be added to the final SAP appendix or to the final study report. Mortality in this short-duration trial is expected to be low and to be comparable between groups and will be ignored in the primary analyses of mean differences in changes in KCCQ total symptom scores, but can be included in the responder analyses (described below) by assigning them to the lowest response (greatest deterioration) category. Should there be a >5% mortality rate in this study, and if it is unequally distributed between treatment arms, then joint modeling of survival and health status (e, g.,KCCQ) will be performed. A win-ratio approach[19,20] may also be considered by analyzing the KCCQ-TSS score as a composite, rank-based outcome, incorporating participant vital status at 12 weeks along with a change in score from baseline to 12 weeks in surviving participants. This essentially is the rank analysis of covariance method, with a corresponding win ratio used to estimate the magnitude of treatment effect.

An exploratory responder analysis will be performed when comparing proportions of participants with a 5- (or 10-) point improvement in the KCCQ-TSS from baseline to Week 12 between the groups. This will be based on a 2-sample difference in proportions test along with a 95% confidence interval for the difference in 2 proportions.

The mean differences between the groups will be augmented with both responder analyses and empirical cumulative distribution and probability density function curves. Specifically, the empirical cumulative distribution function (CDF) of the change from baseline in KCCQ-TSS will be generated. CDF shows the proportion of the population scoring less than or equal to each possible change score. CDFs are useful as they graphically characterize the treatment effect or differences between groups. It also enables a ready assessment of different magnitudes of clinical change (e.g., 5, 10, and ≥20-point changes, corresponding with small to moderate, moderate to large, and large to very large clinical changes). CDFs and histograms will be shown for changes from baseline to Week 12 in the KCCQ TSS. For all CDF plots, the x-axis is the score change from baseline to final time point (i.e., Week 12) for the KCCQ-TSS. The y-axis is the cumulative proportion of the participants in each of the corresponding categories that reach the score change on the x-axis.

According to the Food and Drug Administration's (FDA's) guidance on PRO measures, a responder is defined by the empirically determined score change in a measure, experienced by an individual patient over a predetermined time period that has been demonstrated in the target

population to have a significant treatment benefit. This is also often referred to as responsiveness or ability of the PRO measure to capture the true underlying change in the patient health status over time. Assessing the responsiveness of the PRO measure using an anchor-based approach involves comparing the changes in the PRO scores to other clinically meaningful markers or anchors that represent a valid measure of clinical change.

For the anchor-based analysis of the KCCQ-TSS endpoint, participants will be characterized based on the PGIC scores at Week 12. The mean change in KCCQ-TSS endpoint observed in the smallest improvement category of the PGIC will be examined as a key anchor-based indicator of a responder. The definition for a small improvement in PGIC is given as those who improve by one category (i.e., from “a little improved” to “somewhat improved”). Specifically, the retrospective PGIC responses of “3=a little improved” will be selected to represent time points where participants register meaningful changes in their HF symptoms. Each time a participant responds with a PGIC score of “3=a little improved”, a KCCQ-TSS change score from the preceding visit is computed. For the double-blind treatment period, the PGIC will be collected at 4 time points (i.e., Weeks 2, 4, 6, and 12 respectively) which can yield between zero and 4 KCCQ-TSS change scores by each participant. For each participant with at least one PGIC response of “3=a little improved”, a mean of the individual participant’s KCCQ-TSS change scores is computed. A summary of KCCQ-TSS change scores across PGIC categories[16] will then be presented.

The KCCQ-TSS change scores may also be standardized into effect size (ES) statistics by dividing them by their SD at baseline. The following cutoff values will be used to interpret effect size: small ES = 0.2, moderate ES =0.5, and large ES = 0.8. Together with the CDF, results from the anchor-based methods will be examined and a single responder definition threshold will be determined, if the results from the anchor-based assessments in this study differ substantially from prior studies examining the clinical meaningfulness of changes in the KCCQ.

In addition, a distribution-based change (i.e., minimum detectable [MID] change) may be explored for which the KCCQ-TSS can be derived using the baseline SD. Generally, a value of half the baseline SD has been frequently found to correspond to the MID obtained via the anchor-based method. A value of 0.2*SD may also be examined.

Subgroup analyses for the change from baseline to Week 12 for the primary effectiveness endpoint will be conducted for subgroups (listed in Section 2.4) based on all randomized analysis set. These analyses will assess consistency of the overall treatment effect across subgroup variables.

The main MMRM analyses will be repeated with each subgroup tested separately. Intervention group differences of LS means as well as corresponding 95% CIs within each subgroup level will be presented.

The intervention-by-subgroup interaction p-value will be estimated using a separate MMRM model including intervention, baseline KCCQ value, stratification factor (HFrEF/HFpEF), subgroup, and intervention-by-subgroup interaction term(s). Interactions with p-value <0.1 will be investigated further to determine the nature of interaction (quantitative or qualitative) and the

association with other subgroups. For qualitative interactions, Gail and Simon (1985) approach[15] may be considered.

Results will be presented in a summary table and in a forest plot. The forest plot will display the "overall" treatment effect, based on the main MMRM analysis, as a reference line. LS means of the intervention group differences, the corresponding 95% CIs, and the numbers of participants in the subgroup levels will be presented in the forest plot.

5.3. Secondary Endpoints

5.3.1. Definition

The first key secondary effectiveness endpoint is change in the daily step count from baseline to Week 12 averaged over two-week intervals. Even though evidence of a meaningful change in daily step count in chronic HF patients via Fitbit device is not readily available, there are several studies that have conducted six-minute walk tests (6MWT) among HF patients in general and examined a minimum clinically important difference in daily step counts under different conditions. A change of 30 meters[10] (approximately 1000 steps) may be considered in the present study to be of clinical interest to benchmark the expected mean change in daily step count in this study over the double-blind treatment period. This expected mean change in daily step count will also be examined in relation to variables such as PRO measures (as anchors) and clinical outcomes to better characterize treatment effect on daily activity.

As HF patients are generally not consistent in their daily walk patterns, there will be variations in levels of their daily activity (whether it is a weekday or a weekend) as well as variability in the total wear time of the Fitbit device. As a rule, we will consider Fitbit recorded ≥100 steps[13] taken in a day in the calculations of averages. This will ensure capturing any meaningful level of physical activity while avoiding even smaller variations in daily steps taken by a participant wearing a Fitbit device. For each participant, baseline daily step count will be calculated by averaging daily step counts from Study Day 1 through Study Day 14. All subsequent post-baseline daily step count data will also be averaged in 2-week intervals. The change from baseline to Week 12 in daily step count will be based on subtracting the average of daily step count for Week 11 and Week 12 from the baseline average for each participant. Additionally, daily step count data will be averaged in 3-, 5- and 7-day (1-week) intervals at baseline and throughout the treatment period respectively and corresponding changes from baseline will be calculated.

The variable daily floors climbed will also be aggregated in a similar fashion.

5.3.2. Estimand

The first key secondary estimand to be estimated in the study, is defined by the following -

- Population: FAS (i.e., all randomized participants with HF who either have HFrEF or HFpEF, regardless of T2DM status with at least one dose of study intervention and at least 1 post-baseline KCCQ measurement)
- Treatment: canagliflozin 100 mg or placebo

- Variable: change from baseline to Week 12 in the daily step count averaged over 2-week intervals
- Intercurrent event: treatment discontinuations (either due to AE or other reasons) or treatment switching or temporary disruption in daily activity; use treatment policy strategy to address intercurrent events (i.e., use all available measurements collected during study, regardless of the occurrence of the intercurrent event); events of "loss to follow up" or "study withdrawal" or "death" are not considered intercurrent events but rather missing data.
- Population-level summary: difference in mean change from baseline to Week 12 in the daily step count averaged over 2-week intervals (i.e., difference is based on subtracting the average of daily step count for Week 11 and Week 12 from the baseline 14-day average daily step count) between two randomized groups.

5.3.3. Analysis Methods

Daily step counts and daily floors climbed data at baseline will be presented as 14-day averages of daily counts starting with the first dose date and ending with Day 14 during the double-blind treatment period. This method of averaging will continue until end of Month 9 (Day 252) or, until the time of end of study (EOS) observation is available.

The above two key secondary endpoints will be analyzed in two ways –

1) following the same approach as employed for the primary effectiveness endpoint (i.e. using an MMRM model adjusting for baseline covariates such as treatment, stratification factor and baseline average step count).

2) by calculating mean change from baseline to Week 12 in daily step counts using the corresponding 14-day averages at baseline and Week 12 respectively. That is, the mean difference in the 14-day averages in daily step counts or daily floors climbed is calculated for each randomized intervention group and difference in mean changes between the 2 randomized intervention groups will be compared using a 2-sided t-test.

As stated in Section 1.3, the first key secondary endpoint of daily step count will be tested once the primary endpoint is shown to be statistically significant at 5% significance level.

Other secondary endpoints refer to changes in the KCCQ individual domain scores (physical limitation, quality of life), and the KCCQ clinical summary and overall summary scores from baseline to Week 12. The individual KCCQ domain scores as well as the summary scores will be produced at each time point (based on the KCCQ algorithm) with changes from baseline calculated at each specified post-baseline time point. For these endpoints, an MMRM model (like that specified for the analysis of the primary endpoint) will be employed to summarize changes over time from baseline to Week 12. Hypotheses related to these domains will be independently tested at 5% without any control for overall type I error rate.

5.4. Exploratory Variables

5.4.1. Definition

The exploratory endpoints are the following:

- mean PGIC scores over time
- changes in PGI-S scores from baseline to Week 12
-
- change in HRU and health economics data
- number of outpatient intravenous therapies, ED visits, and hospitalizations
- time to first hospitalization/readmission for heart failure
- time to first hospitalization/readmission for other events
- time to death

5.4.2. Analysis Methods

The exploratory endpoints listed below will be presented using descriptive statistics at each time point measurements are taken. Where specified, 95% confidence intervals (CIs) and p-values will be presented but no multiplicity adjustment will be made. Analyses of mean changes from baseline to Week 12 and month 9 (Day 252) respectively will be conducted, as described below.

- Appendix 6: Patient Global Impression of Change (PGIC) is a validated generic tool for assessment of overall change in the severity of illness following treatment. Participants rate how they feel now compared with how they felt before receiving study intervention on a 7-point scale where 1 is "Very much improved" and 7 is "Very much worse". A mean ± SD PGIC score for each time point will be calculated by randomized intervention group. Distribution of PGIC score at Week 12 will also be presented. Based on the PGIC symmetric scale, a dichotomous scale of "Yes" or "No" will be derived. A favorable change is score of 1-3 = 'Yes', which means there is significant improvement with the study intervention. If the response is 4-7 = 'No', it is considered no significant change. Change in the KCCQ-TSS from baseline to Week 12 by PGIC response at Week 12 will be summarized by treatment group, and, also graphically displayed (i.e., x-axis will display categories of PGIC response at Week 12 and y-axis will display mean change scores from baseline to Week 12 in KCCQ-TSS by treatment group).
- Distribution of participants in PGI-S (see Appendix 7: Patient Global Impression of Severity (PGI-S)) response categories will be summarized by randomized intervention group at baseline and by Week 12. Mean change in PGI-S score between baseline and Week 12 will be summarized by randomized group. Change in the KCCQ-TSS from baseline to Week 12 by PGI-S response at Week 12 will be summarized.
- For daily activity measured by daily step counts, total active days (defined as Fitbit recorded ≥100 steps) and percent of active days to total days spent in the study analysis period will be summarized for each intervention group using mean and SD. Level of daily activity will also be described using categories of total step counts per day – 1) <3000 steps per day (low activity/sedentary), 2) 3000-5999 (moderate activity), and 3) ≥ 6000 steps per day (high activity). Associations between daily step count (categorized into activity levels) and baseline characteristics (eg, age, sex, race, T2DM status, EF type, etc.) will be examined using a 1-way ANOVA for continuous data values and the Fisher exact test for categorical data.

- Correlation between total step counts per day and clinical events (eg, # of outpatient intravenous therapies, # ED visits, # hospitalizations, etc.) will be described for the double-blind treatment period and total study duration separately. Relationships between change in step counts/floors climbed and incidence of reported clinical outcomes and PRO measures will be examined at the end of the double-blind treatment period and end of study separately.
- Daily floors climbed will be summarized by each intervention group for the double-blind treatment period and over the entire study duration separately.
- Descriptive statistics (ie, N, mean, SD, minimum and maximum) will be presented for total HRU and health economics data for each intervention group separately for the double-blind treatment period and the observational (no intervention) period. HRUs and health economics data typically include information regarding participants using health services for managing any clinical events/illnesses, hospitalizations/ED visits, surgeries, medication issues/prescription changes, and costs incurred.
- Descriptive statistics will be presented for participant's satisfaction with his/her experience with the virtual design at the end of the study.
- Using data from claims, the number of outpatient intravenous therapies, ED visits, and hospitalizations by each intervention group will be summarized. These summaries will include descriptive statistics.
- Using data from the claims and data on deaths, time to first hospitalization/readmission for heart failure, time to first hospitalization/readmission for other events, and time to death will each be analyzed using a Cox's regression model both for the double-blind treatment period and the entire study duration and include terms for study intervention and stratification factor (HFrEF/HFpEF). Hazard ratios, corresponding 95% CIs, and associated p-values will be presented. Distribution of the times to first hospitalization/readmission for HF, time to first hospitalization/readmission for other events, and time to death will be displayed with Kaplan-Meier curves. Participants who do not experience any such events of interest during the study analysis period will be considered 'censored' in these analyses.

No adjustment for multiplicity will be performed for the exploratory analyses listed above.

6. SAFETY

Summaries of safety data will be based on the safety analysis set and will use claims data.

6.1. Adverse Events

All AE data summaries will be based on the safety analysis set except where stated otherwise. A treatment emergent AE is defined as an event that occurs after the first dose of study intervention (Study Day 1) and/or worsens in severity during the double-blind treatment period.

Adverse events self-reported to the Call Center will be assessed by Janssen Global Medical Safety (GMS) for start and end dates, causality and meeting SUSAR reporting requirements. If the specific dates pertaining to the self-reported AEs are unknown, there will be limited follow-up for resolution. For any events still ongoing at the end of the study, there will be no follow up (as the Call Center will be closed at that point) possible. Adverse events will be coded according to MedDRA and will be reported by preferred term for each randomized intervention group. Janssen Data Management will request a listing of all AEs/SAEs reported to the Call Center from the

Janssen GMS. This listing will be shared with PRA (external vendor) to convert the information into the SDTM format and for inclusion in the final SDTM data package for summary by various subgroups (e.g., T2DM and non-T2DM).

Adverse events collected through the claims database will be analyzed separately for the 12-week treatment period + 30 days after last dose and the 9-month entire study period respectively. It is assumed that claims database will have dates of service provided (procedure dates, office visits, hospital admissions, etc.) but not actual start/end dates of events prior to claims being filed. Therefore, no imputation of AE start/end dates will be feasible based on the claims data. Dates of service(s) rendered to participants during the study (i.e., starting from the time informed consent is signed until the end of double-blind treatment plus 30 days) will be taken into account. The claims data will utilize ICD codes to identify the SAEs and AEs of interest which are defined in the protocol and for the final analysis. The AEs of interest include all severe hypoglycemic events, acute kidney injury, fractures, diabetic ketoacidosis, and lower limb amputation. In addition, hypotension, Fournier's gangrene, genital mycotic infections, urinary tract infections (including urosepsis and pyelonephritis), increased urination and hypersensitivity reactions (including angioedema and anaphylaxis) will be considered for coding and final analysis.

For the prohibited medications, analysis period will include data from the date of informed consent until the end of double-blind treatment period. For all other concomitant mediations, analysis period will include data from the date of informed consent until the end of the study. For the HRU data analysis, information collected from date of informed consent until the end of study will be considered in the final analysis.

For both the double-blind treatment period + 30 days after last dose and the 9-month entire study period, descriptive summaries (numbers and percentages) of participants having an SAE/AE of interest will be produced, including by participant's diabetes status (T2DM and non-T2DM) and by randomized intervention group. Discontinuations (and reported reasons) will be summarized by randomized intervention group based on available claims data.

There will be no reconciliation/analysis of SAE/AE data that are self-reported to the Call Center with the data obtained from the claims database.

A listing will be presented for treatment emergent hospitalizations and deaths for each randomized intervention group. AEs occurring between informed consent and the day prior to the first dose of study intervention will also be listed. This listing will be based on participants with informed consent into the study. Depending on the claims/pharmacy data, AEs occurring in participants who used prohibited (commercially available SGLT2is other than the study intervention, canagliflozin) will also be listed.

Based on pharmacy profiles of the participants after-consent in this study, the total number of participants concomitantly receiving UGT inducers during the double-blind treatment period will be summarized combined across both study intervention groups. If the number of such participants is >2% of the total 1,900 targeted for enrollment in this study, the users and non-users of UGT

inducers will be summarized by each study intervention group as well and examined further (as necessary) for any relationship to key study outcomes (eg, PRO endpoints and clinical outcomes reported in claims).

6.2. Clinical Laboratory Tests

Not Applicable.

6.3. Vital Signs and Physical Examination Findings

Not Applicable.

6.4. Electrocardiogram

Not Applicable.

6.5. Other Safety Parameters

Not Applicable.

7. PHARMACOKINETICS/PHARMACODYNAMICS

Not Applicable.

8. BIOMARKERS

Not Applicable.

9. HEALTH ECONOMICS

As treatment of participants with symptomatic HF with JNJ-28431754 (canagliflozin)JNJ-28431754 (canagliflozin) versus placebo may result in lower utilization of hospitalization/readmissions due to HF or other reason, ED visits, the duration of stay at each hospitalization/readmission, and discharge destination will be summarized by each intervention group. Comparison will be made between groups by analyzing differences in hospitalizations/readmissions and ED visits using the Fisher exact test. Descriptive statistics (eg, mean, median, minimum, and maximum) will be provided for the total length of stay in hospitals for each intervention group. Cumulative distribution function of the time to first occurrence of hospitalization/readmission will be estimated by the Kaplan-Meier method and tested using a log-rank test, stratified by the type of HF ejection fraction (ie, HFrEF or HFpEF). In addition, a Cox proportional hazards mode with intervention and stratification factor as covariates will be fitted. A point-estimate along with 2-sided 95% CI for HR of the intervention effect (in terms of relative risk reduction (RRR) = 100 x [1-HR] %) will be provided. The cumulative event rate derived from Kaplan-Meier estimate will also be displayed graphically to evaluate the timing of event occurrence and the consistency of the treatment effect over time.

REFERENCES

1. Berglund P and Heeringa S. Multiple Imputation of Missing Data Using SAS®. 2014. Cary, NC: SAS Institute Inc. CDISC. “Analysis Data.
2. Dreyer RP, Jones PG, Kutty S, Spertus JA. Quantifying clinical change: discrepancies between patients' and providers' perspectives. Qual Life Res. 2016 Sep;25(9): 2213-2220.
3. Green, C., Porter C., Bresnahan D., and Spertus, J. 2000. Development and evaluation of the Kansas City Cardiomyopathy Questionnaire: a new health status measure for heart failure. Journal of American College of Cardiology. 2000 Apr; 35(5):1245-1255.
4. International Council For Harmonisation (ICH) of Technical Requirement for Pharmaceuticals for Human Use (final version, 2019): ICH Harmonised Guideline – Addendum on estimands and sensitivity analysis in clinical trials (to the guideline on Statistical Principles for Clinical Trials) - E9 (R1).
5. Khariton Y. et al. Association Between Sacubitril/Valsartan Initiation and Health Status Outcomes in Heart Failure with Reduced Ejection Fraction: Findings from the CHAMP-HF Registry. Data presented at the European Society of Cardiology Heart Failure (ESC-HF); 2018 May 26-29; Vienna, Austria.
6. Luo N, et al. Relationship between changing patient-reported outcomes and subsequent clinical events in patients with chronic heart failure: insights from HF-ACTION, European Journal of Heart Failure (2019) 21, 63-70.
7. McMurray JJV, Solomon SD, Inzucchi SE, et al; DAPA-HF Trial Committees and Investigators. Dapagliflozin in patients with heart failure and reduced ejection fraction. N Engl J Med. 2019 Sep 19.
8. Nassif ME, Windsor SL, Tang F, et al. Dapagliflozin Effects on Biomarkers, Symptoms, and Functional Status in Patients With Heart Failure With Reduced Ejection Fraction: The DEFINE-HF Trial. Circulation. 2019 Oct 29;140(18):1463-1476.
9. Pokharel Y, et al. Association of serial Kansas City Cardiomyopathy Questionnaire assessments with death and hospitalization in patients with heart failure with preserved and reduced ejection fraction. A secondary analysis of 2 randomized clinical trials. JAMA Cardiology. 2017 Dec 1; 2912):1315-1321.
10. Shoemaker M, Curtis AB, Vangsnes E, Dickinson, MG. Clinically Meaningful Change Estimates for the Six-Minute Walk Test and Daily Activity in Individuals With Chronic Heart Failure. Cardiopulm Phys Ther J, Sep 2013;24(3), 21-29.
11. Spertus, J, Jones P. Development and validation of a short version of the Kansas City Cardiomyopathy Questionnaire. Circulation: Cardiovascular Quality and Outcomes, 2015; 8:469-476.
12. Spertus J, Peterson E, Conard MW, et al. Monitoring clinical changes in patients with heart failure: a comparison of methods. Am Heart J 2005;150(4):707-715.
13. Thorup C, Hansen J, Gronkjaer M, et al. Cardiac Patients’ Walking Activity Determined by a Step Counter in Cardiac Telerehabilitation: Data From the Intervention Arm of A Randomized Controlled Trial. J Med Internet Res 2016 Apr; 18(4); e69.
14. Vetrovsky T, Siranec M, Parenica J, et al. Effect of a 6-month pedometer-based walking intervention on functional capacity in patients with chronic heart failure with reduced (HFrEF) and with preserved (HFpEF) ejection fraction: study protocol for two multicenter randomized controlled trials, J Transl Med, 2017; 15(1):153.
15. Gail, M. H., and Simon, R. (1985). “Tests for Qualitative Interactions between Treatment Effects and Patient Subsets.” *Biometrics* 41:361–372.
16. Mehta, L. et al. (2015). Identifying an important change estimate for the multiple sclerosis walking scale-12 (MSWS-12v1) for interpreting clinical trial results, Multiple Sclerosis Journal – Experimental, Translational, Clinical” 1:1-9

17. Nassif, M. et al. (2019). Dapagliflozin Effects on Biomarkers, Symptoms, and Functional Status in Patients With Heart Failure With Reduced Ejection Fraction - The DEFINE-HF Trial. *Circulation*, Vol. 140, No. 18, 1463-1476

18. Bhatt, D. et al. (2020). Sotagliflozin in Patients with Diabetes and Recent Worsening Heart Failure. *N Engl J Med*, 2020; Nov 16

19. Gasparyan, S. et al. (2019). Adjusted Win Ratio with Stratification: Calculation Methods and Interpretation, *Stat.ME*

20. Wang, D. and Pocock, S. (2016). A win-ratio approach to comparing continuous non-normal outcomes in clinical trials, *Pharmaceutical Statistics*, online 11 March 2016

APPENDICES

Appendix 1: The Kansas City Cardiomyopathy Questionnaire (KCCQ)

The KCCQ (Appendix 1: The Kansas City Cardiomyopathy Questionnaire (KCCQ)) is a well-established PRO for use in patients with HF, with published validity and reliability and was recently endorsed by the Center for Devices and Radiological Health (CDRH, US FDA) as a Certified Outcome Assessment through its Medical Device Development Tools Program and is currently under review at the Center for Drug Evaluation and Research (CDER, US FDA).

The Kansas City Cardiomyopathy Questionnaire (KCCQ) is a 23-item self-administered questionnaire developed to independently measure the patient's perception of their health status, which includes heart failure symptoms, impact on physical and social function, and how their heart failure impacts their quality of life (QOL) within a 2-week recall period. This questionnaire requires, on average, 4 to 6 minutes to complete, with a resulting score of 0 to 100, and higher scores indicating better health.

The intent of the study is to enroll participants with NYHA class II and III. As it would be difficult to verify NYHA class with claims and EHR data, the sponsor will use a KCCQ overall summary score at baseline of >40 and <80, which is highly correlated to class II and III in HFrEF and HFpEF based on the research published by Joseph, et al.[6] In addition to the KCCQ overall summary score, a baseline KCCQ TSS score will also be calculated for each participant.

The 23-item KCCQ quantifies 7 domains of patients' HF-related health status:

- Physical Limitation (6 items)
- Symptom Stability (1 item)
- Symptom Frequency (4 items)
- Symptom Burden (3 items)
- Self-Efficacy (2 items)
- Quality of Life (3 items)
- Social Limitations (4 items)

Item responses are coded sequentially (1, 2, 3, etc.) from worst to best status. Scores are generated for each domain and scaled from 0 to 100, with 0 denoting the worst and 100 the best possible status. In addition, three summary scores are calculated: **Total Symptom score** (average of Symptom Frequency and Symptom Burden), **Clinical Summary score** (average of Physical Limitation and Total Symptoms), and **Overall Summary score** (average of Physical Limitation, Total Symptoms, Quality of Life, and Social Limitation).

Among the 7 domains and 3 summary scores of KCCQ described above, this study will focus on analyzing only two individual domains (physical limitation and quality of life) and three summary scores (clinical summary, overall summary, and total symptom).

Below is a representative example of the scale questions that will be used in this study.

The KC Cardiomyopathy Questionnaire

The following questions refer to your **heart failure** and how it may affect your life. Please read and complete the following questions. There are no right or wrong answers. Please mark the answer that best applies to you.

1. **Heart failure** affects different people in different ways. Some feel shortness of breath while others feel fatigue. Please indicate how much you are limited by **heart failure** (shortness of breath or fatigue) in your ability to do the following activities over the past 2 weeks.

Place an **X** in one box on each line

Activity	Extremely Limited	Quite a bit Limited	Moderately Limited	Slightly Limited	Not at all Limited	Limited for other reasons or did not do the activity
Dressing yourself	❑	❑	❑	❑	❑	❑
Showering/Bathing	❑	❑	❑	❑	❑	❑
Walking 1 block on level ground	❑	❑	❑	❑	❑	❑
Doing yardwork, housework or carrying groceries	❑	❑	❑	❑	❑	❑
Climbing a flight of stairs without stopping	❑	❑	❑	❑	❑	❑
Hurrying or jogging (as if to catch a bus)	❑	❑	❑	❑	❑	❑

2. Compared with 2 weeks ago, have your symptoms of **heart failure** (shortness of breath, fatigue, or ankle swelling) changed?

My symptoms of **heart failure** have become…

Much worse	Slightly worse	Not changed	Slightly better	Much better	I've had no symptoms over the last 2 weeks
❑	❑	❑	❑	❑	❑

Copyright ©1992 –2005 John Spertus, MD, MPH Original US English

3. Over the past 2 weeks, how many times did you have **swelling** in your feet, ankles or legs when you woke up in the morning?

Every morning	3 or more times a week, but not every day	1-2 times a week	Less than once a week	Never over the past 2 weeks
❑	❑	❑	❑	❑

4. Over the past 2 weeks, how much has **swelling** in your feet, ankles or legs bothered you?

It has been ...

**Extremely** bothersome	**Quite a bit** bothersome	**Moderately** bothersome	**Slightly** bothersome	**Not at all** bothersome	I've had **no swelling**
❑	❑	❑	❑	❑	❑

5. Over the past 2 weeks, on average, how many times has **fatigue** limited your ability to do what you want?

All of the time	Several times per day	At least once a day	3 or more times per week but not every day	1-2 times per week	Less than once a week	Never over the past 2 weeks
❑	❑	❑	❑	❑	❑	❑

6. Over the past 2 weeks, how much has your **fatigue** bothered you?

It has been ...

**Extremely** bothersome	**Quite a bit** bothersome	**Moderately** bothersome	**Slightly** bothersome	**Not at all** bothersome	I've had **no fatigue**
❑	❑	❑	❑	❑	❑

7. Over the past 2 weeks, on average, how many times has **shortness of breath** limited your ability to do what you wanted?

All of the time	Several times per day	At least once a day	3 or more times per week but not every day	1-2 times per week	Less than once a week	Never over the past 2 weeks
❑	❑	❑	❑	❑	❑	❑

Copyright ©1992 –2005 John Spertus, MD, MPH Original US English

8. Over the past 2 weeks, how much has your **shortness of breath** bothered you?

It has been ...

**Extremely** bothersome	**Quite a bit** bothersome	**Moderately** bothersome	**Slightly** bothersome	**Not at all** bothersome	I've had **no shortness of breath**
❑	❑	❑	❑	❑	❑

9. Over the past 2 weeks, on average, how many times have you been forced to sleep sitting up in a chair or with at least 3 pillows to prop you up because of **shortness of breath**?

Every night	3 or more times a week, but not every day	1-2 times a week	Less than once a week	Never over the past 2 weeks
❑	❑	❑	❑	❑

10. **Heart failure** symptoms can worsen for a number of reasons. How sure are you that you know what to do, or whom to call, if your **heart failure** gets worse?

**Not at all** sure	**Not very** sure	**Somewhat** sure	**Mostly** sure	**Completely** sure
❑	❑	❑	❑	❑

11. How well do you understand what things you are able to do to keep your **heart failure** symptoms from getting worse? (for example, weighing yourself, eating a low salt diet etc.)

Do not understand at all	Do not understand very well	Somewhat understand	Mostly understand	Completely understand
❑	❑	❑	❑	❑

12. Over the past 2 weeks, how much has your **heart failure** limited your enjoyment of life?

It has **extremely** limited my enjoyment of life	It has limited my enjoyment of life **quite a bit**	It has **moderately** limited my enjoyment of life	It has **slightly** limited my enjoyment of life	It has **not limited** my enjoyment of life at all
❑	❑	❑	❑	❑

13. If you had to spend the rest of your life with your **heart failure** the way it is right now, how would you feel about this?

Not at all satisfied	Mostly dissatisfied	Somewhat satisfied	Mostly satisfied	Completely satisfied
❑	❑	❑	❑	❑

Copyright ©1992 –2005 John Spertus, MD, MPH
Original US English

14. Over the past 2 weeks, how often have you felt discouraged or down in the dumps because of your **heart failure**?

I felt that way **all of the time**	I felt that way **most of the time**	I **occasionally** felt that way	I **rarely** felt that way	I **never** felt that way
❑	❑	❑	❑	❑

15. How much does your **heart failure** affect your lifestyle? Please indicate how your **heart failure** may have limited your participation in the following activities over the past 2 weeks.

Please place an **X** in one box on each line

**Activity**	**Severely** limited	Limited **quite a bit**	**Moderately** limited	**Slightly** limited	**Did not** limit at all	Does not apply or did not do for other reasons
Hobbies, recreational activities	❑	❑	❑	❑	❑	❑
Working or doing household chores	❑	❑	❑	❑	❑	❑
Visiting family or friends out of your home	❑	❑	❑	❑	❑	❑
Intimate relationships with loved ones	❑	❑	❑	❑	❑	❑

Copyright ©1992 –2005 John Spertus, MD, MPH Original US English

Appendix 2: Participant Satisfaction Survey

Appendix 2 provides a representative example of the questions that will be provided to the participant.

For each of the statements below, please indicate your level of agreement:	Strongly Disagree	Disagree	Neutral	Agree	Strongly Agree
Trying something new to possibly treat my heart failure was important to me.					
The virtual study was easy for me to sign up for and get started.					
The Fitbit was easy to set up and use.					
The study app was easy to set up on my smartphone.					
The questionnaires were easy to complete on my smartphone.					
The reminders to complete the questionnaires were helpful.					
The study Fitbit provided helpful information to me.					
It was easy to reach the study team when I had questions or concerns.					
I would recommend participating in a study like this to my friends and family.					

Appendix 3: Schedule of Activities

Period	Screening			Double-Blind Treatment				No Treatment		Notes
	Screening	Baseline								
Day	Within 21 days before Day 1	Within 7 days before Day 1	1 (first dose)	14±3	28±3	42±3	84±3 End of Treatment	85-251-±7	252-±7 End of Study	Day -21 to 0=Screening; Day 1 = first dose, Day 84= end of treatment; Day 252=End of Study
Week				2	4	6	12	13-36		Week 12 is equivalent to Day 84 of treatment
Month			0				3	6	9	Month 3 is equivalent to Day 84 of treatment
**Study Procedure**										
Screening/ Administrative										
Electronic Informed consent[a]	X									
Review medical history	X									
Inclusion/ exclusion criteria[b]	X		X							All inclusion/exclusion verification should be completed within the 21-day screening period and before randomization
KCCQ baseline overall summary score <80			X							
Study Intervention Administration										
Randomization			X							
Dispense/administer study intervention			←				→			
Participant-reported adherence via app - weekly			←				→			
Assessments										
KCCQ				X	X	X	X	X[c]	X	Kansas City Cardiomyopathy Questionnaire
PGIC				X	X	X	X	X[c]	X	Patient Global Impression of Change
PGI-S			X	X	X	X	X	X[c]	X	Patient Global Impression of Severity
Step count and floors climbed			←						→	
Claims data collection[d]			←						→	

Period	Screening			Double-Blind Treatment				No Treatment		Notes
	Screening	Baseline								
Day	Within 21 days before Day 1	Within 7 days before Day 1	1 (first dose)	14±3	28±3	42±3	84±3 End of Treatment	85-251-±7	252-±7 End of Study	Day -21 to 0=Screening; Day 1 = first dose, Day 84= end of treatment; Day 252=End of Study
Week				2	4	6	12	13-36		Week 12 is equivalent to Day 84 of treatment
Month			0				3	6	9	Month 3 is equivalent to Day 84 of treatment
Participant Satisfaction Survey (optional)									X	
Health Resource Utilization and Health Economics										
HHF for any reason and duration			←————————————————————→							Hospitalizations for heart failure
Emergency department visits			←————————————————————→							
Readmissions and duration			←————————————————————→							
Ongoing Review of Participant Data										
Prohibited medications and new diagnoses			←————————————————————→							
Reporting of adverse events[e]	←————————————————————→									

Appendix 4: Usage of the Analysis Sets

Appendix 4 presents an overview of the different analysis sets and their usage.

Analyses/data displays	All randomized analysis set	Full analysis set (FAS)	Safety analysis set	Per protocol set
Patient disposition	✓	✓	✓	✓
Demographics	✓	✓	✓	✓
Baseline characteristics		✓		✓
Medical history		✓		
Treatment exposure			✓	✓
Effectiveness: Primary endpoint		✓		✓
Effectiveness: Secondary endpoints		✓		✓
Effectiveness; Exploratory endpoints		✓		
Safety endpoints			✓	
All other patient listings	✓			

Appendix 5: CHIEF-HF Study Data Flow Diagram

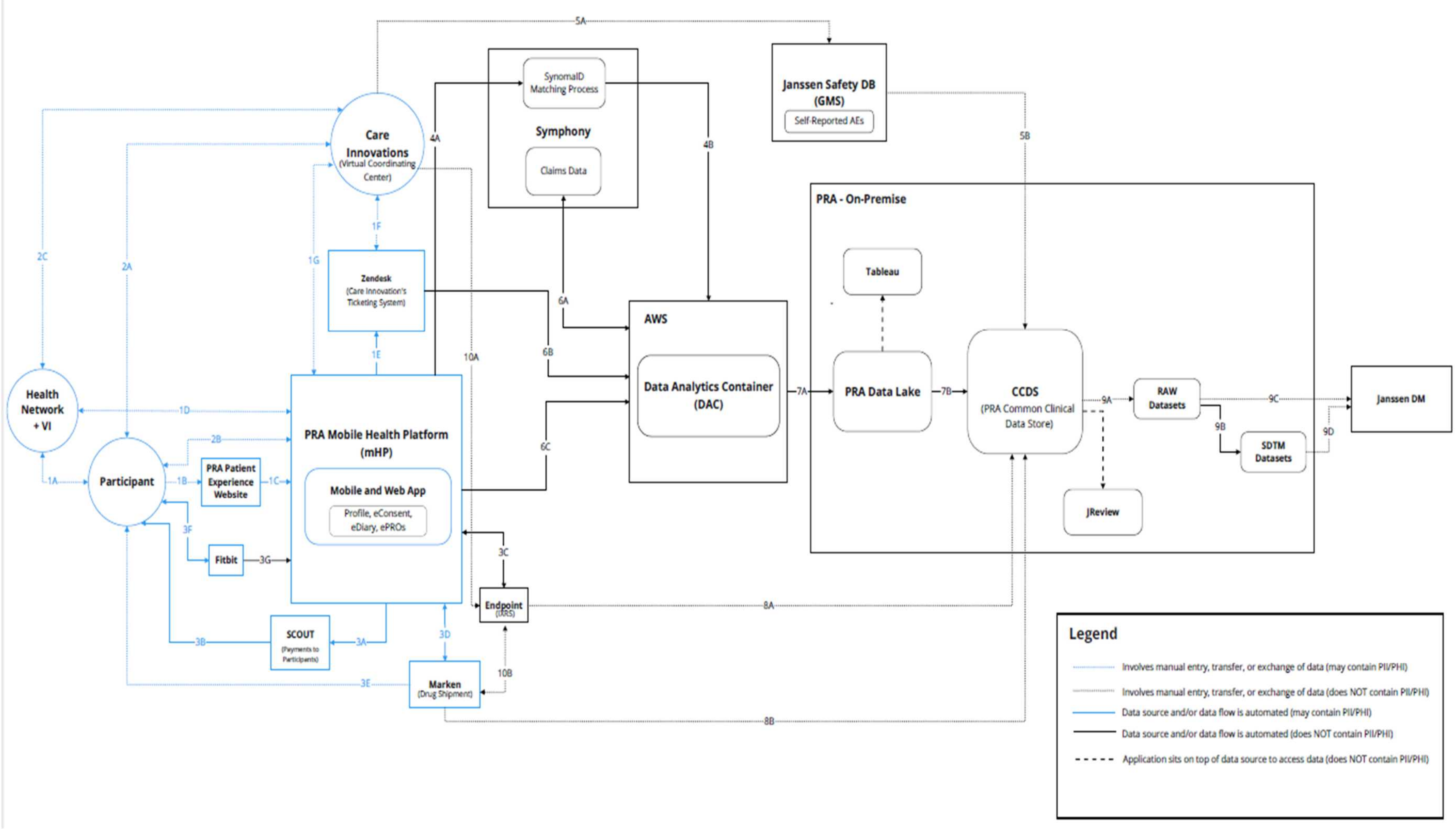

Step	Description
1A	Health Network sends email to prospective study participants.
1B	Pre-consent Participant clicks link to PRA Patient Experience Website and completes Technology Screening form and contact info submission to "opt in".
1C	PRA Patient Experience opt in triggers Patient ID creation in PRA mHP.
1D	Health Network + VI utilize the PRA mHP Web Application to update Pre-Consent Participant demographic info and assess eligibility.
1E	Care Innovations Zendesk Ticketing System automatically pulls information from PRA mHP for Pre-Consent Participants assessed as “Eligible” and creates an assigned open ticket.
1F	Care Innovations interacts with Zendesk Ticketing System to pick up assigned open tickets.
1G	Care Innovations interacts with PRA mHP Web Application to access Pre-Consent Participant contact information to schedule eConsent call.
2A	Care Innovations initiates communication with Pre-consent Participant to schedule eConsent call and verify contact information (mobile number and address).
2B	Pre-consent Participant interacts with PRA mHP Participant Web Application and Mobile Application to complete pre-consent review.
2C	Care Innovations interacts with VI to coordinate and facilitate completion of eConsent call. Post-consent Participant moves to ePRO completion post eConsent call.
3A	PRA mHP sends questionnaire completion details to SCOUT for payment to participants.
3B	SCOUT sends reloadable payment card and payment to consented participants upon completion of study questionnaires.
3C	PRA mHP interacts with Endpoint IRT at Randomization via a two-way database integration.
3D	PRA mHP interacts with Marken's system via a two-way database integration.
3E	Marken schedules directly with Participant to deliver drug to Participant's home. Care Innovations sends study supplies Welcome Kit to Participant.
3F	During Welcome Call with Care Innovations, Participant is guided through the supplies in the Welcome Kit, which includes the Fitbit watch. Participant interacts with Fitbit Mobile Application for Fitbit set-up and wears Fitbit to collect Biodata / ADL data.
3G	Once authorized within the PRA mHP Mobile Application, Fitbit data is retrieved by PRA mHP automatically through Fitbit API.
4A	PRA mHP sends Symphony encrypted, tokenized PII to match consented Participant in PRA mHP with their available medical claims data in Symphony.
4B	Synoma ID Matches identified in Symphony are sent to DAC
5A	Care Innovations manually submits participant-reported AEs directly to Janssen Safety (GMS).
5B	Janssen Safety (GMS) extracts participant-reported AEs and transfers data to PRA for upload into CCDS.
6A	Symphony Claims data is pulled into DAC.
6B	Care Innovations Zendesk ticketing data is sent to DAC via JSON payloads.
6C	PRA mHP data is pulled into DAC.
7A	DAC data is staged and moved into consolidation (CNS) layer, aka "PRA Data Lake".
7B	Consolidated DAC data tables are loaded into CCDS.
8A	Data Transfer from Endpoint is manually loaded into CCDS.
8B	Data Transfer from Marken is manually loaded into CCDS.
9A	RAW SAS datasets are extracted from CCDS for each data delivery.
9B	SDTM Datasets are generated from the RAW data tables for each data delivery.
9C	RAW SAS datasets are manually posted to LSAF for delivery to Janssen.
9D	SDTM SAS datasets are manually posted to LSAF for delivery to Janssen.
10A	Care Innovations manually enters drug resupply request into Endpoint IRT (if applicable)
10B	Endpoint notifies Marken of need for drug resupply (if applicable)

Appendix 6: Patient Global Impression of Change (PGIC)

Appendix 6 provides a representative example of the scale questions that will be used in this study.

Since the start of the treatment you've received in this study, your heart failure symptoms are: (select one response)

1. Very much improved
2. Somewhat improved
3. A little improved
4. No change
5. A little worse
6. Somewhat worse
7. Very much worse

Appendix 7: Patient Global Impression of Severity (PGI-S)

Appendix 7 provides a representative example of the scale question that will be used in this study.

Considering all aspects of your heart failure symptoms right now, would you say your heart failure symptoms are: (select one response)

1. None
2. Mild
3. Moderate
4. Severe
5. Very Severe

Appendix 8: KCCQ Score Instructions

The Kansas City Cardiomyopathy Questionnaire Scoring Instructions

There are 10 summary scores within the KCCQ, which are calculated as follows:

1. PHYSICAL LIMITATION

- Code responses to each of Questions 1a-f as follows:

  Extremely limited = 1
  Quite a bit limited = 2
  Moderately limited = 3 Slightly
  limited = 4
  Not at all limited = 5
  Limited for other reasons or did not do = <*missing value*>

- If at least three of Questions 1a-f are not missing, then compute

  Physical Limitation Score = 100*[(mean of Questions 1a-f actually answered) – 1]/4

  (see footnote at end of this document for explanation of meaning of "actually answered")

2. SYMPTOM STABILITY

- Code the response to Question 2 as follows:

  Much worse = 1

Summary Of Changes in Statistical Analysis Plan

Amendment Page – Summary of Changes (December 30, 2020)

Section #	Original Text/Figure	Amended Text/Figure	Rationale
1.2	Figure 1	Figure 1	Changed entry criterion for KCCQ <80 incorporated
1.4	Added text	After approximately 10 months, the planned enrollment in this study is not expected to reach the target of 1900 participants by the end of January 30, 2021 (the scheduled end of study enrollment window). Instead, the final anticipated enrollment may be in the range of about 400-480 participants. Consequently, a reduction in total sample size of this magnitude will have impact on statistical power at different effect sizes, as shown by the figure below. A large effect size was reported in the recently concluded DEFINE-HF study[17]. Additional KCCQ domains were also reported in this study with robust improvements over baseline[17,18].	Statement about lower than expected sample size enrollment and its impact on statistical power
2.3.2.	Added full analysis set (FAS) definition	This includes all randomized participants who have received at least one dose of study intervention/medication and have at least one post-baseline KCCQ measurement.<br><br>Analyses of the primary, secondary, and exploratory endpoints will be based on the full analysis set (FAS).	Primary, secondary, and exploratory endpoints will be based on FAS (earlier version stated as based on all randomized set)
5.2.2.	Population: all randomized set	Population: FAS - all randomized participants with HF (who either have HFrEF or HFpEF, regardless of T2DM status) with at least one dose of study intervention and at least 1 post-baseline KCCQ measurement	Changed analysis population to FAS
5.2.3.	Added to the analysis method	Mortality in this short-duration trial is expected to be low and to be comparable between groups and will be ignored in the primary analyses of mean differences in changes in KCCQ Total Symptom scores, but can be included in the responder analyses by assigning them to the lowest response (greatest deterioration) category. Should there be a >5% mortality rate and if it is unequally distributed between treatment arms, then joint modeling of survival and health status will be performed. A win-ratio approach[19,20] may also be considered by analyzing the KCCQ-TSS score as a composite, rank-based outcome, incorporating participant vital status at 12	Added to the analysis method regarding missing data due to deaths

		weeks along with a change in score from baseline to 12 weeks in surviving participants. This essentially is the rank analysis of covariance method, with a corresponding win ratio used to estimate the magnitude of treatment effect.	
5.3.2.	Population: all randomized set	Population: FAS - all randomized participants with HF (who either have HFrEF or HFpEF, regardless of T2DM status) with at least one dose of study intervention and at least 1 post-baseline KCCQ measurement	Changed analysis population to FAS
5.3.3.	Added to analysis method	The first key secondary endpoint will be analyzed following the same approach as employed for the primary effectiveness endpoint (i.e. using an MMRM model).	Added to analysis method regarding daily step counts from two-sample t-test to MMRM analysis
References	Added references	Nassif, M. et al. (2019). Dapagliflozin Effects on Biomarkers, Symptoms, and Functional Status in Patients With Heart Failure With Reduced Ejection Fraction - The DEFINE-HF Trial. *Circulation*, Vol. 140, No. 18, 1463-1476<br><br>Bhatt, D. et al. (2020). Sotagliflozin in Patients with Diabetes and Recent Worsening Heart Failure. *N Engl J Med*, 2020; Nov 16<br><br>Gasparyan, S. et al. (2019). Adjusted Win Ratio with Stratification: Calculation Methods and Interpretation, *Stat.ME*<br><br>Wang, D. and Pocock, S. (2016). A win-ratio approach to comparing continuous non-normal outcomes in clinical trials, *Pharmaceutical Statistics*, online 11 March 2016	Added more references pertaining to sample size section and analysis methods such as win-ratio
