# Supplementary material for: The SGLT2 inhibitor canagliflozin in heart failure: the CHIEF-HF remote, patient-centered randomized trial
Source: Nat Med. 2022 Feb 28;28(4):809–13. doi: 10.1038/s41591-022-01703-8 (PMC9018422; doi:10.1038/s41591-022-01703-8)
Supplement: Supplementary file 2 — Reporting Summary [file 41591_2022_1703_MOESM2_ESM.pdf]

nature portfolio

Corresponding author(s): John Spertus
Last updated by author(s): Jan 7, 2022

Reporting Summary

Nature Portfolio wishes to improve the reproducibility of the work that we publish. This form provides structure for consistency and transparency in reporting. For further information on Nature Portfolio policies, see our Editorial Policies and the Editorial Policy Checklist.

Statistics

For all statistical analyses, confirm that the following items are present in the figure legend, table legend, main text, or Methods section.

n/a	Confirmed	
☐	☒	The exact sample size (*n*) for each experimental group/condition, given as a discrete number and unit of measurement
☐	☒	A statement on whether measurements were taken from distinct samples or whether the same sample was measured repeatedly
☐	☒	The statistical test(s) used AND whether they are one- or two-sided<br>*Only common tests should be described solely by name; describe more complex techniques in the Methods section.*
☐	☒	A description of all covariates tested
☐	☒	A description of any assumptions or corrections, such as tests of normality and adjustment for multiple comparisons
☐	☒	A full description of the statistical parameters including central tendency (e.g. means) or other basic estimates (e.g. regression coefficient) AND variation (e.g. standard deviation) or associated estimates of uncertainty (e.g. confidence intervals)
☐	☒	For null hypothesis testing, the test statistic (e.g. *F*, *t*, *r*) with confidence intervals, effect sizes, degrees of freedom and *P* value noted<br>*Give P values as exact values whenever suitable.*
☒	☐	For Bayesian analysis, information on the choice of priors and Markov chain Monte Carlo settings
☒	☐	For hierarchical and complex designs, identification of the appropriate level for tests and full reporting of outcomes
☐	☒	Estimates of effect sizes (e.g. Cohen's *d*, Pearson's *r*), indicating how they were calculated

Our web collection on statistics for biologists contains articles on many of the points above.

Software and code

Policy information about availability of computer code

Data collection	As described in the Methods paper (Circ Heart Fail 2021;14:e007767), a custom-built app was used for data collection.
Data analysis	SAS Version 9.4 was used

For manuscripts utilizing custom algorithms or software that are central to the research but not yet described in published literature, software must be made available to editors and reviewers. We strongly encourage code deposition in a community repository (e.g. GitHub). See the Nature Portfolio guidelines for submitting code & software for further information.

Data

Policy information about availability of data

All manuscripts must include a data availability statement. This statement should provide the following information, where applicable:
- Accession codes, unique identifiers, or web links for publicly available datasets
- A description of any restrictions on data availability
- For clinical datasets or third party data, please ensure that the statement adheres to our policy

Requests access to the study data can be made through Yale Open Data Access (YODA) at http://yoda.yale.edu 18 months after completion of the trial, which is March 1, 2022 (last contact for extended follow-up).

Field-specific reporting

Please select the one below that is the best fit for your research. If you are not sure, read the appropriate sections before making your selection.

☒ Life sciences ☐ Behavioural & social sciences ☐ Ecological, evolutionary & environmental sciences

For a reference copy of the document with all sections, see nature.com/documents/nr-reporting-summary-flat.pdf

Life sciences study design

All studies must disclose on these points even when the disclosure is negative.

Sample size	The original sample size was to include 1,900 randomized participants, but shifting priorities of the sponsor (Janssen Scientific Affairs, LLC) led to administrative closing of the study to enrollment on February 12, 2021. This decision was made without an interim analysis of the data or recalculation of sample sizes and power and was in consultation with the academic Steering Committee.<br>The original sample size, as noted in the SAP was based on achieving >90% power to detect a 3-point difference between arms. Ultimately, 476 were randomized, 28 immediately dropped out without completing a follow-up KCCQ, and 448 were included in the final analyses.
Data exclusions	Twenty-one participants (4.4%) withdrew immediately without every taking a dose of the study medication and 7 (1.5%) had no follow-up KCCQ scores and were excluded from the final analyses.
Replication	The results were replicated by an independent analytic center after database lock and were 100% successful.
Randomization	Patients were randomized, stratified by heart failure type (HFpEF vs. HFrEF).
Blinding	Both patients and site PIs were blinded to treatment.

Reporting for specific materials, systems and methods

We require information from authors about some types of materials, experimental systems and methods used in many studies. Here, indicate whether each material, system or method listed is relevant to your study. If you are not sure if a list item applies to your research, read the appropriate section before selecting a response.

Materials & experimental systems

n/a	Involved in the study	
☒	☐	Antibodies
☒	☐	Eukaryotic cell lines
☒	☐	Palaeontology and archaeology
☒	☐	Animals and other organisms
☐	☒	Human research participants
☐	☒	Clinical data
☒	☐	Dual use research of concern

Methods

n/a	Involved in the study	
☒	☐	ChIP-seq
☒	☐	Flow cytometry
☒	☐	MRI-based neuroimaging

Human research participants

Policy information about studies involving human research participants

Population characteristics	In brief, eligibility required a diagnosis of heart failure (HFrEF with an EF<40% and a primary or 2 HF diagnoses in any position within 18 months; HFpEF with an EF<=40% and similar diagnosis codes as HFrEF and treatment with a loop diuretic or mineralocorticoid receptor antagonist), a Kansas City Cardiomyopathy Overall (KCCQ) Summary score of <=80, English speaking, sole possession of an Apple iPhone 6 or later or a Samsung Galaxy, willingness to wear a Fitbit device and to provide informed consent. Key exclusion criteria included the use of an SGLT2i within 3 months, a history of diabetic ketoacidosis or Type 1 diabetes, and eGFR <30ml/min. Among the 448 patients included in the primary analysis, the mean age was 63.4 (SD=13.3; range of 20 to 94), 84% were White, 45% women, 28% of participants had Type 2 diabetes and 60% had HFpEF.
Recruitment	The different recruitment sites used different strategies for identifying patients to participate, including email, patient portals through the health system's electronic medical record, phone calls and contacting providers before a scheduled visit. Those patients who expressed interest in participating in the study then visited a study-specific website to learn more and, if interested, to undergo final screening and undergo informed consent. There is no way to estimate what selection biases might have been introduced.
Ethics oversight	A central Institutional Review Board (Advarra, Columbia MD) was used for this study.

Note that full information on the approval of the study protocol must also be provided in the manuscript.

Clinical data

Policy information about clinical studies

All manuscripts should comply with the ICMJE guidelines for publication of clinical research and a completed CONSORT checklist must be included with all submissions.

Clinical trial registration	NCT04252287, which can be accessed at https://clinicaltrials.gov/ct2/show/NCT04252287
Study protocol	This will be uploaded as supplementary material
Data collection	All data were collected by patients at their homes, through an app, without any in-person visits. Each patient participated in the primary randomization phase of the study for 12 weeks. Enrollment began on March 26, 2020 and ended on February 12, 2021
Outcomes	The primary outcome was the Kansas City Cardiomyopathy Questionnaire Total Symptom Score. Secondary outcomes included the other domains of the KCCQ and average step counts on a Fitbit.
